# Beyond Variant Evolution: Structurally and Functionally Conserved Regions in the 5′UTR of SARS-CoV-2 as Resilient Antiviral Targets

**DOI:** 10.3390/biomedicines14030622

**Published:** 2026-03-10

**Authors:** Andrea Masotti

**Affiliations:** Bambino Gesù Children’s Hospital-IRCCS, Research Laboratories, 00146 Rome, Italy; andrea.masotti@opbg.net; Tel.: +39-06-6859-2650

**Keywords:** 5′UTR, COVID-19, SARS-CoV-2, miRNAs, siRNAs

## Abstract

**Background**: Severe acute respiratory syndrome coronavirus 2 (SARS-CoV-2) is a positive-sense RNA virus, and its genome includes a highly conserved 5′ untranslated region (5′UTR). This region contains the so-called ‘leader sequence’, a crucial genomic region responsible for the viral replication and the synthesis of all subgenomic RNAs (sgRNAs). It has been demonstrated that targeting highly conserved genomic regions is essential for developing broad-spectrum antiviral therapies that resist viral mutation and evasion. **Hypothesis**: Given the high level of nucleotide homology between SARS-CoV and SARS-CoV-2, particularly in essential regions like the 5′UTR, the identification of a perfect sequence alignment across SARS-CoV-2 variants within this conserved region would provide a robust, mutation-resistant target for novel RNA-based drugs, such as small interfering RNAs (siRNAs) or microRNAs (miRNAs). **Materials and Methods**: Sequence alignment was performed across the different SARS-CoV-2 strains (i.e., the different variants that have appeared so far) to identify conserved genomic areas, leading to the selection of potential target sites for antiviral molecules. Specifically, computational analyses were utilized to map available binding sites for human miRNAs within the SARS-CoV-2 5′UTR. **Results:** Comparative alignments revealed that the leader sequence/5′UTR region is highly stable and conserved in all the considered SARS-CoV-2 sequences, representing a common therapeutic target across different variants and strains. **Discussion:** The perfect alignment observed in the 5′UTR confirms that this region is a highly critical target, less prone to mutations in all the considered variants. This property makes the region ideal for therapeutic intervention using non-coding RNAs. If endogenous miRNAs were found to bind this region (e.g., miR-638, miR-3150b-3p, etc.) and promote viral replication similarly to mechanisms observed in viruses like hepatitis C virus (HCV), their activity could be inhibited using chemically modified antisense analogs, such as locked nucleic acid (LNA) oligonucleotides.

## 1. Introduction

Severe acute respiratory syndrome coronavirus 2 (SARS-CoV-2) has infected almost 780 million people worldwide and led to more than 7 million deaths as of December 2025. Despite the success of vaccines, the continuous development of novel therapeutic strategies to complement vaccination efforts is needed, particularly considering the emergence of variants of concern (VOCs) that exhibit high mutation rates [1,2]. RNA interference (RNAi), mediated by small interfering RNAs (siRNAs) and microRNAs (miRNAs), has emerged as a promising approach that is able to silence viral genes and their expression with high precision [1,3,4]. The use of synthetic siRNAs or exogenous miRNAs represents an attractive modality for antiviral treatment due to their ability to induce post-transcriptional gene silencing, leading to the degradation or suppression of viral RNA [2,5].

The 5′ untranslated region (5′UTR) of the SARS-CoV-2 genome, which contains the crucial leader sequence, represents a particularly relevant target [6,7]. This region is crucial for viral replication and transcription, as the leader sequence is consistently incorporated into all subgenomic RNAs [6,7,8].

It has been previously reported that mutant plasmids with different 5′UTR lengths have different properties: whereas the mutant plasmid lacking the region 1–36 did not display a markedly different activity, the plasmid lacking almost all of the UTR (i.e., the 1–222 region) completely abolished the SARS-CoV promoter activity in human cells [9,10]. It has long been known that subgenomic mRNAs lacking the 5′ leader sequence are not able to replicate, and one of the possible explanations is the presence of at least four stem loops located in the 5′-end region of the coronavirus genome; these secondary structures are actively implicated in viral replication and transcription [11].

Prior research against the related SARS-CoV demonstrated that RNAi successfully inhibited viral replication by targeting the leader sequence and other regions [12,13]. This was also reported by my group in recent years [5], where we calculated that the 5′UTR exhibited approximately 88.76% similarity between SARS-CoV and SARS-CoV-2.

Many in vitro studies have shown that small interfering RNAs (siRNAs) can suppress viral replication by targeting distinct regions of the SARS-CoV genome and reducing viral messenger RNA production. Among the regions tested, siRNAs targeting the Spike protein proved most potent, though sequences directed against the leader sequence, transcription regulatory sequence (TRS), and 3′ untranslated region (3′UTR) also successfully prevented viral infection in Vero-E6 cell cultures [13]. In another work, Li and colleagues demonstrated that a leader sequence-specific siRNA similarly achieved effective viral inhibition in Vero-E6 cells [12]. While this strategy did not constitute a complete antiviral therapy—as treated animals still exhibited clinical symptoms—it nevertheless represents a viable approach for reducing viral burden and disease severity.

A few years ago, we also emphasized the highly conserved genomic region within the first 90 nucleotides that encompasses the transcription regulatory sequence (TRS) (nucleotides 40 to 85) and is identical in both SARS-CoV and the initial SARS-CoV-2 isolates analyzed, thus establishing a highly stable therapeutic target [5]. siRNAs targeting the leader sequence have already demonstrated perfect homology against the Delta variant and maintained activity against the Alpha variant, confirming the stability of this target site [7]. This observed genomic stability reinforces the 5′UTR as a robust, broad-spectrum therapeutic target, highly suitable for the design of mutation-resistant non-coding RNA-based drugs, such as siRNAs and antisense oligonucleotides (ASOs) [5,7,14].

Another viable strategy is to target the 5′UTR regions of the viral RNA where the nucleotides form characteristic secondary structure motifs called pseudoknots [15,16]. For several years, viral RNA pseudoknots have been recognized as widespread motifs with different functions in gene expression and viral genome replication [17]. One example is represented by the frameshift stimulation element (FSE) in SARS-CoV-2, that the virus employs to express fundamental proteins such as RNA-dependent RNA polymerase (RdRp) and other essential non-structural proteins [18].

Given the many SARS-CoV-2 genomic variants that have appeared in the last few years and are continuously arising [1], the novelty of the present opinion resides in the demonstration that this specific, highly conserved TRS region that I identified not only is a valuable targetable region but also maintains perfect sequence alignment across the multiple subsequent and divergent SARS-CoV-2 variants that have appeared so far [14,19]. Therefore, it is reasonable to argue that the structural and functional constraints of specific regions of the 5′UTR will ensure conservation across forthcoming SARS-CoV-2 variants, rendering them resilient and exploitable targets for antiviral interventions.

## 2. Materials and Methods

### 2.1. Sequence Acquisition

The reference genome sequence for SARS-CoV-2, specifically the Wuhan-Hu-1 strain (NCBI accession NC_045512 or NC_045512.2), was acquired for use as the primary template. Comparative sequence analyses also utilized the SARS-CoV reference genome (NCBI NC_004718 or AY310120.1). To extend the conservation analysis to circulating viral lineages, genomic sequences for multiple SARS-CoV-2 variants, including the Alpha (B.1.1.7), Beta (B.1.351), Kappa (B.1.617.1), Delta (B.1.617.2), Epsilon (B.1.427/B.1.429), Gamma (P.1), Eta (B.1.525), Iota (B.1526), Omicron (B.1.1.529, BA.1, BA.2, BA.3, BA.4 and BA.5), Mu (B.1.621), Zeta (P.2), Theta (P.3) and Lambda (C.37) variants, were obtained from the public repository Global Initiative of Sharing All Influenza Data (GISAID) by downloading all sequences available up to June 2025.

Some of these variants were also reported in the literature [2,7,14,19,20].

### 2.2. Comparative Sequence Alignment and Conservation Mapping

**Figure 1:**
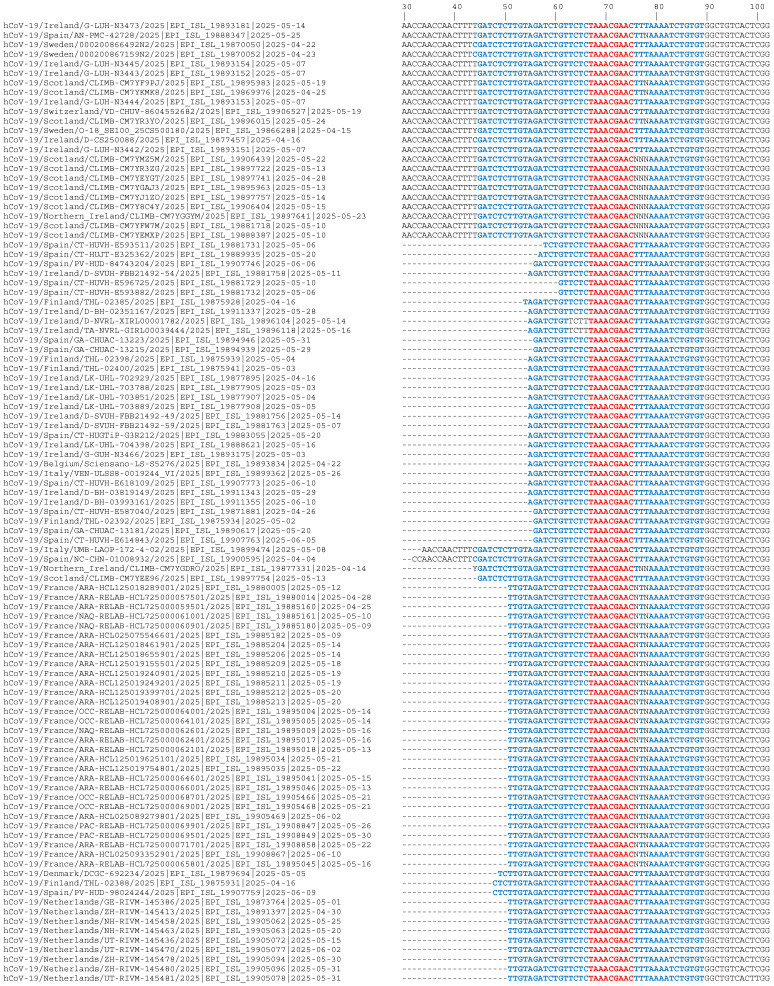
The most relevant genomic sequences of the SARS-CoV-2 variants, where the conserved region is outlined in bold (blue and red), with the leader sequence colored in red. All the genomes considered in this paper have been reported in Supplementary Figures S1 and S2 for clarity reasons.

Multiple sequence alignment (MSA) was performed to compare the downloaded full-length genomic RNA sequences of SARS-CoV-2 variants. The Clustal Omega alignment software (1.2.4) was employed for the alignment. Focus was placed specifically on the conservation of the 5′UTR, including the crucial leader sequence and the transcription regulatory sequence (TRS) previously identified [5]. Therefore, the initial part of the 5′UTR region ranging from 0 to 500 bases was trimmed and aligned (Supplementary Figure S1). The alignment gave a conserved pattern starting from base 30, so that the further comparison was made from 30 to 110 (Supplementary Figure S2). To restrict the analysis and focus on the leader sequence, selected sequences were centered around positions 30–100 (Figure 1).

### 2.3. Secondary Structure Prediction and Pseudoknot Formation

The formation of pseudoknots in the 5′UTR genomic region was assessed by running the online tool “RNAstructure”, a web server for RNA secondary structure prediction (https://rna.urmc.rochester.edu/RNAstructureWeb/Servers/ProbKnot/ProbKnot.html accessed on 26 February 2026). The formation of secondary structures and pseudoknots is reported in Supplementary Figure S3.

## 3. Results

Figure 1 presents a sequence alignment focusing on a portion of the 5′UTR of SARS-CoV-2 variants, specifically spanning nucleotides 30 through 102. The alignment compares isolates that circulated from the initial pandemic burden to June 2025. In particular, the total number of analyzed sequences was 140, and the global mean identity was very high (98.09%), as shown in Table 1. Results indicate a high homogeneity in the majority of countries (i.e., France, Ireland, Netherlands), with an internal identity greater than 99%, suggesting a local circulation of very similar variants.

However, Germany and Spain displayed a higher internal variability (minimal identity of 71.34% and 76.37%, respectively), suggesting the presence of multiple distinct lineages. Northern Ireland has the lowest mean identity relative to other countries (94.89%), which is suggestive of peculiar genomic characteristics compared to the European mean data.

**Figure 2:**
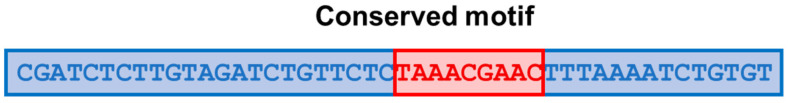
The most conserved motif (blue) in the 5′UTR genomic region of SARS-CoV-2. The transcription-regulatory region (TRS) is indicated in red.

The alignment demonstrates a region of substantial conservation between these distinct variants (Figure 2). This region is significant because it encompasses the TRS (outlined in blue) and the leader sequence (outlined in red), which are crucial for viral replication and transcription and remain largely conserved in the later-stage variants analyzed (Figure 1). A larger portion of the genomic region and those variants not selected for clarity reasons have been reported in Supplementary Figures S1 and S2, but the conclusions that I reached are essentially the same. The full percent identity matrix is available as Table S1 in Supplementary Materials.

Additionally, the 5′UTR region is also able to form pseudoknots (Supplementary Figure S3) that could represent regions potentially targetable by ASOs or siRNAs/miRNAs, thus increasing further the therapeutic arsenal.

## 4. Discussion

The ongoing nature of the COVID-19 pandemic, coupled with the frequent emergence of new VOCs that demonstrate high mutation rates, requires alternative therapeutic strategies that could complement vaccination efforts [1,2,19]. Viral mutations, particularly in highly immunogenic genomic regions like the Spike (S) protein, can reduce the efficacy of neutralizing antibodies and certain small-molecule antivirals, facilitating viral evasion [2,7,14].

RNA interference (RNAi), leveraging highly specific synthetic siRNAs or exogenous miRNAs antisense oligos, offers a promising approach owing to the targeted silencing and degradation of viral RNA transcripts [1,5,21]. For RNAi-based drugs to maintain efficacy across a continually evolving spectrum of viral strains, they must target sequences essential for viral fitness that are inherently resistant to mutation [14,22]. However, one should not forget that RNAi suffers from some drawbacks such as the lack of efficient delivery to respiratory tissue, potential off-target effects, activation of the innate immune response, and other potential regulatory barriers [23] that in some cases can be adequately approached and solved by using modified oligonucleotides (personal unpublished data).

The 5′UTR of the SARS-CoV-2 genome, in particular the region that contains the TRS and the leader sequence, has been established as a valuable target as it satisfies these criteria [5,6]. The leader sequence is essential for viral replication and transcription. In fact, it is incorporated into all sgRNAs required for the synthesis of structural proteins [7,8].

Our initial study reported the genetic stability of this region and outlined that the 5′UTR shares approximately 88.76% similarity between SARS-CoV and SARS-CoV-2, with key segments such as the TRS being identical in the considered genomes [5]. The crucial importance of the present study lies in validating that this high degree of conservation persists even among widely circulating and more recent variants. The alignment data presented in Figure 1 for isolates circulating from 2020 to 2025 confirms that the core sequence in the 5′UTR remains highly stable and conserved despite the increased number of Alpha, Delta, and various Omicron subvariants. Our results emphasize the role of the 5′UTR as a potential mutation-resistant therapeutic target, and a specific region between 30 and 100 nucleotides as the most important. The main key advantages for therapeutic development using siRNAs and miRNAs are manifold: (i) by targeting an identical sequence across all circulating variants, the resulting RNAi therapeutic is inherently ‘broad-spectrum’, offering protection not only against known variants but also potentially against future coronaviruses; (ii) targeting conserved sites outside hypervariable regions (like Spike) greatly reduces the chance of emerging viral escape mutants, thereby enhancing the long-term viability of the drug; (iii) a potent mechanism of action: the leader sequence is present on both the genomic RNA and all sgRNAs, so that by targeting this region one can ensure the simultaneous inhibition of replication and the synthesis of structural proteins, leading to a profound reduction in viral replication and load; (iv) the conservation supports strategies exploiting miRNA targeting. If host miRNAs (like miR-4507 or miR-638, which are highly expressed in lung tissue) bind the conserved 5′UTR to promote viral replication, the design of antisense molecules (such as LNA-based GapmeRs, or antago-miRs) to sequester those miRNAs could represent an indirect yet broad-spectrum antiviral strategy.

## 5. Conclusions

Sustained targeting of these structurally and functionally conserved regions, combined with advances in computational design and delivery systems, positions RNAi as a leading strategy for the development of pan-coronavirus therapeutics with intrinsic resilience to viral evolution.

Finally, the evolutionary and functional constraints governing the 5′UTR provide strong grounds to argue that these conserved regions will be retained across forthcoming SARS-CoV-2 variants, ensuring the sustained relevance of the findings presented here as durable foundations for antiviral target design.

## Figures and Tables

**Table 1:** Country analysis. The table summarizes the sequences per country, showing internal cohesion (intra-country identity) and the mean similarities with the sequences of other countries.

Country	Sequence Number	Intra-Country Identity (%)	Intra-Country Minimal Identity (%)	Mean Identity vs. Others (%)
France	35	99.99	99.79	98.57
Spain	23	97.85	76.37	97.98
Scotland	22	98.99	89.65	98.48
Ireland	21	99.94	99.6	98.69
Netherlands	9	99.96	99.8	98.87
Germany	6	82.8	71.34	86.9
Finland	5	99.84	99.6	98.84
Denmark	3	99.58	99.36	98.81
England	3	100	100	99.02
Northern Ireland	3	90.8	85.92	94.89
Sweden	3	99.87	99.8	99.11
Italy	2	100	100	98.95
Wales	2	100	100	99.02
Belgium	1	100 ^1^	100	98.95
Canary Islands	1	100 ^1^	100	98.9
Switzerland	1	100 ^1^	100	99.1

^1^ For countries with only one sequence, the intra-country identity is set to 100% by default.

## Data Availability

The original contributions presented in this study are included in the article/Supplementary Material. Further inquiries can be directed to the corresponding author.

## Supplementary Materials

The following supporting information can be downloaded at: https://www.mdpi.com/article/10.3390/biomedicines14030622/s1, Figure S1: All of the SARS-CoV-2 genomes considered in this paper. The highly conserved region is outlined in bold (blue and red), and the transcription-regulatory region (TRS) is colored in red. Figure S2. All of the SARS-CoV-2 genomes considered in this paper. The highly conserved region is outlined in bold (blue and red), and the transcription-regulatory region (TRS) is colored in red. Figure S3. The representation of the 5’UTR region of the SARS-CoV-2 genome (from 1 to 265 bases) is considered in this paper. Table S1.

## Abbreviations

The following abbreviations are used in this manuscript:

SARS-CoV-2	severe acute respiratory syndrome coronavirus 2
5′UTR	5′ untranslated region
sgRNAs	subgenomic RNAs
siRNAs	small interfering RNAs
miRNAs	microRNAs
HCV	hepatitis C virus
LNA	locked nucleic acid
VOCs	variants of concern
TRS	transcription regulatory sequence
ASOs	antisense oligonucleotides
GISAID	Global Initiative of Sharing All Influenza Data
MSA	multiple sequence alignment
