# Supplementary material for: Beyond Variant Evolution: Structurally and Functionally Conserved Regions in the 5′UTR of SARS-CoV-2 as Resilient Antiviral Targets
Source: Biomedicines. 2026 Mar 10;14(3):622. doi: 10.3390/biomedicines14030622 (PMC13024171; doi:10.3390/biomedicines14030622)
Supplement: Supplementary file 1 [file biomedicines-14-00622-s001.zip › biomedicines-4094480-supplementary.pdf]

Supplementary Figure S1. All of the SARS-CoV-2 genomes (from 1 to 500 bases) considered in this paper. Homology matrix is displayed separately.

```
hCoV-19/Ireland/D-SVUH-FBA98650-31/2025|EPI_ISL_19866548|2025-05-04          ------------------------------------------------------------ 0
hCoV-19/Spain/PV-HUB-5628663/2025|EPI_ISL_19908839|2025-05-16                ------------------------------------------------------------ 0
hCoV-19/Spain/PV-HUB-41587498/2025|EPI_ISL_19908930|2025-05-20               ------------------------------------------------------------ 0
hCoV-19/Spain/PV-HUB-56291851/2025|EPI_ISL_19908970|2025-05-31               ------------------------------------------------------------ 0
hCoV-19/Northern_Ireland/CLIMB-CM7YR8I6/2025|EPI_ISL_19877334|2025-04-23     ------------------------------------------------------------ 0
hCoV-19/Germany/NW-RKI-I-1148136/2025|EPI_ISL_19871950|2025-04-23            ------------------------------------------------------------ 0
hCoV-19/Germany/NW-RKI-I-1148141/2025|EPI_ISL_19871955|2025-04-23            ------------------------------------------------------------ 0
hCoV-19/Germany/BW-RKI-I-1148164/2025|EPI_ISL_19884987|2025-04-17            ------------------------------------------------------------ 0
hCoV-19/Scotland/CLIMB-CM7YFGW7/2025|EPI_ISL_19897691|2025-05-24             ------------------------------------------------------------ 0
hCoV-19/Ireland/G-LUH-N3473/2025|EPI_ISL_19893181|2025-05-14                 ---------ATACCTTCCCAGGTAACAAACCAACCAACTTTTGATCTCTTGTAGATCTGT 51
hCoV-19/Spain/AN-PMC-42728/2025|EPI_ISL_19888347|2025-05-25                  --------TATACCTTCCCAGGTAACAAACCAACTAACTTTTGATCTCTTGTAGATCTGT 52
hCoV-19/Sweden/000200866492N2/2025|EPI_ISL_19870050|2025-04-22               --AAGGTTTATACCTTCCCAGGTAACAAACCAACCAACTTTCGATCTCTTGTAGATCTGT 58
hCoV-19/Sweden/000200867159N2/2025|EPI_ISL_19870052|2025-04-23               --AAGGTTTATACCTTCCCAGGTAACAAACCAACCAACTTTCGATCTCTTGTAGATCTGT 58
hCoV-19/Ireland/G-LUH-N3445/2025|EPI_ISL_19893154|2025-05-07                 --AAGGTTTATACCTTCCCAGGTAACAAACCAACCAACTTTTGATCTCTTGTAGATCTGT 58
hCoV-19/Ireland/G-LUH-N3443/2025|EPI_ISL_19893152|2025-05-07                 ----GGTTTATACCTTCCCAGGTAACAAACCAACCAACTTTTGATCTCTTGTAGATCTGT 56
hCoV-19/Scotland/CLIMB-CM7YF9PJ/2025|EPI_ISL_19895983|2025-05-19             -----GTTTATACCTTCCCAGGTAACAAACCAACCAACTTTTGATCTCTTGTAGATCTGT 55
hCoV-19/Scotland/CLIMB-CM7YKMK8/2025|EPI_ISL_19869976|2025-04-25             ------TTTATACCTTCCCAGGTAACAAACCAACCAACTTTTGATCTCTTGTAGATCTGT 54
hCoV-19/Ireland/G-LUH-N3444/2025|EPI_ISL_19893153|2025-05-07                 ------TTTATACCTTCCCAGGTAACAAACCAACCAACTTTTGATCTCTTGTAGATCTGT 54
hCoV-19/Switzerland/VD-CHUV-8604552682/2025|EPI_ISL_19906527|2025-05-19      -AAAGGTATAAACCTTCCCAGGTAACAAACCAACCAACTTTTGATCTCTTGTAGATCTGT 59
hCoV-19/Scotland/CLIMB-CM7YR3YO/2025|EPI_ISL_19896015|2025-05-24             TAAAGGTTTATACCTTCCCAGGTAACAAACCAACCAACTTTTGATCTCTTGTAGATCTGT 60
hCoV-19/Sweden/O-18_SE100_25CS500180/2025|EPI_ISL_19866288|2025-04-15        TAAAGGTTTATACCTTCCCAGGTAACAAACCAACCAACTTTYGATCTCTTGTAGATCTGT 60
hCoV-19/Ireland/D-CS250088/2025|EPI_ISL_19877457|2025-04-16                  TAAAGGTTTATACCTTCCCAGGTAACAAACCAACCAACTTTCGATCTCTTGTAGATCTGT 60
hCoV-19/Ireland/G-LUH-N3442/2025|EPI_ISL_19893151|2025-05-07                 TAAAGGTTTATACCTTCCCAGGTAACAAACCAACCAACTTTTGATCTCTTGTAGATCTGT 60
hCoV-19/Scotland/CLIMB-CM7YMZ5M/2025|EPI_ISL_19906439|2025-05-22             -AAAGGTTTATACCTTCCCAGGTAACAAACCAACCAACTTTTGATCTCTTGTAGATCTGT 59
hCoV-19/Scotland/CLIMB-CM7YR3ZG/2025|EPI_ISL_19897722|2025-05-13             --AAGGTTTATACCTTCCCAGGTAACAAACCAACTAACTTTTGATCTCTTGTAGATCTGT 58
hCoV-19/Scotland/CLIMB-CM7YEYGT/2025|EPI_ISL_19897741|2025-04-28             --AAGGTTTATACCTTCCCAGGTAACAAACCAACCAACTTTTGATCTCTTGTAGATCTGT 58
hCoV-19/Scotland/CLIMB-CM7YGAJ3/2025|EPI_ISL_19895963|2025-05-13             -------------CTTCCCAGGTAACAAACCAACCAACTTTTGATCTCTTGTAGATCTGT 47
hCoV-19/Scotland/CLIMB-CM7YJ1ZO/2025|EPI_ISL_19897757|2025-05-14             ------------CCTTCCCAGGTAACAAACCAACCAACTTTTGATCTCTTGTAGATCTGT 48
hCoV-19/Scotland/CLIMB-CM7Y8C4Y/2025|EPI_ISL_19906404|2025-05-15             -----GTTTATACCTTCCCAGGTAACAAACCAACCAACTTTTGATCTCTTGTAGATCTGT 55
hCoV-19/Northern_Ireland/CLIMB-CM7YGGYM/2025|EPI_ISL_19897641|2025-05-23     ----------TACCTTCCCAGGTAACAAACCAACCAACTTTTGATCTCTTGTAGATCTGT 50
hCoV-19/Scotland/CLIMB-CM7YFW7M/2025|EPI_ISL_19881718|2025-05-10             -------TTATACCTTCCCAGGTAACAAACCAACCAACTTTTGATCTCTTGTAGATCTGT 53
hCoV-19/Scotland/CLIMB-CM7YEMXP/2025|EPI_ISL_19888387|2025-05-10             --------TATACCTTCCCAGGTAACAAACCAACCAACTTTTGATCTCTTGTAGATCTGT 52
hCoV-19/Spain/CT-HUVH-E593511/2025|EPI_ISL_19881731|2025-05-06               -------------------------------------------------------TCTGT 5
hCoV-19/Spain/CT-HUJT-E325362/2025|EPI_ISL_19889935|2025-05-20               ------------------------------------------------------ATCTGT 6
hCoV-19/Spain/PV-HUD-84743204/2025|EPI_ISL_19907746|2025-06-06               -----------------------------------------------------GATCTGT 7
hCoV-19/Ireland/D-SVUH-FBB21492-54/2025|EPI_ISL_19881758|2025-05-11          ----------------------------------------------------AGATCTGT 8
hCoV-19/Spain/CT-HUVH-E596725/2025|EPI_ISL_19881729|2025-05-10               ----------------------------------------------------------GT 2
hCoV-19/Spain/CT-HUVH-E593882/2025|EPI_ISL_19881732|2025-05-06               ----------------------------------------------------------GT 2
hCoV-19/Finland/THL-02385/2025|EPI_ISL_19875928|2025-04-16                   ---------------------------------------------------TAGATCTGT 9
hCoV-19/Ireland/D-BH-02351167/2025|EPI_ISL_19911337|2025-05-28               ----------------------------------------------------AGATCTGT 8
hCoV-19/Ireland/D-NVRL-XIRL00001782/2025|EPI_ISL_19896104|2025-05-14         ----------------------------------------------------AGATCTGT 8
hCoV-19/Ireland/TA-NVRL-GIRL00039444/2025|EPI_ISL_19896118|2025-05-16        ----------------------------------------------------AGATCTGT 8
hCoV-19/Spain/GA-CHUAC-13223/2025|EPI_ISL_19894946|2025-05-31                -----------------------------------------------------GATCTGT 7
hCoV-19/Spain/GA-CHUAC-13215/2025|EPI_ISL_19894939|2025-05-29                -----------------------------------------------------GATCTGT 7
hCoV-19/Finland/THL-02398/2025|EPI_ISL_19875939|2025-05-04                   ----------------------------------------------------AGATCTGT 8
hCoV-19/Finland/THL-02400/2025|EPI_ISL_19875941|2025-05-03                   ----------------------------------------------------AGATCTGT 8
hCoV-19/Ireland/LK-UHL-702929/2025|EPI_ISL_19877895|2025-04-16               ----------------------------------------------------AGATCTGT 8
hCoV-19/Ireland/LK-UHL-703788/2025|EPI_ISL_19877905|2025-05-03               ----------------------------------------------------AGATCTGT 8
```

```
hCoV-19/Ireland/LK-UHL-703851/2025|EPI_ISL_19877907|2025-05-04   ----------------------------------------------------AGATCTGT 8
hCoV-19/Ireland/LK-UHL-703889/2025|EPI_ISL_19877908|2025-05-05   ----------------------------------------------------AGATCTGT 8
hCoV-19/Ireland/D-SVUH-FBB21492-49/2025|EPI_ISL_19881756|2025-05-14   ----------------------------------------------------AGATCTGT 8
hCoV-19/Ireland/D-SVUH-FBB21492-59/2025|EPI_ISL_19881763|2025-05-07   ----------------------------------------------------AGATCTGT 8
hCoV-19/Spain/CT-HUGTiP-G3R212/2025|EPI_ISL_19883055|2025-05-20   ----------------------------------------------------AGATCTGT 8
hCoV-19/Ireland/LK-UHL-704398/2025|EPI_ISL_19888621|2025-05-16   ----------------------------------------------------AGATCTGT 8
hCoV-19/Ireland/G-GUH-N3466/2025|EPI_ISL_19893175|2025-05-03   ----------------------------------------------------AGATCTGT 8
hCoV-19/Belgium/Sciensano-LS-S5276/2025|EPI_ISL_19893834|2025-04-22   ----------------------------------------------------AGATCTGT 8
hCoV-19/Italy/VEN-ULSS8-0019244_VI/2025|EPI_ISL_19899362|2025-05-26   ----------------------------------------------------AGATCTGT 8
hCoV-19/Spain/CT-HUVH-E618109/2025|EPI_ISL_19907773|2025-06-10   ----------------------------------------------------AGATCTGT 8
hCoV-19/Ireland/D-BH-03819149/2025|EPI_ISL_19911343|2025-05-29   ----------------------------------------------------AGATCTGT 8
hCoV-19/Ireland/D-BH-03993161/2025|EPI_ISL_19911355|2025-06-10   ----------------------------------------------------AGATCTGT 8
hCoV-19/Spain/CT-HUVH-E587040/2025|EPI_ISL_19871881|2025-04-26   -----------------------------------------------------GATCTGT 7
hCoV-19/Finland/THL-02392/2025|EPI_ISL_19875934|2025-05-02   -----------------------------------------------------GATCTGT 7
hCoV-19/Spain/GA-CHUAC-13181/2025|EPI_ISL_19890617|2025-05-20   -----------------------------------------------------GATCTGT 7
hCoV-19/Spain/CT-HUVH-E614843/2025|EPI_ISL_19907763|2025-06-05   -----------------------------------------------------GATCTGT 7
hCoV-19/Italy/UMB-LAOP-172-4-02/2025|EPI_ISL_19899474|2025-05-08   -------------------------------AACCAACTTTCGATCTCTTGTAGATCTGT 29
hCoV-19/Spain/NC-CHN-01008932/2025|EPI_ISL_19900595|2025-04-04   -----------------------------CCAACCAACTTTCGATCTCTTGTAGATCTGT 31
hCoV-19/Northern_Ireland/CLIMB-CM7YGDRO/2025|EPI_ISL_19877331|2025-04-14   -----------------------------------------YGATCTCTTGTAGATCTGT 19
hCoV-19/Scotland/CLIMB-CM7YEE96/2025|EPI_ISL_19897754|2025-05-13   ------------------------------------------GATCTCTTGTAGATCTGT 18
hCoV-19/France/ARA-HCL125018289001/2025|EPI_ISL_19880005|2025-05-12   ------------------------------------------------TTGTAGATCTGT 12
hCoV-19/France/ARA-RELAB-HCL725000057501/2025|EPI_ISL_19880014|2025-04-28   ------------------------------------------------TTGTAGATCTGT 12
hCoV-19/France/ARA-RELAB-HCL725000059501/2025|EPI_ISL_19885160|2025-04-25   ------------------------------------------------TTGTAGATCTGT 12
hCoV-19/France/NAQ-RELAB-HCL725000061001/2025|EPI_ISL_19885161|2025-05-10   ------------------------------------------------TTGTAGATCTGT 12
hCoV-19/France/NAQ-RELAB-HCL725000060901/2025|EPI_ISL_19885180|2025-05-09   ------------------------------------------------TTGTAGATCTGT 12
hCoV-19/France/ARA-HCL025075546601/2025|EPI_ISL_19885182|2025-05-09   ------------------------------------------------TTGTAGATCTGT 12
hCoV-19/France/ARA-HCL125018461901/2025|EPI_ISL_19885204|2025-05-14   ------------------------------------------------TTGTAGATCTGT 12
hCoV-19/France/ARA-HCL125018655901/2025|EPI_ISL_19885206|2025-05-14   ------------------------------------------------TTGTAGATCTGT 12
hCoV-19/France/ARA-HCL125019155501/2025|EPI_ISL_19885209|2025-05-18   ------------------------------------------------TTGTAGATCTGT 12
hCoV-19/France/ARA-HCL125019240901/2025|EPI_ISL_19885210|2025-05-19   ------------------------------------------------TTGTAGATCTGT 12
hCoV-19/France/ARA-HCL125019249201/2025|EPI_ISL_19885211|2025-05-19   ------------------------------------------------TTGTAGATCTGT 12
hCoV-19/France/ARA-HCL125019399701/2025|EPI_ISL_19885212|2025-05-20   ------------------------------------------------TTGTAGATCTGT 12
hCoV-19/France/ARA-HCL125019408901/2025|EPI_ISL_19885213|2025-05-20   ------------------------------------------------TTGTAGATCTGT 12
hCoV-19/France/OCC-RELAB-HCL725000064001/2025|EPI_ISL_19895004|2025-05-14   ------------------------------------------------TTGTAGATCTGT 12
hCoV-19/France/OCC-RELAB-HCL725000064101/2025|EPI_ISL_19895005|2025-05-14   ------------------------------------------------TTGTAGATCTGT 12
hCoV-19/France/NAQ-RELAB-HCL725000062601/2025|EPI_ISL_19895009|2025-05-16   ------------------------------------------------TTGTAGATCTGT 12
hCoV-19/France/ARA-RELAB-HCL725000062401/2025|EPI_ISL_19895017|2025-05-16   ------------------------------------------------TTGTAGATCTGT 12
hCoV-19/France/ARA-RELAB-HCL725000062101/2025|EPI_ISL_19895018|2025-05-13   ------------------------------------------------TTGTAGATCTGT 12
hCoV-19/France/ARA-HCL125019625101/2025|EPI_ISL_19895034|2025-05-21   ------------------------------------------------TTGTAGATCTGT 12
hCoV-19/France/ARA-HCL125019754801/2025|EPI_ISL_19895035|2025-05-22   ------------------------------------------------TTGTAGATCTGT 12
hCoV-19/France/ARA-RELAB-HCL725000064601/2025|EPI_ISL_19895041|2025-05-15   ------------------------------------------------TTGTAGATCTGT 12
hCoV-19/France/ARA-RELAB-HCL725000066001/2025|EPI_ISL_19895046|2025-05-13   ------------------------------------------------TTGTAGATCTGT 12
hCoV-19/France/OCC-RELAB-HCL725000068701/2025|EPI_ISL_19905466|2025-05-21   ------------------------------------------------TTGTAGATCTGT 12
hCoV-19/France/OCC-RELAB-HCL725000069001/2025|EPI_ISL_19905468|2025-05-21   ------------------------------------------------TTGTAGATCTGT 12
hCoV-19/France/ARA-HCL025089279801/2025|EPI_ISL_19905469|2025-06-02   ------------------------------------------------TTGTAGATCTGT 12
hCoV-19/France/PAC-RELAB-HCL725000069901/2025|EPI_ISL_19908847|2025-05-26   ------------------------------------------------TTGTAGATCTGT 12
hCoV-19/France/PAC-RELAB-HCL725000069501/2025|EPI_ISL_19908849|2025-05-30   ------------------------------------------------TTGTAGATCTGT 12
hCoV-19/France/ARA-RELAB-HCL725000071701/2025|EPI_ISL_19908858|2025-05-22   ------------------------------------------------TTGTAGATCTGT 12
hCoV-19/France/ARA-HCL025093352901/2025|EPI_ISL_19908867|2025-06-10   ------------------------------------------------TTGTAGATCTGT 12
hCoV-19/France/ARA-RELAB-HCL725000065801/2025|EPI_ISL_19895045|2025-05-16   ------------------------------------------------TTGTAGATCTGT 12
hCoV-19/Denmark/DCGC-692234/2025|EPI_ISL_19879694|2025-05-05   ----------------------------------------------TCTTGTAGATCTGT 14
hCoV-19/Finland/THL-02388/2025|EPI_ISL_19875931|2025-04-16   ---------------------------------------------CTCTTGTAGATCTGT 15
hCoV-19/Spain/PV-HUD-98024244/2025|EPI_ISL_19907759|2025-06-09   ---------------------------------------------CTCTTGTAGATCTGT 15
```

```
hCoV-19/Netherlands/GE-RIVM-145386/2025|EPI_ISL_19873764|2025-05-01         ------------------------------------------------TTGTAGATCTGT 12
hCoV-19/Netherlands/ZH-RIVM-145413/2025|EPI_ISL_19891397|2025-04-30         ------------------------------------------------TTGTAGATCTGT 12
hCoV-19/Netherlands/NH-RIVM-145458/2025|EPI_ISL_19905062|2025-05-25         ------------------------------------------------TTGTAGATCTGT 12
hCoV-19/Netherlands/NH-RIVM-145463/2025|EPI_ISL_19905063|2025-05-20         ------------------------------------------------TTGTAGATCTGT 12
hCoV-19/Netherlands/UT-RIVM-145436/2025|EPI_ISL_19905072|2025-05-15         ------------------------------------------------TTGTAGATCTGT 12
hCoV-19/Netherlands/UT-RIVM-145470/2025|EPI_ISL_19905077|2025-06-02         ------------------------------------------------TTGTAGATCTGT 12
hCoV-19/Netherlands/ZH-RIVM-145478/2025|EPI_ISL_19905094|2025-05-30         ------------------------------------------------TTGTAGATCTGT 12
hCoV-19/Netherlands/ZH-RIVM-145480/2025|EPI_ISL_19905096|2025-05-31         ------------------------------------------------TTGTAGATCTGT 12
hCoV-19/Netherlands/UT-RIVM-145481/2025|EPI_ISL_19905078|2025-05-31         ------------------------------------------------TTGTAGATCTGT 12
hCoV-19/Scotland/CLIMB-CM7YRW9H/2025|EPI_ISL_19881711|2025-05-06            ------------------------------------------------------------ 0
hCoV-19/France/IDF-RELAB-IPP04872/2025|EPI_ISL_19911293|2025-05-22          ------------------------------------------------------------ 0
hCoV-19/Canary_Islands/CN-HUC-925112913/2025|EPI_ISL_19900668|2025-04-22    ------------------------------------------------------------ 0
hCoV-19/Scotland/CLIMB-CM7YJ5BB/2025|EPI_ISL_19906458|2025-05-27            ------------------------------------------------------------ 0
hCoV-19/Scotland/CLIMB-CM7Y8PHH/2025|EPI_ISL_19897755|2025-05-14            ------------------------------------------------------------ 0
hCoV-19/Spain/AN-PMC-42706/2025|EPI_ISL_19888325|2025-05-25                 ------------------------------------------------------------ 0
hCoV-19/Scotland/CLIMB-CM7YRIA1/2025|EPI_ISL_19869961|2025-04-20            ------------------------------------------------------------ 0
hCoV-19/Scotland/CLIMB-CM7Y8UWT/2025|EPI_ISL_19869966|2025-04-21            ------------------------------------------------------------ 0
hCoV-19/England/CLIMB-CM7YMFHB/2025|EPI_ISL_19877436|2025-05-10             ------------------------------------------------------------ 0
hCoV-19/Denmark/DCGC-692213/2025|EPI_ISL_19879673|2025-04-28                ------------------------------------------------------------ 0
hCoV-19/Wales/CLIMB-CM7YRAXQ/2025|EPI_ISL_19883075|2025-05-14               ------------------------------------------------------------ 0
hCoV-19/Spain/AN-PMC-42696/2025|EPI_ISL_19888316|2025-05-14                 ------------------------------------------------------------ 0
hCoV-19/Spain/AN-PMC-42719/2025|EPI_ISL_19888338|2025-05-15                 ------------------------------------------------------------ 0
hCoV-19/England/CLIMB-CM7YE7HB/2025|EPI_ISL_19892115|2025-05-19             ------------------------------------------------------------ 0
hCoV-19/France/GES-RELAB-IPP04599/2025|EPI_ISL_19893547|2025-05-05          ------------------------------------------------------------ 0
hCoV-19/Scotland/CLIMB-CM7YEPXZ/2025|EPI_ISL_19896028|2025-05-25            ------------------------------------------------------------ 0
hCoV-19/Scotland/CLIMB-CM7YF4QT/2025|EPI_ISL_19896032|2025-05-27            ------------------------------------------------------------ 0
hCoV-19/Scotland/CLIMB-CM7YKDZ6/2025|EPI_ISL_19896033|2025-05-27            ------------------------------------------------------------ 0
hCoV-19/Scotland/CLIMB-CM7YG8H4/2025|EPI_ISL_19897748|2025-05-02            ------------------------------------------------------------ 0
hCoV-19/Wales/CLIMB-CM7YJXNZ/2025|EPI_ISL_19900036|2025-05-26               ------------------------------------------------------------ 0
hCoV-19/Germany/NW-RKI-I-1148178/2025|EPI_ISL_19902055|2025-05-21           ------------------------------------------------------------ 0
hCoV-19/Germany/NI-RKI-I-1148183/2025|EPI_ISL_19902060|2025-05-18           ------------------------------------------------------------ 0
hCoV-19/Germany/NW-RKI-I-1148191/2025|EPI_ISL_19902068|2025-05-19           ------------------------------------------------------------ 0
hCoV-19/Spain/MD-HGUGM-66414184/2025|EPI_ISL_19906298|2025-05-25            ------------------------------------------------------------ 0
hCoV-19/Spain/MD-HGUGM-66400167/2025|EPI_ISL_19906320|2025-05-22            ------------------------------------------------------------ 0
hCoV-19/France/IDF-RELAB-IPP04646/2025|EPI_ISL_19907194|2025-05-16          ------------------------------------------------------------ 0
hCoV-19/England/CLIMB-CM7YKIUQ/2025|EPI_ISL_19908573|2025-06-07             ------------------------------------------------------------ 0
hCoV-19/France/GES-RELAB-IPP04823/2025|EPI_ISL_19911257|2025-05-26          ------------------------------------------------------------ 0
hCoV-19/France/IDF-RELAB-IPP04863/2025|EPI_ISL_19911265|2025-05-24          ------------------------------------------------------------ 0
hCoV-19/Denmark/DCGC-692245/2025|EPI_ISL_19879704|2025-04-28                ------------------------------------------------------------ 0


hCoV-19/Ireland/D-SVUH-FBA98650-31/2025|EPI_ISL_19866548|2025-05-04         ------------------------------------------------------------ 0
hCoV-19/Spain/PV-HUB-5628663/2025|EPI_ISL_19908839|2025-05-16               ------------------------------------------------------------ 0
hCoV-19/Spain/PV-HUB-41587498/2025|EPI_ISL_19908930|2025-05-20              ------------------------------------------------------------ 0
hCoV-19/Spain/PV-HUB-56291851/2025|EPI_ISL_19908970|2025-05-31              ------------------------------------------------------------ 0
hCoV-19/Northern_Ireland/CLIMB-CM7YR8I6/2025|EPI_ISL_19877334|2025-04-23    ---------------------------------------GCTGCATGCTTAGTGCACTCA 21
hCoV-19/Germany/NW-RKI-I-1148136/2025|EPI_ISL_19871950|2025-04-23           ----------------AAAATCTGTGTGGCTGTCACTCGGCTGCATGCTTAGTGCACTCA 44
hCoV-19/Germany/NW-RKI-I-1148141/2025|EPI_ISL_19871955|2025-04-23           ----------------AAAATCTGTGTGGCTGTCACTCGGCTGCATGCTTAGTGCACTCA 44
hCoV-19/Germany/BW-RKI-I-1148164/2025|EPI_ISL_19884987|2025-04-17           ----------------AAAATCTGTGTGGCTGTCACTCGGCTGCATGCTTAGTGCACTCA 44
hCoV-19/Scotland/CLIMB-CM7YFGW7/2025|EPI_ISL_19897691|2025-05-24            ----------------AAAATCTGTGTGGCTGTCACTCGGCTGCATGCTTAGTGCACTCA 44
hCoV-19/Ireland/G-LUH-N3473/2025|EPI_ISL_19893181|2025-05-14                TCTCTAAACGAACTTTAAAATCTGTGTGGCTGTCACTCGGCTGCATGCTTAGTGCACTCA 111
hCoV-19/Spain/AN-PMC-42728/2025|EPI_ISL_19888347|2025-05-25                 TCTCTAAACGAACTTNAAAATCTGTGTGGCTGTCACTCGGCTGCATGCTTAGTGCACTCA 112
hCoV-19/Sweden/000200866492N2/2025|EPI_ISL_19870050|2025-04-22              TCTCTAAACGAACTTTAAAATCTGTGTGGCTGTCACTCGGCTGCATGCTTAGTGCACTCA 118
```

hCoV-19/Sweden/000200867159N2/2025|EPI_ISL_19870052|2025-04-23	TCTCTAAACGAACTTTAAAATCTGTGTGGCTGTCACTCGGCTGCATGCTTAGTGCACTCA	118
hCoV-19/Ireland/G-LUH-N3445/2025|EPI_ISL_19893154|2025-05-07	TCTCTAAACGAACTTTAAAATCTGTGTGGCTGTCACTCGGCTGCATGCTTAGTGCACTCA	118
hCoV-19/Ireland/G-LUH-N3443/2025|EPI_ISL_19893152|2025-05-07	TCTCTAAACGAACTTTAAAATCTGTGTGGCTGTCACTCGGCTGCATGCTTAGTGCACTCA	116
hCoV-19/Scotland/CLIMB-CM7YF9PJ/2025|EPI_ISL_19895983|2025-05-19	TCTCTAAACGAACTTNAAAATCTGTGTGGCTGTCACTCGGCTGCATGCTTAGTGCACTCA	115
hCoV-19/Scotland/CLIMB-CM7YKMK8/2025|EPI_ISL_19869976|2025-04-25	TCTCTAAACGAACTTNAAAATCTGTGTGGCTGTCACTCGGCTGCATGCTTAGTGCACTCA	114
hCoV-19/Ireland/G-LUH-N3444/2025|EPI_ISL_19893153|2025-05-07	TCTCTAAACGAACTTTAAAATCTGTGTGGCTGTCACTCGGCTGCATGCTTAGTGCACTCA	114
hCoV-19/Switzerland/VD-CHUV-8604552682/2025|EPI_ISL_19906527|2025-05-19	TCTCTAAACGAACTTTAAAATCTGTGTGGCTGTCACTCGGCTGCATGCTTAGTGCACTCA	119
hCoV-19/Scotland/CLIMB-CM7YR3YO/2025|EPI_ISL_19896015|2025-05-24	TCTCTAAACGAACTTNAAAATCTGTGTGGCTGTCACTCGGCTGCATGCTTAGTGCACTCA	120
hCoV-19/Sweden/O-18_SE100_25CS500180/2025|EPI_ISL_19866288|2025-04-15	TCTCTAAACGAACTTTAAAATCTGTGTGGCTGTCACTCGGCTGCATGCTTAGTGCACTCA	120
hCoV-19/Ireland/D-CS250088/2025|EPI_ISL_19877457|2025-04-16	TCTCTAAACGAACTTTAAAATCTGTGTGGCTGTCACTCGGCTGCATGCTTAGTGCACTCA	120
hCoV-19/Ireland/G-LUH-N3442/2025|EPI_ISL_19893151|2025-05-07	TCTCTAAACGAACTTTAAAATCTGTGTGGCTGTCACTCGGCTGCATGCTTAGTGCACTCA	120
hCoV-19/Scotland/CLIMB-CM7YMZ5M/2025|EPI_ISL_19906439|2025-05-22	TCTCTAAACGAACNNNAAAATCTGTGTGGCTGTCACTCGGCTGCATGCTTAGTGCACTCA	119
hCoV-19/Scotland/CLIMB-CM7YR3ZG/2025|EPI_ISL_19897722|2025-05-13	TCTCTAAACGAACNNNAAAATCTGTGTGGCTGTCACTCGGCTGCATGCTTAGTGCACTCA	118
hCoV-19/Scotland/CLIMB-CM7YEYGT/2025|EPI_ISL_19897741|2025-04-28	TCTCTAAACGAACNNNAAAATCTGTGTGGCTGTCACTCGGCTGCATGCTTAGTGCACTCA	118
hCoV-19/Scotland/CLIMB-CM7YGAJ3/2025|EPI_ISL_19895963|2025-05-13	TCTCTAAACGAACNNNAAAATCTGTGTGGCTGTCACTCGGCTGCATGCTTAGTGCACTCA	107
hCoV-19/Scotland/CLIMB-CM7YJ1ZO/2025|EPI_ISL_19897757|2025-05-14	TCTCTAAACGAACNNNAAAATCTGTGTGGCTGTCACTCGGCTGCATGCTTAGTGCACTCA	108
hCoV-19/Scotland/CLIMB-CM7Y8C4Y/2025|EPI_ISL_19906404|2025-05-15	TCTCTAAACGAACNNNAAAATCTGTGTGGCTGTCACTCGGCTGCATGCTTAGTGCACTCA	115
hCoV-19/Northern_Ireland/CLIMB-CM7YGGYM/2025|EPI_ISL_19897641|2025-05-23	TCTCTAAACGAACNNNAAAATCTGTGTGGCTGTCACTCGGCTGCATGCTTAGTGCACTCA	110
hCoV-19/Scotland/CLIMB-CM7YFW7M/2025|EPI_ISL_19881718|2025-05-10	TCTCTAAACGAACNNNAAAATCTGTGTGGCTGTCACTCGGCTGCATGCTTAGTGCACTCA	113
hCoV-19/Scotland/CLIMB-CM7YEMXP/2025|EPI_ISL_19888387|2025-05-10	TCTCTAAACGAACNNNAAAATCTGTGTGGCTGTCACTCGGCTGCATGCTTAGTGCACTCA	112
hCoV-19/Spain/CT-HUVH-E593511/2025|EPI_ISL_19881731|2025-05-06	TCTCTAAACGAACTTTAAAATCTGTGTGGCTGTCACTCGGCTGCATGCTTAGTGCACTCA	65
hCoV-19/Spain/CT-HUJT-E325362/2025|EPI_ISL_19889935|2025-05-20	TCTCTAAACGAACTTTAAAATCTGTGTGGCTGTCACTCGGCTGCATGCTTAGTGCACTCA	66
hCoV-19/Spain/PV-HUD-84743204/2025|EPI_ISL_19907746|2025-06-06	TCTCTAAACGAACTTTAAAATCTGTGTGGCTGTCACTCGGCTGCATGCTTAGTGCACTCA	67
hCoV-19/Ireland/D-SVUH-FBB21492-54/2025|EPI_ISL_19881758|2025-05-11	TCTCTAAACGAACTTTAAAATCTGTGTGGCTGTCACTCGGCTGCATGCTTAGTGCACTCA	68
hCoV-19/Spain/CT-HUVH-E596725/2025|EPI_ISL_19881729|2025-05-10	TCTCTAAACGAACTTTAAAATCTGTGTGGCTGTCACTCGGCTGCATGCTTAGTGCACTCA	62
hCoV-19/Spain/CT-HUVH-E593882/2025|EPI_ISL_19881732|2025-05-06	TCTCTAAACGAACTTTAAAATCTGTGTGGCTGTCACTCGGCTGCATGCTTAGTGCACTCA	62
hCoV-19/Finland/THL-02385/2025|EPI_ISL_19875928|2025-04-16	TCTCTAAACGAACTTTAAAATCTGTGTGGCTGTCACTCGGCTGCATGCTTAGTGCACTCA	69
hCoV-19/Ireland/D-BH-02351167/2025|EPI_ISL_19911337|2025-05-28	TCTCTAAACGAACTTTAAAATCTGTGTGGCTGTCACTTGGCTGCATGCTTAGTGCACTCA	68
hCoV-19/Ireland/D-NVRL-XIRL00001782/2025|EPI_ISL_19896104|2025-05-14	TCTTTAAACGAACTTTAAAATCTGTGTGGCTGTCACTCGGCTGCATGCTTAGTGCACTCA	68
hCoV-19/Ireland/TA-NVRL-GIRL00039444/2025|EPI_ISL_19896118|2025-05-16	TCTTTAAACGAACTTTAAAATCTGTGTGGCTGTCACTCGGCTGCATGCTTAGTGCACTCA	68
hCoV-19/Spain/GA-CHUAC-13223/2025|EPI_ISL_19894946|2025-05-31	TCTCTAAACGAACTTTAAAATCTGTGTGGCTGTCACTCGGCTGCATGCTTAGTGCACTCA	67
hCoV-19/Spain/GA-CHUAC-13215/2025|EPI_ISL_19894939|2025-05-29	TCTCTAAACGAACTTTAAAATCTGTGTGGCTGTCACTCGGCTGTATGCTTAGTGCACTCA	67
hCoV-19/Finland/THL-02398/2025|EPI_ISL_19875939|2025-05-04	TCTCTAAACGAACTTTAAAATCTGTGTGGCTGTCACTCGGCTGCATGCTTAGTGCACTCA	68
hCoV-19/Finland/THL-02400/2025|EPI_ISL_19875941|2025-05-03	TCTCTAAACGAACTTTAAAATCTGTGTGGCTGTCACTCGGCTGCATGCTTAGTGCACTCA	68
hCoV-19/Ireland/LK-UHL-702929/2025|EPI_ISL_19877895|2025-04-16	TCTCTAAACGAACTTTAAAATCTGTGTGGCTGTCACTCGGCTGCATGCTTAGTGCACTCA	68
hCoV-19/Ireland/LK-UHL-703788/2025|EPI_ISL_19877905|2025-05-03	TCTCTAAACGAACTTTAAAATCTGTGTGGCTGTCACTCGGCTGCATGCTTAGTGCACTCA	68
hCoV-19/Ireland/LK-UHL-703851/2025|EPI_ISL_19877907|2025-05-04	TCTCTAAACGAACTTTAAAATCTGTGTGGCTGTCACTCGGCTGCATGCTTAGTGCACTCA	68
hCoV-19/Ireland/LK-UHL-703889/2025|EPI_ISL_19877908|2025-05-05	TCTCTAAACGAACTTTAAAATCTGTGTGGCTGTCACTCGGCTGCATGCTTAGTGCACTCA	68
hCoV-19/Ireland/D-SVUH-FBB21492-49/2025|EPI_ISL_19881756|2025-05-14	TCTCTAAACGAACTTTAAAATCTGTGTGGCTGTCACTCGGCTGCATGCTTAGTGCACTCA	68
hCoV-19/Ireland/D-SVUH-FBB21492-59/2025|EPI_ISL_19881763|2025-05-07	TCTCTAAACGAACTTTAAAATCTGTGTGGCTGTCACTCGGCTGCATGCTTAGTGCACTCA	68
hCoV-19/Spain/CT-HUGTiP-G3R212/2025|EPI_ISL_19883055|2025-05-20	TCTCTAAACGAACTTTAAAATCTGTGTGGCTGTCACTCGGCTGCATGCTTAGTGCACTCA	68
hCoV-19/Ireland/LK-UHL-704398/2025|EPI_ISL_19888621|2025-05-16	TCTCTAAACGAACTTTAAAATCTGTGTGGCTGTCACTCGGCTGCATGCTTAGTGCACTCA	68
hCoV-19/Ireland/G-GUH-N3466/2025|EPI_ISL_19893175|2025-05-03	TCTCTAAACGAACTTTAAAATCTGTGTGGCTGTCACTCGGCTGCATGCTTAGTGCACTCA	68
hCoV-19/Belgium/Sciensano-LS-S5276/2025|EPI_ISL_19893834|2025-04-22	TCTCTAAACGAACTTTAAAATCTGTGTGGCTGTCACTCGGCTGCATGCTTAGTGCACTCA	68
hCoV-19/Italy/VEN-ULSS8-0019244_VI/2025|EPI_ISL_19899362|2025-05-26	TCTCTAAACGAACTTTAAAATCTGTGTGGCTGTCACTCGGCTGCATGCTTAGTGCACTCA	68
hCoV-19/Spain/CT-HUVH-E618109/2025|EPI_ISL_19907773|2025-06-10	TCTCTAAACGAACTTTAAAATCTGTGTGGCTGTCACTCGGCTGCATGCTTAGTGCACTCA	68
hCoV-19/Ireland/D-BH-03819149/2025|EPI_ISL_19911343|2025-05-29	TCTCTAAACGAACTTTAAAATCTGTGTGGCTGTCACTCGGCTGCATGCTTAGTGCACTCA	68
hCoV-19/Ireland/D-BH-03993161/2025|EPI_ISL_19911355|2025-06-10	TCTCTAAACGAACTTTAAAATCTGTGTGGCTGTCACTCGGCTGCATGCTTAGTGCACTCA	68
hCoV-19/Spain/CT-HUVH-E587040/2025|EPI_ISL_19871881|2025-04-26	TCTCTAAACGAACTTTAAAATCTGTGTGGCTGTCACTCGGCTGCATGCTTAGTGCACTCA	67
hCoV-19/Finland/THL-02392/2025|EPI_ISL_19875934|2025-05-02	TCTCTAAACGAACTTTAAAATCTGTGTGGCTGTCACTCGGCTGCATGCTTAGTGCACTCA	67
hCoV-19/Spain/GA-CHUAC-13181/2025|EPI_ISL_19890617|2025-05-20	TCTCTAAACGAACTTTAAAATCTGTGTGGCTGTCACTCGGCTGCATGCTTAGTGCACTCA	67
hCoV-19/Spain/CT-HUVH-E614843/2025|EPI_ISL_19907763|2025-06-05	TCTCTAAACGAACTTTAAAATCTGTGTGGCTGTCACTCGGCTGCATGCTTAGTGCACTCA	67
hCoV-19/Italy/UMB-LAOP-172-4-02/2025|EPI_ISL_19899474|2025-05-08	TCTCTAAACGAACTTTAAAATCTGTGTGGCTGTCACTCGGCTGCATGCTTAGTGCACTCA	89

```
hCoV-19/Spain/NC-CHN-01008932/2025|EPI_ISL_19900595|2025-04-04              TCTCTAAACGAACTTTAAAATCTGTGTGGCTGTCACTCGGCTGCATGCTTAGTGCACTCA 91
hCoV-19/Northern_Ireland/CLIMB-CM7YGDRO/2025|EPI_ISL_19877331|2025-04-14    TCTCTAAACGAACTNNAAAATCTGTGTGGCTGTCACTCGGCTGCATGCTTAGTGCACTCA 79
hCoV-19/Scotland/CLIMB-CM7YEE96/2025|EPI_ISL_19897754|2025-05-13            TCTCTAAACGAACTTTAAAATCTGTGTGGCTGTCACTCGGCTGCATGCTTAGTGCACTCA 78
hCoV-19/France/ARA-HCL125018289001/2025|EPI_ISL_19880005|2025-05-12         TCTCTAAACGAACNTNAAAATCTGTGTGGCTGTCACTCGGCTGCATGCTTAGTGCACTCA 72
hCoV-19/France/ARA-RELAB-HCL725000057501/2025|EPI_ISL_19880014|2025-04-28   TCTCTAAACGAACNTNAAAATCTGTGTGGCTGTCACTCGGCTGCATGCTTAGTGCACTCA 72
hCoV-19/France/ARA-RELAB-HCL725000059501/2025|EPI_ISL_19885160|2025-04-25   TCTCTAAACGAACNTNAAAATCTGTGTGGCTGTCACTCGGCTGCATGCTTAGTGCACTCA 72
hCoV-19/France/NAQ-RELAB-HCL725000061001/2025|EPI_ISL_19885161|2025-05-10   TCTCTAAACGAACNTNAAAATCTGTGTGGCTGTCACTCGGCTGCATGCTTAGTGCACTCA 72
hCoV-19/France/NAQ-RELAB-HCL725000060901/2025|EPI_ISL_19885180|2025-05-09   TCTCTAAACGAACNTNAAAATCTGTGTGGCTGTCACTCGGCTGCATGCTTAGTGCACTCA 72
hCoV-19/France/ARA-HCL025075546601/2025|EPI_ISL_19885182|2025-05-09         TCTCTAAACGAACNTNAAAATCTGTGTGGCTGTCACTCGGCTGCATGCTTAGTGCACTCA 72
hCoV-19/France/ARA-HCL125018461901/2025|EPI_ISL_19885204|2025-05-14         TCTCTAAACGAACNTNAAAATCTGTGTGGCTGTCACTCGGCTGCATGCTTAGTGCACTCA 72
hCoV-19/France/ARA-HCL125018655901/2025|EPI_ISL_19885206|2025-05-14         TCTCTAAACGAACNTNAAAATCTGTGTGGCTGTCACTCGGCTGCATGCTTAGTGCACTCA 72
hCoV-19/France/ARA-HCL125019155501/2025|EPI_ISL_19885209|2025-05-18         TCTCTAAACGAACNTNAAAATCTGTGTGGCTGTCACTCGGCTGCATGCTTAGTGCACTCA 72
hCoV-19/France/ARA-HCL125019240901/2025|EPI_ISL_19885210|2025-05-19         TCTCTAAACGAACNTNAAAATCTGTGTGGCTGTCACTCGGCTGCATGCTTAGTGCACTCA 72
hCoV-19/France/ARA-HCL125019249201/2025|EPI_ISL_19885211|2025-05-19         TCTCTAAACGAACNTNAAAATCTGTGTGGCTGTCACTCGGCTGCATGCTTAGTGCACTCA 72
hCoV-19/France/ARA-HCL125019399701/2025|EPI_ISL_19885212|2025-05-20         TCTCTAAACGAACNTNAAAATCTGTGTGGCTGTCACTCGGCTGCATGCTTAGTGCACTCA 72
hCoV-19/France/ARA-HCL125019408901/2025|EPI_ISL_19885213|2025-05-20         TCTCTAAACGAACNTNAAAATCTGTGTGGCTGTCACTCGGCTGCATGCTTAGTGCACTCA 72
hCoV-19/France/OCC-RELAB-HCL725000064001/2025|EPI_ISL_19895004|2025-05-14   TCTCTAAACGAACNTNAAAATCTGTGTGGCTGTCACTCGGCTGCATGCTTAGTGCACTCA 72
hCoV-19/France/OCC-RELAB-HCL725000064101/2025|EPI_ISL_19895005|2025-05-14   TCTCTAAACGAACNTNAAAATCTGTGTGGCTGTCACTCGGCTGCATGCTTAGTGCACTCA 72
hCoV-19/France/NAQ-RELAB-HCL725000062601/2025|EPI_ISL_19895009|2025-05-16   TCTCTAAACGAACNTNAAAATCTGTGTGGCTGTCACTCGGCTGCATGCTTAGTGCACTCA 72
hCoV-19/France/ARA-RELAB-HCL725000062401/2025|EPI_ISL_19895017|2025-05-16   TCTCTAAACGAACNTNAAAATCTGTGTGGCTGTCACTCGGCTGCATGCTTAGTGCACTCA 72
hCoV-19/France/ARA-RELAB-HCL725000062101/2025|EPI_ISL_19895018|2025-05-13   TCTCTAAACGAACNTNAAAATCTGTGTGGCTGTCACTCGGCTGCATGCTTAGTGCACTCA 72
hCoV-19/France/ARA-HCL125019625101/2025|EPI_ISL_19895034|2025-05-21         TCTCTAAACGAACNTNAAAATCTGTGTGGCTGTCACTCGGCTGCATGCTTAGTGCACTCA 72
hCoV-19/France/ARA-HCL125019754801/2025|EPI_ISL_19895035|2025-05-22         TCTCTAAACGAACNTNAAAATCTGTGTGGCTGTCACTCGGCTGCATGCTTAGTGCACTCA 72
hCoV-19/France/ARA-RELAB-HCL725000064601/2025|EPI_ISL_19895041|2025-05-15   TCTCTAAACGAACNTNAAAATCTGTGTGGCTGTCACTCGGCTGCATGCTTAGTGCACTCA 72
hCoV-19/France/ARA-RELAB-HCL725000066001/2025|EPI_ISL_19895046|2025-05-13   TCTCTAAACGAACNTNAAAATCTGTGTGGCTGTCACTCGGCTGCATGCTTAGTGCACTCA 72
hCoV-19/France/OCC-RELAB-HCL725000068701/2025|EPI_ISL_19905466|2025-05-21   TCTCTAAACGAACNTNAAAATCTGTGTGGCTGTCACTCGGCTGCATGCTTAGTGCACTCA 72
hCoV-19/France/OCC-RELAB-HCL725000069001/2025|EPI_ISL_19905468|2025-05-21   TCTCTAAACGAACNTNAAAATCTGTGTGGCTGTCACTCGGCTGCATGCTTAGTGCACTCA 72
hCoV-19/France/ARA-HCL025089279801/2025|EPI_ISL_19905469|2025-06-02         TCTCTAAACGAACNTNAAAATCTGTGTGGCTGTCACTCGGCTGCATGCTTAGTGCACTCA 72
hCoV-19/France/PAC-RELAB-HCL725000069901/2025|EPI_ISL_19908847|2025-05-26   TCTCTAAACGAACNTNAAAATCTGTGTGGCTGTCACTCGGCTGCATGCTTAGTGCACTCA 72
hCoV-19/France/PAC-RELAB-HCL725000069501/2025|EPI_ISL_19908849|2025-05-30   TCTCTAAACGAACNTNAAAATCTGTGTGGCTGTCACTCGGCTGCATGCTTAGTGCACTCA 72
hCoV-19/France/ARA-RELAB-HCL725000071701/2025|EPI_ISL_19908858|2025-05-22   TCTCTAAACGAACNTNAAAATCTGTGTGGCTGTCACTCGGCTGCATGCTTAGTGCACTCA 72
hCoV-19/France/ARA-HCL025093352901/2025|EPI_ISL_19908867|2025-06-10         TCTCTAAACGAACNTNAAAATCTGTGTGGCTGTCACTCGGCTGCATGCTTAGTGCACTCA 72
hCoV-19/France/ARA-RELAB-HCL725000065801/2025|EPI_ISL_19895045|2025-05-16   TCTCTAAACGAACNTNAAAATCTGTGTGGCTGTCACTCGGCTGCATGCTTAGTGCACTCA 72
hCoV-19/Denmark/DCGC-692234/2025|EPI_ISL_19879694|2025-05-05                TCTCTAAACGAACTTTAAAATCTGTGTGGCTGTCACTCGGCTGCATGCTTAGTGCACTCA 74
hCoV-19/Finland/THL-02388/2025|EPI_ISL_19875931|2025-04-16                  TCTCTAAACGAACTTTAAAATCTGTGTGGCTGTCACTCGGCTGCATGCTTAGTGCACTCA 75
hCoV-19/Spain/PV-HUD-98024244/2025|EPI_ISL_19907759|2025-06-09              TCTCTAAACGAACTTTAAAATCTGTGTGGCTGTCACTCGGCTGCATGCTTAGTGCACTCA 75
hCoV-19/Netherlands/GE-RIVM-145386/2025|EPI_ISL_19873764|2025-05-01         TCTCTAAACGAACTTTAAAATCTGTGTGGCTGTCACTCGGCTGCATGCTTAGTGCACTCA 72
hCoV-19/Netherlands/ZH-RIVM-145413/2025|EPI_ISL_19891397|2025-04-30         TCTCTAAACGAACTTTAAAATCTGTGTGGCTGTCACTCGGCTGCATGCTTAGTGCACTCA 72
hCoV-19/Netherlands/NH-RIVM-145458/2025|EPI_ISL_19905062|2025-05-25         TCTCTAAACGAACTTTAAAATCTGTGTGGCTGTCACTCGGCTGCATGCTTAGTGCACTCA 72
hCoV-19/Netherlands/NH-RIVM-145463/2025|EPI_ISL_19905063|2025-05-20         TCTCTAAACGAACTTTAAAATCTGTGTGGCTGTCACTCGGCTGCATGCTTAGTGCACTCA 72
hCoV-19/Netherlands/UT-RIVM-145436/2025|EPI_ISL_19905072|2025-05-15         TCTCTAAACGAACTTTAAAATCTGTGTGGCTGTCACTCGGCTGCATGCTTAGTGCACTCA 72
hCoV-19/Netherlands/UT-RIVM-145470/2025|EPI_ISL_19905077|2025-06-02         TCTCTAAACGAACTTTAAAATCTGTGTGGCTGTCACTCGGCTGCATGCTTAGTGCACTCA 72
hCoV-19/Netherlands/ZH-RIVM-145478/2025|EPI_ISL_19905094|2025-05-30         TCTCTAAACGAACTTTAAAATCTGTGTGGCTGTCACTCGGCTGCATGCTTAGTGCACTCA 72
hCoV-19/Netherlands/ZH-RIVM-145480/2025|EPI_ISL_19905096|2025-05-31         TCTCTAAACGAACTTTAAAATCTGTGTGGCTGTCACTCGGCTGCATGCTTAGTGCACTCA 72
hCoV-19/Netherlands/UT-RIVM-145481/2025|EPI_ISL_19905078|2025-05-31         TCTCTAAACGAACTTTAAAATCTGTGTGGCTGTCACTTGGCTGCATGCTTAGTGCACTCA 72
hCoV-19/Scotland/CLIMB-CM7YRW9H/2025|EPI_ISL_19881711|2025-05-06            ----------------AAAATCTGTGTGGCTGTCACTCGGCTGCATGCTTAGTGCACTCA 44
hCoV-19/France/IDF-RELAB-IPP04872/2025|EPI_ISL_19911293|2025-05-22          --------------------TCTGTGTGGCTGTCACTCGGCTGCATGCTTAGTGCACTCA 40
hCoV-19/Canary_Islands/CN-HUC-925112913/2025|EPI_ISL_19900668|2025-04-22    ------------CTTTAAAATCTGTGTGGCTGTCACTCGGCTGCATGCTTAGTGCACTCA 48
hCoV-19/Scotland/CLIMB-CM7YJ5BB/2025|EPI_ISL_19906458|2025-05-27            ----------------AAAATCTGTGTGACTGTCACTCGGCTGCATGCTTAGTGCACTCA 44
hCoV-19/Scotland/CLIMB-CM7Y8PHH/2025|EPI_ISL_19897755|2025-05-14            ----------------AAAATCTGTGTGGCTGTCACTCGGCTGCATGCTTAGTGCACTCA 44
hCoV-19/Spain/AN-PMC-42706/2025|EPI_ISL_19888325|2025-05-25                 ----------------AAAATCTGTGTGGCTGTCACTCGGCTGCATGCTTAGTGCACTCA 44
hCoV-19/Scotland/CLIMB-CM7YRIA1/2025|EPI_ISL_19869961|2025-04-20            ----------------AAAATCTGTGTGGCTGTCACTCGGCTGCATGCTTAGTGCACTCA 44
hCoV-19/Scotland/CLIMB-CM7Y8UWT/2025|EPI_ISL_19869966|2025-04-21            ----------------AAAATCTGTGTGGCTGTCACTCGGCTGCATGCTTAGTGCACTCA 44
```

```
hCoV-19/England/CLIMB-CM7YMFHB/2025|EPI_ISL_19877436|2025-05-10          ----------------AAAATCTGTGTGGCTGTCACTCGGCTGCATGCTTAGTGCACTCA 44
hCoV-19/Denmark/DCGC-692213/2025|EPI_ISL_19879673|2025-04-28             ----------------AAAATCTGTGTGGCTGTCACTCGGCTGCATGCTTAGTGCACTCA 44
hCoV-19/Wales/CLIMB-CM7YRAXQ/2025|EPI_ISL_19883075|2025-05-14            ----------------AAAATCTGTGTGGCTGTCACTCGGCTGCATGCTTAGTGCACTCA 44
hCoV-19/Spain/AN-PMC-42696/2025|EPI_ISL_19888316|2025-05-14              ----------------AAAATCTGTGTGGCTGTCACTCGGCTGCATGCTTAGTGCACTCA 44
hCoV-19/Spain/AN-PMC-42719/2025|EPI_ISL_19888338|2025-05-15              ----------------AAAATCTGTGTGGCTGTCACTCGGCTGCATGCTTAGTGCACTCA 44
hCoV-19/England/CLIMB-CM7YE7HB/2025|EPI_ISL_19892115|2025-05-19          ----------------AAAATCTGTGTGGCTGTCACTCGGCTGCATGCTTAGTGCACTCA 44
hCoV-19/France/GES-RELAB-IPP04599/2025|EPI_ISL_19893547|2025-05-05       ----------------AAAATCTGTGTGGCTGTCACTCGGCTGCATGCTTAGTGCACTCA 44
hCoV-19/Scotland/CLIMB-CM7YEPXZ/2025|EPI_ISL_19896028|2025-05-25         ----------------AAAATCTGTGTGGCTGTCACTCGGCTGCATGCTTAGTGCACTCA 44
hCoV-19/Scotland/CLIMB-CM7YF4QT/2025|EPI_ISL_19896032|2025-05-27         ----------------AAAATCTGTGTGGCTGTCACTCGGCTGCATGCTTAGTGCACTCA 44
hCoV-19/Scotland/CLIMB-CM7YKDZ6/2025|EPI_ISL_19896033|2025-05-27         ----------------AAAATCTGTGTGGCTGTCACTCGGCTGCATGCTTAGTGCACTCA 44
hCoV-19/Scotland/CLIMB-CM7YG8H4/2025|EPI_ISL_19897748|2025-05-02         ----------------AAAATCTGTGTGGCTGTCACTCGGCTGCATGCTTAGTGCACTCA 44
hCoV-19/Wales/CLIMB-CM7YJXNZ/2025|EPI_ISL_19900036|2025-05-26            ----------------AAAATCTGTGTGGCTGTCACTCGGCTGCATGCTTAGTGCACTCA 44
hCoV-19/Germany/NW-RKI-I-1148178/2025|EPI_ISL_19902055|2025-05-21        ----------------AAAATCTGTGTGGCTGTCACTCGGCTGCATGCTTAGTGCACTCA 44
hCoV-19/Germany/NI-RKI-I-1148183/2025|EPI_ISL_19902060|2025-05-18        ----------------AAAATCTGTGTGGCTGTCACTCGGCTGCATGCTTAGTGCACTCA 44
hCoV-19/Germany/NW-RKI-I-1148191/2025|EPI_ISL_19902068|2025-05-19        ----------------AAAATCTGTGTGGCTGTCACTCGGCTGCATGCTTAGTGCACTCA 44
hCoV-19/Spain/MD-HGUGM-66414184/2025|EPI_ISL_19906298|2025-05-25         ----------------AAAATCTGTGTGGCTGTCACTCGGCTGCATGCTTAGTGCACTCA 44
hCoV-19/Spain/MD-HGUGM-66400167/2025|EPI_ISL_19906320|2025-05-22         ----------------AAAATCTGTGTGGCTGTCACTCGGCTGCATGCTTAGTGCACTCA 44
hCoV-19/France/IDF-RELAB-IPP04646/2025|EPI_ISL_19907194|2025-05-16       ----------------AAAATCTGTGTGGCTGTCACTCGGCTGCATGCTTAGTGCACTCA 44
hCoV-19/England/CLIMB-CM7YKIUQ/2025|EPI_ISL_19908573|2025-06-07          ----------------AAAATCTGTGTGGCTGTCACTCGGCTGCATGCTTAGTGCACTCA 44
hCoV-19/France/GES-RELAB-IPP04823/2025|EPI_ISL_19911257|2025-05-26       ----------------AAAATCTGTGTGGCTGTCACTCGGCTGCATGCTTAGTGCACTCA 44
hCoV-19/France/IDF-RELAB-IPP04863/2025|EPI_ISL_19911265|2025-05-24       ----------------AAAATCTGTGTGGCTGTCACTCGGCTGCATGCTTAGTGCACTCA 44
hCoV-19/Denmark/DCGC-692245/2025|EPI_ISL_19879704|2025-04-28             ----------------AAAATCTGTGTGGCTGTCACTCGGCTGCATGCTTAGTGCACTCA 44


hCoV-19/Ireland/D-SVUH-FBA98650-31/2025|EPI_ISL_19866548|2025-05-04      ------------------------------------------------------------ 0
hCoV-19/Spain/PV-HUB-5628663/2025|EPI_ISL_19908839|2025-05-16            ------------------------------------------------------------ 0
hCoV-19/Spain/PV-HUB-41587498/2025|EPI_ISL_19908930|2025-05-20           ------------------------------------------------------------ 0
hCoV-19/Spain/PV-HUB-56291851/2025|EPI_ISL_19908970|2025-05-31           ------------------------------------------------------------ 0
hCoV-19/Northern_Ireland/CLIMB-CM7YR8I6/2025|EPI_ISL_19877334|2025-04-23 CGCAGTATAATTAATAACTAATTACTGTCGTTGACAGGACACGAGTAACTCGTCTATCTT 81
hCoV-19/Germany/NW-RKI-I-1148136/2025|EPI_ISL_19871950|2025-04-23        CGCAGTATAATTAATAACTAATTACTGTCGTTGACAGGACACGAGTAACTCGTCTATCTT 104
hCoV-19/Germany/NW-RKI-I-1148141/2025|EPI_ISL_19871955|2025-04-23        CGCAGTATAATTAATAACTAATTACTGTCGTTGACAGGACACGAGTAACTCGTCTATCTT 104
hCoV-19/Germany/BW-RKI-I-1148164/2025|EPI_ISL_19884987|2025-04-17        CGCAGTATAATTAATAACTAATTACTGTCGTTGACAGGACACGAGTAACTCGTCTATCTT 104
hCoV-19/Scotland/CLIMB-CM7YFGW7/2025|EPI_ISL_19897691|2025-05-24         CGCAGTATAATTAATAACTAATTACTGTCGTTGACAGGACACGAGTAACTCGTCTATCTT 104
hCoV-19/Ireland/G-LUH-N3473/2025|EPI_ISL_19893181|2025-05-14             CGCAGTATAATTAATAACTAATTACTGTCGTTGACAGGACACGAGTAACTCGTCTATCTT 171
hCoV-19/Spain/AN-PMC-42728/2025|EPI_ISL_19888347|2025-05-25              CGCAGTATAATTAATAACTAATTACTGTCGTTGACAGGACACGAGTAACTCGTCTATCTT 172
hCoV-19/Sweden/00200866492N2/2025|EPI_ISL_19870050|2025-04-22            CGCAGTATAATTAATAACTAATTACTGTCGTTGACAGGACACGAGTAACTCGTCTATCTT 178
hCoV-19/Sweden/00200867159N2/2025|EPI_ISL_19870052|2025-04-23            CGCAGTATAATTAATAACTAATTACTGTCGTTGACAGGACACGAGTAACTCGTCTATCTT 178
hCoV-19/Ireland/G-LUH-N3445/2025|EPI_ISL_19893154|2025-05-07             CGCAGTATAATTAATAACTAATTACTGTCGTTGACAGGACACGAGTAACTCGTCTATCTT 178
hCoV-19/Ireland/G-LUH-N3443/2025|EPI_ISL_19893152|2025-05-07             CGCAGTATAATTAATAACTAATTACTGTCGTTGACAGGACACGAGTAACTCGTCTATCTT 176
hCoV-19/Scotland/CLIMB-CM7YF9PJ/2025|EPI_ISL_19895983|2025-05-19         CGCAGTATAATTAATAACTAATTACTGTCGTTGACAGGACACGAGTAACTCGTCTATCTT 175
hCoV-19/Scotland/CLIMB-CM7YKMK8/2025|EPI_ISL_19869976|2025-04-25         CGCAGTATAATTAATAACTAATTACTGTCGTTGACAGGACACGAGTAACTCGTCTATCTT 174
hCoV-19/Ireland/G-LUH-N3444/2025|EPI_ISL_19893153|2025-05-07             CGCAGTATAATTAATAACTAATTACTGTCGTTGACAGGACACGAGTAACTCGTCTATCTT 174
hCoV-19/Switzerland/VD-CHUV-8604552682/2025|EPI_ISL_19906527|2025-05-19  CGCAGTATAATTAATAACTAATTACTGTCGTTGACAGGACACGAGTAACTCGTCTATCTT 179
hCoV-19/Scotland/CLIMB-CM7YR3YO/2025|EPI_ISL_19896015|2025-05-24         CGCAGTATAATTAATAACTAATTACTGTCGTTGACAGGACACGAGTAACTCGTCTATCTT 180
hCoV-19/Sweden/O-18_SE100_25CS500180/2025|EPI_ISL_19866288|2025-04-15    CGCAGTATAATTAATAACTAATTACTGTCGTTGACAGGACACGAGTAACTCGTCTATCTT 180
hCoV-19/Ireland/D-CS250088/2025|EPI_ISL_19877457|2025-04-16              CGCAGTATAATTAATAACTAATTACTGTCGTTGACAGGACACGAGTAACTCGTCTATCTT 180
hCoV-19/Ireland/G-LUH-N3442/2025|EPI_ISL_19893151|2025-05-07             CGCAGTATAATTAATAACTAATTACTGTCGTTGACAGGACACGAGTAACTCGTCTATCTT 180
hCoV-19/Scotland/CLIMB-CM7YMZ5M/2025|EPI_ISL_19906439|2025-05-22         CGCAGTATAATTAATAACTAATTACTGTCGTTGACAGGACACGAGTAACTCGTCTATCTT 179
hCoV-19/Scotland/CLIMB-CM7YR3ZG/2025|EPI_ISL_19897722|2025-05-13         CGCAGTATAATTAATAACTAATTACTGTCGTTGACAGGACACGAGTAACTCGTCTATCTT 178
hCoV-19/Scotland/CLIMB-CM7YEYGT/2025|EPI_ISL_19897741|2025-04-28         CGCAGTATAATTAATAACTAATTACTGTCGTTGACAGGACACGAGTAACTCGTCTATCTT 178
hCoV-19/Scotland/CLIMB-CM7YGAJ3/2025|EPI_ISL_19895963|2025-05-13         CGCAGTATAATTAATAACTAATTACTGTCGTTGACAGGACACGAGTAACTCGTCTATCTT 167
hCoV-19/Scotland/CLIMB-CM7YJ1ZO/2025|EPI_ISL_19897757|2025-05-14         CGCAGTATAATTAATAACTAATTACTGTCGTTGACAGGACACGAGTAACTCGTCTATCTT 168
hCoV-19/Scotland/CLIMB-CM7Y8C4Y/2025|EPI_ISL_19906404|2025-05-15         CGCAGTATAATTAATAACTAATTACTGTCGTTGACAGGACACGAGTAACTCGTCTATCTT 175
```

hCoV-19/Northern_Ireland/CLIMB-CM7YGGYM/2025|EPI_ISL_19897641|2025-05-23	CGCAGTATAATTAATAACTAATTACTGTCGTTGACAGGACACGAGTAACTCGTCTATCTT	170
hCoV-19/Scotland/CLIMB-CM7YFW7M/2025|EPI_ISL_19881718|2025-05-10	CGCAGTATAATTAATAACTAATTACTGTCGTTGACAGGACACGAGTAACTCGTCTATCTT	173
hCoV-19/Scotland/CLIMB-CM7YEMXP/2025|EPI_ISL_19888387|2025-05-10	CGCAGTATAATTAATAACTAATTACTGTCGTTGACAGGACACGAGTAACTCGTCTATCTT	172
hCoV-19/Spain/CT-HUVH-E593511/2025|EPI_ISL_19881731|2025-05-06	CGCAGTATAATTAATAACTAATTACTGTCGTTGACAGGACACGAGTAACTCGTCTATCTT	125
hCoV-19/Spain/CT-HUJT-E325362/2025|EPI_ISL_19889935|2025-05-20	CGCAGTATAATTAATAACTAATTACTGTCGTTGACAGGACACGAGTAACTCGTCTATCTT	126
hCoV-19/Spain/PV-HUD-84743204/2025|EPI_ISL_19907746|2025-06-06	CGCAGTATAATTAATAACTAATTACTGTCGTTGACAGGACACGAGTAACTCGTCTATCTT	127
hCoV-19/Ireland/D-SVUH-FBB21492-54/2025|EPI_ISL_19881758|2025-05-11	CGCAGTATAATTAATAACTAATTACTGTCGTTGACAGGACACGAGTAACTCGTCTATCTT	128
hCoV-19/Spain/CT-HUVH-E596725/2025|EPI_ISL_19881729|2025-05-10	CGCAGTATAATTAATAACTAATTACTGTCGTTGACAGGACACGAGTAACTCGTCTATCTT	122
hCoV-19/Spain/CT-HUVH-E593882/2025|EPI_ISL_19881732|2025-05-06	CGCAGTATAATTAATAACTAATTACTGTCGTTGACAGGACACGAGTAACTCGTCTATCTT	122
hCoV-19/Finland/THL-02385/2025|EPI_ISL_19875928|2025-04-16	CGCAGTATAATTAATAACTAATTACTGTCGTTGACAGGACACGAGTAACTCGTCTATCTT	129
hCoV-19/Ireland/D-BH-02351167/2025|EPI_ISL_19911337|2025-05-28	CGCAGTATAATTAATAACTAATTACTGTCGTTGACAGGACACGAGTAACTCGTCTATCTT	128
hCoV-19/Ireland/D-NVRL-XIRL00001782/2025|EPI_ISL_19896104|2025-05-14	CGCAGTATAATTAATAACTAATTACTGTCGTTGACAGGACACGAGTAACTCGTCTATCTT	128
hCoV-19/Ireland/TA-NVRL-GIRL00039444/2025|EPI_ISL_19896118|2025-05-16	CGCAGTATAATTAATAACTAATTACTGTCGTTGACAGGACACGAGTAACTCGTCTATCTT	128
hCoV-19/Spain/GA-CHUAC-13223/2025|EPI_ISL_19894946|2025-05-31	CGCAGTATAATTAATAACTAATTACTGTCGTTGACAGGACACGAGTAACTCGTCTATCTT	127
hCoV-19/Spain/GA-CHUAC-13215/2025|EPI_ISL_19894939|2025-05-29	CGCAGTATAATTAATAACTAATTACTGTCGTTGACAGGACACGAGTAACTCGTCTATCTT	127
hCoV-19/Finland/THL-02398/2025|EPI_ISL_19875939|2025-05-04	CGCAGTATAATTAATAACTAATTACTGTCGTTGACAGGACACGAGTAACTCGTCTATCTT	128
hCoV-19/Finland/THL-02400/2025|EPI_ISL_19875941|2025-05-03	CGCAGTATAATTAATAACTAATTACTGTCGTTGACAGGACACGAGTAACTCGTCTATCTT	128
hCoV-19/Ireland/LK-UHL-702929/2025|EPI_ISL_19877895|2025-04-16	CGCAGTATAATTAATAACTAATTACTGTCGTTGACAGGACACGAGTAACTCGTCTATCTT	128
hCoV-19/Ireland/LK-UHL-703788/2025|EPI_ISL_19877905|2025-05-03	CGCAGTATAATTAATAACTAATTACTGTCGTTGACAGGACACGAGTAACTCGTCTATCTT	128
hCoV-19/Ireland/LK-UHL-703851/2025|EPI_ISL_19877907|2025-05-04	CGCAGTATAATTAATAACTAATTACTGTCGTTGACAGGACACGAGTAACTCGTCTATCTT	128
hCoV-19/Ireland/LK-UHL-703889/2025|EPI_ISL_19877908|2025-05-05	CGCAGTATAATTAATAACTAATTACTGTCGTTGACAGGACACGAGTAACTCGTCTATCTT	128
hCoV-19/Ireland/D-SVUH-FBB21492-49/2025|EPI_ISL_19881756|2025-05-14	CGCAGTATAATTAATAACTAATTACTGTCGTTGACAGGACACGAGTAACTCGTCTATCTT	128
hCoV-19/Ireland/D-SVUH-FBB21492-59/2025|EPI_ISL_19881763|2025-05-07	CGCAGTATAATTAATAACTAATTACTGTCGTTGACAGGACACGAGTAACTCGTCTATCTT	128
hCoV-19/Spain/CT-HUGTiP-G3R212/2025|EPI_ISL_19883055|2025-05-20	CGCAGTATAATTAATAACTAATTACTGTCGTTGACAGGACACGAGTAACTCGTCTATCTT	128
hCoV-19/Ireland/LK-UHL-704398/2025|EPI_ISL_19888621|2025-05-16	CGCAGTATAATTAATAACTAATTACTGTCGTTGACAGGACACGAGTAACTCGTCTATCTT	128
hCoV-19/Ireland/G-GUH-N3466/2025|EPI_ISL_19893175|2025-05-03	CGCAGTATAATTAATAACTAATTACTGTCGTTGACAGGACACGAGTAACTCGTCTATCTT	128
hCoV-19/Belgium/Sciensano-LS-S5276/2025|EPI_ISL_19893834|2025-04-22	CGCAGTATAATTAATAACTAATTACTGTCGTTGACAGGACACGAGTAACTCGTCTATCTT	128
hCoV-19/Italy/VEN-ULSS8-0019244_VI/2025|EPI_ISL_19899362|2025-05-26	CGCAGTATAATTAATAACTAATTACTGTCGTTGACAGGACACGAGTAACTCGTCTATCTT	128
hCoV-19/Spain/CT-HUVH-E618109/2025|EPI_ISL_19907773|2025-06-10	CGCAGTATAATTAATAACTAATTACTGTCGTTGACAGGACACGAGTAACTCGTCTATCTT	128
hCoV-19/Ireland/D-BH-03819149/2025|EPI_ISL_19911343|2025-05-29	CGCAGTATAATTAATAACTAATTACTGTCGTTGACAGGACACGAGTAACTCGTCTATCTT	128
hCoV-19/Ireland/D-BH-03993161/2025|EPI_ISL_19911355|2025-06-10	CGCAGTATAATTAATAACTAATTACTGTCGTTGACAGGACACGAGTAACTCGTCTATCTT	128
hCoV-19/Spain/CT-HUVH-E587040/2025|EPI_ISL_19871881|2025-04-26	CGCAGTATAATTAACAACTAATTACTGTCGTTGACAGGACACGAGTAACTCGTCTATCTT	127
hCoV-19/Finland/THL-02392/2025|EPI_ISL_19875934|2025-05-02	CGCAGTATAATTAATAACTAATTACTGTCGTTGACAGGACACGAGTAACTCGTCTATCTT	127
hCoV-19/Spain/GA-CHUAC-13181/2025|EPI_ISL_19890617|2025-05-20	CGCAGTATAATTAATAACTAATTACTGTCGTTGACAGGACACGAGTAACTCGTCTATCTT	127
hCoV-19/Spain/CT-HUVH-E614843/2025|EPI_ISL_19907763|2025-06-05	CGCAGTATAATTAATAACTAATTACTGTCGTTGACAGGACACGAGTAACTCGTCTATCTT	127
hCoV-19/Italy/UMB-LAOP-172-4-02/2025|EPI_ISL_19899474|2025-05-08	CGCAGTATAATTAATAACTAATTACTGTCGTTGACAGGACACGAGTAACTCGTCTATCTT	149
hCoV-19/Spain/NC-CHN-01008932/2025|EPI_ISL_19900595|2025-04-04	CGCAGTATAATTAATAACTAATTACTGTCGTTGACAGGACACGAGTAACTCGTCTATCTT	151
hCoV-19/Northern_Ireland/CLIMB-CM7YGDRO/2025|EPI_ISL_19877331|2025-04-14	CGCAGTATAATTAATAACTAATTACTGTCGTTGACAGGACACGAGTAACTCGTCTATCTT	139
hCoV-19/Scotland/CLIMB-CM7YEE96/2025|EPI_ISL_19897754|2025-05-13	CGCAGTATAATTAATAACTAATTACTGTCGTTGACAGGACACGAGTAACTCGTCTATCTT	138
hCoV-19/France/ARA-HCL125018289001/2025|EPI_ISL_19880005|2025-05-12	CGCAGTATAATTAATAACTAATTACTGTCGTTGACAGGACACGAGTAACTCGTCTATCTT	132
hCoV-19/France/ARA-RELAB-HCL725000057501/2025|EPI_ISL_19880014|2025-04-28	CGCAGTATAATTAATAACTAATTACTGTCGTTGACAGGACACGAGTAACTCGTCTATCTT	132
hCoV-19/France/ARA-RELAB-HCL725000059501/2025|EPI_ISL_19885160|2025-04-25	CGCAGTATAATTAATAACTAATTACTGTCGTTGACAGGACACGAGTAACTCGTCTATCTT	132
hCoV-19/France/NAQ-RELAB-HCL725000061001/2025|EPI_ISL_19885161|2025-05-10	CGCAGTATAATTAATAACTAATTACTGTCGTTGACAGGACACGAGTAACTCGTCTATCTT	132
hCoV-19/France/NAQ-RELAB-HCL725000060901/2025|EPI_ISL_19885180|2025-05-09	CGCAGTATAATTAATAACTAATTACTGTCGTTGACAGGACACGAGTAACTCGTCTATCTT	132
hCoV-19/France/ARA-HCL025075546601/2025|EPI_ISL_19885182|2025-05-09	CGCAGTATAATTAATAACTAATTACTGTCGTTGACAGGACACGAGTAACTCGTCTATCTT	132
hCoV-19/France/ARA-HCL125018461901/2025|EPI_ISL_19885204|2025-05-14	CGCAGTATAATTAATAACTAATTACTGTCGTTGACAGGACACGAGTAACTCGTCTATCTT	132
hCoV-19/France/ARA-HCL125018655901/2025|EPI_ISL_19885206|2025-05-14	CGCAGTATAATTAATAACTAATTACTGTCGTTGACAGGACACGAGTAACTCGTCTATCTT	132
hCoV-19/France/ARA-HCL125019155501/2025|EPI_ISL_19885209|2025-05-18	CGCAGTATAATTAATAACTAATTACTGTCGTTGACAGGACACGAGTAACTCGTCTATCTT	132
hCoV-19/France/ARA-HCL125019240901/2025|EPI_ISL_19885210|2025-05-19	CGCAGTATAATTAATAACTAATTACTGTCGTTGACAGGACACGAGTAACTCGTCTATCTT	132
hCoV-19/France/ARA-HCL125019249201/2025|EPI_ISL_19885211|2025-05-19	CGCAGTATAATTAATAACTAATTACTGTCGTTGACAGGACACGAGTAACTCGTCTATCTT	132
hCoV-19/France/ARA-HCL125019399701/2025|EPI_ISL_19885212|2025-05-20	CGCAGTATAATTAATAACTAATTACTGTCGTTGACAGGACACGAGTAACTCGTCTATCTT	132
hCoV-19/France/ARA-HCL125019408901/2025|EPI_ISL_19885213|2025-05-20	CGCAGTATAATTAATAACTAATTACTGTCGTTGACAGGACACGAGTAACTCGTCTATCTT	132
hCoV-19/France/OCC-RELAB-HCL725000064001/2025|EPI_ISL_19895004|2025-05-14	CGCAGTATAATTAATAACTAATTACTGTCGTTGACAGGACACGAGTAACTCGTCTATCTT	132

hCoV-19/France/OCC-RELAB-HCL725000064101/2025|EPI_ISL_19895005|2025-05-14 CGCAGTATAATTAATAACTAATTACTGTCGTTGACAGGACACGAGTAACTCGTCTATCTT 132
hCoV-19/France/NAQ-RELAB-HCL725000062601/2025|EPI_ISL_19895009|2025-05-16 CGCAGTATAATTAATAACTAATTACTGTCGTTGACAGGACACGAGTAACTCGTCTATCTT 132
hCoV-19/France/ARA-RELAB-HCL725000062401/2025|EPI_ISL_19895017|2025-05-16 CGCAGTATAATTAATAACTAATTACTGTCGTTGACAGGACACGAGTAACTCGTCTATCTT 132
hCoV-19/France/ARA-RELAB-HCL725000062101/2025|EPI_ISL_19895018|2025-05-13 CGCAGTATAATTAATAACTAATTACTGTCGTTGACAGGACACGAGTAACTCGTCTATCTT 132
hCoV-19/France/ARA-HCL125019625101/2025|EPI_ISL_19895034|2025-05-21 CGCAGTATAATTAATAACTAATTACTGTCGTTGACAGGACACGAGTAACTCGTCTATCTT 132
hCoV-19/France/ARA-HCL125019754801/2025|EPI_ISL_19895035|2025-05-22 CGCAGTATAATTAATAACTAATTACTGTCGTTGACAGGACACGAGTAACTCGTCTATCTT 132
hCoV-19/France/ARA-RELAB-HCL725000064601/2025|EPI_ISL_19895041|2025-05-15 CGCAGTATAATTAATAACTAATTACTGTCGTTGACAGGACACGAGTAACTCGTCTATCTT 132
hCoV-19/France/ARA-RELAB-HCL725000066001/2025|EPI_ISL_19895046|2025-05-13 CGCAGTATAATTAATAACTAATTACTGTCGTTGACAGGACACGAGTAACTCGTCTATCTT 132
hCoV-19/France/OCC-RELAB-HCL725000068701/2025|EPI_ISL_19905466|2025-05-21 CGCAGTATAATTAATAACTAATTACTGTCGTTGACAGGACACGAGTAACTCGTCTATCTT 132
hCoV-19/France/OCC-RELAB-HCL725000069001/2025|EPI_ISL_19905468|2025-05-21 CGCAGTATAATTAATAACTAATTACTGTCGTTGACAGGACACGAGTAACTCGTCTATCTT 132
hCoV-19/France/ARA-HCL025089279801/2025|EPI_ISL_19905469|2025-06-02 CGCAGTATAATTAATAACTAATTACTGTCGTTGACAGGACACGAGTAACTCGTCTATCTT 132
hCoV-19/France/PAC-RELAB-HCL725000069901/2025|EPI_ISL_19908847|2025-05-26 CGCAGTATAATTAATAACTAATTACTGTCGTTGACAGGACACGAGTAACTCGTCTATCTT 132
hCoV-19/France/PAC-RELAB-HCL725000069501/2025|EPI_ISL_19908849|2025-05-30 CGCAGTATAATTAATAACTAATTACTGTCGTTGACAGGACACGAGTAACTCGTCTATCTT 132
hCoV-19/France/ARA-RELAB-HCL725000071701/2025|EPI_ISL_19908858|2025-05-22 CGCAGTATAATTAATAACTAATTACTGTCGTTGACAGGACACGAGTAACTCGTCTATCTT 132
hCoV-19/France/ARA-HCL025093352901/2025|EPI_ISL_19908867|2025-06-10 CGCAGTATAATTAATAACTAATTACTGTCGTTGACAGGACACGAGTAACTCGTCTATCTT 132
hCoV-19/France/ARA-RELAB-HCL725000065801/2025|EPI_ISL_19895045|2025-05-16 CGCAGTATAATTAATAACTAATTACTGTCGTTGACAGGACACGAGTAACTCGTCTATCTT 132
hCoV-19/Denmark/DCGC-692234/2025|EPI_ISL_19879694|2025-05-05 CGCAGTATAATTAATAACTAATTACTGTCGTTGACAGGACACGAGTAACTCGTCTATCTT 134
hCoV-19/Finland/THL-02388/2025|EPI_ISL_19875931|2025-04-16 CGCAGTATAATTAATAACTAATTACTGTCGTTGACAGGACACGAGTAACTCGTCTATCTT 135
hCoV-19/Spain/PV-HUD-98024244/2025|EPI_ISL_19907759|2025-06-09 CGCAGTATAATTAATAACTAATTACTGTCGTTGACAGGACACGAGTAACTCGTCTATCTT 135
hCoV-19/Netherlands/GE-RIVM-145386/2025|EPI_ISL_19873764|2025-05-01 CGCAGTATAATTAATAACTAATTACTGTCGTTGACAGGACACGAGTAACTCGTCTATCTT 132
hCoV-19/Netherlands/ZH-RIVM-145413/2025|EPI_ISL_19891397|2025-04-30 CGCAGTATAATTAATAACTAATTACTGTCGTTGACAGGACACGAGTAACTCGTCTATCTT 132
hCoV-19/Netherlands/NH-RIVM-145458/2025|EPI_ISL_19905062|2025-05-25 CGCAGTATAATTAATAACTAATTACTGTCGTTGACAGGACACGAGTAACTCGTCTATCTT 132
hCoV-19/Netherlands/NH-RIVM-145463/2025|EPI_ISL_19905063|2025-05-20 CGCAGTATAATTAATAACTAATTACTGTCGTTGACAGGACACGAGTAACTCGTCTATCTT 132
hCoV-19/Netherlands/UT-RIVM-145436/2025|EPI_ISL_19905072|2025-05-15 CGCAGTATAATTAATAACTAATTACTGTCGTTGACAGGACACGAGTAACTCGTCTATCTT 132
hCoV-19/Netherlands/UT-RIVM-145470/2025|EPI_ISL_19905077|2025-06-02 CGCAGTATAATTAATAACTAATTACTGTCGTTGACAGGACACGAGTAACTCGTCTATCTT 132
hCoV-19/Netherlands/ZH-RIVM-145478/2025|EPI_ISL_19905094|2025-05-30 CGCAGTATAATTAATAACTAATTACTGTCGTTGACAGGACACGAGTAACTCGTCTATCTT 132
hCoV-19/Netherlands/ZH-RIVM-145480/2025|EPI_ISL_19905096|2025-05-31 CGCAGTATAATTAATAACTAATTACTGTCGTTGACAGGACACGAGTAACTCGTCTATCTT 132
hCoV-19/Netherlands/UT-RIVM-145481/2025|EPI_ISL_19905078|2025-05-31 CGCAGTATAATTAATAACTAATTACTGTCGTTGACAGGACACGAGTAACTCGTCTATCTT 132
hCoV-19/Scotland/CLIMB-CM7YRW9H/2025|EPI_ISL_19881711|2025-05-06 CGCAGTATAATTAATAACTAATTACTGTCGTTGACAGGACACGAGTAACTCGTCTATCTT 104
hCoV-19/France/IDF-RELAB-IPP04872/2025|EPI_ISL_19911293|2025-05-22 CGCAGTATAATTAATAACTAATTACTGTCGTTGACAGGACACGAGTAACTCGTCTATCTT 100
hCoV-19/Canary_Islands/CN-HUC-925112913/2025|EPI_ISL_19900668|2025-04-22 CGCAGTATAATTAATAACTAATTACTGTCGTTGACAGGACACGAGTAACTCGTCTATCTT 108
hCoV-19/Scotland/CLIMB-CM7YJ5BB/2025|EPI_ISL_19906458|2025-05-27 CGCAGTATAATTAATAACTAATTACTGTCGTTGACAGGACACGAGTAACTCGTCTATCTT 104
hCoV-19/Scotland/CLIMB-CM7Y8PHH/2025|EPI_ISL_19897755|2025-05-14 CGCAGTATAATTAATAACTAATTACTGTCGTTGACAGGACACGAGTAACTCGTCTATCTT 104
hCoV-19/Spain/AN-PMC-42706/2025|EPI_ISL_19888325|2025-05-25 CGCAGTATAATTAATAACTAATTACTGTCGTTGACAGGACACGAGTAACTCGTCTATCTT 104
hCoV-19/Scotland/CLIMB-CM7YRIA1/2025|EPI_ISL_19869961|2025-04-20 CGCAGTATAATTAATAACTAATTACTGTCGTTGACAGGACACGAGTAACTCGTCTATCTT 104
hCoV-19/Scotland/CLIMB-CM7Y8UWT/2025|EPI_ISL_19869966|2025-04-21 CGCAGTATAATTAATAACTAATTACTGTCGTTGACAGGACACGAGTAACTCGTCTATCTT 104
hCoV-19/England/CLIMB-CM7YMFHB/2025|EPI_ISL_19877436|2025-05-10 CGCAGTATAATTAATAACTAATTACTGTCGTTGACAGGACACGAGTAACTCGTCTATCTT 104
hCoV-19/Denmark/DCGC-692213/2025|EPI_ISL_19879673|2025-04-28 CGCAGTATAATTAATAACTAATTACTGTCGTTGACAGGACACGAGTAACTCGTCTATCTT 104
hCoV-19/Wales/CLIMB-CM7YRAXQ/2025|EPI_ISL_19883075|2025-05-14 CGCAGTATAATTAATAACTAATTACTGTCGTTGACAGGACACGAGTAACTCGTCTATCTT 104
hCoV-19/Spain/AN-PMC-42696/2025|EPI_ISL_19888316|2025-05-14 CGCAGTATAATTAATAACTAATTACTGTCGTTGACAGGACACGAGTAACTCGTCTATCTT 104
hCoV-19/Spain/AN-PMC-42719/2025|EPI_ISL_19888338|2025-05-15 CGCAGTATAATTAATAACTAATTACTGTCGTTGACAGGACACGAGTAACTCGTCTATCTT 104
hCoV-19/England/CLIMB-CM7YE7HB/2025|EPI_ISL_19892115|2025-05-19 CGCAGTATAATTAATAACTAATTACTGTCGTTGACAGGACACGAGTAACTCGTCTATCTT 104
hCoV-19/France/GES-RELAB-IPP04599/2025|EPI_ISL_19893547|2025-05-05 CGCAGTATAATTAATAACTAATTACTGTCGTTGACAGGACACGAGTAACTCGTCTATCTT 104
hCoV-19/Scotland/CLIMB-CM7YEPXZ/2025|EPI_ISL_19896028|2025-05-25 CGCAGTATAATTAATAACTAATTACTGTCGTTGACAGGACACGAGTAACTCGTCTATCTT 104
hCoV-19/Scotland/CLIMB-CM7YF4QT/2025|EPI_ISL_19896032|2025-05-27 CGCAGTATAATTAATAACTAATTACTGTCGTTGACAGGACACGAGTAACTCGTCTATCTT 104
hCoV-19/Scotland/CLIMB-CM7YKDZ6/2025|EPI_ISL_19896033|2025-05-27 CGCAGTATAATTAATAACTAATTACTGTCGTTGACAGGACACGAGTAACTCGTCTATCTT 104
hCoV-19/Scotland/CLIMB-CM7YG8H4/2025|EPI_ISL_19897748|2025-05-02 CGCAGTATAATTAATAACTAATTACTGTCGTTGACAGGACACGAGTAACTCGTCTATCTT 104
hCoV-19/Wales/CLIMB-CM7YJXNZ/2025|EPI_ISL_19900036|2025-05-26 CGCAGTATAATTAATAACTAATTACTGTCGTTGACAGGACACGAGTAACTCGTCTATCTT 104
hCoV-19/Germany/NW-RKI-I-1148178/2025|EPI_ISL_19902055|2025-05-21 CGCAGTATAATTAATAACTAATTACTGTCGTTGACAGGACACGAGTAACTCGTCTATCTT 104
hCoV-19/Germany/NI-RKI-I-1148183/2025|EPI_ISL_19902060|2025-05-18 CGCAGTATAATTAATAACTAATTACTGTCGTTGACAGGACACGAGTAACTCGTCTATCTT 104
hCoV-19/Germany/NW-RKI-I-1148191/2025|EPI_ISL_19902068|2025-05-19 CGCAGTATAATTAATAACTAATTACTGTCGTTGACAGGACACGAGTAACTCGTCTATCTT 104
hCoV-19/Spain/MD-HGUGM-66414184/2025|EPI_ISL_19906298|2025-05-25 CGCAGTATAATTAATAACTAATTACTGTCGTTGACAGGACACGAGTAACTCGTCTATCTT 104
hCoV-19/Spain/MD-HGUGM-66400167/2025|EPI_ISL_19906320|2025-05-22 CGCAGTATAATTAATAACTAATTACTGTCGTTGACAGGACACGAGTAACTCGTCTATCTT 104

```
hCoV-19/France/IDF-RELAB-IPP04646/2025|EPI_ISL_19907194|2025-05-16        CGCAGTATAATTAATAACTAATTACTGTCGTTGACAGGACACGAGTAACTCGTCTATCTT 104
hCoV-19/England/CLIMB-CM7YKIUQ/2025|EPI_ISL_19908573|2025-06-07           CGCAGTATAATTAATAACTAATTACTGTCGTTGACAGGACACGAGTAACTCGTCTATCTT 104
hCoV-19/France/GES-RELAB-IPP04823/2025|EPI_ISL_19911257|2025-05-26        CGCAGTATAATTAATAACTAATTACTGTCGTTGACAGGACACGAGTAACTCGTCTATCTT 104
hCoV-19/France/IDF-RELAB-IPP04863/2025|EPI_ISL_19911265|2025-05-24        CGCAGTATAATTAATAACTAATTACTGTCGTTGACAGGACACGAGTAACTCGTCTATCTT 104
hCoV-19/Denmark/DCGC-692245/2025|EPI_ISL_19879704|2025-04-28              CGCAGTATAATTAATAACTAATTACTGTCGTTGACAGGACACGAGTAACTCGTCTATCTT 104


hCoV-19/Ireland/D-SVUH-FBA98650-31/2025|EPI_ISL_19866548|2025-05-04       ------------------------------------------------------------ 0
hCoV-19/Spain/PV-HUB-5628663/2025|EPI_ISL_19908839|2025-05-16             -----------------------CCGTGTTGCAGCCGATCATCAGCACATCTAGGTTTTG 37
hCoV-19/Spain/PV-HUB-41587498/2025|EPI_ISL_19908930|2025-05-20            ----------------------------TTGCAGCCGATCATCAGCACATCTAGGTTTTG 32
hCoV-19/Spain/PV-HUB-56291851/2025|EPI_ISL_19908970|2025-05-31            ---------------------------GTTGCAGCCGATCATCAGCACATCTAGGTTTTG 33
hCoV-19/Northern_Ireland/CLIMB-CM7YR8I6/2025|EPI_ISL_19877334|2025-04-23  CTGCAGGCTGCTTACGGTTTCGTCCGTGTTGCAGCCGATCATCAGCACATCTAGGTTTTG 141
hCoV-19/Germany/NW-RKI-I-1148136/2025|EPI_ISL_19871950|2025-04-23         CTGCAGGCTGCTTACGGTTTCGTCCGTGTTGCAGCCGATCATCAGCACATCTAGGTTTTG 164
hCoV-19/Germany/NW-RKI-I-1148141/2025|EPI_ISL_19871955|2025-04-23         CTGCAGGCTGCTTACGGTTTCGTCCGTGTTGCAGCCGATCATCAGCACATCTAGGTTTTG 164
hCoV-19/Germany/BW-RKI-I-1148164/2025|EPI_ISL_19884987|2025-04-17         CTGCAGGCTGCTTACGGTTTCGTCCGTGTTGCAGCCGATCATCAGCACATCTAGGTTTTG 164
hCoV-19/Scotland/CLIMB-CM7YFGW7/2025|EPI_ISL_19897691|2025-05-24          CTGCAGGCTGCTTACGGTTTCGTCCGTGTTGCAGCCGATCATCAGCACATCTAGGTTTTG 164
hCoV-19/Ireland/G-LUH-N3473/2025|EPI_ISL_19893181|2025-05-14              CTGCAGGCTGCTTACGGTTTCGTCCGTGTTGCAGCCGATCATCAGCACATCTAGGTTTTG 231
hCoV-19/Spain/AN-PMC-42728/2025|EPI_ISL_19888347|2025-05-25               CTGCAGGCTGCTTACGGTTTCGTCCGTGTTGCAGCCGATCATCAGCACATCTAGGTTTTG 232
hCoV-19/Sweden/00200866492N2/2025|EPI_ISL_19870050|2025-04-22             CTGCAGGCTGCTTACGGTTTCGTCCGTGTTGCAGCCGATCATCAGCACATCTAGGTTTTG 238
hCoV-19/Sweden/00200867159N2/2025|EPI_ISL_19870052|2025-04-23             CTGCAGGCTGCTTACGGTTTCGTCCGTGTTGCAGCCGATCATCAGCACATCTAGGTTTTG 238
hCoV-19/Ireland/G-LUH-N3445/2025|EPI_ISL_19893154|2025-05-07              CTGCAGGCTGCTTACGGTTTCGTCCGTGTTGCAGCCGATCATCAGCACATCTAGGTTTTG 238
hCoV-19/Ireland/G-LUH-N3443/2025|EPI_ISL_19893152|2025-05-07              CTGCAGGCTGCTTACGGTTTCGTCCGTGTTGCAGCCGATCATCAGCACATCTAGGTTTTG 236
hCoV-19/Scotland/CLIMB-CM7YF9PJ/2025|EPI_ISL_19895983|2025-05-19          CTGCAGGCTGCTTACGGTTTCGTCCGTGTTGCAGCCGATCATCAGCACATCTAGGTTTTG 235
hCoV-19/Scotland/CLIMB-CM7YKMK8/2025|EPI_ISL_19869976|2025-04-25          CTGCAGGCTGCTTACGGTTTCGTCCGTGTTGCAGCCGATCATCAGCACATCTAGGTTTTG 234
hCoV-19/Ireland/G-LUH-N3444/2025|EPI_ISL_19893153|2025-05-07              CTGCAGGCTGCTTACGGTTTCGTCCGTGTTGCAGCCGATCATCAGCACATCTAGGTTTTG 234
hCoV-19/Switzerland/VD-CHUV-8604552682/2025|EPI_ISL_19906527|2025-05-19   CTGCAGGCTGCTTACGGTTTCGTCCGTGTTGCAGCCGATCATCAGCACATCTAGGTTTTG 239
hCoV-19/Scotland/CLIMB-CM7YR3YO/2025|EPI_ISL_19896015|2025-05-24          CTGCAGGCTGCTTACGGTTTCGTCCGTGTTGCAGCCGATCATCAGCACATCTAGGTTTTG 240
hCoV-19/Sweden/O-18_SE100_25CS500180/2025|EPI_ISL_19866288|2025-04-15    CTGCAGGCTGCTTACGGTTTCGTCCGTGTTGCAGCCGATCATCAGCACATCTAGGTTTTG 240
hCoV-19/Ireland/D-CS250088/2025|EPI_ISL_19877457|2025-04-16               CTGCAGGCTGCTTACGGTTTCGTCCGTGTTGCAGCCGATCATCAGCACATCTAGGTTTTG 240
hCoV-19/Ireland/G-LUH-N3442/2025|EPI_ISL_19893151|2025-05-07              CTGCAGGCTGCTTACGGTTTCGTCCGTGTTGCAGCCGATCATCAGCACATCTAGGTTTTG 240
hCoV-19/Scotland/CLIMB-CM7YMZ5M/2025|EPI_ISL_19906439|2025-05-22          CTGCAGGCTGCTTACGGTTTCGTCCGTGTTGCAGCCGATCATCAGCACATCTAGGTTTTG 239
hCoV-19/Scotland/CLIMB-CM7YR3ZG/2025|EPI_ISL_19897722|2025-05-13          CTGCAGGCTGCTTACGGTTTCGTCCGTGTTGCAGCCGATCATCAGCACATCTAGGTTTTG 238
hCoV-19/Scotland/CLIMB-CM7YEYGT/2025|EPI_ISL_19897741|2025-04-28          CTGCAGGCTGCTTACGGTTTCGTCCGTGTTGCAGCCGATCATCAGCACATCTAGGTTTTG 238
hCoV-19/Scotland/CLIMB-CM7YGAJ3/2025|EPI_ISL_19895963|2025-05-13          CTGCAGGCTGCTTACGGTTTCGTCCGTGTTGCAGCCGATCATCAGCACATCTAGGTTTTG 227
hCoV-19/Scotland/CLIMB-CM7YJ1ZO/2025|EPI_ISL_19897757|2025-05-14          CTGCAGGCTGCCTACGGTTTCGTCCGTGTTGCAGCCGATCATCAGCACATCTAGGTTTTG 228
hCoV-19/Scotland/CLIMB-CM7Y8C4Y/2025|EPI_ISL_19906404|2025-05-15          CTGCAGGCTGCTTACGGTTTCGTCCGTGTTGCAGCCGATCATCAGCACATCTAGGTTTTG 235
hCoV-19/Northern_Ireland/CLIMB-CM7YGGYM/2025|EPI_ISL_19897641|2025-05-23  CTGCAGGCTGCTTACGGTTTCGTCCGTGTTGCAGCCGATCATCAGCACATCTAGGTTTTG 230
hCoV-19/Scotland/CLIMB-CM7YFW7M/2025|EPI_ISL_19881718|2025-05-10          CTGCAGGCTGCTTACGGTTTCGTCCGTGTTGCAGCCGATCATCAGCACATCTAGGTTTTG 233
hCoV-19/Scotland/CLIMB-CM7YEMXP/2025|EPI_ISL_19888387|2025-05-10          CTGCAGGCTGCTTACGGTTTCGTCCGTGTTGCAGCCGATCATCAGCACATCTAGGTTTTG 232
hCoV-19/Spain/CT-HUVH-E593511/2025|EPI_ISL_19881731|2025-05-06            CTGCAGGCTGCTTACGGTTTCGTCCGTGTTGCAGCCGATCATCAGCACATCTAGGTTTTG 185
hCoV-19/Spain/CT-HUJT-E325362/2025|EPI_ISL_19889935|2025-05-20            CTGCAGGCTGCTTACGGTTTCGTCCGTGTTGCAGCCGATCATCAGCACATCTAGGTTTTG 186
hCoV-19/Spain/PV-HUD-84743204/2025|EPI_ISL_19907746|2025-06-06            CTGCAGGCTGCTTACGGTTTCGTCCGTGTTGCAGCCGATCATCAGCACATCTAGGTTTTG 187
hCoV-19/Ireland/D-SVUH-FBB21492-54/2025|EPI_ISL_19881758|2025-05-11       CTGCAGGCTGCTTACGGTTTCGTCCGTGTTGCAGCCGATCATCAGCACATCTAGGTTTTG 188
hCoV-19/Spain/CT-HUVH-E596725/2025|EPI_ISL_19881729|2025-05-10            CTGCAGGCTGCTTACGGTTTCGTCCGTGTTGCAGCCGATCATCAGCACATCTAGGTTTTG 182
hCoV-19/Spain/CT-HUVH-E593882/2025|EPI_ISL_19881732|2025-05-06            CTGCAGGCTGCTTACGGTTTCGTCCGTGTTGCAGCCGATCATCAGCACATCTAGGTTTTG 182
hCoV-19/Finland/THL-02385/2025|EPI_ISL_19875928|2025-04-16                CTGCAGGCTGCTTACGGTTTCGTCCGTGTTGCAGTCGATCATCAGCACATCTAGGTTTTG 189
hCoV-19/Ireland/D-BH-02351167/2025|EPI_ISL_19911337|2025-05-28            CTGCAGGCTGCTTACGGTTTCGTCCGTGTTGCAGCCGATCATCAGCACATCTAGGTTTTG 188
hCoV-19/Ireland/D-NVRL-XIRL00001782/2025|EPI_ISL_19896104|2025-05-14      CTGCAGGCTGCTTACGGTTTCGTCCGTGTTGCAGCCGATCATCAGCACATCTAGGTTTTG 188
hCoV-19/Ireland/TA-NVRL-GIRL00039444/2025|EPI_ISL_19896118|2025-05-16     CTGCAGGCTGCTTACGGTTTCGTCCGTGTTGCAGCCGATCATCAGCACATCTAGGTTTTG 188
hCoV-19/Spain/GA-CHUAC-13223/2025|EPI_ISL_19894946|2025-05-31             CTGCAGGCTGCTTACGGTTTCGTCCGTGTTGCAGCCGATCATCAGCACATCTAGGTTTTG 187
hCoV-19/Spain/GA-CHUAC-13215/2025|EPI_ISL_19894939|2025-05-29             CTGCAGGCTGCTTACGGTTTCGTCCGTGTTGCAGCCGATCATCAGCACATCTAGGTTTTG 187
hCoV-19/Finland/THL-02398/2025|EPI_ISL_19875939|2025-05-04                CTGCAGGCTGCTTACGGTTTCGTCCGTGTTGCAGCCGATCATCAGCACATCTAGGTTTTG 188
hCoV-19/Finland/THL-02400/2025|EPI_ISL_19875941|2025-05-03                CTGCAGGCTGCTTACGGTTTCGTCCGTGTTGCAGCCGATCATCAGCACATCTAGGTTTTG 188
```

```
hCoV-19/Ireland/LK-UHL-702929/2025|EPI_ISL_19877895|2025-04-16          CTGCAGGCTGCTTACGGTTTCGTCCGTGTTGCAGCCGATCATCAGCACATCTAGGTTTTG 188
hCoV-19/Ireland/LK-UHL-703788/2025|EPI_ISL_19877905|2025-05-03          CTGCAGGCTGCTTACGGTTTCGTCCGTGTTGCAGCCGATCATCAGCACATCTAGGTTTTG 188
hCoV-19/Ireland/LK-UHL-703851/2025|EPI_ISL_19877907|2025-05-04          CTGCAGGCTGCTTACGGTTTCGTCCGTGTTGCAGCCGATCATCAGCACATCTAGGTTTTG 188
hCoV-19/Ireland/LK-UHL-703889/2025|EPI_ISL_19877908|2025-05-05          CTGCAGGCTGCTTACGGTTTCGTCCGTGTTGCAGCCGATCATCAGCACATCTAGGTTTTG 188
hCoV-19/Ireland/D-SVUH-FBB21492-49/2025|EPI_ISL_19881756|2025-05-14     CTGCAGGCTGCTTACGGTTTCGTCCGTGTTGCAGCCGATCATCAGCACATCTAGGTTTTG 188
hCoV-19/Ireland/D-SVUH-FBB21492-59/2025|EPI_ISL_19881763|2025-05-07     CTGCAGGCTGCTTACGGTTTCGTCCGTGTTGCAGCCGATCATCAGCACATCTAGGTTTTG 188
hCoV-19/Spain/CT-HUGTiP-G3R212/2025|EPI_ISL_19883055|2025-05-20         CTGCAGGCTGCTTACGGTTTCGTCCGTGTTGCAGCCGATCATCAGCACATCTAGGTTTTG 188
hCoV-19/Ireland/LK-UHL-704398/2025|EPI_ISL_19888621|2025-05-16          CTGCAGGCTGCTTACGGTTTCGTCCGTGTTGCAGCCGATCATCAGCACATCTAGGTTTTG 188
hCoV-19/Ireland/G-GUH-N3466/2025|EPI_ISL_19893175|2025-05-03            CTGCAGGCTGCTTACGGTTTCGTCCGTGTTGCAGCCGATCATCAGCACATCTAGGTTTTG 188
hCoV-19/Belgium/Sciensano-LS-S5276/2025|EPI_ISL_19893834|2025-04-22     CTGCAGGCTGCTTACGGTTTCGTCCGTGTTGCAGCCGATCATCAGCACATCTAGGTTTTG 188
hCoV-19/Italy/VEN-ULSS8-0019244_VI/2025|EPI_ISL_19899362|2025-05-26     CTGCAGGCTGCTTACGGTTTCGTCCGTGTTGCAGCCGATCATCAGCACATCTAGGTTTTG 188
hCoV-19/Spain/CT-HUVH-E618109/2025|EPI_ISL_19907773|2025-06-10          CTGCAGGCTGCTTACGGTTTCGTCCGTGTTGCAGCCGATCATCAGCACATCTAGGTTTTG 188
hCoV-19/Ireland/D-BH-03819149/2025|EPI_ISL_19911343|2025-05-29          CTGCAGGCTGCTTACGGTTTCGTCCGTGTTGCAGCCGATCATCAGCACATCTAGGTTTTG 188
hCoV-19/Ireland/D-BH-03993161/2025|EPI_ISL_19911355|2025-06-10          CTGCAGGCTGCTTACGGTTTCGTCCGTGTTGCAGCCGATCATCAGCACATCTAGGTTTTG 188
hCoV-19/Spain/CT-HUVH-E587040/2025|EPI_ISL_19871881|2025-04-26          CTGCAGGCTGCTTACGGTTTCGTCCGTGTTGCAGCCGATCATCAGCACATCTAGGTTTTG 187
hCoV-19/Finland/THL-02392/2025|EPI_ISL_19875934|2025-05-02              CTGCAGGCTGCTTACGGTTTCGTCCGTGTTGCAGCCGATCATCAGCACATCTAGGTTTTG 187
hCoV-19/Spain/GA-CHUAC-13181/2025|EPI_ISL_19890617|2025-05-20           CTGCAGGCTGCTTACGGTTTCGTCCGTGTTGCAGCCGATCATCAGCACATCTAGGTTTTG 187
hCoV-19/Spain/CT-HUVH-E614843/2025|EPI_ISL_19907763|2025-06-05          CTGCAGGCTGCTTACGGTTTCGTCCGTGTTGCAGCCGATCATCAGCACATCTAGGTTTTG 187
hCoV-19/Italy/UMB-LAOP-172-4-02/2025|EPI_ISL_19899474|2025-05-08        CTGCAGGCTGCTTACGGTTTCGTCCGTGTTGCAGCCGATCATCAGCACATCTAGGTTTTG 209
hCoV-19/Spain/NC-CHN-01008932/2025|EPI_ISL_19900595|2025-04-04          CTGCAGGCTGCTTACGGTTTCGTCCGTGTTGCAGCCGATCATCAGCACATCTAGGTTTTG 211
hCoV-19/Northern_Ireland/CLIMB-CM7YGDRO/2025|EPI_ISL_19877331|2025-04-14 CTGCAGGCTGCTTACGGTTTCGTCCGTGTTGCAGCCGATCATCAGCACATCTAGGTTTTG 199
hCoV-19/Scotland/CLIMB-CM7YEE96/2025|EPI_ISL_19897754|2025-05-13        CTGCAGGCTGCTTACGGTTTCGTCCGTGTTGCAGCCGATCATCAGCACATCTAGGTTTTG 198
hCoV-19/France/ARA-HCL125018289001/2025|EPI_ISL_19880005|2025-05-12     CTGCAGGCTGCTTACGGTTTCGTCCGTGTTGCAGCCGATCATCAGCACATCTAGGTTTTG 192
hCoV-19/France/ARA-RELAB-HCL725000057501/2025|EPI_ISL_19880014|2025-04-28 CTGCAGGCTGCTTACGGTTTCGTCCGTGTTGCAGCCGATCATCAGCACATCTAGGTTTTG 192
hCoV-19/France/ARA-RELAB-HCL725000059501/2025|EPI_ISL_19885160|2025-04-25 CTGCAGGCTGCTTACGGTTTCGTCCGTGTTGCAGCCGATCATCAGCACATCTAGGTTTTG 192
hCoV-19/France/NAQ-RELAB-HCL725000061001/2025|EPI_ISL_19885161|2025-05-10 CTGCAGGCTGCTTACGGTTTCGTCCGTGTTGCAGCCGATCATCAGCACATCTAGGTTTTG 192
hCoV-19/France/NAQ-RELAB-HCL725000060901/2025|EPI_ISL_19885180|2025-05-09 CTGCAGGCTGCTTACGGTTTCGTCCGTGTTGCAGCCGATCATCAGCACATCTAGGTTTTG 192
hCoV-19/France/ARA-HCL025075546601/2025|EPI_ISL_19885182|2025-05-09     CTGCAGGCTGCTTACGGTTTCGTCCGTGTTGCAGCCGATCATCAGCACATCTAGGTTTTG 192
hCoV-19/France/ARA-HCL125018461901/2025|EPI_ISL_19885204|2025-05-14     CTGCAGGCTGCTTACGGTTTCGTCCGTGTTGCAGCCGATCATCAGCACATCTAGGTTTTG 192
hCoV-19/France/ARA-HCL125018655901/2025|EPI_ISL_19885206|2025-05-14     CTGCAGGCTGCTTACGGTTTCGTCCGTGTTGCAGCCGATCATCAGCACATCTAGGTTTTG 192
hCoV-19/France/ARA-HCL125019155501/2025|EPI_ISL_19885209|2025-05-18     CTGCAGGCTGCTTACGGTTTCGTCCGTGTTGCAGCCGATCATCAGCACATCTAGGTTTTG 192
hCoV-19/France/ARA-HCL125019240901/2025|EPI_ISL_19885210|2025-05-19     CTGCAGGCTGCTTACGGTTTCGTCCGTGTTGCAGCCGATCATCAGCACATCTAGGTTTTG 192
hCoV-19/France/ARA-HCL125019249201/2025|EPI_ISL_19885211|2025-05-19     CTGCAGGCTGCTTACGGTTTCGTCCGTGTTGCAGCCGATCATCAGCACATCTAGGTTTTG 192
hCoV-19/France/ARA-HCL125019399701/2025|EPI_ISL_19885212|2025-05-20     CTGCAGGCTGCTTACGGTTTCGTCCGTGTTGCAGCCGATCATCAGCACATCTAGGTTTTG 192
hCoV-19/France/ARA-HCL125019408901/2025|EPI_ISL_19885213|2025-05-20     CTGCAGGCTGCTTACGGTTTCGTCCGTGTTGCAGCCGATCATCAGCACATCTAGGTTTTG 192
hCoV-19/France/OCC-RELAB-HCL725000064001/2025|EPI_ISL_19895004|2025-05-14 CTGCAGGCTGCTTACGGTTTCGTCCGTGTTGCAGCCGATCATCAGCACATCTAGGTTTTG 192
hCoV-19/France/OCC-RELAB-HCL725000064101/2025|EPI_ISL_19895005|2025-05-14 CTGCAGGCTGCTTACGGTTTCGTCCGTGTTGCAGCCGATCATCAGCACATCTAGGTTTTG 192
hCoV-19/France/NAQ-RELAB-HCL725000062601/2025|EPI_ISL_19895009|2025-05-16 CTGCAGGCTGCTTACGGTTTCGTCCGTGTTGCAGCCGATCATCAGCACATCTAGGTTTTG 192
hCoV-19/France/ARA-RELAB-HCL725000062401/2025|EPI_ISL_19895017|2025-05-16 CTGCAGGCTGCTTACGGTTTCGTCCGTGTTGCAGCCGATCATCAGCACATCTAGGTTTTG 192
hCoV-19/France/ARA-RELAB-HCL725000062101/2025|EPI_ISL_19895018|2025-05-13 CTGCAGGCTGCTTACGGTTTCGTCCGTGTTGCAGCCGATCATCAGCACATCTAGGTTTTG 192
hCoV-19/France/ARA-HCL125019625101/2025|EPI_ISL_19895034|2025-05-21     CTGCAGGCTGCTTACGGTTTCGTCCGTGTTGCAGCCGATCATCAGCACATCTAGGTTTTG 192
hCoV-19/France/ARA-HCL125019754801/2025|EPI_ISL_19895035|2025-05-22     CTGCAGGCTGCTTACGGTTTCGTCCGTGTTGCAGCCGATCATCAGCACATCTAGGTTTTG 192
hCoV-19/France/ARA-RELAB-HCL725000064601/2025|EPI_ISL_19895041|2025-05-15 CTGCAGGCTGCTTACGGTTTCGTCCGTGTTGCAGCCGATCATCAGCACATCTAGGTTTTG 192
hCoV-19/France/ARA-RELAB-HCL725000066001/2025|EPI_ISL_19895046|2025-05-13 CTGCAGGCTGCTTACGGTTTCGTCCGTGTTGCAGCCGATCATCAGCACATCTAGGTTTTG 192
hCoV-19/France/OCC-RELAB-HCL725000068701/2025|EPI_ISL_19905466|2025-05-21 CTGCAGGCTGCTTACGGTTTCGTCCGTGTTGCAGCCGATCATCAGCACATCTAGGTTTTG 192
hCoV-19/France/OCC-RELAB-HCL725000069001/2025|EPI_ISL_19905468|2025-05-21 CTGCAGGCTGCTTACGGTTTCGTCCGTGTTGCAGCCGATCATCAGCACATCTAGGTTTTG 192
hCoV-19/France/ARA-HCL025089279801/2025|EPI_ISL_19905469|2025-06-02     CTGCAGGCTGCTTACGGTTTCGTCCGTGTTGCAGCCGATCATCAGCACATCTAGGTTTTG 192
hCoV-19/France/PAC-RELAB-HCL725000069901/2025|EPI_ISL_19908847|2025-05-26 CTGCAGGCTGCTTACGGTTTCGTCCGTGTTGCAGCCGATCATCAGCACATCTAGGTTTTG 192
hCoV-19/France/PAC-RELAB-HCL725000069501/2025|EPI_ISL_19908849|2025-05-30 CTGCAGGCTGCTTACGGTTTCGTCCGTGTTGCAGCCGATCATCAGCACATCTAGGTTTTG 192
hCoV-19/France/ARA-RELAB-HCL725000071701/2025|EPI_ISL_19908858|2025-05-22 CTGCAGGCTGCTTACGGTTTCGTCCGTGTTGCAGCCGATCATCAGCACATCTAGGTTTTG 192
hCoV-19/France/ARA-HCL025093352901/2025|EPI_ISL_19908867|2025-06-10     CTGCAGGCTGCTTACGGTTTCGTCCGTGTTGCAGCCGATCATCAGCACATCTAGGTTTTG 192
hCoV-19/France/ARA-RELAB-HCL725000065801/2025|EPI_ISL_19895045|2025-05-16 CTGCAGGCTGCTTACGGTTTCGTCCGTGTTGCAGCCGATTATCAGCACATCTAGGTTTTG 192
hCoV-19/Denmark/DCGC-692234/2025|EPI_ISL_19879694|2025-05-05            CTGCAGGCTGCTTACGGTTTCGTCCGTGTTGCAGCCGATCATCAGCACATCTAGGTTTTG 194
```

```
hCoV-19/Finland/THL-02388/2025|EPI_ISL_19875931|2025-04-16                 CTGCAGGCTGCTTACGGTTTCGTCCGTGTTGCAGCCGATCATCAGCACATCTAGGTTTTG 195
hCoV-19/Spain/PV-HUD-98024244/2025|EPI_ISL_19907759|2025-06-09             CTGCAGGCTGCTTACGGTTTCGTCCGTGTTGCAGCCGATCATCAGCACATCTAGGTTTTG 195
hCoV-19/Netherlands/GE-RIVM-145386/2025|EPI_ISL_19873764|2025-05-01        CTGCAGGCTGCTTACGGTTTCGTCCGTGTTGCAGCCGATCATCAGCACATCTAGGTTTTG 192
hCoV-19/Netherlands/ZH-RIVM-145413/2025|EPI_ISL_19891397|2025-04-30        CTGCAGGCTGCTTACGGTTTCGTCCGTGTTGCAGCCGATCATCAGCACATCTAGGTTTTG 192
hCoV-19/Netherlands/NH-RIVM-145458/2025|EPI_ISL_19905062|2025-05-25        CTGCAGGCTGCTTACGGTTTCGTCCGTGTTGCAGCCGATCATCAGCACATCTAGGTTTTG 192
hCoV-19/Netherlands/NH-RIVM-145463/2025|EPI_ISL_19905063|2025-05-20        CTGCAGGCTGCTTACGGTTTCGTCCGTGTTGCAGCCGATCATCAGCACATCTAGGTTTTG 192
hCoV-19/Netherlands/UT-RIVM-145436/2025|EPI_ISL_19905072|2025-05-15        CTGCAGGCTGCTTACGGTTTCGTCCGTGTTGCAGCCGATCATCAGCACATCTAGGTTTTG 192
hCoV-19/Netherlands/UT-RIVM-145470/2025|EPI_ISL_19905077|2025-06-02        CTGCAGGCTGCTTACGGTTTCGTCCGTGTTGCAGCCGATCATCAGCACATCTAGGTTTTG 192
hCoV-19/Netherlands/ZH-RIVM-145478/2025|EPI_ISL_19905094|2025-05-30        CTGCAGGCTGCTTACGGTTTCGTCCGTGTTGCAGCCGATCATCAGCACATCTAGGTTTTG 192
hCoV-19/Netherlands/ZH-RIVM-145480/2025|EPI_ISL_19905096|2025-05-31        CTGCAGGCTGCTTACGGTTTCGTCCGTGTTGCAGCCGATCATCAGCACATCTAGGTTTTG 192
hCoV-19/Netherlands/UT-RIVM-145481/2025|EPI_ISL_19905078|2025-05-31        CTGCAGGCTGCTTACGGTTTCGTCCGTGTTGCAGCCGATCATCAGCACATCTAGGTTTTG 192
hCoV-19/Scotland/CLIMB-CM7YRW9H/2025|EPI_ISL_19881711|2025-05-06           CTGCAGGCTGCTTACGGTTTCGTCCGTGTTGCAGCCGATCATCAGCACATCTAGGTTTTG 164
hCoV-19/France/IDF-RELAB-IPP04872/2025|EPI_ISL_19911293|2025-05-22         CTGCAGGCTGCTTACGGTTTCGTCCGTGTTGCAGCCGATCATCAGCACATCTAGGTTTTG 160
hCoV-19/Canary_Islands/CN-HUC-925112913/2025|EPI_ISL_19900668|2025-04-22   CTGCAGGCTGCTTACGGTTTCGTCCGTGTTGCAGCCGATCATCAGCACATCTAGGTTTTG 168
hCoV-19/Scotland/CLIMB-CM7YJ5BB/2025|EPI_ISL_19906458|2025-05-27           CTGCAGGCTGCTTACGGTTTCGTCCGTGTTGCAGCCGATCATCAGCACATCTAGGTTTTG 164
hCoV-19/Scotland/CLIMB-CM7Y8PHH/2025|EPI_ISL_19897755|2025-05-14           CTGCAGGCTGCCTACGGTTTCGTCCGTGTTGCAGCCGATCATCAGCACATCTAGGTTTTG 164
hCoV-19/Spain/AN-PMC-42706/2025|EPI_ISL_19888325|2025-05-25                CTGCAGGCTGCTTACGGTTTCGTCCGTGTTGCAGCCGATCATCAGCACATCTAGGTTTTG 164
hCoV-19/Scotland/CLIMB-CM7YRIA1/2025|EPI_ISL_19869961|2025-04-20           CTGCAGGCTGCTTACGGTTTCGTCCGTGTTGCAGCCGATCATCAGCACATCTAGGTTTTG 164
hCoV-19/Scotland/CLIMB-CM7Y8UWT/2025|EPI_ISL_19869966|2025-04-21           CTGCAGGCTGCTTACGGTTTCGTCCGTGTTGCAGCCGATCATCAGCACATCTAGGTTTTG 164
hCoV-19/England/CLIMB-CM7YMFHB/2025|EPI_ISL_19877436|2025-05-10            CTGCAGGCTGCTTACGGTTTCGTCCGTGTTGCAGCCGATCATCAGCACATCTAGGTTTTG 164
hCoV-19/Denmark/DCGC-692213/2025|EPI_ISL_19879673|2025-04-28               CTGCAGGCTGCTTACGGTTTCGTCCGTGTTGCAGCCGATCATCAGCACATCTAGGTTTTG 164
hCoV-19/Wales/CLIMB-CM7YRAXQ/2025|EPI_ISL_19883075|2025-05-14              CTGCAGGCTGCTTACGGTTTCGTCCGTGTTGCAGCCGATCATCAGCACATCTAGGTTTTG 164
hCoV-19/Spain/AN-PMC-42696/2025|EPI_ISL_19888316|2025-05-14                CTGCAGGCTGCTTACGGTTTCGTCCGTGTTGCAGCCGATCATCAGCACATCTAGGTTTTG 164
hCoV-19/Spain/AN-PMC-42719/2025|EPI_ISL_19888338|2025-05-15                CTGCAGGCTGCTTACGGTTTCGTCCGTGTTGCAGCCGATCATCAGCACATCTAGGTTTTG 164
hCoV-19/England/CLIMB-CM7YE7HB/2025|EPI_ISL_19892115|2025-05-19            CTGCAGGCTGCTTACGGTTTCGTCCGTGTTGCAGCCGATCATCAGCACATCTAGGTTTTG 164
hCoV-19/France/GES-RELAB-IPP04599/2025|EPI_ISL_19893547|2025-05-05         CTGCAGGCTGCTTACGGTTTCGTCCGTGTTGCAGCCGATCATCAGCACATCTAGGTTTTG 164
hCoV-19/Scotland/CLIMB-CM7YEPXZ/2025|EPI_ISL_19896028|2025-05-25           CTGCAGGCTGCTTACGGTTTCGTCCGTGTTGCAGCCGATCATCAGCACATCTAGGTTTTG 164
hCoV-19/Scotland/CLIMB-CM7YF4QT/2025|EPI_ISL_19896032|2025-05-27           CTGCAGGCTGCTTACGGTTTCGTCCGTGTTGCAGCCGATCATCAGCACATCTAGGTTTTG 164
hCoV-19/Scotland/CLIMB-CM7YKDZ6/2025|EPI_ISL_19896033|2025-05-27           CTGCAGGCTGCTTACGGTTTCGTCCGTGTTGCAGCCGATCATCAGCACATCTAGGTTTTG 164
hCoV-19/Scotland/CLIMB-CM7YG8H4/2025|EPI_ISL_19897748|2025-05-02           CTGCAGGCTGCTTACGGTTTCGTCCGTGTTGCAGCCGATCATCAGCACATCTAGGTTTTG 164
hCoV-19/Wales/CLIMB-CM7YJXNZ/2025|EPI_ISL_19900036|2025-05-26              CTGCAGGCTGCTTACGGTTTCGTCCGTGTTGCAGCCGATCATCAGCACATCTAGGTTTTG 164
hCoV-19/Germany/NW-RKI-I-1148178/2025|EPI_ISL_19902055|2025-05-21          CTGCAGGCTGCTTACGGTTTCGTCCGTGTTGCAGCCGATCATCAGCACATCTAGGTTTTG 164
hCoV-19/Germany/NI-RKI-I-1148183/2025|EPI_ISL_19902060|2025-05-18          CTGCAGGCTGCTTACGGTTTCGTCCGTGTTGCAGCCGATCATCAGCACATCTAGGTTTTG 164
hCoV-19/Germany/NW-RKI-I-1148191/2025|EPI_ISL_19902068|2025-05-19          CTGCAGGCTGCTTACGGTTTCGTCCGTGTTGCAGCCGATCATCAGCACATCTAGGTTTTG 164
hCoV-19/Spain/MD-HGUGM-66414184/2025|EPI_ISL_19906298|2025-05-25           CTGCAGGCTGCTTACGGTTTCGTCCGTGTTGCAGCCGATCATCAGCACATCTAGGTTTTG 164
hCoV-19/Spain/MD-HGUGM-66400167/2025|EPI_ISL_19906320|2025-05-22           CTGCAGGCTGCTTACGGTTTCGTCCGTGTTGCAGCCGATCATCAGCACATCTAGGTTTTG 164
hCoV-19/France/IDF-RELAB-IPP04646/2025|EPI_ISL_19907194|2025-05-16         CTGCAGGCTGCTTACGGTTTCGTCCGTGTTGCAGCCGATCATCAGCACATCTAGGTTTTG 164
hCoV-19/England/CLIMB-CM7YKIUQ/2025|EPI_ISL_19908573|2025-06-07            CTGCAGGCTGCTTACGGTTTCGTCCGTGTTGCAGCCGATCATCAGCACATCTAGGTTTTG 164
hCoV-19/France/GES-RELAB-IPP04823/2025|EPI_ISL_19911257|2025-05-26         CTGCAGGCTGCTTACGGTTTCGTCCGTGTTGCAGCCGATCATCAGCACATCTAGGTTTTG 164
hCoV-19/France/IDF-RELAB-IPP04863/2025|EPI_ISL_19911265|2025-05-24         CTGCAGGCTGCTTACGGTTTCGTCCGTGTTGCAGCCGATCATCAGCACATCTAGGTTTTG 164
hCoV-19/Denmark/DCGC-692245/2025|EPI_ISL_19879704|2025-04-28               CTGCAGGCTGCTTACGGTTTCGTCCGTGTTGCAGCCGATCATCAGCACATCTAGGTTTTG 164


hCoV-19/Ireland/D-SVUH-FBA98650-31/2025|EPI_ISL_19866548|2025-05-04        ------------------------------------------------------------ 0
hCoV-19/Spain/PV-HUB-5628663/2025|EPI_ISL_19908839|2025-05-16              TCCGGGTGTGACCGAAAGGTAAGATGGAGAGCCTTGTCCCTGGTTTCAACGAGAAAACAC 97
hCoV-19/Spain/PV-HUB-41587498/2025|EPI_ISL_19908930|2025-05-20             TCCGGGTGTGACCGAAAGGTAAGATGGAGAGCCTTGTCCCTGGTTTCAACGAGAAAACAC 92
hCoV-19/Spain/PV-HUB-56291851/2025|EPI_ISL_19908970|2025-05-31             TCCGGGTGTGACCGAAAGGTAAGATGGAGAGCCTTGTCCCTGGTTTCAACGAGAAAACAC 93
hCoV-19/Northern_Ireland/CLIMB-CM7YR8I6/2025|EPI_ISL_19877334|2025-04-23   TCCGGGTGTGACCGAAAGGTAAGATGGAGAGCCTTGTCCCTGGTTTCAACGAGAAAACAC 201
hCoV-19/Germany/NW-RKI-I-1148136/2025|EPI_ISL_19871950|2025-04-23          TCCGGGTGTGACCGAAAGGTAAGATGGAGAGCCTTGTCCCTGGTTTCAACGAGAAAACAC 224
hCoV-19/Germany/NW-RKI-I-1148141/2025|EPI_ISL_19871955|2025-04-23          TCCGGGTGTGACCGAAAGGTAAGATGGAGAGCCTTGTCCCTGGTTTCAACGAGAAAACAC 224
hCoV-19/Germany/BW-RKI-I-1148164/2025|EPI_ISL_19884987|2025-04-17          TCCGGGTGTGACCGAAAGGTAAGATGGAGAGCCTTGTCCCTGGTTTCAACGAGAAAACAC 224
hCoV-19/Scotland/CLIMB-CM7YFGW7/2025|EPI_ISL_19897691|2025-05-24           TCCGGGTGTGACCGAAAGGTAAGATGGAGAGCCTTGTCCCTGGTTTCAACGAGAAAACAC 224
hCoV-19/Ireland/G-LUH-N3473/2025|EPI_ISL_19893181|2025-05-14               TCCGGGTGTGACCGAAAGGTAAGATGGAGAGCCTTGTCCCTGGTTTCAACGAGAAAACAC 291
```

hCoV-19/Spain/AN-PMC-42728/2025|EPI_ISL_19888347|2025-05-25	TCCGGGTGTGACCGAAAGGTAAGATGGAGAGCCTTGTCCCTGGTTTCAACGAGAAAACAC	292
hCoV-19/Sweden/00200866492N2/2025|EPI_ISL_19870050|2025-04-22	TCCGGGTGTGACCGAAAGGTAAGATGGAGAGCCTTGTCCCTGGTTTCAACGAGAAAACAC	298
hCoV-19/Sweden/00200867159N2/2025|EPI_ISL_19870052|2025-04-23	TCCGGGTGTGACCGAAAGGTAAGATGGAGAGCCTTGTCCCTGGTTTCAACGAGAAAACAC	298
hCoV-19/Ireland/G-LUH-N3445/2025|EPI_ISL_19893154|2025-05-07	TCCGGGTGTGACCGAAAGGTAAGATGGAGAGCCTTGTCCCTGGTTTCAACGAGAAAACAC	298
hCoV-19/Ireland/G-LUH-N3443/2025|EPI_ISL_19893152|2025-05-07	TCCGGGTGTGACCGAAAGGTAAGATGGAGAGCCTTGTCCCTGGTTTCAACGAGAAAACAC	296
hCoV-19/Scotland/CLIMB-CM7YF9PJ/2025|EPI_ISL_19895983|2025-05-19	TCCGGGTGTGACCGAAAGGTAAGATGGAGAGCCTTGTCCCTGGTTTCAACGAGAAAACAC	295
hCoV-19/Scotland/CLIMB-CM7YKMK8/2025|EPI_ISL_19869976|2025-04-25	TCCGGGTGTGACCGAAAGGTAAGATGGAGAGCCTTGTCCCTGGTTTCAACGAGAAAACAC	294
hCoV-19/Ireland/G-LUH-N3444/2025|EPI_ISL_19893153|2025-05-07	TCCGGGTGTGACCGAAAGGTAAGATGGAGAGCCTTGTCCCTGGTTTCAACGAGAAAACAC	294
hCoV-19/Switzerland/VD-CHUV-8604552682/2025|EPI_ISL_19906527|2025-05-19	TCCGGGTGTGACCGAAAGGTAAGATGGAGAGCCTTGTCCCTGGTTTCAACGAGAAAACAC	299
hCoV-19/Scotland/CLIMB-CM7YR3YO/2025|EPI_ISL_19896015|2025-05-24	TCCGGGTGTGACCGAAAGGTAAGATGGAGAGCCTTGTCCCTGGTTTCAACGAGAAAACAC	300
hCoV-19/Sweden/O-18_SE100_25CS500180/2025|EPI_ISL_19866288|2025-04-15	TCCGGGTGTGACCGAAAGGTAAGATGGAGAGCCTTGTCCCTGGTTTCAACGAGAAAACAC	300
hCoV-19/Ireland/D-CS250088/2025|EPI_ISL_19877457|2025-04-16	TCCGGGTGTGACCGAAAGGTAAGATGGAGAGCCTTGTCCCTGGTTTCAACGAGAAAACAC	300
hCoV-19/Ireland/G-LUH-N3442/2025|EPI_ISL_19893151|2025-05-07	TCCGGGTGTGACCGAAAGGTAAGATGGAGAGCCTTGTCCCTGGTTTCAACGAGAAAACAC	300
hCoV-19/Scotland/CLIMB-CM7YMZ5M/2025|EPI_ISL_19906439|2025-05-22	TCCGGGTGTGACCGAAAGGTAAGATGGAGAGCCTTGTCCCTGGTTTCAACGAGAAAACAC	299
hCoV-19/Scotland/CLIMB-CM7YR3ZG/2025|EPI_ISL_19897722|2025-05-13	TCCGGGTGTGACCGAAAGGTAAGATGGAGAGCCTTGTCCCTGGTTTCAACGAGAAAACAC	298
hCoV-19/Scotland/CLIMB-CM7YEYGT/2025|EPI_ISL_19897741|2025-04-28	TCCGGGTGTGACCGAAAGGTAAGATGGAGAGCCTTGTCCCTGGTTTCAACGAGAAAACAC	298
hCoV-19/Scotland/CLIMB-CM7YGAJ3/2025|EPI_ISL_19895963|2025-05-13	TCCGGGTGTGACCGAAAGGTAAGATGGAGAGCCTTGTCCCTGGTTTCAACGAGAAAACAC	287
hCoV-19/Scotland/CLIMB-CM7YJ1ZO/2025|EPI_ISL_19897757|2025-05-14	TCCGGGTGTGACCGAAAGGTAAGATGGAGAGCCTTGTCCCTGGTTTCAACGAGAAAACAC	288
hCoV-19/Scotland/CLIMB-CM7Y8C4Y/2025|EPI_ISL_19906404|2025-05-15	TCCGGGTGTGACCGAAAGGTAAGATGGAGAGCCTTGTCCCTGGTTTCAACGAGAAAACAC	295
hCoV-19/Northern_Ireland/CLIMB-CM7YGGYM/2025|EPI_ISL_19897641|2025-05-23	TCCGGGTGTGACCGAAAGGTAAGATGGAGAGCCTTGTCCCTGGTTTCAACGAGAAAACAC	290
hCoV-19/Scotland/CLIMB-CM7YFW7M/2025|EPI_ISL_19881718|2025-05-10	TCCGGGTGTGACCGAAAGGTAAGATGGAGAGCCTTGTCCCTGGTTTCAACGAGAAAACAC	293
hCoV-19/Scotland/CLIMB-CM7YEMXP/2025|EPI_ISL_19888387|2025-05-10	TCCGGGTGTGACCGAAAGGTAAGATGGAGAGCCTTGTCCCTGGTTTCAACGAGAAAACAC	292
hCoV-19/Spain/CT-HUVH-E593511/2025|EPI_ISL_19881731|2025-05-06	TCCGGGTGTGACCGAAAGGTAAGATGGAGAGCCTTGTCCCTGGTTTCAACGAGAAAACAC	245
hCoV-19/Spain/CT-HUJT-E325362/2025|EPI_ISL_19889935|2025-05-20	TCCGGGTGTGACCGAAAGGTAAGATGGAGAGCCTTGTCCCTGGTTTCAACGAGAAAACAC	246
hCoV-19/Spain/PV-HUD-84743204/2025|EPI_ISL_19907746|2025-06-06	TCCGGGTGTGACCGAAAGGTAAGATGGAGAGCCTTGTCCCTGGTTTCAACGAGAAAACAC	247
hCoV-19/Ireland/D-SVUH-FBB21492-54/2025|EPI_ISL_19881758|2025-05-11	TCCGGGTGTGACCGAAAGGTAAGATGGAGAGCCTTGTCCCTGGTTTCAACGAGAAAACAC	248
hCoV-19/Spain/CT-HUVH-E596725/2025|EPI_ISL_19881729|2025-05-10	TCCGGGTGTGACCGAAAGGTAAGATGGAGAGCCTTGTCCCTGGTTTCAACGAGAAAACAC	242
hCoV-19/Spain/CT-HUVH-E593882/2025|EPI_ISL_19881732|2025-05-06	TCCGGGTGTGACCGAAAGGTAAGATGGAGAGCCTTGTCCCTGGTTTCAACGAGAAAACAC	242
hCoV-19/Finland/THL-02385/2025|EPI_ISL_19875928|2025-04-16	TCCGGGTGTGACCGAAAGGTAAGATGGAGAGCCTTGTCCCTGGTTTCAACGAGAAAATAC	249
hCoV-19/Ireland/D-BH-02351167/2025|EPI_ISL_19911337|2025-05-28	TCCGGGTGTGACCGAAAGGTAAGATGGAGAGCCTTGTCCCTGGTTTCAACGAGAAAACAC	248
hCoV-19/Ireland/D-NVRL-XIRL00001782/2025|EPI_ISL_19896104|2025-05-14	TCCGGGTGTGACCGAAAGGTAAGATGGAGAGCCTTGTCCCTGGTTTCAACGAGAAAACAC	248
hCoV-19/Ireland/TA-NVRL-GIRL00039444/2025|EPI_ISL_19896118|2025-05-16	TCCGGGTGTGACCGAAAGGTAAGATGGAGAGCCTTGTCCCTGGTTTCAACGAGAAAACAC	248
hCoV-19/Spain/GA-CHUAC-13223/2025|EPI_ISL_19894946|2025-05-31	TCCGGGTGTGACCGAAAGGTAAGATGGAGAGCCTTGTCCCTGGTTTCAACGAGAAAACAC	247
hCoV-19/Spain/GA-CHUAC-13215/2025|EPI_ISL_19894939|2025-05-29	TCCGGGTGTGACCGAAAGGTAAGATGGAGAGCCTTGTCCCTGGTTTCAACGAGAAAACAC	247
hCoV-19/Finland/THL-02398/2025|EPI_ISL_19875939|2025-05-04	TCCGGGTGTGACCGAAAGGTAAGATGGAGAGCCTTGTCCCTGGTTTCAACGAGAAAACAC	248
hCoV-19/Finland/THL-02400/2025|EPI_ISL_19875941|2025-05-03	TCCGGGTGTGACCGAAAGGTAAGATGGAGAGCCTTGTCCCTGGTTTCAACGAGAAAACAC	248
hCoV-19/Ireland/LK-UHL-702929/2025|EPI_ISL_19877895|2025-04-16	TCCGGGTGTGACCGAAAGGTAAGATGGAGAGCCTTGTCCCTGGTTTCAACGAGAAAACAC	248
hCoV-19/Ireland/LK-UHL-703788/2025|EPI_ISL_19877905|2025-05-03	TCCGGGTGTGACCGAAAGGTAAGATGGAGAGCCTTGTCCCTGGTTTCAACGAGAAAACAC	248
hCoV-19/Ireland/LK-UHL-703851/2025|EPI_ISL_19877907|2025-05-04	TCCGGGTGTGACCGAAAGGTAAGATGGAGAGCCTTGTCCCTGGTTTCAACGAGAAAACAC	248
hCoV-19/Ireland/LK-UHL-703889/2025|EPI_ISL_19877908|2025-05-05	TCCGGGTGTGACCGAAAGGTAAGATGGAGAGCCTTGTCCCTGGTTTCAACGAGAAAACAC	248
hCoV-19/Ireland/D-SVUH-FBB21492-49/2025|EPI_ISL_19881756|2025-05-14	TCCGGGTGTGACCGAAAGGTAAGATGGAGAGCCTTGTCCCTGGTTTCAACGAGAAAACAC	248
hCoV-19/Ireland/D-SVUH-FBB21492-59/2025|EPI_ISL_19881763|2025-05-07	TCCGGGTGTGACCGAAAGGTAAGATGGAGAGCCTTGTCCCTGGTTTCAACGAGAAAACAC	248
hCoV-19/Spain/CT-HUGTiP-G3R212/2025|EPI_ISL_19883055|2025-05-20	TCCGGGTGTGACCGAAAGGTAAGATGGAGAGCCTTGTCCCTGGTTTCAACGAGAAAACAC	248
hCoV-19/Ireland/LK-UHL-704398/2025|EPI_ISL_19888621|2025-05-16	TCCGGGTGTGACCGAAAGGTAAGATGGAGAGCCTTGTCCCTGGTTTCAACGAGAAAACAC	248
hCoV-19/Ireland/G-GUH-N3466/2025|EPI_ISL_19893175|2025-05-03	TCCGGGTGTGACCGAAAGGTAAGATGGAGAGCCTTGTCCCTGGTTTCAACGAGAAAACAC	248
hCoV-19/Belgium/Sciensano-LS-S5276/2025|EPI_ISL_19893834|2025-04-22	TCCGGGTGTGACCGAAAGGTAAGATGGAGAGCCTTGTCCCTGGTTTCAACGAGAAAACAC	248
hCoV-19/Italy/VEN-ULSS8-0019244_VI/2025|EPI_ISL_19899362|2025-05-26	TCCGGGTGTGACCGAAAGGTAAGATGGAGAGCCTTGTCCCTGGTTTCAACGAGAAAACAC	248
hCoV-19/Spain/CT-HUVH-E618109/2025|EPI_ISL_19907773|2025-06-10	TCCGGGTGTGACCGAAAGGTAAGATGGAGAGCCTTGTCCCTGGTTTCAACGAGAAAACAC	248
hCoV-19/Ireland/D-BH-03819149/2025|EPI_ISL_19911343|2025-05-29	TCCGGGTGTGACCGAAAGGTAAGATGGAGAGCCTTGTCCCTGGTTTCAACGAGAAAACAC	248
hCoV-19/Ireland/D-BH-03993161/2025|EPI_ISL_19911355|2025-06-10	TCCGGGTGTGACCGAAAGGTAAGATGGAGAGCCTTGTCCCTGGTTTCAACGAGAAAACAC	248
hCoV-19/Spain/CT-HUVH-E587040/2025|EPI_ISL_19871881|2025-04-26	TCCGGGTGTGACCGAAAGGTAAGATGGAGAGCCTTGTCCCTGGTTTCAACGAGAAAACAC	247
hCoV-19/Finland/THL-02392/2025|EPI_ISL_19875934|2025-05-02	TCCGGGTGTGACCGAAAGGTAAGATGGAGAGCCTTGTCCCTGGTTTCAACGAGAAAACAC	247
hCoV-19/Spain/GA-CHUAC-13181/2025|EPI_ISL_19890617|2025-05-20	TCCGGGTGTGACCGAAAGGTAAGATGGAGAGCCTTGTCCCTGGTTTCAACGAGAAAACAC	247

hCoV-19/Spain/CT-HUVH-E614843/2025|EPI_ISL_19907763|2025-06-05	TCCGGGTGTGACCGAAAGGTAAGATGGAGAGCCTTGTCCCTGGTTTCAACGAGAAAACAC	247
hCoV-19/Italy/UMB-LAOP-172-4-02/2025|EPI_ISL_19899474|2025-05-08	TCCGGGTGTGACCGAAAGGTAAGATGGAGAGCCTTGTCCCTGGTTTCAACGAGAAAACAC	269
hCoV-19/Spain/NC-CHN-01008932/2025|EPI_ISL_19900595|2025-04-04	TCCGGGTGTGACCGAAAGGTAAGATGGAGAGCCTTGTCCCTGGTTTCAACGAGAAAACAC	271
hCoV-19/Northern_Ireland/CLIMB-CM7YGDRO/2025|EPI_ISL_19877331|2025-04-14	TCCGGGTGTGACCGAAAGGTAAGATGGAGAGCCTTGTCCCTGGTTTCAACGAGAAAACAC	259
hCoV-19/Scotland/CLIMB-CM7YEE96/2025|EPI_ISL_19897754|2025-05-13	TCCGGGTGTGACCGAAAGGTAAGATGGAGAGCCTTGTCCCTGGTTTCAACGAGAAAACAC	258
hCoV-19/France/ARA-HCL125018289001/2025|EPI_ISL_19880005|2025-05-12	TCCGGGTGTGACCGAAAGGTAAGATGGAGAGCCTTGTCCCTGGTTTCAACGAGAAAACAC	252
hCoV-19/France/ARA-RELAB-HCL725000057501/2025|EPI_ISL_19880014|2025-04-28	TCCGGGTGTGACCGAAAGGTAAGATGGAGAGCCTTGTCCCTGGTTTCAACGAGAAAACAC	252
hCoV-19/France/ARA-RELAB-HCL725000059501/2025|EPI_ISL_19885160|2025-04-25	TCCGGGTGTGACCGAAAGGTAAGATGGAGAGCCTTGTCCCTGGTTTCAACGAGAAAACAC	252
hCoV-19/France/NAQ-RELAB-HCL725000061001/2025|EPI_ISL_19885161|2025-05-10	TCCGGGTGTGACCGAAAGGTAAGATGGAGAGCCTTGTCCCTGGTTTCAACGAGAAAACAC	252
hCoV-19/France/NAQ-RELAB-HCL725000060901/2025|EPI_ISL_19885180|2025-05-09	TCCGGGTGTGACCGAAAGGTAAGATGGAGAGCCTTGTCCCTGGTTTCAACGAGAAAACAC	252
hCoV-19/France/ARA-HCL025075546601/2025|EPI_ISL_19885182|2025-05-09	TCCGGGTGTGACCGAAAGGTAAGATGGAGAGCCTTGTCCCTGGTTTCAACGAGAAAACAC	252
hCoV-19/France/ARA-HCL125018461901/2025|EPI_ISL_19885204|2025-05-14	TCCGGGTGTGACCGAAAGGTAAGATGGAGAGCCTTGTCCCTGGTTTCAACGAGAAAACAC	252
hCoV-19/France/ARA-HCL125018655901/2025|EPI_ISL_19885206|2025-05-14	TCCGGGTGTGACCGAAAGGTAAGATGGAGAGCCTTGTCCCTGGTTTCAACGAGAAAACAC	252
hCoV-19/France/ARA-HCL125019155501/2025|EPI_ISL_19885209|2025-05-18	TCCGGGTGTGACCGAAAGGTAAGATGGAGAGCCTTGTCCCTGGTTTCAACGAGAAAACAC	252
hCoV-19/France/ARA-HCL125019240901/2025|EPI_ISL_19885210|2025-05-19	TCCGGGTGTGACCGAAAGGTAAGATGGAGAGCCTTGTCCCTGGTTTCAACGAGAAAACAC	252
hCoV-19/France/ARA-HCL125019249201/2025|EPI_ISL_19885211|2025-05-19	TCCGGGTGTGACCGAAAGGTAAGATGGAGAGCCTTGTCCCTGGTTTCAACGAGAAAACAC	252
hCoV-19/France/ARA-HCL125019399701/2025|EPI_ISL_19885212|2025-05-20	TCCGGGTGTGACCGAAAGGTAAGATGGAGAGCCTTGTCCCTGGTTTCAACGAGAAAACAC	252
hCoV-19/France/ARA-HCL125019408901/2025|EPI_ISL_19885213|2025-05-20	TCCGGGTGTGACCGAAAGGTAAGATGGAGAGCCTTGTCCCTGGTTTCAACGAGAAAACAC	252
hCoV-19/France/OCC-RELAB-HCL725000064001/2025|EPI_ISL_19895004|2025-05-14	TCCGGGTGTGACCGAAAGGTAAGATGGAGAGCCTTGTCCCTGGTTTCAACGAGAAAACAC	252
hCoV-19/France/OCC-RELAB-HCL725000064101/2025|EPI_ISL_19895005|2025-05-14	TCCGGGTGTGACCGAAAGGTAAGATGGAGAGCCTTGTCCCTGGTTTCAACGAGAAAACAC	252
hCoV-19/France/NAQ-RELAB-HCL725000062601/2025|EPI_ISL_19895009|2025-05-16	TCCGGGTGTGACCGAAAGGTAAGATGGAGAGCCTTGTCCCTGGTTTCAACGAGAAAACAC	252
hCoV-19/France/ARA-RELAB-HCL725000062401/2025|EPI_ISL_19895017|2025-05-16	TCCGGGTGTGACCGAAAGGTAAGATGGAGAGCCTTGTCCCTGGTTTCAACGAGAAAACAC	252
hCoV-19/France/ARA-RELAB-HCL725000062101/2025|EPI_ISL_19895018|2025-05-13	TCCGGGTGTGACCGAAAGGTAAGATGGAGAGCCTTGTCCCTGGTTTCAACGAGAAAACAC	252
hCoV-19/France/ARA-HCL125019625101/2025|EPI_ISL_19895034|2025-05-21	TCCGGGTGTGACCGAAAGGTAAGATGGAGAGCCTTGTCCCTGGTTTCAACGAGAAAACAC	252
hCoV-19/France/ARA-HCL125019754801/2025|EPI_ISL_19895035|2025-05-22	TCCGGGTGTGACCGAAAGGTAAGATGGAGAGCCTTGTCCCTGGTTTCAACGAGAAAACAC	252
hCoV-19/France/ARA-RELAB-HCL725000064601/2025|EPI_ISL_19895041|2025-05-15	TCCGGGTGTGACCGAAAGGTAAGATGGAGAGCCTTGTCCCTGGTTTCAACGAGAAAACAC	252
hCoV-19/France/ARA-RELAB-HCL725000066001/2025|EPI_ISL_19895046|2025-05-13	TCCGGGTGTGACCGAAAGGTAAGATGGAGAGCCTTGTCCCTGGTTTCAACGAGAAAACAC	252
hCoV-19/France/OCC-RELAB-HCL725000068701/2025|EPI_ISL_19905466|2025-05-21	TCCGGGTGTGACCGAAAGGTAAGATGGAGAGCCTTGTCCCTGGTTTCAACGAGAAAACAC	252
hCoV-19/France/OCC-RELAB-HCL725000069001/2025|EPI_ISL_19905468|2025-05-21	TCCGGGTGTGACCGAAAGGTAAGATGGAGAGCCTTGTCCCTGGTTTCAACGAGAAAACAC	252
hCoV-19/France/ARA-HCL025089279801/2025|EPI_ISL_19905469|2025-06-02	TCCGGGTGTGACCGAAAGGTAAGATGGAGAGCCTTGTCCCTGGTTTCAACGAGAAAACAC	252
hCoV-19/France/PAC-RELAB-HCL725000069901/2025|EPI_ISL_19908847|2025-05-26	TCCGGGTGTGACCGAAAGGTAAGATGGAGAGCCTTGTCCCTGGTTTCAACGAGAAAACAC	252
hCoV-19/France/PAC-RELAB-HCL725000069501/2025|EPI_ISL_19908849|2025-05-30	TCCGGGTGTGACCGAAAGGTAAGATGGAGAGCCTTGTCCCTGGTTTCAACGAGAAAACAC	252
hCoV-19/France/ARA-RELAB-HCL725000071701/2025|EPI_ISL_19908858|2025-05-22	TCCGGGTGTGACCGAAAGGTAAGATGGAGAGCCTTGTCCCTGGTTTCAACGAGAAAACAC	252
hCoV-19/France/ARA-HCL025093352901/2025|EPI_ISL_19908867|2025-06-10	TCCGGGTGTGACCGAAAGGTAAGATGGAGAGCCTTGTCCCTGGTTTCAACGAGAAAACAC	252
hCoV-19/France/ARA-RELAB-HCL725000065801/2025|EPI_ISL_19895045|2025-05-16	TCCGGGTGTGACCGAAAGGTAAGATGGAGAGCCTTGTCCCTGGTTTCAACGAGAAAACAC	252
hCoV-19/Denmark/DCGC-692234/2025|EPI_ISL_19879694|2025-05-05	TCCGGGTGTGACCGAAAGGTAAGATGGAGAGCCTTGTCCCTGGTTTCAACGAGAAAACAC	254
hCoV-19/Finland/THL-02388/2025|EPI_ISL_19875931|2025-04-16	TCCGGGTGTGACCGAAAGGTAAGATGGAGAGCCTTGTCCCTGGTTTCAACGAGAAAACAC	255
hCoV-19/Spain/PV-HUD-98024244/2025|EPI_ISL_19907759|2025-06-09	TCCGGGTGTGACCGAAAGGTAAGATGGAGAGCCTTGTCCCTGGTTTCAACGAGAAAACAC	255
hCoV-19/Netherlands/GE-RIVM-145386/2025|EPI_ISL_19873764|2025-05-01	TCCGGGTGTGACCGAAAGGTAAGATGGAGAGCCTTGTCCCTGGTTTCAACGAGAAAACAC	252
hCoV-19/Netherlands/ZH-RIVM-145413/2025|EPI_ISL_19891397|2025-04-30	TCCGGGTGTGACCGAAAGGTAAGATGGAGAGCCTTGTCCCTGGTTTCAACGAGAAAACAC	252
hCoV-19/Netherlands/NH-RIVM-145458/2025|EPI_ISL_19905062|2025-05-25	TCCGGGTGTGACCGAAAGGTAAGATGGAGAGCCTTGTCCCTGGTTTCAACGAGAAAACAC	252
hCoV-19/Netherlands/NH-RIVM-145463/2025|EPI_ISL_19905063|2025-05-20	TCCGGGTGTGACCGAAAGGTAAGATGGAGAGCCTTGTCCCTGGTTTCAACGAGAAAACAC	252
hCoV-19/Netherlands/UT-RIVM-145436/2025|EPI_ISL_19905072|2025-05-15	TCCGGGTGTGACCGAAAGGTAAGATGGAGAGCCTTGTCCCTGGTTTCAACGAGAAAACAC	252
hCoV-19/Netherlands/UT-RIVM-145470/2025|EPI_ISL_19905077|2025-06-02	TCCGGGTGTGACCGAAAGGTAAGATGGAGAGCCTTGTCCCTGGTTTCAACGAGAAAACAC	252
hCoV-19/Netherlands/ZH-RIVM-145478/2025|EPI_ISL_19905094|2025-05-30	TCCGGGTGTGACCGAAAGGTAAGATGGAGAGCCTTGTCCCTGGTTTCAACGAGAAAACAC	252
hCoV-19/Netherlands/ZH-RIVM-145480/2025|EPI_ISL_19905096|2025-05-31	TCCGGGTGTGACCGAAAGGTAAGATGGAGAGCCTTGTCCCTGGTTTCAACGAGAAAACAC	252
hCoV-19/Netherlands/UT-RIVM-145481/2025|EPI_ISL_19905078|2025-05-31	TCCGGGTGTGACCGAAAGGTAAGATGGAGAGCCTTGTCCCTGGTTTCAACGAGAAAACAC	252
hCoV-19/Scotland/CLIMB-CM7YRW9H/2025|EPI_ISL_19881711|2025-05-06	TCCGGGTGTGACCGAAAGGTAAGATGGAGAGCCTTGTCCCTGGTTTCAACGAGAAAACAC	224
hCoV-19/France/IDF-RELAB-IPP04872/2025|EPI_ISL_19911293|2025-05-22	TCCGGGTGTGACCGAAAGGTAAGATGGAGAGCCTTGTCCCTGGTTTCAACGAGAAAACAC	220
hCoV-19/Canary_Islands/CN-HUC-925112913/2025|EPI_ISL_19900668|2025-04-22	TCCGGGTGTGACCGAAAGGTAAGATGGAGAGCCTTGTCCCTGGTTTCAACGAGAAAACAC	228
hCoV-19/Scotland/CLIMB-CM7YJ5BB/2025|EPI_ISL_19906458|2025-05-27	TCCGGGTGTGACCGAAAGGTAAGATGGAGAGCCTTGTCCCTGGTTTCAACGAGAAAACAC	224
hCoV-19/Scotland/CLIMB-CM7Y8PHH/2025|EPI_ISL_19897755|2025-05-14	TCCGGGTGTGACCGAAAGGTAAGATGGAGAGCCTTGTCCCTGGTTTCAACGAGAAAACAC	224
hCoV-19/Spain/AN-PMC-42706/2025|EPI_ISL_19888325|2025-05-25	TCCGGGTGTGACCGAAAGGTAAGATGGAGAGCCTTGTCCCTGGTTTCAACGAGAAAACAC	224

```
hCoV-19/Scotland/CLIMB-CM7YRIA1/2025|EPI_ISL_19869961|2025-04-20          TCCGGGTGTGACCGAAAGGTAAGATGGAGAGCCTTGTCCCTGGTTTCAACGAGAAAACAC 224
hCoV-19/Scotland/CLIMB-CM7Y8UWT/2025|EPI_ISL_19869966|2025-04-21          TCCGGGTGTGACCGAAAGGTAAGATGGAGAGCCTTGTCCCTGGTTTCAACGAGAAAACAC 224
hCoV-19/England/CLIMB-CM7YMFHB/2025|EPI_ISL_19877436|2025-05-10           TCCGGGTGTGACCGAAAGGTAAGATGGAGAGCCTTGTCCCTGGTTTCAACGAGAAAACAC 224
hCoV-19/Denmark/DCGC-692213/2025|EPI_ISL_19879673|2025-04-28              TCCGGGTGTGACCGAAAGGTAAGATGGAGAGCCTTGTCCCTGGTTTCAACGAGAAAACAC 224
hCoV-19/Wales/CLIMB-CM7YRAXQ/2025|EPI_ISL_19883075|2025-05-14             TCCGGGTGTGACCGAAAGGTAAGATGGAGAGCCTTGTCCCTGGTTTCAACGAGAAAACAC 224
hCoV-19/Spain/AN-PMC-42696/2025|EPI_ISL_19888316|2025-05-14               TCCGGGTGTGACCGAAAGGTAAGATGGAGAGCCTTGTCCCTGGTTTCAACGAGAAAACAC 224
hCoV-19/Spain/AN-PMC-42719/2025|EPI_ISL_19888338|2025-05-15               TCCGGGTGTGACCGAAAGGTAAGATGGAGAGCCTTGTCCCTGGTTTCAACGAGAAAACAC 224
hCoV-19/England/CLIMB-CM7YE7HB/2025|EPI_ISL_19892115|2025-05-19           TCCGGGTGTGACCGAAAGGTAAGATGGAGAGCCTTGTCCCTGGTTTCAACGAGAAAACAC 224
hCoV-19/France/GES-RELAB-IPP04599/2025|EPI_ISL_19893547|2025-05-05        TCCGGGTGTGACCGAAAGGTAAGATGGAGAGCCTTGTCCCTGGTTTCAACGAGAAAACAC 224
hCoV-19/Scotland/CLIMB-CM7YEPXZ/2025|EPI_ISL_19896028|2025-05-25          TCCGGGTGTGACCGAAAGGTAAGATGGAGAGCCTTGTCCCTGGTTTCAACGAGAAAACAC 224
hCoV-19/Scotland/CLIMB-CM7YF4QT/2025|EPI_ISL_19896032|2025-05-27          TCCGGGTGTGACCGAAAGGTAAGATGGAGAGCCTTGTCCCTGGTTTCAACGAGAAAACAC 224
hCoV-19/Scotland/CLIMB-CM7YKDZ6/2025|EPI_ISL_19896033|2025-05-27          TCCGGGTGTGACCGAAAGGTAAGATGGAGAGCCTTGTCCCTGGTTTCAACGAGAAAACAC 224
hCoV-19/Scotland/CLIMB-CM7YG8H4/2025|EPI_ISL_19897748|2025-05-02          TCCGGGTGTGACCGAAAGGTAAGATGGAGAGCCTTGTCCCTGGTTTCAACGAGAAAACAC 224
hCoV-19/Wales/CLIMB-CM7YJXNZ/2025|EPI_ISL_19900036|2025-05-26             TCCGGGTGTGACCGAAAGGTAAGATGGAGAGCCTTGTCCCTGGTTTCAACGAGAAAACAC 224
hCoV-19/Germany/NW-RKI-I-1148178/2025|EPI_ISL_19902055|2025-05-21         TCCGGGTGTGACCGAAAGGTAAGATGGAGAGCCTTGTCCCTGGTTTCAACGAGAAAACAC 224
hCoV-19/Germany/NI-RKI-I-1148183/2025|EPI_ISL_19902060|2025-05-18         TCCGGGTGTGACCGAAAGGTAAGATGGAGAGCCTTGTCCCTGGTTTCAACGAGAAAACAC 224
hCoV-19/Germany/NW-RKI-I-1148191/2025|EPI_ISL_19902068|2025-05-19         TCCGGGTGTGACCGAAAGGTAAGATGGAGAGCCTTGTCCCTGGTTTCAACGAGAAAACAC 224
hCoV-19/Spain/MD-HGUGM-66414184/2025|EPI_ISL_19906298|2025-05-25          TCCGGGTGTGACCGAAAGGTAAGATGGAGAGCCTTGTCCCTGGTTTCAACGAGAAAACAC 224
hCoV-19/Spain/MD-HGUGM-66400167/2025|EPI_ISL_19906320|2025-05-22          TCCGGGTGTGACCGAAAGGTAAGATGGAGAGCCTTGTCCCTGGTTTCAACGAGAAAACAC 224
hCoV-19/France/IDF-RELAB-IPP04646/2025|EPI_ISL_19907194|2025-05-16        TCCGGGTGTGACCGAAAGGTAAGATGGAGAGCCTTGTCCCTGGTTTCAACGAGAAAACAC 224
hCoV-19/England/CLIMB-CM7YKIUQ/2025|EPI_ISL_19908573|2025-06-07           TCCGGGTGTGACCGAAAGGTAAGATGGAGAGCCTTGTCCCTGGTTTCAACGAGAAAACAC 224
hCoV-19/France/GES-RELAB-IPP04823/2025|EPI_ISL_19911257|2025-05-26        TCCGGGTGTGACCGAAAGGTAAGATGGAGAGCCTTGTCCCTGGTTTCAACGAGAAAACAC 224
hCoV-19/France/IDF-RELAB-IPP04863/2025|EPI_ISL_19911265|2025-05-24        TCCGGGTGTGACCGAAAGGTAAGATGGAGAGCCTTGTCCCTGGTTTCAACGAGAAAACAC 224
hCoV-19/Denmark/DCGC-692245/2025|EPI_ISL_19879704|2025-04-28              TCCGGGTGTGACCGAAAGGTAAGATGGAGAGCCTTGTCCCTGGTTTCAACGAGAAAACAC 224


hCoV-19/Ireland/D-SVUH-FBA98650-31/2025|EPI_ISL_19866548|2025-05-04       ------------------------------------------------------------ 0
hCoV-19/Spain/PV-HUB-5628663/2025|EPI_ISL_19908839|2025-05-16             ACGTCCAACTCAGTTTGCCTGTTTTACAGGTTCGCGACGTGCTCGTACGTGGCTTTGGAG 157
hCoV-19/Spain/PV-HUB-41587498/2025|EPI_ISL_19908930|2025-05-20            ACGTCCAACTCAGTTTGCCTGTTTTACAGGTTCGCGACGTGCTCGTACGTGGCTTTGGAG 152
hCoV-19/Spain/PV-HUB-56291851/2025|EPI_ISL_19908970|2025-05-31            ACGTCCAACTCAGTTTGCCTGTTTTACAGGTTCGCGACGTGCTCGTACGTGGCTTTGGAG 153
hCoV-19/Northern_Ireland/CLIMB-CM7YR8I6/2025|EPI_ISL_19877334|2025-04-23  ACGTCCAACTCAGTTTGCCTGTTTTACAGGTTCGCGACGTGCTCGTACGTGGCTTTGGAG 261
hCoV-19/Germany/NW-RKI-I-1148136/2025|EPI_ISL_19871950|2025-04-23         ACGTCCAACTCAGTTTGCCTGTTTTACAGGTTCGCGACGTGCTCGTACGTGGCTTTGGAG 284
hCoV-19/Germany/NW-RKI-I-1148141/2025|EPI_ISL_19871955|2025-04-23         ACGTCCAACTCAGTTTGCCTGTTTTACAGGTTCGCGACGTGCTCGTACGTGGCTTTGGAG 284
hCoV-19/Germany/BW-RKI-I-1148164/2025|EPI_ISL_19884987|2025-04-17         ACGTCCAACTCAGTTTGCCTGTTTTACAGGTTCGCGACGTGCTCGTACGTGGCTTTGGAG 284
hCoV-19/Scotland/CLIMB-CM7YFGW7/2025|EPI_ISL_19897691|2025-05-24          ACGTCCAACTCAGTTTGCCTGTTTTACAGGTTCGCGACGTGCTCGTACGTGGCTTTGGAG 284
hCoV-19/Ireland/G-LUH-N3473/2025|EPI_ISL_19893181|2025-05-14              ACGTCCAACTCAGTTTGCCTGTTTTACAGGTTCGCGACGTGCTCGTACGTGGCTTTGGAG 351
hCoV-19/Spain/AN-PMC-42728/2025|EPI_ISL_19888347|2025-05-25               ACGTCCAACTCAGTTTGCCTGTTTTACAGGTTCGCGACGTGCTCGTACGTGGCTTTGGAG 352
hCoV-19/Sweden/000200866492N2/2025|EPI_ISL_19870050|2025-04-22            ACGTCCAACTCAGTTTGCCTGTTTTACAGGTTCGCGACGTGCTCGTACGTGGCTTTGGAG 358
hCoV-19/Sweden/000200867159N2/2025|EPI_ISL_19870052|2025-04-23            ACGTCCAACTCAGTTTGCCTGTTTTACAGGTTCGCGACGTGCTCGTACGTGGCTTTGGAG 358
hCoV-19/Ireland/G-LUH-N3445/2025|EPI_ISL_19893154|2025-05-07              ACGTCCAACTCAGTTTGCCTGTTTTACAGGTTCGCGACGTGCTCGTACGTGGCTTTGGAG 358
hCoV-19/Ireland/G-LUH-N3443/2025|EPI_ISL_19893152|2025-05-07              ACGTCCAACTCAGTTTGCCTGTTTTACAGGTTCGCGACGTGCTCGTACGTGGCTTTGGAG 356
hCoV-19/Scotland/CLIMB-CM7YF9PJ/2025|EPI_ISL_19895983|2025-05-19          ACGTCCAACTCAGTTTGCCTGTTTTACAGGTTCGCGACGTGCTCGTACGTGGCTTTGGAG 355
hCoV-19/Scotland/CLIMB-CM7YKMK8/2025|EPI_ISL_19869976|2025-04-25          ACGTCCAACTCAGTTTGCCTGTTTTACAGGTTCGCGACGTGCTCGTACGTGGCTTTGGAG 354
hCoV-19/Ireland/G-LUH-N3444/2025|EPI_ISL_19893153|2025-05-07              ACGTCCAACTCAGTTTGCCTGTTTTACAGGTTCGCGACGTGCTCGTACGTGGCTTTGGAG 354
hCoV-19/Switzerland/VD-CHUV-8604552682/2025|EPI_ISL_19906527|2025-05-19   ACGTCCAACTCAGTTTGCCTGTTTTACAGGTTCGCGACGTGCTCGTACGTGGCTTTGGAG 359
hCoV-19/Scotland/CLIMB-CM7YR3YO/2025|EPI_ISL_19896015|2025-05-24          ACGTCCAACTCAGTTTGCCTGTTTTACAGGTTCGCGACGTGCTCGTACGTGGCTTTGGAG 360
hCoV-19/Sweden/O-18_SE100_25CS500180/2025|EPI_ISL_19866288|2025-04-15     ACGTCCAACTCAGTTTGCCTGTTTTACAGGTTCGCGACGTGCTCGTACGTGGCTTTGGAG 360
hCoV-19/Ireland/D-CS250088/2025|EPI_ISL_19877457|2025-04-16               ACGTCCAACTCAGTTTGCCTGTTTTACAGGTTCGCGACGTGCTCGTACGTGGCTTTGGAG 360
hCoV-19/Ireland/G-LUH-N3442/2025|EPI_ISL_19893151|2025-05-07              ACGTCCAACTCAGTTTGCCTGTTTTACAGGTTCGCGACGTGCTCGTACGTGGCTTTGGAG 360
hCoV-19/Scotland/CLIMB-CM7YMZ5M/2025|EPI_ISL_19906439|2025-05-22          ACGTCCAACTCAGTTTGCCTGTTTTACAGGTTCGCGACGTGCTCGTACGTGGCTTTGGAG 359
hCoV-19/Scotland/CLIMB-CM7YR3ZG/2025|EPI_ISL_19897722|2025-05-13          ACGTCCAACTCAGTTTGCCTGTTTTACAGGTTCGCGACGTGCTCGTACGTGGCTTTGGAG 358
hCoV-19/Scotland/CLIMB-CM7YEYGT/2025|EPI_ISL_19897741|2025-04-28          ACGTCCAACTCAGTTTGCCTGTTTTACAGGTTCGCGACGTGCTCGTACGTGGCTTTGGAG 358
hCoV-19/Scotland/CLIMB-CM7YGAJ3/2025|EPI_ISL_19895963|2025-05-13          ACGTCCAACTCAGTTTGCCTGTTTTACAGGTTCGCGACGTGCTCGTACGTGGCTTTGGAG 347
```

```
hCoV-19/Scotland/CLIMB-CM7YJ1ZO/2025|EPI_ISL_19897757|2025-05-14          ACGTCCAACTCAGTTTGCCTGTTTTACAGGTTCGCGACGTGCTCGTACGTGGCTTTGGAG 348
hCoV-19/Scotland/CLIMB-CM7Y8C4Y/2025|EPI_ISL_19906404|2025-05-15          ACGTCCAACTCAGTTTGCCTGTTTTACAGGTTCGCGACGTGCTCGTACGTGGCTTTGGAG 355
hCoV-19/Northern_Ireland/CLIMB-CM7YGGYM/2025|EPI_ISL_19897641|2025-05-23  ACGTCCAACTCAGTTTGCCTGTTTTACAGGTTCGCGACGTGCTCGTACGTGGCTTTGGAG 350
hCoV-19/Scotland/CLIMB-CM7YFW7M/2025|EPI_ISL_19881718|2025-05-10          ACGTCCAACTCAGTTTGCCTGTTTTACAGGTTCGCGACGTGCTCGTACGTGGCTTTGGAG 353
hCoV-19/Scotland/CLIMB-CM7YEMXP/2025|EPI_ISL_19888387|2025-05-10          ACGTCCAACTCAGTTTGCCTGTTTTACAGGTTCGCGACGTGCTCGTACGTGGCTTTGGAG 352
hCoV-19/Spain/CT-HUVH-E593511/2025|EPI_ISL_19881731|2025-05-06            ACGTCCAACTCAGTTTGCCTGTTTTACAGGTTCGCGACGTGCTCGTACGTGGCTTTGGAG 305
hCoV-19/Spain/CT-HUJT-E325362/2025|EPI_ISL_19889935|2025-05-20            ACGTCCAACTCAGTTTGCCTGTTTTACAGGTTCGCGACGTGCTCGTACGTGGCTTTGGAG 306
hCoV-19/Spain/PV-HUD-84743204/2025|EPI_ISL_19907746|2025-06-06            ACGTCCAACTCAGTTTGCCTGTTTTACAGGTTCGCGACGTGCTCGTACGTGGCTTTGGAG 307
hCoV-19/Ireland/D-SVUH-FBB21492-54/2025|EPI_ISL_19881758|2025-05-11       ACGTCCAACTCAGTTTGCCTGTTTTACAGGTTCGCGACGTGCTCGTACGTGGCTTTGGAG 308
hCoV-19/Spain/CT-HUVH-E596725/2025|EPI_ISL_19881729|2025-05-10            ACGTCCAACTCAGTTTGCCTGTTTTACAGGTTCGCGACGTGCTCGTACGTGGCTTTGGAG 302
hCoV-19/Spain/CT-HUVH-E593882/2025|EPI_ISL_19881732|2025-05-06            ACGTCCAACTCAGTTTGCCTGTTTTACAGGTTCGCGACGTGCTCGTACGTGGCTTTGGAG 302
hCoV-19/Finland/THL-02385/2025|EPI_ISL_19875928|2025-04-16                ACGTCCAACTCAGTTTGCCTGTTTTACAGGTTCGCGACGTGCTCGTACGTGGCTTTGGAG 309
hCoV-19/Ireland/D-BH-02351167/2025|EPI_ISL_19911337|2025-05-28            ACGTCCAACTCAGTTTGCCTGTTTTACAGGTTCGCGACGTGCTCGTACGTGGCTTTGGAG 308
hCoV-19/Ireland/D-NVRL-XIRL00001782/2025|EPI_ISL_19896104|2025-05-14      ACGTCCAACTCAGTTTGCCTGTTTTACAGGTTCGCGACGTGCTCGTACGTGGCTTTGGAG 308
hCoV-19/Ireland/TA-NVRL-GIRL00039444/2025|EPI_ISL_19896118|2025-05-16     ACGTCCAACTCAGTTTGCCTGTTTTACAGGTTCGCGACGTGCTCGTACGTGGCTTTGGAG 308
hCoV-19/Spain/GA-CHUAC-13223/2025|EPI_ISL_19894946|2025-05-31             ACGTCCAATTCAGTTTGCCTGTTTTACAGGTTCGCGACGTGCTCGTACGTGGCTTTGGAG 307
hCoV-19/Spain/GA-CHUAC-13215/2025|EPI_ISL_19894939|2025-05-29             ACGTCCAACTCAGTTTGCCTGTTTTACAGGTTCGCGACGTGCTCGTACGTGGCTTTGGAG 307
hCoV-19/Finland/THL-02398/2025|EPI_ISL_19875939|2025-05-04                ACGTCCAACTCAGTTTGCCTGTTTTACAGGTTCGCGACGTGCTCGTACGTGGCTTTGGAG 308
hCoV-19/Finland/THL-02400/2025|EPI_ISL_19875941|2025-05-03                ACGTCCAACTCAGTTTGCCTGTTTTACAGGTTCGCGACGTGCTCGTACGTGGCTTTGGAG 308
hCoV-19/Ireland/LK-UHL-702929/2025|EPI_ISL_19877895|2025-04-16            ACGTCCAACTCAGTTTGCCTGTTTTACAGGTTCGCGACGTGCTCGTACGTGGCTTTGGAG 308
hCoV-19/Ireland/LK-UHL-703788/2025|EPI_ISL_19877905|2025-05-03            ACGTCCAACTCAGTTTGCCTGTTTTACAGGTTCGCGACGTGCTCGTACGTGGCTTTGGAG 308
hCoV-19/Ireland/LK-UHL-703851/2025|EPI_ISL_19877907|2025-05-04            ACGTCCAACTCAGTTTGCCTGTTTTACAGGTTCGCGACGTGCTCGTACGTGGCTTTGGAG 308
hCoV-19/Ireland/LK-UHL-703889/2025|EPI_ISL_19877908|2025-05-05            ACGTCCAACTCAGTTTGCCTGTTTTACAGGTTCGCGACGTGCTCGTACGTGGCTTTGGAG 308
hCoV-19/Ireland/D-SVUH-FBB21492-49/2025|EPI_ISL_19881756|2025-05-14       ACGTCCAACTCAGTTTGCCTGTTTTACAGGTTCGCGACGTGCTCGTACGTGGCTTTGGAG 308
hCoV-19/Ireland/D-SVUH-FBB21492-59/2025|EPI_ISL_19881763|2025-05-07       ACGTCCAACTCAGTTTGCCTGTTTTACAGGTTCGCGACGTGCTCGTACGTGGCTTTGGAG 308
hCoV-19/Spain/CT-HUGTiP-G3R212/2025|EPI_ISL_19883055|2025-05-20           ACGTCCAACTCAGTTTGCCTGTTTTACAGGTTCGCGACGTGCTCGTACGTGGCTTTGGAG 308
hCoV-19/Ireland/LK-UHL-704398/2025|EPI_ISL_19888621|2025-05-16            ACGTCCAACTCAGTTTGCCTGTTTTACAGGTTCGCGACGTGCTCGTACGTGGCTTTGGAG 308
hCoV-19/Ireland/G-GUH-N3466/2025|EPI_ISL_19893175|2025-05-03              ACGTCCAACTCAGTTTGCCTGTTTTACAGGTTCGCGACGTGCTCGTACGTGGCTTTGGAG 308
hCoV-19/Belgium/Sciensano-LS-S5276/2025|EPI_ISL_19893834|2025-04-22       ACGTCCAACTCAGTTTGCCTGTTTTACAGGTTCGCGACGTGCTCGTACGTGGCTTTGGAG 308
hCoV-19/Italy/VEN-ULSS8-0019244_VI/2025|EPI_ISL_19899362|2025-05-26       ACGTCCAACTCAGTTTGCCTGTTTTACAGGTTCGCGACGTGCTCGTACGTGGCTTTGGAG 308
hCoV-19/Spain/CT-HUVH-E618109/2025|EPI_ISL_19907773|2025-06-10            ACGTCCAACTCAGTTTGCCTGTTTTACAGGTTCGCGACGTGCTCGTACGTGGCTTTGGAG 308
hCoV-19/Ireland/D-BH-03819149/2025|EPI_ISL_19911343|2025-05-29            ACGTCCAACTCAGTTTGCCTGTTTTACAGGTTCGCGACGTGCTCGTACGTGGCTTTGGAG 308
hCoV-19/Ireland/D-BH-03993161/2025|EPI_ISL_19911355|2025-06-10            ACGTCCAACTCAGTTTGCCTGTTTTACAGGTTCGCGACGTGCTCGTACGTGGCTTTGGAG 308
hCoV-19/Spain/CT-HUVH-E587040/2025|EPI_ISL_19871881|2025-04-26            ACGTCCAACTCAGTTTGCCTGTTTTACAGGTTCGCGACGTGCTCGTACGTGGCTTTGGAG 307
hCoV-19/Finland/THL-02392/2025|EPI_ISL_19875934|2025-05-02                ACGTCCAACTCAGTTTGCCTGTTTTACAGGTTCGCGACGTGCTCGTACGTGGCTTTGGAG 307
hCoV-19/Spain/GA-CHUAC-13181/2025|EPI_ISL_19890617|2025-05-20             ACGTCCAACTCAGTTTGCCTGTTTTACAGGTTCGCGACGTGCTCGTACGTGGCTTTGGAG 307
hCoV-19/Spain/CT-HUVH-E614843/2025|EPI_ISL_19907763|2025-06-05            ACGTCCAACTCAGTTTGCCTGTTTTACAGGTTCGCGACGTGCTCGTACGTGGCTTTGGAG 307
hCoV-19/Italy/UMB-LAOP-172-4-02/2025|EPI_ISL_19899474|2025-05-08          ACGTCCAACTCAGTTTGCCTGTTTTACAGGTTCGCGACGTGCTCGTACGTGGCTTTGGAG 329
hCoV-19/Spain/NC-CHN-01008932/2025|EPI_ISL_19900595|2025-04-04            ACGTCCAACTCAGTTTGCCTGTTTTACAGGTTCGCGACGTGCTCGTACGTGGCTTTGGAG 331
hCoV-19/Northern_Ireland/CLIMB-CM7YGDRO/2025|EPI_ISL_19877331|2025-04-14  ACGTCCAACTCAGTTTGCCTGTTTTACAGGTTCGCGACGTGCTCGTACGTGGCTTTGGAG 319
hCoV-19/Scotland/CLIMB-CM7YEE96/2025|EPI_ISL_19897754|2025-05-13          ACGTCCAACTCAGTTTGCCTGTTTTACAGGTTCGCGACGTGCTCGTACGTGGCTTTGGAG 318
hCoV-19/France/ARA-HCL125018289001/2025|EPI_ISL_19880005|2025-05-12       ACGTCCAACTCAGTTTGCCTGTTTTACAGGTTCGCGACGTGCTCGTACGTGGCTTTGGAG 312
hCoV-19/France/ARA-RELAB-HCL725000057501/2025|EPI_ISL_19880014|2025-04-28 ACGTCCAACTCAGTTTGCCTGTTTTACAGGTTCGCGACGTGCTCGTACGTGGCTTTGGAG 312
hCoV-19/France/ARA-RELAB-HCL725000059501/2025|EPI_ISL_19885160|2025-04-25 ACGTCCAACTCAGTTTGCCTGTTTTACAGGTTCGCGACGTGCTCGTACGTGGCTTTGGAG 312
hCoV-19/France/NAQ-RELAB-HCL725000061001/2025|EPI_ISL_19885161|2025-05-10 ACGTCCAACTCAGTTTGCCTGTTTTACAGGTTCGCGACGTGCTCGTACGTGGCTTTGGAG 312
hCoV-19/France/NAQ-RELAB-HCL725000060901/2025|EPI_ISL_19885180|2025-05-09 ACGTCCAACTCAGTTTGCCTGTTTTACAGGTTCGCGACGTGCTCGTACGTGGCTTTGGAG 312
hCoV-19/France/ARA-HCL025075546601/2025|EPI_ISL_19885182|2025-05-09       ACGTCCAACTCAGTTTGCCTGTTTTACAGGTTCGCGACGTGCTCGTACGTGGCTTTGGAG 312
hCoV-19/France/ARA-HCL125018461901/2025|EPI_ISL_19885204|2025-05-14       ACGTCCAACTCAGTTTGCCTGTTTTACAGGTTCGCGACGTGCTCGTACGTGGCTTTGGAG 312
hCoV-19/France/ARA-HCL125018655901/2025|EPI_ISL_19885206|2025-05-14       ACGTCCAACTCAGTTTGCCTGTTTTACAGGTTCGCGACGTGCTCGTACGTGGCTTTGGAG 312
hCoV-19/France/ARA-HCL125019155501/2025|EPI_ISL_19885209|2025-05-18       ACGTCCAACTCAGTTTGCCTGTTTTACAGGTTCGCGACGTGCTCGTACGTGGCTTTGGAG 312
hCoV-19/France/ARA-HCL125019240901/2025|EPI_ISL_19885210|2025-05-19       ACGTCCAACTCAGTTTGCCTGTTTTACAGGTTCGCGACGTGCTCGTACGTGGCTTTGGAG 312
hCoV-19/France/ARA-HCL125019249201/2025|EPI_ISL_19885211|2025-05-19       ACGTCCAACTCAGTTTGCCTGTTTTACAGGTTCGCGACGTGCTCGTACGTGGCTTTGGAG 312
hCoV-19/France/ARA-HCL125019399701/2025|EPI_ISL_19885212|2025-05-20       ACGTCCAACTCAGTTTGCCTGTTTTACAGGTTCGCGACGTGCTCGTACGTGGCTTTGGAG 312
```

hCoV-19/France/ARA-HCL125019408901/2025|EPI_ISL_19885213|2025-05-20	ACGTCCAACTCAGTTTGCCTGTTTTACAGGTTCGCGACGTGCTCGTACGTGGCTTTGGAG	312
hCoV-19/France/OCC-RELAB-HCL725000064001/2025|EPI_ISL_19895004|2025-05-14	ACGTCCAACTCAGTTTGCCTGTTTTACAGGTTCGCGACGTGCTCGTACGTGGCTTTGGAG	312
hCoV-19/France/OCC-RELAB-HCL725000064101/2025|EPI_ISL_19895005|2025-05-14	ACGTCCAACTCAGTTTGCCTGTTTTACAGGTTCGCGACGTGCTCGTACGTGGCTTTGGAG	312
hCoV-19/France/NAQ-RELAB-HCL725000062601/2025|EPI_ISL_19895009|2025-05-16	ACGTCCAACTCAGTTTGCCTGTTTTACAGGTTCGCGACGTGCTCGTACGTGGCTTTGGAG	312
hCoV-19/France/ARA-RELAB-HCL725000062401/2025|EPI_ISL_19895017|2025-05-16	ACGTCCAACTCAGTTTGCCTGTTTTACAGGTTCGCGACGTGCTCGTACGTGGCTTTGGAG	312
hCoV-19/France/ARA-RELAB-HCL725000062101/2025|EPI_ISL_19895018|2025-05-13	ACGTCCAACTCAGTTTGCCTGTTTTACAGGTTCGCGACGTGCTCGTACGTGGCTTTGGAG	312
hCoV-19/France/ARA-HCL125019625101/2025|EPI_ISL_19895034|2025-05-21	ACGTCCAACTCAGTTTGCCTGTTTTACAGGTTCGCGACGTGCTCGTACGTGGCTTTGGAG	312
hCoV-19/France/ARA-HCL125019754801/2025|EPI_ISL_19895035|2025-05-22	ACGTCCAACTCAGTTTGCCTGTTTTACAGGTTCGCGACGTGCTCGTACGTGGCTTTGGAG	312
hCoV-19/France/ARA-RELAB-HCL725000064601/2025|EPI_ISL_19895041|2025-05-15	ACGTCCAACTCAGTTTGCCTGTTTTACAGGTTCGCGACGTGCTCGTACGTGGCTTTGGAG	312
hCoV-19/France/ARA-RELAB-HCL725000066001/2025|EPI_ISL_19895046|2025-05-13	ACGTCCAACTCAGTTTGCCTGTTTTACAGGTTCGCGACGTGCTCGTACGTGGCTTTGGAG	312
hCoV-19/France/OCC-RELAB-HCL725000068701/2025|EPI_ISL_19905466|2025-05-21	ACGTCCAACTCAGTTTGCCTGTTTTACAGGTTCGCGACGTGCTCGTACGTGGCTTTGGAG	312
hCoV-19/France/OCC-RELAB-HCL725000069001/2025|EPI_ISL_19905468|2025-05-21	ACGTCCAACTCAGTTTGCCTGTTTTACAGGTTCGCGACGTGCTCGTACGTGGCTTTGGAG	312
hCoV-19/France/ARA-HCL025089279801/2025|EPI_ISL_19905469|2025-06-02	ACGTCCAACTCAGTTTGCCTGTTTTACAGGTTCGCGACGTGCTCGTACGTGGCTTTGGAG	312
hCoV-19/France/PAC-RELAB-HCL725000069901/2025|EPI_ISL_19908847|2025-05-26	ACGTCCAACTCAGTTTGCCTGTTTTACAGGTTCGCGACGTGCTCGTACGTGGCTTTGGAG	312
hCoV-19/France/PAC-RELAB-HCL725000069501/2025|EPI_ISL_19908849|2025-05-30	ACGTCCAACTCAGTTTGCCTGTTTTACAGGTTCGCGACGTGCTCGTACGTGGCTTTGGAG	312
hCoV-19/France/ARA-RELAB-HCL725000071701/2025|EPI_ISL_19908858|2025-05-22	ACGTCCAACTCAGTTTGCCTGTTTTACAGGTTCGCGACGTGCTCGTACGTGGCTTTGGAG	312
hCoV-19/France/ARA-HCL025093352901/2025|EPI_ISL_19908867|2025-06-10	ACGTCCAACTCAGTTTGCCTGTTTTACAGGTTCGCGACGTGCTCGTACGTGGCTTTGGAG	312
hCoV-19/France/ARA-RELAB-HCL725000065801/2025|EPI_ISL_19895045|2025-05-16	ACGTCCAACTCAGTTTGCCTGTTTTACAGGTTCGCGACGTGCTCGTACGTGGCTTTGGAG	312
hCoV-19/Denmark/DCGC-692234/2025|EPI_ISL_19879694|2025-05-05	ACGTCCAACTCAGTTTGCCTGTTTTACAGGTTCGCGACGTGCTCGTACGTGGCTTTGGAG	314
hCoV-19/Finland/THL-02388/2025|EPI_ISL_19875931|2025-04-16	ACGTCCAACTCAGTTTGCCTGTTTTACAGGTTCGCGACGTGCTCGTACGTGGCTTTGGAG	315
hCoV-19/Spain/PV-HUD-98024244/2025|EPI_ISL_19907759|2025-06-09	ACGTCCAACTCAGTTTGCCTGTTTTACAGGTTCGCGACGTGCTCGTACGTGGCTTTGGAG	315
hCoV-19/Netherlands/GE-RIVM-145386/2025|EPI_ISL_19873764|2025-05-01	ACGTCCAACTCAGTTTGCCTGTTTTACAGGTTCGCGACGTGCTCGTACGTGGCTTTGGAG	312
hCoV-19/Netherlands/ZH-RIVM-145413/2025|EPI_ISL_19891397|2025-04-30	ACGTCCAACTCAGTTTGCCTGTTTTACAGGTTCGCGACGTGCTCGTACGTGGCTTTGGAG	312
hCoV-19/Netherlands/NH-RIVM-145458/2025|EPI_ISL_19905062|2025-05-25	ACGTCCAACTCAGTTTGCCTGTTTTACAGGTTCGCGACGTGCTCGTACGTGGCTTTGGAG	312
hCoV-19/Netherlands/NH-RIVM-145463/2025|EPI_ISL_19905063|2025-05-20	ACGTCCAACTCAGTTTGCCTGTTTTACAGGTTCGCGACGTGCTCGTACGTGGCTTTGGAG	312
hCoV-19/Netherlands/UT-RIVM-145436/2025|EPI_ISL_19905072|2025-05-15	ACGTCCAACTCAGTTTGCCTGTTTTACAGGTTCGCGACGTGCTCGTACGTGGCTTTGGAG	312
hCoV-19/Netherlands/UT-RIVM-145470/2025|EPI_ISL_19905077|2025-06-02	ACGTCCAACTCAGTTTGCCTGTTTTACAGGTTCGCGACGTGCTCGTACGTGGCTTTGGAG	312
hCoV-19/Netherlands/ZH-RIVM-145478/2025|EPI_ISL_19905094|2025-05-30	ACGTCCAACTCAGTTTGCCTGTTTTACAGGTTCGCGACGTGCTCGTACGTGGCTTTGGAG	312
hCoV-19/Netherlands/ZH-RIVM-145480/2025|EPI_ISL_19905096|2025-05-31	ACGTCCAACTCAGTTTGCCTGTTTTACAGGTTCGCGACGTGCTCGTACGTGGCTTTGGAG	312
hCoV-19/Netherlands/UT-RIVM-145481/2025|EPI_ISL_19905078|2025-05-31	ACGTCCAACTCAGTTTGCCTGTTTTACAGGTTCGCGACGTGCTCGTACGTGGCTTTGGAG	312
hCoV-19/Scotland/CLIMB-CM7YRW9H/2025|EPI_ISL_19881711|2025-05-06	ACGTCCAACTCAGTTTGCCTGTTTTACAGGTTCGCGACGTGCTCGTACGTGGCTTTGGAG	284
hCoV-19/France/IDF-RELAB-IPP04872/2025|EPI_ISL_19911293|2025-05-22	ACGTCCAACTCAGTTTGCCTGTTTTACAGGTTCGCGACGTGCTCGTACGTGGCTTTGGAG	280
hCoV-19/Canary_Islands/CN-HUC-925112913/2025|EPI_ISL_19900668|2025-04-22	ACGTCCAACTCAGTTTGCCTGTTTTACAGGTTCGCGACGTGCTCGTACGTGGCTTTGGAG	288
hCoV-19/Scotland/CLIMB-CM7YJ5BB/2025|EPI_ISL_19906458|2025-05-27	ACGTCCAACTCAGTTTGCCTGTTTTACAGGTTCGCGACGTGCTCGTACGTGGCTTTGGAG	284
hCoV-19/Scotland/CLIMB-CM7Y8PHH/2025|EPI_ISL_19897755|2025-05-14	ACGTCCAACTCAGTTTGCCTGTTTTACAGGTTCGCGACGTGCTCGTACGTGGCTTTGGAG	284
hCoV-19/Spain/AN-PMC-42706/2025|EPI_ISL_19888325|2025-05-25	ACGTCCAACTCAGTTTGCCTGTTTTACAGGTTCGTGACGTGCTCGTACGTGGCTTTGGAG	284
hCoV-19/Scotland/CLIMB-CM7YRIA1/2025|EPI_ISL_19869961|2025-04-20	ACGTCCAACTCAGTTTGCCTGTTTTACAGGTTCGCGACGTGCTCGTACGTGGCTTTGGAG	284
hCoV-19/Scotland/CLIMB-CM7Y8UWT/2025|EPI_ISL_19869966|2025-04-21	ACGTCCAACTCAGTTTGCCTGTTTTACAGGTTCGCGACGTGCTCGTACGTGGCTTTGGAG	284
hCoV-19/England/CLIMB-CM7YMFHB/2025|EPI_ISL_19877436|2025-05-10	ACGTCCAACTCAGTTTGCCTGTTTTACAGGTTCGCGACGTGCTCGTACGTGGCTTTGGAG	284
hCoV-19/Denmark/DCGC-692213/2025|EPI_ISL_19879673|2025-04-28	ACGTCCAACTCAGTTTGCCTGTTTTACAGGTTCGCGACGTGCTCGTACGTGGCTTTGGAG	284
hCoV-19/Wales/CLIMB-CM7YRAXQ/2025|EPI_ISL_19883075|2025-05-14	ACGTCCAACTCAGTTTGCCTGTTTTACAGGTTCGCGACGTGCTCGTACGTGGCTTTGGAG	284
hCoV-19/Spain/AN-PMC-42696/2025|EPI_ISL_19888316|2025-05-14	ACGTCCAACTCAGTTTGCCTGTTTTACAGGTTCGCGACGTGCTCGTACGTGGCTTTGGAG	284
hCoV-19/Spain/AN-PMC-42719/2025|EPI_ISL_19888338|2025-05-15	ACGTCCAACTCAGTTTGCCTGTTTTACAGGTTCGCGACGTGCTCGTACGTGGCTTTGGAG	284
hCoV-19/England/CLIMB-CM7YE7HB/2025|EPI_ISL_19892115|2025-05-19	ACGTCCAACTCAGTTTGCCTGTTTTACAGGTTCGCGACGTGCTCGTACGTGGCTTTGGAG	284
hCoV-19/France/GES-RELAB-IPP04599/2025|EPI_ISL_19893547|2025-05-05	ACGTCCAACTCAGTTTGCCTGTTTTACAGGTTCGCGACGTGCTCGTACGTGGCTTTGGAG	284
hCoV-19/Scotland/CLIMB-CM7YEPXZ/2025|EPI_ISL_19896028|2025-05-25	ACGTCCAACTCAGTTTGCCTGTTTTACAGGTTCGCGACGTGCTCGTACGTGGCTTTGGAG	284
hCoV-19/Scotland/CLIMB-CM7YF4QT/2025|EPI_ISL_19896032|2025-05-27	ACGTCCAACTCAGTTTGCCTGTTTTACAGGTTCGCGACGTGCTCGTACGTGGCTTTGGAG	284
hCoV-19/Scotland/CLIMB-CM7YKDZ6/2025|EPI_ISL_19896033|2025-05-27	ACGTCCAACTCAGTTTGCCTGTTTTACAGGTTCGCGACGTGCTCGTACGTGGCTTTGGAG	284
hCoV-19/Scotland/CLIMB-CM7YG8H4/2025|EPI_ISL_19897748|2025-05-02	ACGTCCAACTCAGTTTGCCTGTTTTACAGGTTCGCGACGTGCTCGTACGTGGCTTTGGAG	284
hCoV-19/Wales/CLIMB-CM7YJXNZ/2025|EPI_ISL_19900036|2025-05-26	ACGTCCAACTCAGTTTGCCTGTTTTACAGGTTCGCGACGTGCTCGTACGTGGCTTTGGAG	284
hCoV-19/Germany/NW-RKI-I-1148178/2025|EPI_ISL_19902055|2025-05-21	ACGTCCAACTCAGTTTGCCTGTTTTACAGGTTCGCGACGTGCTCGTACGTGGCTTTGGAG	284
hCoV-19/Germany/NI-RKI-I-1148183/2025|EPI_ISL_19902060|2025-05-18	ACGTCCAACTCAGTTTGCCTGTTTTACAGGTTCGCGACGTGCTCGTACGTGGCTTTGGAG	284
hCoV-19/Germany/NW-RKI-I-1148191/2025|EPI_ISL_19902068|2025-05-19	ACGTCCAACTCAGTTTGCCTGTTTTACAGGTTCGCGACGTGCTCGTACGTGGCTTTGGAG	284

```
hCoV-19/Spain/MD-HGUGM-66414184/2025|EPI_ISL_19906298|2025-05-25          ACGTCCAACTCAGTTTGCCTGTTTTACAGGTTCGCGACGTGCTCGTACGTGGCTTTGGAG 284
hCoV-19/Spain/MD-HGUGM-66400167/2025|EPI_ISL_19906320|2025-05-22          ACGTCCAACTCAGTTTGCCTGTTTTACAGGTTCGCGACGTGCTCGTACGTGGCTTTGGAG 284
hCoV-19/France/IDF-RELAB-IPP04646/2025|EPI_ISL_19907194|2025-05-16        ACGTCCAACTCAGTTTGCCTGTTTTACAGGTTCGCGACGTGCTCGTACGTGGCTTTGGAG 284
hCoV-19/England/CLIMB-CM7YKIUQ/2025|EPI_ISL_19908573|2025-06-07           ACGTCCAACTCAGTTTGCCTGTTTTACAGGTTCGCGACGTGCTCGTACGTGGCTTTGGAG 284
hCoV-19/France/GES-RELAB-IPP04823/2025|EPI_ISL_19911257|2025-05-26        ACGTCCAACTCAGTTTGCCTGTTTTACAGGTTCGCGACGTGCTCGTACGTGGCTTTGGAG 284
hCoV-19/France/IDF-RELAB-IPP04863/2025|EPI_ISL_19911265|2025-05-24        ACGTCCAACTCAGTTTGCCTGTTTTACAGGTTCGCGACGTGCTCGTACGTGGCTTTGGAG 284
hCoV-19/Denmark/DCGC-692245/2025|EPI_ISL_19879704|2025-04-28              ACGTCCAACTCAGTTTGCCTGTTTTACAGGTTCGCGACGTGCTCGTACGTGGCTTTGGAG 284


hCoV-19/Ireland/D-SVUH-FBA98650-31/2025|EPI_ISL_19866548|2025-05-04       ------------------------------------------------------------ 0
hCoV-19/Spain/PV-HUB-5628663/2025|EPI_ISL_19908839|2025-05-16             ACTCCGTGGAGGAGGTCTTATCAGAGGCACGTCAACATCTTAGAGATGGCACTTGTGGCT 217
hCoV-19/Spain/PV-HUB-41587498/2025|EPI_ISL_19908930|2025-05-20            ACTCCGTGGAGGAGGTCTTATCAGAGGCACGTCAACATCTTAGAGATGGCACTTGTGGCT 212
hCoV-19/Spain/PV-HUB-56291851/2025|EPI_ISL_19908970|2025-05-31            ACTCCGTGGAGGAGGTCTTATCAGAGGCACGTCAACATCTTAGAGATGGCACTTGTGGCT 213
hCoV-19/Northern_Ireland/CLIMB-CM7YR8I6/2025|EPI_ISL_19877334|2025-04-23  ACTNCGTGGAGGAGGTCTTATCAGAGGCACGTCAACATCTTAGAGATGGCACTTGTGNNN 321
hCoV-19/Germany/NW-RKI-I-1148136/2025|EPI_ISL_19871950|2025-04-23         ACTCCGTGGAGGAGGTCTTATCAGAGGCACGTCAACATCTTAGAGATGGCACTTGTGNNN 344
hCoV-19/Germany/NW-RKI-I-1148141/2025|EPI_ISL_19871955|2025-04-23         ACTCCGTGGAGGAGGTCTTATCAGAGGCACGTCAACATCTTAGAGATGGCACTTGTGNNN 344
hCoV-19/Germany/BW-RKI-I-1148164/2025|EPI_ISL_19884987|2025-04-17         ACTCCGTGGAGGAGGTCTTATCAGAGGCACGTCAACATCTTAGAGATGGCACTTGTGNNN 344
hCoV-19/Scotland/CLIMB-CM7YFGW7/2025|EPI_ISL_19897691|2025-05-24          ACTCCGTGGAGGAGGTCTTATCAGAGGCACGTCAACATCTTAGAGATGGCACTTGTGNNN 344
hCoV-19/Ireland/G-LUH-N3473/2025|EPI_ISL_19893181|2025-05-14              ACTCCGTGGAGGAGGTCTTATCAGAGGCACGTCAACATCTTAGAGATGGCACTTGTGGCT 411
hCoV-19/Spain/AN-PMC-42728/2025|EPI_ISL_19888347|2025-05-25               ACTCCGTGGAGGAGGTCTTATCAGAGGCACGTCAACATCTTAGAGATGGCACTTGTGGCT 412
hCoV-19/Sweden/000200866492N2/2025|EPI_ISL_19870050|2025-04-22            ACTCCGTGGAGGAGGTCTTATCAGAGGCACGTCAACATCTTAGAGATGGCACTTGTGGCT 418
hCoV-19/Sweden/000200867159N2/2025|EPI_ISL_19870052|2025-04-23            ACTCCGTGGAGGAGGTCTTATCAGAGGCACGTCAACATCTTAGAGATGGCACTTGTGGCT 418
hCoV-19/Ireland/G-LUH-N3445/2025|EPI_ISL_19893154|2025-05-07              ACTCCGTGGAGGAGGTCTTATCAGAGGCACGTCAACATCTTAGAGATGGCACTTGTGGCT 418
hCoV-19/Ireland/G-LUH-N3443/2025|EPI_ISL_19893152|2025-05-07              ACTCCGTGGAGGAGGTCTTATCAGAGGCACGTCAACATCTTAGAGATGGCACTTGTGGCT 416
hCoV-19/Scotland/CLIMB-CM7YF9PJ/2025|EPI_ISL_19895983|2025-05-19          ACTCCGTGGAGGAGGTCTTATCAGAGGCACGTCAACATCTTAGAGATGGCACTTGTGGCT 415
hCoV-19/Scotland/CLIMB-CM7YKMK8/2025|EPI_ISL_19869976|2025-04-25          ACTCCGTGGAGGAGGTCTTATCAGAGGCACGTCAACATCTTAGAGATGGCACTTGTGGCT 414
hCoV-19/Ireland/G-LUH-N3444/2025|EPI_ISL_19893153|2025-05-07              ACTCCGTGGAGGAGGTCTTATCAGAGGCACGTCAACATCTTAGAGATGGCACTTGTGGCT 414
hCoV-19/Switzerland/VD-CHUV-8604552682/2025|EPI_ISL_19906527|2025-05-19   ACTCCGTGGAGGAGGTCTTATCAGAGGCACGTCAACATCTTAGAGATGGCACTTGTGGCT 419
hCoV-19/Scotland/CLIMB-CM7YR3YO/2025|EPI_ISL_19896015|2025-05-24          ACTCCGTGGAGGAGGTCTTATCAGAGGCACGTCAACATCTTAGAGATGGCACTTGTGGCT 420
hCoV-19/Sweden/O-18_SE100_25CS500180/2025|EPI_ISL_19866288|2025-04-15     ACTCCGTGGAGGAGGTCTTATCAGAGGCACGTCAACATCTTAGAGATGGCACTTGTGGCT 420
hCoV-19/Ireland/D-CS250088/2025|EPI_ISL_19877457|2025-04-16               ACTCCGTGGAGGAGGTCTTATCAGAGGCACGTCAACATCTTAGAGATGGCACTTGTGGCT 420
hCoV-19/Ireland/G-LUH-N3442/2025|EPI_ISL_19893151|2025-05-07              ACTCCGTGGAGGAGGTCTTATCAGAGGCACGTCAACATCTTAGAGATGGCACTTGTGGCT 420
hCoV-19/Scotland/CLIMB-CM7YMZ5M/2025|EPI_ISL_19906439|2025-05-22          ACTCCGTGGAGGAGGTCTTATCAGAGGCACGTCAACATCTTAGAGATGGCACTTGTGGCT 419
hCoV-19/Scotland/CLIMB-CM7YR3ZG/2025|EPI_ISL_19897722|2025-05-13          ACTCCGTGGAGGAGGTCTTATCAGAGGCACGTCAACATCTTAGAGATGGCACTTGTGGCT 418
hCoV-19/Scotland/CLIMB-CM7YEYGT/2025|EPI_ISL_19897741|2025-04-28          ACTCCGTGGAGGAGGTCTTATCAGAGGCACGTCAACATCTTAGAGATGGCACTTGTGGCT 418
hCoV-19/Scotland/CLIMB-CM7YGAJ3/2025|EPI_ISL_19895963|2025-05-13          ACTCCGTGGAGGAGGTCTTATCAGAGGCACGTCAACATCTTAGAGATGGCACTTGTGGCT 407
hCoV-19/Scotland/CLIMB-CM7YJ1ZO/2025|EPI_ISL_19897757|2025-05-14          ACTCCGTGGAGGAGGTCTTATCAGAGGCACGTCAACATCTTAGAGATGGCACTTGTGGCT 408
hCoV-19/Scotland/CLIMB-CM7Y8C4Y/2025|EPI_ISL_19906404|2025-05-15          ACTCCGTGGAGGAGGTCTTATCAGAGGCACGTCAACATCTTAGAGATGGCACTTGTGGCT 415
hCoV-19/Northern_Ireland/CLIMB-CM7YGGYM/2025|EPI_ISL_19897641|2025-05-23  ACTCCGTGGAGGAGGTCTTATCAGAGGCACGTCAACATCTTAGAGATGGCACTTGTGGCT 410
hCoV-19/Scotland/CLIMB-CM7YFW7M/2025|EPI_ISL_19881718|2025-05-10          ACTCCGTGGAGGAGGTCTTATCAGAGGCACGTCAACATCTTAGAGATGGCACTTGTGGCT 413
hCoV-19/Scotland/CLIMB-CM7YEMXP/2025|EPI_ISL_19888387|2025-05-10          ACTCCGTGGAGGAGGTCTTATCAGAGGCACGTCAACATCTTAGAGATGGCACTTGTGGCT 412
hCoV-19/Spain/CT-HUVH-E593511/2025|EPI_ISL_19881731|2025-05-06            ACTCCGTGGAGGAGGTCTTATCAGAGGCACGTCAACATCTTAGAGATGGCACTTGTGGCT 365
hCoV-19/Spain/CT-HUJT-E325362/2025|EPI_ISL_19889935|2025-05-20            ACTCCGTGGAGGAGGTCTTATCAGAGGCACGTCAACATCTTAGAGATGGCACTTGTGGCT 366
hCoV-19/Spain/PV-HUD-84743204/2025|EPI_ISL_19907746|2025-06-06            ACTCCGTGGAGGAGGTCTTATCAGAGGCACGTCAACATCTTAGAGATGGCACTTGTGGCT 367
hCoV-19/Ireland/D-SVUH-FBB21492-54/2025|EPI_ISL_19881758|2025-05-11       ACTCCGTGGAGGAGGTCTTATCAGAGGCACGTCAACATCTTAGAGATGGCACTTGTGGCT 368
hCoV-19/Spain/CT-HUVH-E596725/2025|EPI_ISL_19881729|2025-05-10            ACTCCGTGGAGGAGGTCTTATCAGAGGCACGTCAACATCTTAGAGATGGCACTTGTGGCT 362
hCoV-19/Spain/CT-HUVH-E593882/2025|EPI_ISL_19881732|2025-05-06            ACTCCGTGGAGGAGGTCTTATCAGAGGCACGTCAACATCTTAGAGATGGCACTTGTGGCT 362
hCoV-19/Finland/THL-02385/2025|EPI_ISL_19875928|2025-04-16                ACTCCGTGGAGGAGGTCTTATCAGAGGCACGTCAACATCTTAGAGATGGCACTTGTGGCT 369
hCoV-19/Ireland/D-BH-02351167/2025|EPI_ISL_19911337|2025-05-28            ACTCCGTGGAGGAGGTCTTATCAGAGGCACGTCAACATCTTAGAGATGGCACTTGTGGCT 368
hCoV-19/Ireland/D-NVRL-XIRL00001782/2025|EPI_ISL_19896104|2025-05-14      ACTCCGTGGAGGAGGTCTTATCAGAGGCACGTCAACATCTTAGAGATGGCACTTGTGGCT 368
hCoV-19/Ireland/TA-NVRL-GIRL00039444/2025|EPI_ISL_19896118|2025-05-16     ACTCCGTGGAGGAGGTCTTATCAGAGGCACGTCAACATCTTAGAGATGGCACTTGTGGCT 368
hCoV-19/Spain/GA-CHUAC-13223/2025|EPI_ISL_19894946|2025-05-31             ACTCCGTGGAGGAGGTCTTATCAGAGGCACGTCAACATCTTAGAGATGGCACTTGTGGCT 367
hCoV-19/Spain/GA-CHUAC-13215/2025|EPI_ISL_19894939|2025-05-29             ACTCCGTGGAGGAGGTCTTATCAGAGGCACGTCAACATCTTAGAGATGGCACTTGTGGCT 367
```

```
hCoV-19/Finland/THL-02398/2025|EPI_ISL_19875939|2025-05-04                ACTCCGTGGAGGAGGTCTTATCAGAGGCACGTCAACATCTTAGAGATGGCACTTGTGGCT 368
hCoV-19/Finland/THL-02400/2025|EPI_ISL_19875941|2025-05-03                ACTCCGTGGAGGAGGTCTTATCAGAGGCACGTCAACATCTTAGAGATGGCACTTGTGGCT 368
hCoV-19/Ireland/LK-UHL-702929/2025|EPI_ISL_19877895|2025-04-16            ACTCCGTGGAGGAGGTCTTATCAGAGGCACGTCAACATCTTAGAGATGGCACTTGTGGCT 368
hCoV-19/Ireland/LK-UHL-703788/2025|EPI_ISL_19877905|2025-05-03            ACTCCGTGGAGGAGGTCTTATCAGAGGCACGTCAACATCTTAGAGATGGCACTTGTGGCT 368
hCoV-19/Ireland/LK-UHL-703851/2025|EPI_ISL_19877907|2025-05-04            ACTCCGTGGAGGAGGTCTTATCAGAGGCACGTCAACATCTTAGAGATGGCACTTGTGGCT 368
hCoV-19/Ireland/LK-UHL-703889/2025|EPI_ISL_19877908|2025-05-05            ACTCCGTGGAGGAGGTCTTATCAGAGGCACGTCAACATCTTAGAGATGGCACTTGTGGCT 368
hCoV-19/Ireland/D-SVUH-FBB21492-49/2025|EPI_ISL_19881756|2025-05-14       ACTCCGTGGAGGAGGTCTTATCAGAGGCACGTCAACATCTTAGAGATGGCACTTGTGGCT 368
hCoV-19/Ireland/D-SVUH-FBB21492-59/2025|EPI_ISL_19881763|2025-05-07       ACTCCGTGGAGGAGGTCTTATCAGAGGCACGTCAACATCTTAGAGATGGCACTTGTGGCT 368
hCoV-19/Spain/CT-HUGTiP-G3R212/2025|EPI_ISL_19883055|2025-05-20           ACTCCGTGGAGGAGGTCTTATCAGAGGCACGTCAACATCTTAGAGATGGCACTTGTGGCT 368
hCoV-19/Ireland/LK-UHL-704398/2025|EPI_ISL_19888621|2025-05-16            ACTCCGTGGAGGAGGTCTTATCAGAGGCACGTCAACATCTTAGAGATGGCACTTGTGGCT 368
hCoV-19/Ireland/G-GUH-N3466/2025|EPI_ISL_19893175|2025-05-03              ACTCCGTGGAGGAGGTCTTATCAGAGGCACGTCAACATCTTAGAGATGGCACTTGTGGCT 368
hCoV-19/Belgium/Sciensano-LS-S5276/2025|EPI_ISL_19893834|2025-04-22       ACTCCGTGGAGGAGGTCTTATCAGAGGCACGTCAACATCTTAGAGATGGCACTTGTGGCT 368
hCoV-19/Italy/VEN-ULSS8-0019244_VI/2025|EPI_ISL_19899362|2025-05-26       ACTCCGTGGAGGAGGTCTTATCAGAGGCACGTCAACATCTTAGAGATGGCACTTGTGGCT 368
hCoV-19/Spain/CT-HUVH-E618109/2025|EPI_ISL_19907773|2025-06-10            ACTCCGTGGAGGAGGTCTTATCAGAGGCACGTCAACATCTTAGAGATGGCACTTGTGGCT 368
hCoV-19/Ireland/D-BH-03819149/2025|EPI_ISL_19911343|2025-05-29            ACTCCGTGGAGGAGGTCTTATCAGAGGCACGTCAACATCTTAGAGATGGCACTTGTGGCT 368
hCoV-19/Ireland/D-BH-03993161/2025|EPI_ISL_19911355|2025-06-10            ACTCCGTGGAGGAGGTCTTATCAGAGGCACGTCAACATCTTAGAGATGGCACTTGTGGCT 368
hCoV-19/Spain/CT-HUVH-E587040/2025|EPI_ISL_19871881|2025-04-26            ACTCCGTGGAGGAGGTCTTATCAGAGGCACGTCAACATCTTAGAGATGGCACTTGTGGCT 367
hCoV-19/Finland/THL-02392/2025|EPI_ISL_19875934|2025-05-02                ACTCCGTGGAGGAGGTCTTATCAGAGGCACGTCAACATCTTAGAGATGGCACTTGTGGCT 367
hCoV-19/Spain/GA-CHUAC-13181/2025|EPI_ISL_19890617|2025-05-20             ACTCCGTGGAGGAGGTCTTATCAGAGGCACGTCAACATCTTAGAGATGGCACTTGTGGCT 367
hCoV-19/Spain/CT-HUVH-E614843/2025|EPI_ISL_19907763|2025-06-05            ACTCCGTGGAGGAGGTCTTATCAGAGGCACGTCAACATCTTAGAGATGGCACTTGTGGCT 367
hCoV-19/Italy/UMB-LAOP-172-4-02/2025|EPI_ISL_19899474|2025-05-08          ACTCCGTGGAGGAGGTCTTATCAGAGGCACGTCAACATCTTAGAGATGGCACTTGTGGCT 389
hCoV-19/Spain/NC-CHN-01008932/2025|EPI_ISL_19900595|2025-04-04            ACTCCGTGGAGGAGGTCTTATCAGAGGCACGTCAACATCTTAGAGATGGCACTTGTGGCT 391
hCoV-19/Northern_Ireland/CLIMB-CM7YGDRO/2025|EPI_ISL_19877331|2025-04-14  ACTCCGTGGAGGAGGTCTTATCAGAGGCACGTCAACATCTTAGAGATGGCACTTGTGGCT 379
hCoV-19/Scotland/CLIMB-CM7YEE96/2025|EPI_ISL_19897754|2025-05-13          ACTCCGTGGAGGAGGTCTTATCAGAGGCACGTCAACATCTTAGAGATGGCACTTGTGGCT 378
hCoV-19/France/ARA-HCL125018289001/2025|EPI_ISL_19880005|2025-05-12       ACTCCGTGGAGGAGGTCTTATCAGAGGCACGTCAACATCTTAGAGATGGCACTTGTGGCT 372
hCoV-19/France/ARA-RELAB-HCL725000057501/2025|EPI_ISL_19880014|2025-04-28 ACTCCGTGGAGGAGGTCTTATCAGAGGCACGTCAACATCTTAGAGATGGCACTTGTGGCT 372
hCoV-19/France/ARA-RELAB-HCL725000059501/2025|EPI_ISL_19885160|2025-04-25 ACTCCGTGGAGGAGGTCTTATCAGAGGCACGTCAACATCTTAGAGATGGCACTTGTGGCT 372
hCoV-19/France/NAQ-RELAB-HCL725000061001/2025|EPI_ISL_19885161|2025-05-10 ACTCCGTGGAGGAGGTCTTATCAGAGGCACGTCAACATCTTAGAGATGGCACTTGTGGCT 372
hCoV-19/France/NAQ-RELAB-HCL725000060901/2025|EPI_ISL_19885180|2025-05-09 ACTCCGTGGAGGAGGTCTTATCAGAGGCACGTCAACATCTTAGAGATGGCACTTGTGGCT 372
hCoV-19/France/ARA-HCL025075546601/2025|EPI_ISL_19885182|2025-05-09       ACTCCGTGGAGGAGGTCTTATCAGAGGCACGTCAACATCTTAGAGATGGCACTTGTGGCT 372
hCoV-19/France/ARA-HCL125018461901/2025|EPI_ISL_19885204|2025-05-14       ACTCCGTGGAGGAGGTCTTATCAGAGGCACGTCAACATCTTAGAGATGGCACTTGTGGCT 372
hCoV-19/France/ARA-HCL125018655901/2025|EPI_ISL_19885206|2025-05-14       ACTCCGTGGAGGAGGTCTTATCAGAGGCACGTCAACATCTTAGAGATGGCACTTGTGGCT 372
hCoV-19/France/ARA-HCL125019155501/2025|EPI_ISL_19885209|2025-05-18       ACTCCGTGGAGGAGGTCTTATCAGAGGCACGTCAACATCTTAGAGATGGCACTTGTGGCT 372
hCoV-19/France/ARA-HCL125019240901/2025|EPI_ISL_19885210|2025-05-19       ACTCCGTGGAGGAGGTCTTATCAGAGGCACGTCAACATCTTAGAGATGGCACTTGTGGCT 372
hCoV-19/France/ARA-HCL125019249201/2025|EPI_ISL_19885211|2025-05-19       ACTCCGTGGAGGAGGTCTTATCAGAGGCACGTCAACATCTTAGAGATGGCACTTGTGGCT 372
hCoV-19/France/ARA-HCL125019399701/2025|EPI_ISL_19885212|2025-05-20       ACTCCGTGGAGGAGGTCTTATCAGAGGCACGTCAACATCTTAGAGATGGCACTTGTGGCT 372
hCoV-19/France/ARA-HCL125019408901/2025|EPI_ISL_19885213|2025-05-20       ACTCCGTGGAGGAGGTCTTATCAGAGGCACGTCAACATCTTAGAGATGGCACTTGTGGCT 372
hCoV-19/France/OCC-RELAB-HCL725000064001/2025|EPI_ISL_19895004|2025-05-14 ACTCCGTGGAGGAGGTCTTATCAGAGGCACGTCAACATCTTAGAGATGGCACTTGTGGCT 372
hCoV-19/France/OCC-RELAB-HCL725000064101/2025|EPI_ISL_19895005|2025-05-14 ACTCCGTGGAGGAGGTCTTATCAGAGGCACGTCAACATCTTAGAGATGGCACTTGTGGCT 372
hCoV-19/France/NAQ-RELAB-HCL725000062601/2025|EPI_ISL_19895009|2025-05-16 ACTCCGTGGAGGAGGTCTTATCAGAGGCACGTCAACATCTTAGAGATGGCACTTGTGGCT 372
hCoV-19/France/ARA-RELAB-HCL725000062401/2025|EPI_ISL_19895017|2025-05-16 ACTCCGTGGAGGAGGTCTTATCAGAGGCACGTCAACATCTTAGAGATGGCACTTGTGGCT 372
hCoV-19/France/ARA-RELAB-HCL725000062101/2025|EPI_ISL_19895018|2025-05-13 ACTCCGTGGAGGAGGTCTTATCAGAGGCACGTCAACATCTTAGAGATGGCACTTGTGGCT 372
hCoV-19/France/ARA-HCL125019625101/2025|EPI_ISL_19895034|2025-05-21       ACTCCGTGGAGGAGGTCTTATCAGAGGCACGTCAACATCTTAGAGATGGCACTTGTGGCT 372
hCoV-19/France/ARA-HCL125019754801/2025|EPI_ISL_19895035|2025-05-22       ACTCCGTGGAGGAGGTCTTATCAGAGGCACGTCAACATCTTAGAGATGGCACTTGTGGCT 372
hCoV-19/France/ARA-RELAB-HCL725000064601/2025|EPI_ISL_19895041|2025-05-15 ACTCCGTGGAGGAGGTCTTATCAGAGGCACGTCAACATCTTAGAGATGGCACTTGTGGCT 372
hCoV-19/France/ARA-RELAB-HCL725000066001/2025|EPI_ISL_19895046|2025-05-13 ACTCCGTGGAGGAGGTCTTATCAGAGGCACGTCAACATCTTAGAGATGGCACTTGTGGCT 372
hCoV-19/France/OCC-RELAB-HCL725000068701/2025|EPI_ISL_19905466|2025-05-21 ACTCCGTGGAGGAGGTCTTATCAGAGGCACGTCAACATCTTAGAGATGGCACTTGTGGCT 372
hCoV-19/France/OCC-RELAB-HCL725000069001/2025|EPI_ISL_19905468|2025-05-21 ACTCCGTGGAGGAGGTCTTATCAGAGGCACGTCAACATCTTAGAGATGGCACTTGTGGCT 372
hCoV-19/France/ARA-HCL025089279801/2025|EPI_ISL_19905469|2025-06-02       ACTCCGTGGAGGAGGTCTTATCAGAGGCACGTCAACATCTTAGAGATGGCACTTGTGGCT 372
hCoV-19/France/PAC-RELAB-HCL725000069901/2025|EPI_ISL_19908847|2025-05-26 ACTCCGTGGAGGAGGTCTTATCAGAGGCACGTCAACATCTTAGAGATGGCACTTGTGGCT 372
hCoV-19/France/PAC-RELAB-HCL725000069501/2025|EPI_ISL_19908849|2025-05-30 ACTCCGTGGAGGAGGTCTTATCAGAGGCACGTCAACATCTTAGAGATGGCACTTGTGGCT 372
hCoV-19/France/ARA-RELAB-HCL725000071701/2025|EPI_ISL_19908858|2025-05-22 ACTCCGTGGAGGAGGTCTTATCAGAGGCACGTCAACATCTTAGAGATGGCACTTGTGGCT 372
hCoV-19/France/ARA-HCL025093352901/2025|EPI_ISL_19908867|2025-06-10       ACTCCGTGGAGGAGGTCTTATCAGAGGCACGTCAACATCTTAGAGATGGCACTTGTGGCT 372
```

```
hCoV-19/France/ARA-RELAB-HCL725000065801/2025|EPI_ISL_19895045|2025-05-16      ACTCCGTGGAGGAGGTCTTATCAGAGGCACGTCAACATCTTAGAGATGGCACTTGTGGCT 372
hCoV-19/Denmark/DCGC-692234/2025|EPI_ISL_19879694|2025-05-05                   ACTCCGTGGAGGAGGTCTTATCAGAGGCACGTCAACATCTTAGAGATGGCACTTGTGGCT 374
hCoV-19/Finland/THL-02388/2025|EPI_ISL_19875931|2025-04-16                     ACTCCGTGGAGGAGGTCTTATCAGAGGCACGTCAACATCTTAGAGATGGCACTTGTGGCT 375
hCoV-19/Spain/PV-HUD-98024244/2025|EPI_ISL_19907759|2025-06-09                 ACTCCGTGGAGGAGGTCTTATCAGAGGCACGTCAACATCTTAGAGATGGCACTTGTGGCT 375
hCoV-19/Netherlands/GE-RIVM-145386/2025|EPI_ISL_19873764|2025-05-01            ACTCCGTGGAGGAGGTCTTATCAGAGGCACGTCAACATCTTAGAGATGGCACTTGTGGCT 372
hCoV-19/Netherlands/ZH-RIVM-145413/2025|EPI_ISL_19891397|2025-04-30            ACTCCGTGGAGGAGGTCTTATCAGAGGCACGTCAACATCTTAGAGATGGCACTTGTGGCT 372
hCoV-19/Netherlands/NH-RIVM-145458/2025|EPI_ISL_19905062|2025-05-25            ACTCCGTGGAGGAGGTCTTATCAGAGGCACGTCAACATCTTAGAGATGGCACTTGTGGCT 372
hCoV-19/Netherlands/NH-RIVM-145463/2025|EPI_ISL_19905063|2025-05-20            ACTCCGTGGAGGAGGTCTTATCAGAGGCACGTCAACATCTTAGAGATGGCACTTGTGGCT 372
hCoV-19/Netherlands/UT-RIVM-145436/2025|EPI_ISL_19905072|2025-05-15            ACTCCGTGGAGGAGGTCTTATCAGAGGCACGTCAACATCTTAGAGATGGCACTTGTGGCT 372
hCoV-19/Netherlands/UT-RIVM-145470/2025|EPI_ISL_19905077|2025-06-02            ACTCCGTGGAGGAGGTCTTATCAGAGGCACGTCAACATCTTAGAGATGGCACTTGTGGCT 372
hCoV-19/Netherlands/ZH-RIVM-145478/2025|EPI_ISL_19905094|2025-05-30            ACTCCGTGGAGGAGGTCTTATCAGAGGCACGTCAACATCTTAGAGATGGCACTTGTGGCT 372
hCoV-19/Netherlands/ZH-RIVM-145480/2025|EPI_ISL_19905096|2025-05-31            ACTCCGTGGAGGAGGTCTTATCAGAGGCACGTCAACATCTTAGAGATGGCACTTGTGGCT 372
hCoV-19/Netherlands/UT-RIVM-145481/2025|EPI_ISL_19905078|2025-05-31            ACTCCGTGGAGGAGGTCTTATCAGAGGCACGTCAACATCTTAGAGATGGCACTTGTGGCT 372
hCoV-19/Scotland/CLIMB-CM7YRW9H/2025|EPI_ISL_19881711|2025-05-06               ACTCCGTGGAGGAGGTCTTATCAGAGGCACGTCAACATCTTAGAGATGGCACTTGTGGCT 344
hCoV-19/France/IDF-RELAB-IPP04872/2025|EPI_ISL_19911293|2025-05-22             ACTCCGTGGAGGAGGTCTTATCAGAGGCACGTCAACATCTTAGAGATGGCACTTGTGGCT 340
hCoV-19/Canary_Islands/CN-HUC-925112913/2025|EPI_ISL_19900668|2025-04-22       ACTCCGTGGAGGAGGTCTTATCAGAGGCACGTCAACATCTTAGAGATGGCACTTGTGGCT 348
hCoV-19/Scotland/CLIMB-CM7YJ5BB/2025|EPI_ISL_19906458|2025-05-27               ACTCCGTGGAGGAGGTCTTATCAGAGGCACGTCAACATCTTAGAGATGGCACTTGTGGCT 344
hCoV-19/Scotland/CLIMB-CM7Y8PHH/2025|EPI_ISL_19897755|2025-05-14               ACTCCGTGGAGGAGGTCTTATCAGAGGCACGTCAACATCTTAGAGATGGCACTTGTGGCT 344
hCoV-19/Spain/AN-PMC-42706/2025|EPI_ISL_19888325|2025-05-25                    ACTCCGTGGAGGAGGTCTTATCAGAGGCACGTCAACATCTTAGAGATGGCACTTGTGGCT 344
hCoV-19/Scotland/CLIMB-CM7YRIA1/2025|EPI_ISL_19869961|2025-04-20               ACTCCGTGGAGGAGGTCTTATCAGAGGCACGTCAACATCTTAGAGATGGCACTTGTGGCT 344
hCoV-19/Scotland/CLIMB-CM7Y8UWT/2025|EPI_ISL_19869966|2025-04-21               ACTCCGTGGAGGAGGTCTTATCAGAGGCACGTCAACATCTTAGAGATGGCACTTGTGGCT 344
hCoV-19/England/CLIMB-CM7YMFHB/2025|EPI_ISL_19877436|2025-05-10                ACTCCGTGGAGGAGGTCTTATCAGAGGCACGTCAACATCTTAGAGATGGCACTTGTGGCT 344
hCoV-19/Denmark/DCGC-692213/2025|EPI_ISL_19879673|2025-04-28                   ACTCCGTGGAGGAGGTCTTATCAGAGGCACGTCAACATCTTAGAGATGGCACTTGTGGCT 344
hCoV-19/Wales/CLIMB-CM7YRAXQ/2025|EPI_ISL_19883075|2025-05-14                  ACTCCGTGGAGGAGGTCTTATCAGAGGCACGTCAACATCTTAGAGATGGCACTTGTGGCT 344
hCoV-19/Spain/AN-PMC-42696/2025|EPI_ISL_19888316|2025-05-14                    ACTCCGTGGAGGAGGTCTTATCAGAGGCACGTCAACATCTTAGAGATGGCACTTGTGGCT 344
hCoV-19/Spain/AN-PMC-42719/2025|EPI_ISL_19888338|2025-05-15                    ACTCCGTGGAGGAGGTCTTATCAGAGGCACGTCAACATCTTAGAGATGGCACTTGTGGCT 344
hCoV-19/England/CLIMB-CM7YE7HB/2025|EPI_ISL_19892115|2025-05-19                ACTCCGTGGAGGAGGTCTTATCAGAGGCACGTCAACATCTTAGAGATGGCACTTGTGGCT 344
hCoV-19/France/GES-RELAB-IPP04599/2025|EPI_ISL_19893547|2025-05-05             ACTCCGTGGAGGAGGTCTTATCAGAGGCACGTCAACATCTTAGAGATGGCACTTGTGGCT 344
hCoV-19/Scotland/CLIMB-CM7YEPXZ/2025|EPI_ISL_19896028|2025-05-25               ACTCCGTGGAGGAGGTCTTATCAGAGGCACGTCAACATCTTAGAGATGGCACTTGTGGCT 344
hCoV-19/Scotland/CLIMB-CM7YF4QT/2025|EPI_ISL_19896032|2025-05-27               ACTCCGTGGAGGAGGTCTTATCAGAGGCACGTCAACATCTTAGAGATGGCACTTGTGGCT 344
hCoV-19/Scotland/CLIMB-CM7YKDZ6/2025|EPI_ISL_19896033|2025-05-27               ACTCCGTGGAGGAGGTCTTATCAGAGGCACGTCAACATCTTAGAGATGGCACTTGTGGCT 344
hCoV-19/Scotland/CLIMB-CM7YG8H4/2025|EPI_ISL_19897748|2025-05-02               ACTCCGTGGAGGAGGTCTTATCAGAGGCACGTCAACATCTTAGAGATGGCACTTGTGGCT 344
hCoV-19/Wales/CLIMB-CM7YJXNZ/2025|EPI_ISL_19900036|2025-05-26                  ACTCCGTGGAGGAGGTCTTATCAGAGGCACGTCAACATCTTAGAGATGGCACTTGTGGCT 344
hCoV-19/Germany/NW-RKI-I-1148178/2025|EPI_ISL_19902055|2025-05-21              ACTCCGTGGAGGAGGTCTTATCAGAGGCACGTCAACATCTTAGAGATGGCACTTGTGGCT 344
hCoV-19/Germany/NI-RKI-I-1148183/2025|EPI_ISL_19902060|2025-05-18              ACTCCGTGGAGGAGGTCTTATCAGAGGCACGTCAACATCTTAGAGATGGCACTTGTGGCT 344
hCoV-19/Germany/NW-RKI-I-1148191/2025|EPI_ISL_19902068|2025-05-19              ACTCCGTGGAGGAGGTCTTATCAGAGGCACGTCAACATCTTAGAGATGGCACTTGTGGCT 344
hCoV-19/Spain/MD-HGUGM-66414184/2025|EPI_ISL_19906298|2025-05-25               ACTCCGTGGAGGAGGTCTTATCAGAGGCACGTCAACATCTTAGAGATGGCACTTGTGGCT 344
hCoV-19/Spain/MD-HGUGM-66400167/2025|EPI_ISL_19906320|2025-05-22               ACTCCGTGGAGGAGGTCTTATCAGAGGCACGTCAACATCTTAGAGATGGCACTTGTGGCT 344
hCoV-19/France/IDF-RELAB-IPP04646/2025|EPI_ISL_19907194|2025-05-16             ACTCCGTGGAGGAGGTCTTATCAGAGGCACGTCAACATCTTAGAGATGGCACTTGTGGCT 344
hCoV-19/England/CLIMB-CM7YKIUQ/2025|EPI_ISL_19908573|2025-06-07                ACTCCGTGGAGGAGGTCTTATCAGAGGCACGTCAACATCTTAGAGATGGCACTTGTGGCT 344
hCoV-19/France/GES-RELAB-IPP04823/2025|EPI_ISL_19911257|2025-05-26             ACTCCGTGGAGGAGGTCTTATCAGAGGCACGTCAACATCTTAGAGATGGCACTTGTGGCT 344
hCoV-19/France/IDF-RELAB-IPP04863/2025|EPI_ISL_19911265|2025-05-24             ACTCCGTGGAGGAGGTCTTATCAGAGGCACGTCAACATCTTAGAGATGGCACTTGTGGCT 344
hCoV-19/Denmark/DCGC-692245/2025|EPI_ISL_19879704|2025-04-28                   ACTCCGTGGAGGAGGTCTTATCAGAGGCACGTCAACATCTTAGAGATGGCACTTGTGGCT 344


hCoV-19/Ireland/D-SVUH-FBA98650-31/2025|EPI_ISL_19866548|2025-05-04            ------------------------------------AACAGCCCTATGTGTTCATCAAAC 24
hCoV-19/Spain/PV-HUB-5628663/2025|EPI_ISL_19908839|2025-05-16                  TAGTAGAAGTTGAAAAAGGCGTTTTGCCTCAACTTGAACAGCCCTATGTGTTCATCAAAC 277
hCoV-19/Spain/PV-HUB-41587498/2025|EPI_ISL_19908930|2025-05-20                 TAGTAGAAGTTGAAAAAGGCGTTTTGCCTCAACTTGAACAGCCCTATGTGTTCATCAAAC 272
hCoV-19/Spain/PV-HUB-56291851/2025|EPI_ISL_19908970|2025-05-31                 TAGTAGAAGTTGAAAAAGGCGTTTTGCCTCAACTTGAACAGCCCTATGTGTTCATCAAAC 273
hCoV-19/Northern_Ireland/CLIMB-CM7YR8I6/2025|EPI_ISL_19877334|2025-04-23       NNNNNNNNNTTGAAAAAGGCGTTTTGCCTCAACTTGAANNNCCCTATGTNNNNNNNNNNN 381
hCoV-19/Germany/NW-RKI-I-1148136/2025|EPI_ISL_19871950|2025-04-23              NNNNNNNNNNNNNNNNNNNNNNNNNNNNNNNNNNNNNNNNNNNN-----NNNNNNNNNNN 399
hCoV-19/Germany/NW-RKI-I-1148141/2025|EPI_ISL_19871955|2025-04-23              NNNNNNNNNNNNNNNNNNNNNNNNNNNNNNNNNNNNNNNNNNNN-----NNNNNNNNNNN 399
hCoV-19/Germany/BW-RKI-I-1148164/2025|EPI_ISL_19884987|2025-04-17              NNNNNNNNNNNNNNNNNNNNNNNNNNNNNNNNNNNNNNNNNNNN-----NNNNNNNNNNN 399
```

```
hCoV-19/Scotland/CLIMB-CM7YFGW7/2025|EPI_ISL_19897691|2025-05-24          NNNNNNNNNNNNNNNNNNNNNNNNNNNNNNNNNNNNNNNNNCCCTATGTGTTCATCAAAC 404
hCoV-19/Ireland/G-LUH-N3473/2025|EPI_ISL_19893181|2025-05-14              TAGTAGAAGTTGAAAAAGGCGTTTTGCCTCAACTTGAACAGCCCTATGTGTTCATCAAAC 471
hCoV-19/Spain/AN-PMC-42728/2025|EPI_ISL_19888347|2025-05-25               TAGTAGAAGTTGAAAAAGGCGTTTTGCCTCAACTTGAACAGCCCTATGTGTTCATCAAAC 472
hCoV-19/Sweden/000200866492N2/2025|EPI_ISL_19870050|2025-04-22            TAGTAGAAGTTGAAAAAGGCGTTTTGCCTCAACTTGAACAGCCCTATGTGTTCATCAAAC 478
hCoV-19/Sweden/000200867159N2/2025|EPI_ISL_19870052|2025-04-23            TAGTAGAAGTTGAAAAAGGCGTTTTGCCTCAACTTGAACAGCCCTATGTGTTCATCAAAC 478
hCoV-19/Ireland/G-LUH-N3445/2025|EPI_ISL_19893154|2025-05-07              TAGTAGAAGTTGAAAAAGGCGTTTTGCCTCAACTTGAACAGCCCTATGTGTTCATCAAAC 478
hCoV-19/Ireland/G-LUH-N3443/2025|EPI_ISL_19893152|2025-05-07              TAGTAGAAGTTGAAAAAGGCGTTTTGCCTCAACTTGAACAGCCCTATGTGTTCATCAAAC 476
hCoV-19/Scotland/CLIMB-CM7YF9PJ/2025|EPI_ISL_19895983|2025-05-19          TAGTAGAAGTTGAAAAAGGCGTTTTGCCTCAACTTGAACAGCCCTATGTGTTCATCAAAC 475
hCoV-19/Scotland/CLIMB-CM7YKMK8/2025|EPI_ISL_19869976|2025-04-25          TAGTAGAAGTTGAAAAAGGCGTTTTGCCTCAACTTGAACAGCCCTATGTGTTCATCAAAC 474
hCoV-19/Ireland/G-LUH-N3444/2025|EPI_ISL_19893153|2025-05-07              TAGTAGAAGTTGAAAAAGGCGTTTTGCCTCAACTTGAACAGCCCTATGTGTTCATCAAAC 474
hCoV-19/Switzerland/VD-CHUV-8604552682/2025|EPI_ISL_19906527|2025-05-19   TAGTAGAAGTTGAAAAAGGCGTTTTGCCTCAACTTGAACAGCCCTATGTGTTCATCAAAC 479
hCoV-19/Scotland/CLIMB-CM7YR3YO/2025|EPI_ISL_19896015|2025-05-24          TAGTAGAAGTTGAAAAAGGCGTTTTGCCTCAACTTGAACAGCCCTATGTGTTCATCAAAC 480
hCoV-19/Sweden/O-18_SE100_25CS500180/2025|EPI_ISL_19866288|2025-04-15     TAGTAGAAGTTGAAAAAGGCGTTTTGCCTCAACTTGAACAGCCCTATGTGTTCATCAAAC 480
hCoV-19/Ireland/D-CS250088/2025|EPI_ISL_19877457|2025-04-16               TAGTAGAAGTTGAAAAAGGCGTTTTGCCTCAACTTGAACAGCCCTATGTGTTCATCAAAC 480
hCoV-19/Ireland/G-LUH-N3442/2025|EPI_ISL_19893151|2025-05-07              TAGTAGAAGTTGAAAAAGGCGTTTTGCCTCAACTTGAACAGCCCTATGTGTTCATCAAAC 480
hCoV-19/Scotland/CLIMB-CM7YMZ5M/2025|EPI_ISL_19906439|2025-05-22          TAGTAGAAGTTGAAAAAGGCGTTTTGCCTCAACTTGAACAGCCCTATGTGTTCATCAAAC 479
hCoV-19/Scotland/CLIMB-CM7YR3ZG/2025|EPI_ISL_19897722|2025-05-13          TAGTAGAAGTTGAAAAAGGCGTTTTGCCTCAACTTGAACAGCCCTATGTGTTCATCAAAC 478
hCoV-19/Scotland/CLIMB-CM7YEYGT/2025|EPI_ISL_19897741|2025-04-28          TAGTAGAAGTTGAAAAAGGCGTTTTGCCTCAACTTGAACAGCCCTATGTGTTCATCAAAC 478
hCoV-19/Scotland/CLIMB-CM7YGAJ3/2025|EPI_ISL_19895963|2025-05-13          TAGTAGAAGTTGAAAAAGGCGTTTTGCCTCAACTTGAACAGCCCTATGTGTTCATCAAAC 467
hCoV-19/Scotland/CLIMB-CM7YJ1ZO/2025|EPI_ISL_19897757|2025-05-14          TAGTAGAAGTTGAAAAAGGCGTTTTGCCTCAACTTGAACAGCCCTATGTGTTCATCAAAC 468
hCoV-19/Scotland/CLIMB-CM7Y8C4Y/2025|EPI_ISL_19906404|2025-05-15          TAGTAGAAGTTGAAAAAGGCGTTTTGCCTCAACTTGAACAGCCCTATGTGTTCATCAAAC 475
hCoV-19/Northern_Ireland/CLIMB-CM7YGGYM/2025|EPI_ISL_19897641|2025-05-23  TAGTAGAAGTTGAAAAAGGCGTTTTGCCTCAACTTGAACAGCCCTATGTGTTCATCAAAC 470
hCoV-19/Scotland/CLIMB-CM7YFW7M/2025|EPI_ISL_19881718|2025-05-10          TAGTAGAAGTTGAAAAAGGCGTTTTGCCTCAACTTGAACAGCCCTATGTGTTCATCAAAC 473
hCoV-19/Scotland/CLIMB-CM7YEMXP/2025|EPI_ISL_19888387|2025-05-10          TAGTAGAAGTTGAAAAAGGCGTTTTGCCTCAACTTGAACAGCCCTATGTGTTCATCAAAC 472
hCoV-19/Spain/CT-HUVH-E593511/2025|EPI_ISL_19881731|2025-05-06            TAGTAGAAGTTGAAAAAGGCGTTTTGCCTCAACTTGAACAGCCCTATGTGTTCATCAAAC 425
hCoV-19/Spain/CT-HUJT-E325362/2025|EPI_ISL_19889935|2025-05-20            TAGTAGAAGTTGAAAAAGGCGTTTTGCCTCAANNNNNNNNNNNNNNNNNNNNCANNNNNN 426
hCoV-19/Spain/PV-HUD-84743204/2025|EPI_ISL_19907746|2025-06-06            TAGTAGAAGTTGAAAAAGGCGTTTTGCCTCAACTTGAACAGCCCTATGTGTTCATCAAAC 427
hCoV-19/Ireland/D-SVUH-FBB21492-54/2025|EPI_ISL_19881758|2025-05-11       TAGTAGAAGTTGAAAAAGGCGTTTTGCCTCAACTTGAACAGCCCTATGTGTTCATCAAAC 428
hCoV-19/Spain/CT-HUVH-E596725/2025|EPI_ISL_19881729|2025-05-10            TAGTAGAAGTTGAAAAAGGCGTTTTGCCTCAACTTGAACAGCCCTATGTGTTCATCAAAC 422
hCoV-19/Spain/CT-HUVH-E593882/2025|EPI_ISL_19881732|2025-05-06            TAGTAGAAGTTGAAAAAGGCGTTTTGCCTCAACTTGAACAGCCCTATGTGTTCATCAAAC 422
hCoV-19/Finland/THL-02385/2025|EPI_ISL_19875928|2025-04-16                TAGTAGAAGTTGAAAAAGGCGTTTTGCCTCAACTTGAACAGCCCTATGTGTTCATCAAAC 429
hCoV-19/Ireland/D-BH-02351167/2025|EPI_ISL_19911337|2025-05-28            TAGTAGAAGTTGAAAAAGGCGTTTTGCCTCAACTTGAACAGCCCTATGTGTTCATCAAAC 428
hCoV-19/Ireland/D-NVRL-XIRL00001782/2025|EPI_ISL_19896104|2025-05-14      TAGTAGAAGTTGAAAAAGGCGTTTTGCCTCAACTTGAACAGCCCTATGTGTTCATCAAAC 428
hCoV-19/Ireland/TA-NVRL-GIRL00039444/2025|EPI_ISL_19896118|2025-05-16     TAGTAGAAGTTGAAAAAGGCGTTTTGCCTCAACTTGAACAGCCCTATGTGTTCATCAAAC 428
hCoV-19/Spain/GA-CHUAC-13223/2025|EPI_ISL_19894946|2025-05-31             TAGTAGAAGTTGAAAAAGGCGTTTTGCCTCAACTTGAACAGCCCTATGTGTTCATCAAAC 427
hCoV-19/Spain/GA-CHUAC-13215/2025|EPI_ISL_19894939|2025-05-29             TAGTAGAAGTTGAAAAAGGCGTTTTGCCTCAACTTGAACAGCCCTATGTGTTCATCAAAC 427
hCoV-19/Finland/THL-02398/2025|EPI_ISL_19875939|2025-05-04                TAGTAGAAGTTGAAAAAGGCGTTTTGCCTCAACTTGAACAGCCCTATGTGTTCATCAAAC 428
hCoV-19/Finland/THL-02400/2025|EPI_ISL_19875941|2025-05-03                TAGTAGAAGTTGAAAAAGGCGTTTTGCCTCAACTTGAACAGCCCTATGTGTTCATCAAAC 428
hCoV-19/Ireland/LK-UHL-702929/2025|EPI_ISL_19877895|2025-04-16            TAGTAGAAGTTGAAAAAGGCGTTTTGCCTCAACTTGAACAGCCCTATGTGTTCATCAAAC 428
hCoV-19/Ireland/LK-UHL-703788/2025|EPI_ISL_19877905|2025-05-03            TAGTAGAAGTTGAAAAAGGCGTTTTGCCTCAACTTGAACAGCCCTATGTGTTCATCAAAC 428
hCoV-19/Ireland/LK-UHL-703851/2025|EPI_ISL_19877907|2025-05-04            TAGTAGAAGTTGAAAAAGGCGTTTTGCCTCAACTTGAACAGCCCTATGTGTTCATCAAAC 428
hCoV-19/Ireland/LK-UHL-703889/2025|EPI_ISL_19877908|2025-05-05            TAGTAGAAGTTGAAAAAGGCGTTTTGCCTCAACTTGAACAGCCCTATGTGTTCATCAAAC 428
hCoV-19/Ireland/D-SVUH-FBB21492-49/2025|EPI_ISL_19881756|2025-05-14       TAGTAGAAGTTGAAAAAGGCGTTTTGCCTCAACTTGAACAGCCCTATGTGTTCATCAAAC 428
hCoV-19/Ireland/D-SVUH-FBB21492-59/2025|EPI_ISL_19881763|2025-05-07       TAGTAGAAGTTGAAAAAGGCGTTTTGCCTCAACTTGAACAGCCCTATGTGTTCATCAAAC 428
hCoV-19/Spain/CT-HUGTiP-G3R212/2025|EPI_ISL_19883055|2025-05-20           TAGTAGAAGTTGAAAAAGGCGTTTTGCCTCAACTTGAACAGCCCTATGTGTTCATCAAAC 428
hCoV-19/Ireland/LK-UHL-704398/2025|EPI_ISL_19888621|2025-05-16            TAGTAGAAGTTGAAAAAGGCGTTTTGCCTCAACTTGAACAGCCCTATGTGTTCATCAAAC 428
hCoV-19/Ireland/G-GUH-N3466/2025|EPI_ISL_19893175|2025-05-03              TAGTAGAAGTTGAAAAAGGCGTTTTGCCTCAACTTGAACAGCCCTATGTGTTCATCAAAC 428
hCoV-19/Belgium/Sciensano-LS-S5276/2025|EPI_ISL_19893834|2025-04-22       TAGTAGAAGTTGAAAAAGGCGTTTTGCCTCAACTTGAACAGCCCTATGTGTTCATCAAAC 428
hCoV-19/Italy/VEN-ULSS8-0019244_VI/2025|EPI_ISL_19899362|2025-05-26       TAGTAGAAGTTGAAAAAGGCGTTTTGCCTCAACTTGAACAGCCCTATGTGTTCATCAAAC 428
hCoV-19/Spain/CT-HUVH-E618109/2025|EPI_ISL_19907773|2025-06-10            TAGTAGAAGTTGAAAAAGGCGTTTTGCCTCAACTTGAACAGCCCTATGTGTTCATCAAAC 428
hCoV-19/Ireland/D-BH-03819149/2025|EPI_ISL_19911343|2025-05-29            TAGTAGAAGTTGAAAAAGGCGTTTTGCCTCAACTTGAACAGCCCTATGTGTTCATCAAAC 428
hCoV-19/Ireland/D-BH-03993161/2025|EPI_ISL_19911355|2025-06-10            TAGTAGAAGTTGAAAAAGGCGTTTTGCCTCAACTTGAACAGCCCTATGTGTTCATCAAAC 428
hCoV-19/Spain/CT-HUVH-E587040/2025|EPI_ISL_19871881|2025-04-26            TAGTAGAAGTTGAAAAAGGCGTTTTGCCTCAACTTGAACAGCCCTATGTGTTCATCAAAC 427
```

```
hCoV-19/Finland/THL-02392/2025|EPI_ISL_19875934|2025-05-02                   TAGTAGAAGTTGAAAAAGGCGTTTTGCCTCAACTTGAACAGCCCTATGTGTTCATCAAAC 427
hCoV-19/Spain/GA-CHUAC-13181/2025|EPI_ISL_19890617|2025-05-20                TAGTAGAAGTTGAAAAAGGCGTTTTGCCTCAACTTGAACAGCCCTATGTGTTCATCAAAC 427
hCoV-19/Spain/CT-HUVH-E614843/2025|EPI_ISL_19907763|2025-06-05               TAGTAGAAGTTGAAAAAGGCGTTTTGCCTCAACTTGAACAGCCCTATGTGTTCATCAAAC 427
hCoV-19/Italy/UMB-LAOP-172-4-02/2025|EPI_ISL_19899474|2025-05-08             TAGTAGAAGTTGAAAAAGGCGTTTTGCCTCAACTTGAACAGCCCTATGTGTTCATCAAAC 449
hCoV-19/Spain/NC-CHN-01008932/2025|EPI_ISL_19900595|2025-04-04               TAGTAGAAGTTGAAAAAGGCGTTTTGCCTCAACTTGAACAGCCCTATGTGTTCATCAAAC 451
hCoV-19/Northern_Ireland/CLIMB-CM7YGDRO/2025|EPI_ISL_19877331|2025-04-14     TAGTAGAAGTTGAAAAAGGCGTTTTGCCTCAACTTGAACAGCCCTATGTGTTCATCAAAC 439
hCoV-19/Scotland/CLIMB-CM7YEE96/2025|EPI_ISL_19897754|2025-05-13             TAGTAGAAGTTGAAAAAGGCGTTTTGCCTCAACTTGAACAGCCCTATGTGTTCATCAAAC 438
hCoV-19/France/ARA-HCL125018289001/2025|EPI_ISL_19880005|2025-05-12          TAGTAGAAGTTGAAAAAGGCGTTTTGCCTCAACTTGAACAGCCCTATGTGTTCATCAAAC 432
hCoV-19/France/ARA-RELAB-HCL725000057501/2025|EPI_ISL_19880014|2025-04-28    TAGTAGAAGTTGAAAAAGGCGTTTTGCCTCAACTTGAACAGCCCTATGTGTTCATCAAAC 432
hCoV-19/France/ARA-RELAB-HCL725000059501/2025|EPI_ISL_19885160|2025-04-25    TAGTAGAAGTTGAAAAAGGCGTTTTGCCTCAACTTGAACAGCCCTATGTGTTCATCAAAC 432
hCoV-19/France/NAQ-RELAB-HCL725000061001/2025|EPI_ISL_19885161|2025-05-10    TAGTAGAAGTTGAAAAAGGCGTTTTGCCTCAACTTGAACAGCCCTATGTGTTCATCAAAC 432
hCoV-19/France/NAQ-RELAB-HCL725000060901/2025|EPI_ISL_19885180|2025-05-09    TAGTAGAAGTTGAAAAAGGCGTTTTGCCTCAACTTGAACAGCCCTATGTGTTCATCAAAC 432
hCoV-19/France/ARA-HCL025075546601/2025|EPI_ISL_19885182|2025-05-09          TAGTAGAAGTTGAAAAAGGCGTTTTGCCTCAACTTGAACAGCCCTATGTGTTCATCAAAC 432
hCoV-19/France/ARA-HCL125018461901/2025|EPI_ISL_19885204|2025-05-14          TAGTAGAAGTTGAAAAAGGCGTTTTGCCTCAACTTGAACAGCCCTATGTGTTCATCAAAC 432
hCoV-19/France/ARA-HCL125018655901/2025|EPI_ISL_19885206|2025-05-14          TAGTAGAAGTTGAAAAAGGCGTTTTGCCTCAACTTGAACAGCCCTATGTGTTCATCAAAC 432
hCoV-19/France/ARA-HCL125019155501/2025|EPI_ISL_19885209|2025-05-18          TAGTAGAAGTTGAAAAAGGCGTTTTGCCTCAACTTGAACAGCCCTATGTGTTCATCAAAC 432
hCoV-19/France/ARA-HCL125019240901/2025|EPI_ISL_19885210|2025-05-19          TAGTAGAAGTTGAAAAAGGCGTTTTGCCTCAACTTGAACAGCCCTATGTGTTCATCAAAC 432
hCoV-19/France/ARA-HCL125019249201/2025|EPI_ISL_19885211|2025-05-19          TAGTAGAAGTTGAAAAAGGCGTTTTGCCTCAACTTGAACAGCCCTATGTGTTCATCAAAC 432
hCoV-19/France/ARA-HCL125019399701/2025|EPI_ISL_19885212|2025-05-20          TAGTAGAAGTTGAAAAAGGCGTTTTGCCTCAACTTGAACAGCCCTATGTGTTCATCAAAC 432
hCoV-19/France/ARA-HCL125019408901/2025|EPI_ISL_19885213|2025-05-20          TAGTAGAAGTTGAAAAAGGCGTTTTGCCTCAACTTGAACAGCCCTATGTGTTCATCAAAC 432
hCoV-19/France/OCC-RELAB-HCL725000064001/2025|EPI_ISL_19895004|2025-05-14    TAGTAGAAGTTGAAAAAGGCGTTTTGCCTCAACTTGAACAGCCCTATGTGTTCATCAAAC 432
hCoV-19/France/OCC-RELAB-HCL725000064101/2025|EPI_ISL_19895005|2025-05-14    TAGTAGAAGTTGAAAAAGGCGTTTTGCCTCAACTTGAACAGCCCTATGTGTTCATCAAAC 432
hCoV-19/France/NAQ-RELAB-HCL725000062601/2025|EPI_ISL_19895009|2025-05-16    TAGTAGAAGTTGAAAAAGGCGTTTTGCCTCAACTTGAACAGCCCTATGTGTTCATCAAAC 432
hCoV-19/France/ARA-RELAB-HCL725000062401/2025|EPI_ISL_19895017|2025-05-16    TAGTAGAAGTTGAAAAAGGCGTTTTGCCTCAACTTGAACAGCCCTATGTGTTCATCAAAC 432
hCoV-19/France/ARA-RELAB-HCL725000062101/2025|EPI_ISL_19895018|2025-05-13    TAGTAGAAGTTGAAAAAGGCGTTTTGCCTCAACTTGAACAGCCCTATGTGTTCATCAAAC 432
hCoV-19/France/ARA-HCL125019625101/2025|EPI_ISL_19895034|2025-05-21          TAGTAGAAGTTGAAAAAGGCGTTTTGCCTCAACTTGAACAGCCCTATGTGTTCATCAAAC 432
hCoV-19/France/ARA-HCL125019754801/2025|EPI_ISL_19895035|2025-05-22          TAGTAGAAGTTGAAAAAGGCGTTTTGCCTCAACTTGAACAGCCCTATGTGTTCATCAAAC 432
hCoV-19/France/ARA-RELAB-HCL725000064601/2025|EPI_ISL_19895041|2025-05-15    TAGTAGAAGTTGAAAAAGGCGTTTTGCCTCAACTTGAACAGCCCTATGTGTTCATCAAAC 432
hCoV-19/France/ARA-RELAB-HCL725000066001/2025|EPI_ISL_19895046|2025-05-13    TAGTAGAAGTTGAAAAAGGCGTTTTGCCTCAACTTGAACAGCCCTATGTGTTCATCAAAC 432
hCoV-19/France/OCC-RELAB-HCL725000068701/2025|EPI_ISL_19905466|2025-05-21    TAGTAGAAGTTGAAAAAGGCGTTTTGCCTCAACTTGAACAGCCCTATGTGTTCATCAAAC 432
hCoV-19/France/OCC-RELAB-HCL725000069001/2025|EPI_ISL_19905468|2025-05-21    TAGTAGAAGTTGAAAAAGGCGTTTTGCCTCAACTTGAACAGCCCTATGTGTTCATCAAAC 432
hCoV-19/France/ARA-HCL025089279801/2025|EPI_ISL_19905469|2025-06-02          TAGTAGAAGTTGAAAAAGGCGTTTTGCCTCAACTTGAACAGCCCTATGTGTTCATCAAAC 432
hCoV-19/France/PAC-RELAB-HCL725000069901/2025|EPI_ISL_19908847|2025-05-26    TAGTAGAAGTTGAAAAAGGCGTTTTGCCTCAACTTGAACAGCCCTATGTGTTCATCAAAC 432
hCoV-19/France/PAC-RELAB-HCL725000069501/2025|EPI_ISL_19908849|2025-05-30    TAGTAGAAGTTGAAAAAGGCGTTTTGCCTCAACTTGAACAGCCCTATGTGTTCATCAAAC 432
hCoV-19/France/ARA-RELAB-HCL725000071701/2025|EPI_ISL_19908858|2025-05-22    TAGTAGAAGTTGAAAAAGGCGTTTTGCCTCAACTTGAACAGCCCTATGTGTTCATCAAAC 432
hCoV-19/France/ARA-HCL025093352901/2025|EPI_ISL_19908867|2025-06-10          TAGTAGAAGTTGAAAAAGGCGTTTTGCCTCAACTTGAACAGCCCTATGTGTTCATCAAAC 432
hCoV-19/France/ARA-RELAB-HCL725000065801/2025|EPI_ISL_19895045|2025-05-16    TAGTAGAAGTTGAAAAAGGCGTTTTGCCTCAACTTGAACAGCCCTATGTGTTCATCAAAC 432
hCoV-19/Denmark/DCGC-692234/2025|EPI_ISL_19879694|2025-05-05                 TAGTAGAAGTTGAAAAAGGCGTTTTGCCTCAACTTGAACAGCCCTATGTGTTCATCAAAN 434
hCoV-19/Finland/THL-02388/2025|EPI_ISL_19875931|2025-04-16                   TAGTAGAAGTTGAAAAAGGCGTTTTGCCTCAACTTGAACAGCCCTATGTGTTCATCAAAC 435
hCoV-19/Spain/PV-HUD-98024244/2025|EPI_ISL_19907759|2025-06-09               TAGTAGAAGTTGAAAAAGGCGTTTTGCCTCAACTTGAACAGCCCTATGTGTTCATCAAAC 435
hCoV-19/Netherlands/GE-RIVM-145386/2025|EPI_ISL_19873764|2025-05-01          TAGTAGAAGTTGAAAAAGGCGTTTTGCCTCAACTTGAACAGCCCTATGTGTTCATCAAAC 432
hCoV-19/Netherlands/ZH-RIVM-145413/2025|EPI_ISL_19891397|2025-04-30          TAGTAGAAGTTGAAAAAGGCGTTTTGCCTCAACTTGAACAGCCCTATGTGTTCATCAAAC 432
hCoV-19/Netherlands/NH-RIVM-145458/2025|EPI_ISL_19905062|2025-05-25          TAGTAGAAGTTGAAAAAGGCGTTTTGCCTCAACTTGAACAGCCCTATGTGTTCATCAAAC 432
hCoV-19/Netherlands/NH-RIVM-145463/2025|EPI_ISL_19905063|2025-05-20          TAGTAGAAGTTGAAAAAGGCGTTTTGCCTCAACTTGAACAGCCCTATGTGTTCATCAAAC 432
hCoV-19/Netherlands/UT-RIVM-145436/2025|EPI_ISL_19905072|2025-05-15          TAGTAGAAGTTGAAAAAGGCGTTTTGCCTCAACTTGAACAGCCCTATGTGTTCATCAAAC 432
hCoV-19/Netherlands/UT-RIVM-145470/2025|EPI_ISL_19905077|2025-06-02          TAGTAGAAGTTGAAAAAGGCGTTTTGCCTCAACTTGAACAGCCCTATGTGTTCATCAAAC 432
hCoV-19/Netherlands/ZH-RIVM-145478/2025|EPI_ISL_19905094|2025-05-30          TAGTAGAAGTTGAAAAAGGCGTTTTGCCTCAACTTGAACAGCCCTATGTGTTCATCAAAC 432
hCoV-19/Netherlands/ZH-RIVM-145480/2025|EPI_ISL_19905096|2025-05-31          TAGTAGAAGTTGAAAAAGGCGTTTTGCCTCAACTTGAACAGCCCTATGTGTTCATCAAAC 432
hCoV-19/Netherlands/UT-RIVM-145481/2025|EPI_ISL_19905078|2025-05-31          TAGTAGAAGTTGAAAAAGGCGTTTTGCCTCAACTTGAACAGCCCTATGTGTTCATCAAAC 432
hCoV-19/Scotland/CLIMB-CM7YRW9H/2025|EPI_ISL_19881711|2025-05-06             TAGTAGAAGTTGAAAAAGGCGTTTTGCCTCAACTTGAACAGCCCTATGTGTTCATCAAAC 404
hCoV-19/France/IDF-RELAB-IPP04872/2025|EPI_ISL_19911293|2025-05-22           TAGTAGAAGTTGAAAAAGGCGTTTTGCCTCAACTTGAACAGCCCTATGTGTTCATCAAAC 400
hCoV-19/Canary_Islands/CN-HUC-925112913/2025|EPI_ISL_19900668|2025-04-22     TAGTAGAAGTTGAAAAAGGCGTTTTGCCTCAACTTGAACAGCCCTATGTGTTCATCAAAC 408
hCoV-19/Scotland/CLIMB-CM7YJ5BB/2025|EPI_ISL_19906458|2025-05-27             TAGTAGAAGTTGAAAAAGGCGTTTTGCCTCAACTTGAACAGCCCTATGTGTTCATCAAAC 404
```

```
hCoV-19/Scotland/CLIMB-CM7Y8PHH/2025|EPI_ISL_19897755|2025-05-14            TAGTAGAAGTTGAAAAAGGCGTTTTGCCTCAACTTGAACAGCCCTATGTGTTCATCAAAC 404
hCoV-19/Spain/AN-PMC-42706/2025|EPI_ISL_19888325|2025-05-25                 TAGTAGAAGTTGAAAAAGGCGTTTTGCCTCAACTTGAACAGCCCTATGTGTTCATCAAAC 404
hCoV-19/Scotland/CLIMB-CM7YRIA1/2025|EPI_ISL_19869961|2025-04-20            TAGTAGAAGTTGAAAAAGGCGTTTTGCCTCAACTTGAACAGCCCTATGTGTTCATCAAAC 404
hCoV-19/Scotland/CLIMB-CM7Y8UWT/2025|EPI_ISL_19869966|2025-04-21            TAGTAGAAGTTGAAAAAGGCGTTTTGCCTCAACTTGAACAGCCCTATGTGTTCATCAAAC 404
hCoV-19/England/CLIMB-CM7YMFHB/2025|EPI_ISL_19877436|2025-05-10             TAGTAGAAGTTGAAAAAGGCGTTTTGCCTCAACTTGAACAGCCCTATGTGTTCATCAAAC 404
hCoV-19/Denmark/DCGC-692213/2025|EPI_ISL_19879673|2025-04-28                TAGTAGAAGTTGAAAAAGGCGTTTTGCCTCAACTTGAACAGCCCTATGTGTTCATCAAAC 404
hCoV-19/Wales/CLIMB-CM7YRAXQ/2025|EPI_ISL_19883075|2025-05-14               TAGTAGAAGTTGAAAAAGGCGTTTTGCCTCAACTTGAACAGCCCTATGTGTTCATCAAAC 404
hCoV-19/Spain/AN-PMC-42696/2025|EPI_ISL_19888316|2025-05-14                 TAGTAGAAGTTGAAAAAGGCGTTTTGCCTCAACTTGAACAGCCCTATGTGTTCATCAAAC 404
hCoV-19/Spain/AN-PMC-42719/2025|EPI_ISL_19888338|2025-05-15                 TAGTAGAAGTTGAAAAAGGCGTTTTGCCTCAACTTGAACAGCCCTATGTGTTCATCAAAC 404
hCoV-19/England/CLIMB-CM7YE7HB/2025|EPI_ISL_19892115|2025-05-19             TAGTAGAAGTTGAAAAAGGCGTTTTGCCTCAACTTGAACAGCCCTATGTGTTCATCAAAC 404
hCoV-19/France/GES-RELAB-IPP04599/2025|EPI_ISL_19893547|2025-05-05          TAGTAGAAGTTGAAAAAGGCGTTTTGCCTCAACTTGAACAGCCCTATGTGTTCATCAAAC 404
hCoV-19/Scotland/CLIMB-CM7YEPXZ/2025|EPI_ISL_19896028|2025-05-25            TAGTAGAAGTTGAAAAAGGCGTTTTGCCTCAACTTGAACAGCCCTATGTGTTCATCAAAC 404
hCoV-19/Scotland/CLIMB-CM7YF4QT/2025|EPI_ISL_19896032|2025-05-27            TAGTAGAAGTTGAAAAAGGCGTTTTGCCTCAACTTGAACAGCCCTATGTGTTCATCAAAC 404
hCoV-19/Scotland/CLIMB-CM7YKDZ6/2025|EPI_ISL_19896033|2025-05-27            TAGTAGAAGTTGAAAAAGGCGTTTTGCCTCAACTTGAACAGCCCTATGTGTTCATCAAAC 404
hCoV-19/Scotland/CLIMB-CM7YG8H4/2025|EPI_ISL_19897748|2025-05-02            TAGTAGAAGTTGAAAAAGGCGTTTTGCCTCAACTTGAACAGCCCTATGTGTTCATCAAAC 404
hCoV-19/Wales/CLIMB-CM7YJXNZ/2025|EPI_ISL_19900036|2025-05-26               TAGTAGAAGTTGAAAAAGGCGTTTTGCCTCAACTTGAACAGCCCTATGTGTTCATCAAAC 404
hCoV-19/Germany/NW-RKI-I-1148178/2025|EPI_ISL_19902055|2025-05-21           TAGTAGAAGTTGAAAAAGGCGTTTTGCCTCAACTTGAACAGCCCTATGTGTTCATCAAAC 404
hCoV-19/Germany/NI-RKI-I-1148183/2025|EPI_ISL_19902060|2025-05-18           TAGTAGAAGTTGAAAAAGGCGTTTTGCCTCAACTTGAACAGCCCTATGTGTTCATCAAAC 404
hCoV-19/Germany/NW-RKI-I-1148191/2025|EPI_ISL_19902068|2025-05-19           TAGTAGAAGTTGAAAAAGGCGTTTTGCCTCAACTTGAACAGCCCTATGTGTTCATCAAAC 404
hCoV-19/Spain/MD-HGUGM-66414184/2025|EPI_ISL_19906298|2025-05-25            TAGTAGAAGTTGAAAAAGGCGTTTTGCCTCAACTTGAACAGCCCTATGTGTTCATCAAAC 404
hCoV-19/Spain/MD-HGUGM-66400167/2025|EPI_ISL_19906320|2025-05-22            TAGTAGAAGTTGAAAAAGGCGTTTTGCCTCAACTTGAACAGCCCTATGTGTTCATCAAAC 404
hCoV-19/France/IDF-RELAB-IPP04646/2025|EPI_ISL_19907194|2025-05-16          TAGTAGAAGTTGAAAAAGGCGTTTTGCCTCAACTTGAACAGCCCTATGTGTTCATCAAAC 404
hCoV-19/England/CLIMB-CM7YKIUQ/2025|EPI_ISL_19908573|2025-06-07             TAGTAGAAGTTGAAAAAGGCGTTTTGCCTCAACTTGAACAGCCCTATGTGTTCATCAAAC 404
hCoV-19/France/GES-RELAB-IPP04823/2025|EPI_ISL_19911257|2025-05-26          TAGTAGAAGTTGAAAAAGGCGTTTTGCCTCAACTTGAACAGCCCTATGTGTTCATCAAAC 404
hCoV-19/France/IDF-RELAB-IPP04863/2025|EPI_ISL_19911265|2025-05-24          TAGTAGAAGTTGAAAAAGGCGTTTTGCCTCAACTTGAACAGCCCTATGTGTTCATCAAAC 404
hCoV-19/Denmark/DCGC-692245/2025|EPI_ISL_19879704|2025-04-28                TAGTAGAAGTTGAAAAAGGCGTTTTGCCTCAACTTGAACAGCCCTATGTGTTCATCAAAC 404

hCoV-19/Ireland/D-SVUH-FBA98650-31/2025|EPI_ISL_19866548|2025-05-04         GTTCGGATGCTCGAACTGCACCTCATGGTCATGTTATGGTTGAGCTGGTAGCAGAACTTG 84
hCoV-19/Spain/PV-HUB-5628663/2025|EPI_ISL_19908839|2025-05-16               GTTCGGATGCTCGAACTGCACCTCATGGTCATGTTATGGTTGAGCTGGTAGCAGAACTTG 337
hCoV-19/Spain/PV-HUB-41587498/2025|EPI_ISL_19908930|2025-05-20              GTTCGGATGCTCGAACTGCACCTCATGGTCATGTTATGGTTGAGCTGGTAGCAGAACTTG 332
hCoV-19/Spain/PV-HUB-56291851/2025|EPI_ISL_19908970|2025-05-31              GTTCGGATGCTCGAACTGCACCTCATGGTCATGTTATGGTTGAGCTGGTAGCAGAACTTG 333
hCoV-19/Northern_Ireland/CLIMB-CM7YR8I6/2025|EPI_ISL_19877334|2025-04-23    NNNNNNNNNNNNNNNNNNNNNNNNNNNNNNNTGTTATGGTTGAGCTGGTAGCAGAACTTG 441
hCoV-19/Germany/NW-RKI-I-1148136/2025|EPI_ISL_19871950|2025-04-23           NNNNNNNNNNNNNNNNNNNNNNNNNNNNNNNNNNN--NNN-N-NNNNNN-NNNN--NN-N 451
hCoV-19/Germany/NW-RKI-I-1148141/2025|EPI_ISL_19871955|2025-04-23           NNNNNNNNNNNNNNNNNNNNNNNNNNNNNNNNNNN--NNN-N-NNNNNN-NNNN--NN-N 451
hCoV-19/Germany/BW-RKI-I-1148164/2025|EPI_ISL_19884987|2025-04-17           NNNNNNNNNNNNNNNNNNNNNNNNNNNNNNNNNNN--NNN-N-NNNNNN-NNNN--NN-N 451
hCoV-19/Scotland/CLIMB-CM7YFGW7/2025|EPI_ISL_19897691|2025-05-24            GTTCGGATGCTCGAACTGCACCTCATGGTCATGTTANGGTTGAGCTGGTAGCAGAACTTG 464
hCoV-19/Ireland/G-LUH-N3473/2025|EPI_ISL_19893181|2025-05-14                GTTCGGATGCTCGAACTGCACCTCATGGTC------------------------------ 501
hCoV-19/Spain/AN-PMC-42728/2025|EPI_ISL_19888347|2025-05-25                 GTTCGGATGCTCGAACTGCACCTCATGGT------------------------------- 501
hCoV-19/Sweden/00200866492N2/2025|EPI_ISL_19870050|2025-04-22               GTTCGGATGCTCGAACTGCACCT------------------------------------- 501
hCoV-19/Sweden/00200867159N2/2025|EPI_ISL_19870052|2025-04-23               GTTCGGATGCTCGAACTGCACCT------------------------------------- 501
hCoV-19/Ireland/G-LUH-N3445/2025|EPI_ISL_19893154|2025-05-07                GTTCGGATGCTCGAACTGCACCT------------------------------------- 501
hCoV-19/Ireland/G-LUH-N3443/2025|EPI_ISL_19893152|2025-05-07                GTTCGGATGCTCGAACTGCACCTCA----------------------------------- 501
hCoV-19/Scotland/CLIMB-CM7YF9PJ/2025|EPI_ISL_19895983|2025-05-19            GTTCGGATGCTCGAACTGCACCTCAT---------------------------------- 501
hCoV-19/Scotland/CLIMB-CM7YKMK8/2025|EPI_ISL_19869976|2025-04-25            GTTCGGATGCTCGAACTGCACCTCATG--------------------------------- 501
hCoV-19/Ireland/G-LUH-N3444/2025|EPI_ISL_19893153|2025-05-07                GTTCGGATGCTCGAACTGCACCTCATG--------------------------------- 501
hCoV-19/Switzerland/VD-CHUV-8604552682/2025|EPI_ISL_19906527|2025-05-19     GTTCGGATGCTCGAACTGCACC-------------------------------------- 501
hCoV-19/Scotland/CLIMB-CM7YR3YO/2025|EPI_ISL_19896015|2025-05-24            GTTCGGATGCTCGAACTGCAC--------------------------------------- 501
hCoV-19/Sweden/O-18_SE100_25CS500180/2025|EPI_ISL_19866288|2025-04-15       GTTCGGATGCTCGAACTGCAC--------------------------------------- 501
hCoV-19/Ireland/D-CS250088/2025|EPI_ISL_19877457|2025-04-16                 GTTCGGATGCTCGAACTGCAC--------------------------------------- 501
hCoV-19/Ireland/G-LUH-N3442/2025|EPI_ISL_19893151|2025-05-07                GTTCGGATGCTCGAACTGCAC--------------------------------------- 501
hCoV-19/Scotland/CLIMB-CM7YMZ5M/2025|EPI_ISL_19906439|2025-05-22            GTTCGGATGCTCGAACTGCACC-------------------------------------- 501
hCoV-19/Scotland/CLIMB-CM7YR3ZG/2025|EPI_ISL_19897722|2025-05-13            GTTCGGATGCTCGAACTGCACCT------------------------------------- 501
```

```
hCoV-19/Scotland/CLIMB-CM7YEYGT/2025|EPI_ISL_19897741|2025-04-28            GTTCGGATGCTCGAACTGCACCT------------------------------------- 501
hCoV-19/Scotland/CLIMB-CM7YGAJ3/2025|EPI_ISL_19895963|2025-05-13            GTTCGGATGCTCGAACTGCACCTCATGGTCATGT-------------------------- 501
hCoV-19/Scotland/CLIMB-CM7YJ1ZO/2025|EPI_ISL_19897757|2025-05-14            GTTCGGATGCTCGAACTGCACCTCATGGTCATG--------------------------- 501
hCoV-19/Scotland/CLIMB-CM7Y8C4Y/2025|EPI_ISL_19906404|2025-05-15            GTTCGGATGCTCGAACTGCACCTCAT---------------------------------- 501
hCoV-19/Northern_Ireland/CLIMB-CM7YGGYM/2025|EPI_ISL_19897641|2025-05-23    GTTCGGATGCTCGAACTGCACCTCATGGTCA----------------------------- 501
hCoV-19/Scotland/CLIMB-CM7YFW7M/2025|EPI_ISL_19881718|2025-05-10            GTTCGGATGCTCGAACTGCACCTCATGG-------------------------------- 501
hCoV-19/Scotland/CLIMB-CM7YEMXP/2025|EPI_ISL_19888387|2025-05-10            GTTCGGATGCTCGAACTGCACCTCATGGT------------------------------- 501
hCoV-19/Spain/CT-HUVH-E593511/2025|EPI_ISL_19881731|2025-05-06              GTTCGGATGNNNNNNNNNNNNNNNNNNNNNNNNNNNNNNNNNNNCTGGTAGCAGAACTTG 485
hCoV-19/Spain/CT-HUJT-E325362/2025|EPI_ISL_19889935|2025-05-20              NNNNNNNTGNNNNNNNNNNNNNNNNNNNNNNNNNNNNNNNNNNNNNNNNNNNNGAACTTG 486
hCoV-19/Spain/PV-HUD-84743204/2025|EPI_ISL_19907746|2025-06-06              GTTCGGATGCTCGAACTGCACCTCAT---------------GAGCTGGTAGCAGAACTTG 472
hCoV-19/Ireland/D-SVUH-FBB21492-54/2025|EPI_ISL_19881758|2025-05-11         GTTCGGATGCTCGAACTGCACCTCATGGTCATGTT---GTTGAGCTGGTAGCAGAACTTG 485
hCoV-19/Spain/CT-HUVH-E596725/2025|EPI_ISL_19881729|2025-05-10              GTTCGGATGCTCGAACTGCACCTCATGGTCATGTTATGGTTGAGCTGGTAGCAGAACTTG 482
hCoV-19/Spain/CT-HUVH-E593882/2025|EPI_ISL_19881732|2025-05-06              GTTCGGATGCTCGAACTGCACCTCATGGTCATGTTATGGTTGAGCTGGTAGCAGAACTTG 482
hCoV-19/Finland/THL-02385/2025|EPI_ISL_19875928|2025-04-16                  GTTCGGATGCTCGAACTGCACCTCATGGTCATGTTATGGTTGAGCTGGTAGCAGAACTTG 489
hCoV-19/Ireland/D-BH-02351167/2025|EPI_ISL_19911337|2025-05-28              GTTCGGATGCTCGAACTGCACCTCATGGTCATGTTATGGTTGAGCTGGTAGCAGAACTTG 488
hCoV-19/Ireland/D-NVRL-XIRL00001782/2025|EPI_ISL_19896104|2025-05-14        GTTCGGATGCTCGAACTGCACCTCATGGTCATGTTATGGTTGAGCTGGTAGCAGAACTTG 488
hCoV-19/Ireland/TA-NVRL-GIRL00039444/2025|EPI_ISL_19896118|2025-05-16       GTTCGGATGCTCGAACTGCACCTCATGGTCATGTTATGGTTGAGCTGGTAGCAGAACTTG 488
hCoV-19/Spain/GA-CHUAC-13223/2025|EPI_ISL_19894946|2025-05-31               GTTCGGATGCTCGAACTGCACCTCATGGTCATGTTATGGTTGAGCTGGTAGCAGAACTTG 487
hCoV-19/Spain/GA-CHUAC-13215/2025|EPI_ISL_19894939|2025-05-29               GTTCGGATGCTCGAACTGCACCTCATGGTCATGTTATGGTTGAGCTGGTAGCAGAACTTG 487
hCoV-19/Finland/THL-02398/2025|EPI_ISL_19875939|2025-05-04                  GTTCGGATGCTCGAACTGCACCTCATGGTCATGTTATGGTTGAGCTGGTAGCAGAACTTG 488
hCoV-19/Finland/THL-02400/2025|EPI_ISL_19875941|2025-05-03                  GTTCGGATGCTCGAACTGCACCTCATGGTCATGTTATGGTTGAGCTGGTAGCAGAACTTG 488
hCoV-19/Ireland/LK-UHL-702929/2025|EPI_ISL_19877895|2025-04-16              GTTCGGATGCTCGAACTGCACCTCATGGTCATGTTATGGTTGAGCTGGTAGCAGAACTTG 488
hCoV-19/Ireland/LK-UHL-703788/2025|EPI_ISL_19877905|2025-05-03              GTTCGGATGCTCGAACTGCACCTCATGGTCATGTTATGGTTGAGCTGGTAGCAGAACTTG 488
hCoV-19/Ireland/LK-UHL-703851/2025|EPI_ISL_19877907|2025-05-04              GTTCGGATGCTCGAACTGCACCTCATGGTCATGTTATGGTTGAGCTGGTAGCAGAACTTG 488
hCoV-19/Ireland/LK-UHL-703889/2025|EPI_ISL_19877908|2025-05-05              GTTCGGATGCTCGAACTGCACCTCATGGTCATGTTATGGTTGAGCTGGTAGCAGAACTTG 488
hCoV-19/Ireland/D-SVUH-FBB21492-49/2025|EPI_ISL_19881756|2025-05-14         GTTCGGATGCTCGAACTGCACCTCATGGTCATGTTATGGTTGAGCTGGTAGCAGAACTTG 488
hCoV-19/Ireland/D-SVUH-FBB21492-59/2025|EPI_ISL_19881763|2025-05-07         GTTCGGATGCTCGAACTGCACCTCATGGTCATGTTATGGTTGAGCTGGTAGCAGAACTTG 488
hCoV-19/Spain/CT-HUGTiP-G3R212/2025|EPI_ISL_19883055|2025-05-20             GTTCGGATGCTCGAACTGCACCTCATGGTCATGTTATGGTTGAGCTGGTAGCAGAACTTG 488
hCoV-19/Ireland/LK-UHL-704398/2025|EPI_ISL_19888621|2025-05-16              GTTCGGATGCTCGAACTGCACCTCATGGTCATGTTATGGTTGAGCTGGTAGCAGAACTTG 488
hCoV-19/Ireland/G-GUH-N3466/2025|EPI_ISL_19893175|2025-05-03                GTTCGGATGCTCGAACTGCACCTCATGGTCATGTTATGGTTGAGCTGGTAGCAGAACTTG 488
hCoV-19/Belgium/Sciensano-LS-S5276/2025|EPI_ISL_19893834|2025-04-22         GTTCGGATGCTCGAACTGCACCTCATGGTCATGTTATGGTTGAGCTGGTAGCAGAACTTG 488
hCoV-19/Italy/VEN-ULSS8-0019244_VI/2025|EPI_ISL_19899362|2025-05-26         GTTCGGATGCTCGAACTGCACCTCATGGTCATGTTATGGTTGAGCTGGTAGCAGAACTTG 488
hCoV-19/Spain/CT-HUVH-E618109/2025|EPI_ISL_19907773|2025-06-10              GTTCGGATGCTCGAACTGCACCTCATGGTCATGTTATGGTTGAGCTGGTAGCAGAACTTG 488
hCoV-19/Ireland/D-BH-03819149/2025|EPI_ISL_19911343|2025-05-29              GTTCGGATGCTCGAACTGCACCTCATGGTCATGTTATGGTTGAGCTGGTAGCAGAACTTG 488
hCoV-19/Ireland/D-BH-03993161/2025|EPI_ISL_19911355|2025-06-10              GTTCGGATGCTCGAACTGCACCTCATGGTCATGTTATGGTTGAGCTGGTAGCAGAACTTG 488
hCoV-19/Spain/CT-HUVH-E587040/2025|EPI_ISL_19871881|2025-04-26              GTTCGGATGCTCGAACTGCACCTCATGGTCATGTTATGGTTGAGCTGGTAGCAGAACTTG 487
hCoV-19/Finland/THL-02392/2025|EPI_ISL_19875934|2025-05-02                  GTTCGGATGCTCGAACTGCACCTCATGGTCATGTTATGGTTGAGCTGGTAGCAGAACTTG 487
hCoV-19/Spain/GA-CHUAC-13181/2025|EPI_ISL_19890617|2025-05-20               GTTCGGATGCTCGAACTGCACCTCATGGTCATGTTATGGTTGAGCTGGTAGCAGAACTTG 487
hCoV-19/Spain/CT-HUVH-E614843/2025|EPI_ISL_19907763|2025-06-05              GTTCGGATGCTCGAACTGCACCTCATGGTCATGTTATGGTTGAGCTGGTAGCAGAACTTG 487
hCoV-19/Italy/UMB-LAOP-172-4-02/2025|EPI_ISL_19899474|2025-05-08            GTTCGGATGCTCGAACTGCACCTCATGGTCATGTTATGGTTGAGCTGGTAGC-------- 501
hCoV-19/Spain/NC-CHN-01008932/2025|EPI_ISL_19900595|2025-04-04              GTTCGGATGCTCGAACTGCACCTCATGGTCATGTTATGGTTGAGCTGGTA---------- 501
hCoV-19/Northern_Ireland/CLIMB-CM7YGDRO/2025|EPI_ISL_19877331|2025-04-14    GTTCGGATGCTCGAACTGCACCTCATGGTCATGTTATGGTTGAGCTGGTAGCAGAACTTG 499
hCoV-19/Scotland/CLIMB-CM7YEE96/2025|EPI_ISL_19897754|2025-05-13            GTTCGGATGCTCGAACTGCACCTCATGGTCATGTTATGGTTGAGCTGGTAGCAGAACTTG 498
hCoV-19/France/ARA-HCL125018289001/2025|EPI_ISL_19880005|2025-05-12         GTTCGGATGCTCGAACTGCACCTCATGGTCATGTTATGGTTGAGCTGGTAGCAGAACTTG 492
hCoV-19/France/ARA-RELAB-HCL725000057501/2025|EPI_ISL_19880014|2025-04-28   GTTCGGATGCTCGAACTGCACCTCATGGTCATGTTATGGTTGAGCTGGTAGCAGAACTTG 492
hCoV-19/France/ARA-RELAB-HCL725000059501/2025|EPI_ISL_19885160|2025-04-25   GTTCGGATGCTCGAACTGCACCTCATGGTCATGTTATGGTTGAGCTGGTAGCAGAACTTG 492
hCoV-19/France/NAQ-RELAB-HCL725000061001/2025|EPI_ISL_19885161|2025-05-10   GTTCGGATGCTCGAACTGCACCTCATGGTCATGTTATGGTTGAGCTGGTAGCAGAACTTG 492
hCoV-19/France/NAQ-RELAB-HCL725000060901/2025|EPI_ISL_19885180|2025-05-09   GTTCGGATGCTCGAACTGCACCTCATGGTCATGTTATGGTTGAGCTGGTAGCAGAACTTG 492
hCoV-19/France/ARA-HCL025075546601/2025|EPI_ISL_19885182|2025-05-09         GTTCGGATGCTCGAACTGCACCTCATGGTCATGTTATGGTTGAGCTGGTAGCAGAACTTG 492
hCoV-19/France/ARA-HCL125018461901/2025|EPI_ISL_19885204|2025-05-14         GTTCGGATGCTCGAACTGCACCTCATGGTCATGTTATGGTTGAGCTGGTAGCAGAACTTG 492
hCoV-19/France/ARA-HCL125018655901/2025|EPI_ISL_19885206|2025-05-14         GTTCGGATGCTCGAACTGCACCTCATGGTCATGTTATGGTTGAGCTGGTAGCAGAACTTG 492
hCoV-19/France/ARA-HCL125019155501/2025|EPI_ISL_19885209|2025-05-18         GTTCGGATGCTCGAACTGCACCTCATGGTCATGTTATGGTTGAGCTGGTAGCAGAACTTG 492
hCoV-19/France/ARA-HCL125019240901/2025|EPI_ISL_19885210|2025-05-19         GTTCGGATGCTCGAACTGCACCTCATGGTCATGTTATGGTTGAGCTGGTAGCAGAACTTG 492
```

hCoV-19/France/ARA-HCL125019249201/2025|EPI_ISL_19885211|2025-05-19	GTTCGGATGCTCGAACTGCACCTCATGGTCATGTTATGGTTGAGCTGGTAGCAGAACTTG	492
hCoV-19/France/ARA-HCL125019399701/2025|EPI_ISL_19885212|2025-05-20	GTTCGGATGCTCGAACTGCACCTCATGGTCATGTTATGGTTGAGCTGGTAGCAGAACTTG	492
hCoV-19/France/ARA-HCL125019408901/2025|EPI_ISL_19885213|2025-05-20	GTTCGGATGCTCGAACTGCACCTCATGGTCATGTTATGGTTGAGCTGGTAGCAGAACTTG	492
hCoV-19/France/OCC-RELAB-HCL725000064001/2025|EPI_ISL_19895004|2025-05-14	GTTCGGATGCTCGAACTGCACCTCATGGTCATGTTATGGTTGAGCTGGTAGCAGAACTTG	492
hCoV-19/France/OCC-RELAB-HCL725000064101/2025|EPI_ISL_19895005|2025-05-14	GTTCGGATGCTCGAACTGCACCTCATGGTCATGTTATGGTTGAGCTGGTAGCAGAACTTG	492
hCoV-19/France/NAQ-RELAB-HCL725000062601/2025|EPI_ISL_19895009|2025-05-16	GTTCGGATGCTCGAACTGCACCTCATGGTCATGTTATGGTTGAGCTGGTAGCAGAACTTG	492
hCoV-19/France/ARA-RELAB-HCL725000062401/2025|EPI_ISL_19895017|2025-05-16	GTTCGGATGCTCGAACTGCACCTCATGGTCATGTTATGGTTGAGCTGGTAGCAGAACTTG	492
hCoV-19/France/ARA-RELAB-HCL725000062101/2025|EPI_ISL_19895018|2025-05-13	GTTCGGATGCTCGAACTGCACCTCATGGTCATGTTATGGTTGAGCTGGTAGCAGAACTTG	492
hCoV-19/France/ARA-HCL125019625101/2025|EPI_ISL_19895034|2025-05-21	GTTCGGATGCTCGAACTGCACCTCATGGTCATGTTATGGTTGAGCTGGTAGCAGAACTTG	492
hCoV-19/France/ARA-HCL125019754801/2025|EPI_ISL_19895035|2025-05-22	GTTCGGATGCTCGAACTGCACCTCATGGTCATGTTATGGTTGAGCTGGTAGCAGAACTTG	492
hCoV-19/France/ARA-RELAB-HCL725000064601/2025|EPI_ISL_19895041|2025-05-15	GTTCGGATGCTCGAACTGCACCTCATGGTCATGTTATGGTTGAGCTGGTAGCAGAACTTG	492
hCoV-19/France/ARA-RELAB-HCL725000066001/2025|EPI_ISL_19895046|2025-05-13	GTTCGGATGCTCGAACTGCACCTCATGGTCATGTTATGGTTGAGCTGGTAGCAGAACTTG	492
hCoV-19/France/OCC-RELAB-HCL725000068701/2025|EPI_ISL_19905466|2025-05-21	GTTCGGATGCTCGAACTGCACCTCATGGTCATGTTATGGTTGAGCTGGTAGCAGAACTTG	492
hCoV-19/France/OCC-RELAB-HCL725000069001/2025|EPI_ISL_19905468|2025-05-21	GTTCGGATGCTCGAACTGCACCTCATGGTCATGTTATGGTTGAGCTGGTAGCAGAACTTG	492
hCoV-19/France/ARA-HCL025089279801/2025|EPI_ISL_19905469|2025-06-02	GTTCGGATGCTCGAACTGCACCTCATGGTCATGTTATGGTTGAGCTGGTAGCAGAACTTG	492
hCoV-19/France/PAC-RELAB-HCL725000069901/2025|EPI_ISL_19908847|2025-05-26	GTTCGGATGCTCGAACTGCACCTCATGGTCATGTTATGGTTGAGCTGGTAGCAGAACTTG	492
hCoV-19/France/PAC-RELAB-HCL725000069501/2025|EPI_ISL_19908849|2025-05-30	GTTCGGATGCTCGAACTGCACCTCATGGTCATGTTATGGTTGAGCTGGTAGCAGAACTTG	492
hCoV-19/France/ARA-RELAB-HCL725000071701/2025|EPI_ISL_19908858|2025-05-22	GTTCGGATGCTCGAACTGCACCTCATGGTCATGTTATGGTTGAGCTGGTAGCAGAACTTG	492
hCoV-19/France/ARA-HCL025093352901/2025|EPI_ISL_19908867|2025-06-10	GTTCGGATGCTCGAACTGCACCTCATGGTCATGTTATGGTTGAGCTGGTAGCAGAACTTG	492
hCoV-19/France/ARA-RELAB-HCL725000065801/2025|EPI_ISL_19895045|2025-05-16	GTTCGGATGCTCGAACTGCACCTCATGGTCATGTTATGGTTGAGCTGGTAGCAGAACTTG	492
hCoV-19/Denmark/DCGC-692234/2025|EPI_ISL_19879694|2025-05-05	NTTCGGATGCTCGAACTGCACCTCATGGTCATGTTATGGTTGAGCTGGTAGCAGAACTTG	494
hCoV-19/Finland/THL-02388/2025|EPI_ISL_19875931|2025-04-16	GTTCGGATGCTCGAACTGCACCTCATGGTCATGTTATGGTTGAGCTGGTAGCAGAACTTG	495
hCoV-19/Spain/PV-HUD-98024244/2025|EPI_ISL_19907759|2025-06-09	GTTCGGATGCTCGAACTGCACCTCATGGTCATGTTATGGTTGAGCTGGTAGCAGAACTTG	495
hCoV-19/Netherlands/GE-RIVM-145386/2025|EPI_ISL_19873764|2025-05-01	GTTCGGATGCTCGAACTGCACCTCATGGTCATGTTATGGTTGAGCTGGTAGCAGAACTTG	492
hCoV-19/Netherlands/ZH-RIVM-145413/2025|EPI_ISL_19891397|2025-04-30	GTTCGGATGCTCGAACTGCACCTCATGGTCATGTTATGGTTGAGCTGGTAGCAGAACTTG	492
hCoV-19/Netherlands/NH-RIVM-145458/2025|EPI_ISL_19905062|2025-05-25	GTTCGGATGCTCGAACTGCACCTCATGGTCATGTTATGGTTGAGCTGGTAGCAGAACTTG	492
hCoV-19/Netherlands/NH-RIVM-145463/2025|EPI_ISL_19905063|2025-05-20	GTTCGGATGCTCGAACTGCACCTCATGGTCATGTTATGGTTGAGCTGGTAGCAGAACTTG	492
hCoV-19/Netherlands/UT-RIVM-145436/2025|EPI_ISL_19905072|2025-05-15	GTTCGGATGCTCGAACTGCACCTCATGGTCATGTTATGGTTGAGCTGGTAGCAGAACTTG	492
hCoV-19/Netherlands/UT-RIVM-145470/2025|EPI_ISL_19905077|2025-06-02	GTTCGGATGCTCGAACTGCACCTCATGGTCATGTTATGGTTGAGCTGGTAGCAGAACTTG	492
hCoV-19/Netherlands/ZH-RIVM-145478/2025|EPI_ISL_19905094|2025-05-30	GTTCGGATGCTCGAACTGCACCTCATGGTCATGTTATGGTTGAGCTGGTAGCAGAACTTG	492
hCoV-19/Netherlands/ZH-RIVM-145480/2025|EPI_ISL_19905096|2025-05-31	GTTCGGATGCTCGAACTGCACCTCATGGTCATGTTATGGTTGAGCTGGTAGCAGAACTTG	492
hCoV-19/Netherlands/UT-RIVM-145481/2025|EPI_ISL_19905078|2025-05-31	GTTCGGATGCTCGAACTGCACCTCATGGTCATGTTATGGTTGAGCTGGTAGCAGAACTTG	492
hCoV-19/Scotland/CLIMB-CM7YRW9H/2025|EPI_ISL_19881711|2025-05-06	GTTCGGATGCTCGAACTGCACCTCATGGTCATGTT---GTTGAGCTGGTAGCAGAACTTG	461
hCoV-19/France/IDF-RELAB-IPP04872/2025|EPI_ISL_19911293|2025-05-22	GTTCGGATGCTCGAACTGCACCTCATGGTCATGTTATGGTTGAGCTGGTAGCAGAACTTG	460
hCoV-19/Canary_Islands/CN-HUC-925112913/2025|EPI_ISL_19900668|2025-04-22	GTTCGGATGCTCGAACTGCACCTCATGGTCATGTTATGGTTGAGCTGGTAGCAGAACTTG	468
hCoV-19/Scotland/CLIMB-CM7YJ5BB/2025|EPI_ISL_19906458|2025-05-27	GTTCGGATGCTCGAACTGCACCTCATGGTCATGTTATGGTTGAGCTGGTAGCAGAACTTG	464
hCoV-19/Scotland/CLIMB-CM7Y8PHH/2025|EPI_ISL_19897755|2025-05-14	GTTCGGATGCTCGAACTGCACCTCATGGTCATGTTATGGTTGAGCTGGTAGCAGAACTTG	464
hCoV-19/Spain/AN-PMC-42706/2025|EPI_ISL_19888325|2025-05-25	GTTCGGATGCTCGAACTGCACCTCATGGTCATGTTATGGTTGAGCTGGTAGCAGAACTTG	464
hCoV-19/Scotland/CLIMB-CM7YRIA1/2025|EPI_ISL_19869961|2025-04-20	GTTCGGATGCTCGAACTGCACCTCATGGTCATGTTATGGTTGAGCTGGTAGCAGAACTTG	464
hCoV-19/Scotland/CLIMB-CM7Y8UWT/2025|EPI_ISL_19869966|2025-04-21	GTTCGGATGCTCGAACTGCACCTCATGGTCATGTTATGGTTGAGCTGGTAGCAGAACTTG	464
hCoV-19/England/CLIMB-CM7YMFHB/2025|EPI_ISL_19877436|2025-05-10	GTTCGGATGCTCGAACTGCACCTCATGGTCATGTTATGGTTGAGCTGGTAGCAGAACTTG	464
hCoV-19/Denmark/DCGC-692213/2025|EPI_ISL_19879673|2025-04-28	GTTCGGATGCTCGAACTGCACCTCATGGTCATGTTATGGTTGAGCTGGTAGCAGAACTTG	464
hCoV-19/Wales/CLIMB-CM7YRAXQ/2025|EPI_ISL_19883075|2025-05-14	GTTCGGATGCTCGAACTGCACCTCATGGTCATGTTATGGTTGAGCTGGTAGCAGAACTTG	464
hCoV-19/Spain/AN-PMC-42696/2025|EPI_ISL_19888316|2025-05-14	GTTCGGATGCTCGAACTGCACCTCATGGTCATGTTATGGTTGAGCTGGTAGCAGAACTTG	464
hCoV-19/Spain/AN-PMC-42719/2025|EPI_ISL_19888338|2025-05-15	GTTCGGATGCTCGAACTGCACCTCATGGTCATGTTATGGTTGAGCTGGTAGCAGAACTTG	464
hCoV-19/England/CLIMB-CM7YE7HB/2025|EPI_ISL_19892115|2025-05-19	GTTCGGATGCTCGAACTGCACCTCATGGTCATGTTATGGTTGAGCTGGTAGCAGAACTTG	464
hCoV-19/France/GES-RELAB-IPP04599/2025|EPI_ISL_19893547|2025-05-05	GTTCGGATGCTCGAACTGCACCTCATGGTCATGTTATGGTTGAGCTGGTAGCAGAACTTG	464
hCoV-19/Scotland/CLIMB-CM7YEPXZ/2025|EPI_ISL_19896028|2025-05-25	GTTCGGATGCTCGAACTGCACCTCATGGTCATGTTATGGTTGAGCTGGTAGCAGAACTTG	464
hCoV-19/Scotland/CLIMB-CM7YF4QT/2025|EPI_ISL_19896032|2025-05-27	GTTCGGATGCTCGAACTGCACCTCATGGTCATGTTATGGTTGAGCTGGTAGCAGAACTTG	464
hCoV-19/Scotland/CLIMB-CM7YKDZ6/2025|EPI_ISL_19896033|2025-05-27	GTTCGGATGCTCGAACTGCACCTCATGGTCATGTTATGGTTGAGCTGGTAGCAGAACTTG	464
hCoV-19/Scotland/CLIMB-CM7YG8H4/2025|EPI_ISL_19897748|2025-05-02	GTTCGGATGCTCGAACTGCACCTCATGGTCATGTTATGGTTGAGCTGGTAGCAGAACTTG	464
hCoV-19/Wales/CLIMB-CM7YJXNZ/2025|EPI_ISL_19900036|2025-05-26	GTTCGGATGCTCGAACTGCACCTCATGGTCATGTTATGGTTGAGCTGGTAGCAGAACTTG	464
hCoV-19/Germany/NW-RKI-I-1148178/2025|EPI_ISL_19902055|2025-05-21	GTTCGGATGCTCGAACTGCACCTCATGGTCATGTTATGGTTGAGCTGGTAGCAGAACTTG	464

```
hCoV-19/Germany/NI-RKI-I-1148183/2025|EPI_ISL_19902060|2025-05-18          GTTCGGATGCTCGAACTGCACCTCATGGTCATGTTATGGTTGAGCTGGTAGCAGAACTTG 464
hCoV-19/Germany/NW-RKI-I-1148191/2025|EPI_ISL_19902068|2025-05-19          GTTCGGATGCTCGAACTGCACCTCATGGTCATGTTATGGTTGAGCTGGTAGCAGAACTTG 464
hCoV-19/Spain/MD-HGUGM-66414184/2025|EPI_ISL_19906298|2025-05-25           GTTCGGATGCTCGAACTGCACCTCATGGTCATGTTATGGTTGAGCTGGTAGCAGAACTTG 464
hCoV-19/Spain/MD-HGUGM-66400167/2025|EPI_ISL_19906320|2025-05-22           GTTCGGATGCTCGAACTGCACCTCATGGTCATGTTATGGTTGAGCTGGTAGCAGAACTTG 464
hCoV-19/France/IDF-RELAB-IPP04646/2025|EPI_ISL_19907194|2025-05-16         GTTCGGATGCTCGAACTGCACCTCATGGTCATGTTATGGTTGAGCTGGTAGCAGAACTTG 464
hCoV-19/England/CLIMB-CM7YKIUQ/2025|EPI_ISL_19908573|2025-06-07            GTTCGGATGCTCGAACTGCACCTCATGGTCATGTTATGGTTGAGCTGGTAGCAGAACTTG 464
hCoV-19/France/GES-RELAB-IPP04823/2025|EPI_ISL_19911257|2025-05-26         GTTCGGATGCTCGAACTGCACCTCATGGTCATGTTATGGTTGAGCTGGTAGCAGAACTTG 464
hCoV-19/France/IDF-RELAB-IPP04863/2025|EPI_ISL_19911265|2025-05-24         GTTCGGATGCTCGAACTGCACCTCATGGTCATGTTATGGTTGAGCTGGTAGCAGAACTTG 464
hCoV-19/Denmark/DCGC-692245/2025|EPI_ISL_19879704|2025-04-28               GTTCGGATGCTCGAACTGCACCTCATGGTCATGTTATGGTTGAGCTGGTGGCAGAACTTG 464


hCoV-19/Ireland/D-SVUH-FBA98650-31/2025|EPI_ISL_19866548|2025-05-04        AAGGCATTCAGTACGGTCGTAGTGGTGAGACACTTGGTGTCCTTGTCCCTCATGTGGGCG 144
hCoV-19/Spain/PV-HUB-5628663/2025|EPI_ISL_19908839|2025-05-16              AAGGCATTCAGTACGGTCGTAGTGGTGAGACACTTGGTGTCCTTGTCCCTCATGTGGGCG 397
hCoV-19/Spain/PV-HUB-41587498/2025|EPI_ISL_19908930|2025-05-20             AAGGCATTCAGTACGGTCGTAGTGGTGAGACACTTGGTGTCCTTGTCCCTCATGTGGGCG 392
hCoV-19/Spain/PV-HUB-56291851/2025|EPI_ISL_19908970|2025-05-31             AAGGCATTCAGTACGGTCGTAGTGGTGAGACACTTGGTGTCCTTGTCCCTCATGTGGGCG 393
hCoV-19/Northern_Ireland/CLIMB-CM7YR8I6/2025|EPI_ISL_19877334|2025-04-23   AAGGCATTCAGTACGGTCGTAGTGGTGAGACACTTGGTGTCNNNNNNNNNNNNNNNNNNN 501
hCoV-19/Germany/NW-RKI-I-1148136/2025|EPI_ISL_19871950|2025-04-23          -NNNN--NN-NN-NNNNNNN-NNNN-N-N-NNN-NNNNNNNNNNNNNNNNNNNNNNNNNN 501
hCoV-19/Germany/NW-RKI-I-1148141/2025|EPI_ISL_19871955|2025-04-23          -NNNN--NN-NN-NNNNNNN-NNNN-N-N-NNN-NNNNNNNNNNNNNNNNNNNNNNNNNN 501
hCoV-19/Germany/BW-RKI-I-1148164/2025|EPI_ISL_19884987|2025-04-17          -NNNN--NN-NN-NNNNNNN-NNNN-N-N-NNN-NNNNNNNNNNNNNNNNNNNNNNNNNN 501
hCoV-19/Scotland/CLIMB-CM7YFGW7/2025|EPI_ISL_19897691|2025-05-24           AAGGCATTCAGTACGGTCGTAGTGGTGAGACACTTGG----------------------- 501
hCoV-19/Ireland/G-LUH-N3473/2025|EPI_ISL_19893181|2025-05-14               ------------------------------------------------------------ 501
hCoV-19/Spain/AN-PMC-42728/2025|EPI_ISL_19888347|2025-05-25                ------------------------------------------------------------ 501
hCoV-19/Sweden/000200866492N2/2025|EPI_ISL_19870050|2025-04-22             ------------------------------------------------------------ 501
hCoV-19/Sweden/000200867159N2/2025|EPI_ISL_19870052|2025-04-23             ------------------------------------------------------------ 501
hCoV-19/Ireland/G-LUH-N3445/2025|EPI_ISL_19893154|2025-05-07               ------------------------------------------------------------ 501
hCoV-19/Ireland/G-LUH-N3443/2025|EPI_ISL_19893152|2025-05-07               ------------------------------------------------------------ 501
hCoV-19/Scotland/CLIMB-CM7YF9PJ/2025|EPI_ISL_19895983|2025-05-19           ------------------------------------------------------------ 501
hCoV-19/Scotland/CLIMB-CM7YKMK8/2025|EPI_ISL_19869976|2025-04-25           ------------------------------------------------------------ 501
hCoV-19/Ireland/G-LUH-N3444/2025|EPI_ISL_19893153|2025-05-07               ------------------------------------------------------------ 501
hCoV-19/Switzerland/VD-CHUV-8604552682/2025|EPI_ISL_19906527|2025-05-19    ------------------------------------------------------------ 501
hCoV-19/Scotland/CLIMB-CM7YR3YO/2025|EPI_ISL_19896015|2025-05-24           ------------------------------------------------------------ 501
hCoV-19/Sweden/O-18_SE100_25CS500180/2025|EPI_ISL_19866288|2025-04-15      ------------------------------------------------------------ 501
hCoV-19/Ireland/D-CS250088/2025|EPI_ISL_19877457|2025-04-16                ------------------------------------------------------------ 501
hCoV-19/Ireland/G-LUH-N3442/2025|EPI_ISL_19893151|2025-05-07               ------------------------------------------------------------ 501
hCoV-19/Scotland/CLIMB-CM7YMZ5M/2025|EPI_ISL_19906439|2025-05-22           ------------------------------------------------------------ 501
hCoV-19/Scotland/CLIMB-CM7YR3ZG/2025|EPI_ISL_19897722|2025-05-13           ------------------------------------------------------------ 501
hCoV-19/Scotland/CLIMB-CM7YEYGT/2025|EPI_ISL_19897741|2025-04-28           ------------------------------------------------------------ 501
hCoV-19/Scotland/CLIMB-CM7YGAJ3/2025|EPI_ISL_19895963|2025-05-13           ------------------------------------------------------------ 501
hCoV-19/Scotland/CLIMB-CM7YJ1ZO/2025|EPI_ISL_19897757|2025-05-14           ------------------------------------------------------------ 501
hCoV-19/Scotland/CLIMB-CM7Y8C4Y/2025|EPI_ISL_19906404|2025-05-15           ------------------------------------------------------------ 501
hCoV-19/Northern_Ireland/CLIMB-CM7YGGYM/2025|EPI_ISL_19897641|2025-05-23   ------------------------------------------------------------ 501
hCoV-19/Scotland/CLIMB-CM7YFW7M/2025|EPI_ISL_19881718|2025-05-10           ------------------------------------------------------------ 501
hCoV-19/Scotland/CLIMB-CM7YEMXP/2025|EPI_ISL_19888387|2025-05-10           ------------------------------------------------------------ 501
hCoV-19/Spain/CT-HUVH-E593511/2025|EPI_ISL_19881731|2025-05-06             AAGGCATTCAGTACGG-------------------------------------------- 501
hCoV-19/Spain/CT-HUJT-E325362/2025|EPI_ISL_19889935|2025-05-20             AAGNNNNNCAGTACG--------------------------------------------- 501
hCoV-19/Spain/PV-HUD-84743204/2025|EPI_ISL_19907746|2025-06-06             AAGGCATTCAGTACGGTCGTAGTGGTGAG------------------------------- 501
hCoV-19/Ireland/D-SVUH-FBB21492-54/2025|EPI_ISL_19881758|2025-05-11        AAGGCATTCAGTACGG-------------------------------------------- 501
hCoV-19/Spain/CT-HUVH-E596725/2025|EPI_ISL_19881729|2025-05-10             AAGGCATTCAGTACGGTCG----------------------------------------- 501
hCoV-19/Spain/CT-HUVH-E593882/2025|EPI_ISL_19881732|2025-05-06             AAGGCATTCAGTACGGTCG----------------------------------------- 501
hCoV-19/Finland/THL-02385/2025|EPI_ISL_19875928|2025-04-16                 AAGGCATTCAGT------------------------------------------------ 501
hCoV-19/Ireland/D-BH-02351167/2025|EPI_ISL_19911337|2025-05-28             AAGGCATTCAGTA----------------------------------------------- 501
hCoV-19/Ireland/D-NVRL-XIRL00001782/2025|EPI_ISL_19896104|2025-05-14       AAGGCATTCAGTA----------------------------------------------- 501
hCoV-19/Ireland/TA-NVRL-GIRL00039444/2025|EPI_ISL_19896118|2025-05-16      AAGGCATTCAGTA----------------------------------------------- 501
```

```
hCoV-19/Spain/GA-CHUAC-13223/2025|EPI_ISL_19894946|2025-05-31               AAGGCATTCAGTAC---------------------------------------------- 501
hCoV-19/Spain/GA-CHUAC-13215/2025|EPI_ISL_19894939|2025-05-29               AAGGCATTCAGTAC---------------------------------------------- 501
hCoV-19/Finland/THL-02398/2025|EPI_ISL_19875939|2025-05-04                  AAGGCATTCAGTA----------------------------------------------- 501
hCoV-19/Finland/THL-02400/2025|EPI_ISL_19875941|2025-05-03                  AAGGCATTCAGTA----------------------------------------------- 501
hCoV-19/Ireland/LK-UHL-702929/2025|EPI_ISL_19877895|2025-04-16              AAGGCATTCAGTA----------------------------------------------- 501
hCoV-19/Ireland/LK-UHL-703788/2025|EPI_ISL_19877905|2025-05-03              AAGGCATTCAGTA----------------------------------------------- 501
hCoV-19/Ireland/LK-UHL-703851/2025|EPI_ISL_19877907|2025-05-04              AAGGCATTCAGTA----------------------------------------------- 501
hCoV-19/Ireland/LK-UHL-703889/2025|EPI_ISL_19877908|2025-05-05              AAGGCATTCAGTA----------------------------------------------- 501
hCoV-19/Ireland/D-SVUH-FBB21492-49/2025|EPI_ISL_19881756|2025-05-14         AAGGCATTCAGTA----------------------------------------------- 501
hCoV-19/Ireland/D-SVUH-FBB21492-59/2025|EPI_ISL_19881763|2025-05-07         AAGGCATTCAGTA----------------------------------------------- 501
hCoV-19/Spain/CT-HUGTiP-G3R212/2025|EPI_ISL_19883055|2025-05-20             AAGGCATTCAGTA----------------------------------------------- 501
hCoV-19/Ireland/LK-UHL-704398/2025|EPI_ISL_19888621|2025-05-16              AAGGCATTCAGTA----------------------------------------------- 501
hCoV-19/Ireland/G-GUH-N3466/2025|EPI_ISL_19893175|2025-05-03                AAGGCATTCAGTA----------------------------------------------- 501
hCoV-19/Belgium/Sciensano-LS-S5276/2025|EPI_ISL_19893834|2025-04-22         AAGGCATTCAGTA----------------------------------------------- 501
hCoV-19/Italy/VEN-ULSS8-0019244_VI/2025|EPI_ISL_19899362|2025-05-26         AAGGCATTCAGTA----------------------------------------------- 501
hCoV-19/Spain/CT-HUVH-E618109/2025|EPI_ISL_19907773|2025-06-10              AAGGCATTCAGTA----------------------------------------------- 501
hCoV-19/Ireland/D-BH-03819149/2025|EPI_ISL_19911343|2025-05-29              AAGGCATTCAGTA----------------------------------------------- 501
hCoV-19/Ireland/D-BH-03993161/2025|EPI_ISL_19911355|2025-06-10              AAGGCATTCAGTA----------------------------------------------- 501
hCoV-19/Spain/CT-HUVH-E587040/2025|EPI_ISL_19871881|2025-04-26              AAGGCATTCAGTAC---------------------------------------------- 501
hCoV-19/Finland/THL-02392/2025|EPI_ISL_19875934|2025-05-02                  AAGGCATTCAGTAC---------------------------------------------- 501
hCoV-19/Spain/GA-CHUAC-13181/2025|EPI_ISL_19890617|2025-05-20               AAGGCATTCAGTAC---------------------------------------------- 501
hCoV-19/Spain/CT-HUVH-E614843/2025|EPI_ISL_19907763|2025-06-05              AAGGCATTCAGTAC---------------------------------------------- 501
hCoV-19/Italy/UMB-LAOP-172-4-02/2025|EPI_ISL_19899474|2025-05-08            ------------------------------------------------------------ 501
hCoV-19/Spain/NC-CHN-01008932/2025|EPI_ISL_19900595|2025-04-04              ------------------------------------------------------------ 501
hCoV-19/Northern_Ireland/CLIMB-CM7YGDRO/2025|EPI_ISL_19877331|2025-04-14    AA---------------------------------------------------------- 501
hCoV-19/Scotland/CLIMB-CM7YEE96/2025|EPI_ISL_19897754|2025-05-13            AAG--------------------------------------------------------- 501
hCoV-19/France/ARA-HCL125018289001/2025|EPI_ISL_19880005|2025-05-12         AAGGCATTC--------------------------------------------------- 501
hCoV-19/France/ARA-RELAB-HCL725000057501/2025|EPI_ISL_19880014|2025-04-28   AAGGCATTC--------------------------------------------------- 501
hCoV-19/France/ARA-RELAB-HCL725000059501/2025|EPI_ISL_19885160|2025-04-25   AAGGCATTC--------------------------------------------------- 501
hCoV-19/France/NAQ-RELAB-HCL725000061001/2025|EPI_ISL_19885161|2025-05-10   AAGGCATTC--------------------------------------------------- 501
hCoV-19/France/NAQ-RELAB-HCL725000060901/2025|EPI_ISL_19885180|2025-05-09   AAGGCATTC--------------------------------------------------- 501
hCoV-19/France/ARA-HCL025075546601/2025|EPI_ISL_19885182|2025-05-09         AAGGCATTC--------------------------------------------------- 501
hCoV-19/France/ARA-HCL125018461901/2025|EPI_ISL_19885204|2025-05-14         AAGGCATTC--------------------------------------------------- 501
hCoV-19/France/ARA-HCL125018655901/2025|EPI_ISL_19885206|2025-05-14         AAGGCATTC--------------------------------------------------- 501
hCoV-19/France/ARA-HCL125019155501/2025|EPI_ISL_19885209|2025-05-18         AAGGCATTC--------------------------------------------------- 501
hCoV-19/France/ARA-HCL125019240901/2025|EPI_ISL_19885210|2025-05-19         AAGGCATTC--------------------------------------------------- 501
hCoV-19/France/ARA-HCL125019249201/2025|EPI_ISL_19885211|2025-05-19         AAGGCATTC--------------------------------------------------- 501
hCoV-19/France/ARA-HCL125019399701/2025|EPI_ISL_19885212|2025-05-20         AAGGCATTC--------------------------------------------------- 501
hCoV-19/France/ARA-HCL125019408901/2025|EPI_ISL_19885213|2025-05-20         AAGGCATTC--------------------------------------------------- 501
hCoV-19/France/OCC-RELAB-HCL725000064001/2025|EPI_ISL_19895004|2025-05-14   AAGGCATTC--------------------------------------------------- 501
hCoV-19/France/OCC-RELAB-HCL725000064101/2025|EPI_ISL_19895005|2025-05-14   AAGGCATTC--------------------------------------------------- 501
hCoV-19/France/NAQ-RELAB-HCL725000062601/2025|EPI_ISL_19895009|2025-05-16   AAGGCATTC--------------------------------------------------- 501
hCoV-19/France/ARA-RELAB-HCL725000062401/2025|EPI_ISL_19895017|2025-05-16   AAGGCATTC--------------------------------------------------- 501
hCoV-19/France/ARA-RELAB-HCL725000062101/2025|EPI_ISL_19895018|2025-05-13   AAGGCATTC--------------------------------------------------- 501
hCoV-19/France/ARA-HCL125019625101/2025|EPI_ISL_19895034|2025-05-21         AAGGCATTC--------------------------------------------------- 501
hCoV-19/France/ARA-HCL125019754801/2025|EPI_ISL_19895035|2025-05-22         AAGGCATTC--------------------------------------------------- 501
hCoV-19/France/ARA-RELAB-HCL725000064601/2025|EPI_ISL_19895041|2025-05-15   AAGGCATTC--------------------------------------------------- 501
hCoV-19/France/ARA-RELAB-HCL725000066001/2025|EPI_ISL_19895046|2025-05-13   AAGGCATTC--------------------------------------------------- 501
hCoV-19/France/OCC-RELAB-HCL725000068701/2025|EPI_ISL_19905466|2025-05-21   AAGGCATTC--------------------------------------------------- 501
hCoV-19/France/OCC-RELAB-HCL725000069001/2025|EPI_ISL_19905468|2025-05-21   AAGGCATTC--------------------------------------------------- 501
hCoV-19/France/ARA-HCL025089279801/2025|EPI_ISL_19905469|2025-06-02         AAGGCATTC--------------------------------------------------- 501
hCoV-19/France/PAC-RELAB-HCL725000069901/2025|EPI_ISL_19908847|2025-05-26   AAGGCATTC--------------------------------------------------- 501
hCoV-19/France/PAC-RELAB-HCL725000069501/2025|EPI_ISL_19908849|2025-05-30   AAGGCATTC--------------------------------------------------- 501
```

```
hCoV-19/France/ARA-RELAB-HCL725000071701/2025|EPI_ISL_19908858|2025-05-22        AAGGCATTC--------------------------------------------------- 501
hCoV-19/France/ARA-HCL025093352901/2025|EPI_ISL_19908867|2025-06-10              AAGGCATTC--------------------------------------------------- 501
hCoV-19/France/ARA-RELAB-HCL725000065801/2025|EPI_ISL_19895045|2025-05-16        AAGGCATTC--------------------------------------------------- 501
hCoV-19/Denmark/DCGC-692234/2025|EPI_ISL_19879694|2025-05-05                     AAGGCAT----------------------------------------------------- 501
hCoV-19/Finland/THL-02388/2025|EPI_ISL_19875931|2025-04-16                       AAGGCA------------------------------------------------------ 501
hCoV-19/Spain/PV-HUD-98024244/2025|EPI_ISL_19907759|2025-06-09                   AAGGCA------------------------------------------------------ 501
hCoV-19/Netherlands/GE-RIVM-145386/2025|EPI_ISL_19873764|2025-05-01              AAGGCATTC--------------------------------------------------- 501
hCoV-19/Netherlands/ZH-RIVM-145413/2025|EPI_ISL_19891397|2025-04-30              AAGGCATTC--------------------------------------------------- 501
hCoV-19/Netherlands/NH-RIVM-145458/2025|EPI_ISL_19905062|2025-05-25              AAGGCATTC--------------------------------------------------- 501
hCoV-19/Netherlands/NH-RIVM-145463/2025|EPI_ISL_19905063|2025-05-20              AAGGCATTC--------------------------------------------------- 501
hCoV-19/Netherlands/UT-RIVM-145436/2025|EPI_ISL_19905072|2025-05-15              AAGGCATTC--------------------------------------------------- 501
hCoV-19/Netherlands/UT-RIVM-145470/2025|EPI_ISL_19905077|2025-06-02              AAGGCATTC--------------------------------------------------- 501
hCoV-19/Netherlands/ZH-RIVM-145478/2025|EPI_ISL_19905094|2025-05-30              AAGGCATTC--------------------------------------------------- 501
hCoV-19/Netherlands/ZH-RIVM-145480/2025|EPI_ISL_19905096|2025-05-31              AAGGCATTC--------------------------------------------------- 501
hCoV-19/Netherlands/UT-RIVM-145481/2025|EPI_ISL_19905078|2025-05-31              AAGGCATTC--------------------------------------------------- 501
hCoV-19/Scotland/CLIMB-CM7YRW9H/2025|EPI_ISL_19881711|2025-05-06                 AAGGCATTCAGTACGGTCGTAGTGGTGAGACACTTGGTGT-------------------- 501
hCoV-19/France/IDF-RELAB-IPP04872/2025|EPI_ISL_19911293|2025-05-22               AAGGCATTCAGTACGGTCGTAGTGGTGAGACACTTGGTGTC------------------- 501
hCoV-19/Canary_Islands/CN-HUC-925112913/2025|EPI_ISL_19900668|2025-04-22         AAGGCATTCAGTACGGTCGTAGTGGTGAGACAC--------------------------- 501
hCoV-19/Scotland/CLIMB-CM7YJ5BB/2025|EPI_ISL_19906458|2025-05-27                 AAGGCATTCAGTACGGTCGTAGTGGTGAGACACTTGG----------------------- 501
hCoV-19/Scotland/CLIMB-CM7Y8PHH/2025|EPI_ISL_19897755|2025-05-14                 AAGGCATTCAGTACGGTCGTAGTGGTGAGACACTTGG----------------------- 501
hCoV-19/Spain/AN-PMC-42706/2025|EPI_ISL_19888325|2025-05-25                      AAGGCATTCAGTACGGTCGTAGTGGTGAGACACTTGG----------------------- 501
hCoV-19/Scotland/CLIMB-CM7YRIA1/2025|EPI_ISL_19869961|2025-04-20                 AAGGCATTCAGTACGGTCGTAGTGGTGAGACACTTGG----------------------- 501
hCoV-19/Scotland/CLIMB-CM7Y8UWT/2025|EPI_ISL_19869966|2025-04-21                 AAGGCATTCAGTACGGTCGTAGTGGTGAGACACTTGG----------------------- 501
hCoV-19/England/CLIMB-CM7YMFHB/2025|EPI_ISL_19877436|2025-05-10                  AAGGCATTCAGTACGGTCGTAGTGGTGAGACACTTGG----------------------- 501
hCoV-19/Denmark/DCGC-692213/2025|EPI_ISL_19879673|2025-04-28                     AAGGCATTCAGTACGGTCGTAGTGGTGAGACACTTGG----------------------- 501
hCoV-19/Wales/CLIMB-CM7YRAXQ/2025|EPI_ISL_19883075|2025-05-14                    AAGGCATTCAGTACGGTCGTAGTGGTGAGACACTTGG----------------------- 501
hCoV-19/Spain/AN-PMC-42696/2025|EPI_ISL_19888316|2025-05-14                      AAGGCATTCAGTACGGTCGTAGTGGTGAGACACTTGG----------------------- 501
hCoV-19/Spain/AN-PMC-42719/2025|EPI_ISL_19888338|2025-05-15                      AAGGCATTCAGTACGGTCGTAGTGGTGAGACACTTGG----------------------- 501
hCoV-19/England/CLIMB-CM7YE7HB/2025|EPI_ISL_19892115|2025-05-19                  AAGGCATTCAGTACGGTCGTAGTGGTGAGACACTTGG----------------------- 501
hCoV-19/France/GES-RELAB-IPP04599/2025|EPI_ISL_19893547|2025-05-05               AAGGCATTCAGTACGGTCGTAGTGGTGAGACACTTGG----------------------- 501
hCoV-19/Scotland/CLIMB-CM7YEPXZ/2025|EPI_ISL_19896028|2025-05-25                 AAGGCATTCAGTACGGTCGTAGTGGTGAGACACTTGG----------------------- 501
hCoV-19/Scotland/CLIMB-CM7YF4QT/2025|EPI_ISL_19896032|2025-05-27                 AAGGCATTCAGTACGGTCGTAGTGGTGAGACACTTGG----------------------- 501
hCoV-19/Scotland/CLIMB-CM7YKDZ6/2025|EPI_ISL_19896033|2025-05-27                 AAGGCATTCAGTACGGTCGTAGTGGTGAGACACTTGG----------------------- 501
hCoV-19/Scotland/CLIMB-CM7YG8H4/2025|EPI_ISL_19897748|2025-05-02                 AAGGCATTCAGTACGGTCGTAGTGGTGAGACACTTGG----------------------- 501
hCoV-19/Wales/CLIMB-CM7YJXNZ/2025|EPI_ISL_19900036|2025-05-26                    AAGGCATTCAGTACGGTCGTAGTGGTGAGACACTTGG----------------------- 501
hCoV-19/Germany/NW-RKI-I-1148178/2025|EPI_ISL_19902055|2025-05-21                AAGGCATTCAGTACGGTCGTAGTGGTGAGACACTTGG----------------------- 501
hCoV-19/Germany/NI-RKI-I-1148183/2025|EPI_ISL_19902060|2025-05-18                AAGGCATTCAGTACGGTCGTAGTGGTGAGACACTTGG----------------------- 501
hCoV-19/Germany/NW-RKI-I-1148191/2025|EPI_ISL_19902068|2025-05-19                AAGGCATTCAGTACGGTCGTAGTGGTGAGACACTTGG----------------------- 501
hCoV-19/Spain/MD-HGUGM-66414184/2025|EPI_ISL_19906298|2025-05-25                 AAGGCATTCAGTACGGTCGTAGTGGTGAGACACTTGG----------------------- 501
hCoV-19/Spain/MD-HGUGM-66400167/2025|EPI_ISL_19906320|2025-05-22                 AAGGCATTCAGTACGGTCGTAGTGGTGAGACACTTGG----------------------- 501
hCoV-19/France/IDF-RELAB-IPP04646/2025|EPI_ISL_19907194|2025-05-16               AAGGCATTCAGTACGGTCGTAGTGGTGAGACACTTGG----------------------- 501
hCoV-19/England/CLIMB-CM7YKIUQ/2025|EPI_ISL_19908573|2025-06-07                  AAGGCATTCAGTACGGTCGTAGTGGTGAGACACTTGG----------------------- 501
hCoV-19/France/GES-RELAB-IPP04823/2025|EPI_ISL_19911257|2025-05-26               AAGGCATTCAGTACGGTCGTAGTGGTGAGACACTTGG----------------------- 501
hCoV-19/France/IDF-RELAB-IPP04863/2025|EPI_ISL_19911265|2025-05-24               AAGGCATTCAGTACGGTCGTAGTGGTGAGACACTTGG----------------------- 501
hCoV-19/Denmark/DCGC-692245/2025|EPI_ISL_19879704|2025-04-28                     AAGGCATTCAGTACGGTCGTAGTGGTGAGACACTTGG----------------------- 501


hCoV-19/Ireland/D-SVUH-FBA98650-31/2025|EPI_ISL_19866548|2025-05-04              AAATACCAGTGGCTTACCGCAAGGTTCTTCTTCGTAAGAACGGTAATAAAGGAGCTGGTG 204
hCoV-19/Spain/PV-HUB-5628663/2025|EPI_ISL_19908839|2025-05-16                    AAATACCAGTGGCTTACCGCAAGGTTCTTCTTCGTAAGAACGGTAATAAAGGAGCTGGTG 457
hCoV-19/Spain/PV-HUB-41587498/2025|EPI_ISL_19908930|2025-05-20                   AAATACCAGTGGCTTACCGCAAGGTTCTTCTTCGTAAGAACGGTAATAAAGGAGCTGGTG 452
hCoV-19/Spain/PV-HUB-56291851/2025|EPI_ISL_19908970|2025-05-31                   AAATACCAGTGGCTTACCGCAAGGTTCTTCTTCGTAAGAACGGTAATAAAGGAGCTGGTG 453
hCoV-19/Northern_Ireland/CLIMB-CM7YR8I6/2025|EPI_ISL_19877334|2025-04-23         ------------------------------------------------------------ 501
hCoV-19/Germany/NW-RKI-I-1148136/2025|EPI_ISL_19871950|2025-04-23                ------------------------------------------------------------ 501
```

```
hCoV-19/Germany/NW-RKI-I-1148141/2025|EPI_ISL_19871955|2025-04-23          ------------------------------------------------------------ 501
hCoV-19/Germany/BW-RKI-I-1148164/2025|EPI_ISL_19884987|2025-04-17          ------------------------------------------------------------ 501
hCoV-19/Scotland/CLIMB-CM7YFGW7/2025|EPI_ISL_19897691|2025-05-24           ------------------------------------------------------------ 501
hCoV-19/Ireland/G-LUH-N3473/2025|EPI_ISL_19893181|2025-05-14               ------------------------------------------------------------ 501
hCoV-19/Spain/AN-PMC-42728/2025|EPI_ISL_19888347|2025-05-25                ------------------------------------------------------------ 501
hCoV-19/Sweden/000200866492N2/2025|EPI_ISL_19870050|2025-04-22             ------------------------------------------------------------ 501
hCoV-19/Sweden/000200867159N2/2025|EPI_ISL_19870052|2025-04-23             ------------------------------------------------------------ 501
hCoV-19/Ireland/G-LUH-N3445/2025|EPI_ISL_19893154|2025-05-07               ------------------------------------------------------------ 501
hCoV-19/Ireland/G-LUH-N3443/2025|EPI_ISL_19893152|2025-05-07               ------------------------------------------------------------ 501
hCoV-19/Scotland/CLIMB-CM7YF9PJ/2025|EPI_ISL_19895983|2025-05-19           ------------------------------------------------------------ 501
hCoV-19/Scotland/CLIMB-CM7YKMK8/2025|EPI_ISL_19869976|2025-04-25           ------------------------------------------------------------ 501
hCoV-19/Ireland/G-LUH-N3444/2025|EPI_ISL_19893153|2025-05-07               ------------------------------------------------------------ 501
hCoV-19/Switzerland/VD-CHUV-8604552682/2025|EPI_ISL_19906527|2025-05-19    ------------------------------------------------------------ 501
hCoV-19/Scotland/CLIMB-CM7YR3YO/2025|EPI_ISL_19896015|2025-05-24           ------------------------------------------------------------ 501
hCoV-19/Sweden/O-18_SE100_25CS500180/2025|EPI_ISL_19866288|2025-04-15      ------------------------------------------------------------ 501
hCoV-19/Ireland/D-CS250088/2025|EPI_ISL_19877457|2025-04-16                ------------------------------------------------------------ 501
hCoV-19/Ireland/G-LUH-N3442/2025|EPI_ISL_19893151|2025-05-07               ------------------------------------------------------------ 501
hCoV-19/Scotland/CLIMB-CM7YMZ5M/2025|EPI_ISL_19906439|2025-05-22           ------------------------------------------------------------ 501
hCoV-19/Scotland/CLIMB-CM7YR3ZG/2025|EPI_ISL_19897722|2025-05-13           ------------------------------------------------------------ 501
hCoV-19/Scotland/CLIMB-CM7YEYGT/2025|EPI_ISL_19897741|2025-04-28           ------------------------------------------------------------ 501
hCoV-19/Scotland/CLIMB-CM7YGAJ3/2025|EPI_ISL_19895963|2025-05-13           ------------------------------------------------------------ 501
hCoV-19/Scotland/CLIMB-CM7YJ1ZO/2025|EPI_ISL_19897757|2025-05-14           ------------------------------------------------------------ 501
hCoV-19/Scotland/CLIMB-CM7Y8C4Y/2025|EPI_ISL_19906404|2025-05-15           ------------------------------------------------------------ 501
hCoV-19/Northern_Ireland/CLIMB-CM7YGGYM/2025|EPI_ISL_19897641|2025-05-23   ------------------------------------------------------------ 501
hCoV-19/Scotland/CLIMB-CM7YFW7M/2025|EPI_ISL_19881718|2025-05-10           ------------------------------------------------------------ 501
hCoV-19/Scotland/CLIMB-CM7YEMXP/2025|EPI_ISL_19888387|2025-05-10           ------------------------------------------------------------ 501
hCoV-19/Spain/CT-HUVH-E593511/2025|EPI_ISL_19881731|2025-05-06             ------------------------------------------------------------ 501
hCoV-19/Spain/CT-HUJT-E325362/2025|EPI_ISL_19889935|2025-05-20             ------------------------------------------------------------ 501
hCoV-19/Spain/PV-HUD-84743204/2025|EPI_ISL_19907746|2025-06-06             ------------------------------------------------------------ 501
hCoV-19/Ireland/D-SVUH-FBB21492-54/2025|EPI_ISL_19881758|2025-05-11        ------------------------------------------------------------ 501
hCoV-19/Spain/CT-HUVH-E596725/2025|EPI_ISL_19881729|2025-05-10             ------------------------------------------------------------ 501
hCoV-19/Spain/CT-HUVH-E593882/2025|EPI_ISL_19881732|2025-05-06             ------------------------------------------------------------ 501
hCoV-19/Finland/THL-02385/2025|EPI_ISL_19875928|2025-04-16                 ------------------------------------------------------------ 501
hCoV-19/Ireland/D-BH-02351167/2025|EPI_ISL_19911337|2025-05-28             ------------------------------------------------------------ 501
hCoV-19/Ireland/D-NVRL-XIRL00001782/2025|EPI_ISL_19896104|2025-05-14       ------------------------------------------------------------ 501
hCoV-19/Ireland/TA-NVRL-GIRL00039444/2025|EPI_ISL_19896118|2025-05-16      ------------------------------------------------------------ 501
hCoV-19/Spain/GA-CHUAC-13223/2025|EPI_ISL_19894946|2025-05-31              ------------------------------------------------------------ 501
hCoV-19/Spain/GA-CHUAC-13215/2025|EPI_ISL_19894939|2025-05-29              ------------------------------------------------------------ 501
hCoV-19/Finland/THL-02398/2025|EPI_ISL_19875939|2025-05-04                 ------------------------------------------------------------ 501
hCoV-19/Finland/THL-02400/2025|EPI_ISL_19875941|2025-05-03                 ------------------------------------------------------------ 501
hCoV-19/Ireland/LK-UHL-702929/2025|EPI_ISL_19877895|2025-04-16             ------------------------------------------------------------ 501
hCoV-19/Ireland/LK-UHL-703788/2025|EPI_ISL_19877905|2025-05-03             ------------------------------------------------------------ 501
hCoV-19/Ireland/LK-UHL-703851/2025|EPI_ISL_19877907|2025-05-04             ------------------------------------------------------------ 501
hCoV-19/Ireland/LK-UHL-703889/2025|EPI_ISL_19877908|2025-05-05             ------------------------------------------------------------ 501
hCoV-19/Ireland/D-SVUH-FBB21492-49/2025|EPI_ISL_19881756|2025-05-14        ------------------------------------------------------------ 501
hCoV-19/Ireland/D-SVUH-FBB21492-59/2025|EPI_ISL_19881763|2025-05-07        ------------------------------------------------------------ 501
hCoV-19/Spain/CT-HUGTiP-G3R212/2025|EPI_ISL_19883055|2025-05-20            ------------------------------------------------------------ 501
hCoV-19/Ireland/LK-UHL-704398/2025|EPI_ISL_19888621|2025-05-16             ------------------------------------------------------------ 501
hCoV-19/Ireland/G-GUH-N3466/2025|EPI_ISL_19893175|2025-05-03               ------------------------------------------------------------ 501
hCoV-19/Belgium/Sciensano-LS-S5276/2025|EPI_ISL_19893834|2025-04-22        ------------------------------------------------------------ 501
hCoV-19/Italy/VEN-ULSS8-0019244_VI/2025|EPI_ISL_19899362|2025-05-26        ------------------------------------------------------------ 501
hCoV-19/Spain/CT-HUVH-E618109/2025|EPI_ISL_19907773|2025-06-10             ------------------------------------------------------------ 501
hCoV-19/Ireland/D-BH-03819149/2025|EPI_ISL_19911343|2025-05-29             ------------------------------------------------------------ 501
```

```
hCoV-19/Ireland/D-BH-03993161/2025|EPI_ISL_19911355|2025-06-10                  ------------------------------------------------------------ 501
hCoV-19/Spain/CT-HUVH-E587040/2025|EPI_ISL_19871881|2025-04-26                  ------------------------------------------------------------ 501
hCoV-19/Finland/THL-02392/2025|EPI_ISL_19875934|2025-05-02                      ------------------------------------------------------------ 501
hCoV-19/Spain/GA-CHUAC-13181/2025|EPI_ISL_19890617|2025-05-20                   ------------------------------------------------------------ 501
hCoV-19/Spain/CT-HUVH-E614843/2025|EPI_ISL_19907763|2025-06-05                  ------------------------------------------------------------ 501
hCoV-19/Italy/UMB-LAOP-172-4-02/2025|EPI_ISL_19899474|2025-05-08                ------------------------------------------------------------ 501
hCoV-19/Spain/NC-CHN-01008932/2025|EPI_ISL_19900595|2025-04-04                  ------------------------------------------------------------ 501
hCoV-19/Northern_Ireland/CLIMB-CM7YGDRO/2025|EPI_ISL_19877331|2025-04-14        ------------------------------------------------------------ 501
hCoV-19/Scotland/CLIMB-CM7YEE96/2025|EPI_ISL_19897754|2025-05-13                ------------------------------------------------------------ 501
hCoV-19/France/ARA-HCL125018289001/2025|EPI_ISL_19880005|2025-05-12             ------------------------------------------------------------ 501
hCoV-19/France/ARA-RELAB-HCL725000057501/2025|EPI_ISL_19880014|2025-04-28       ------------------------------------------------------------ 501
hCoV-19/France/ARA-RELAB-HCL725000059501/2025|EPI_ISL_19885160|2025-04-25       ------------------------------------------------------------ 501
hCoV-19/France/NAQ-RELAB-HCL725000061001/2025|EPI_ISL_19885161|2025-05-10       ------------------------------------------------------------ 501
hCoV-19/France/NAQ-RELAB-HCL725000060901/2025|EPI_ISL_19885180|2025-05-09       ------------------------------------------------------------ 501
hCoV-19/France/ARA-HCL025075546601/2025|EPI_ISL_19885182|2025-05-09             ------------------------------------------------------------ 501
hCoV-19/France/ARA-HCL125018461901/2025|EPI_ISL_19885204|2025-05-14             ------------------------------------------------------------ 501
hCoV-19/France/ARA-HCL125018655901/2025|EPI_ISL_19885206|2025-05-14             ------------------------------------------------------------ 501
hCoV-19/France/ARA-HCL125019155501/2025|EPI_ISL_19885209|2025-05-18             ------------------------------------------------------------ 501
hCoV-19/France/ARA-HCL125019240901/2025|EPI_ISL_19885210|2025-05-19             ------------------------------------------------------------ 501
hCoV-19/France/ARA-HCL125019249201/2025|EPI_ISL_19885211|2025-05-19             ------------------------------------------------------------ 501
hCoV-19/France/ARA-HCL125019399701/2025|EPI_ISL_19885212|2025-05-20             ------------------------------------------------------------ 501
hCoV-19/France/ARA-HCL125019408901/2025|EPI_ISL_19885213|2025-05-20             ------------------------------------------------------------ 501
hCoV-19/France/OCC-RELAB-HCL725000064001/2025|EPI_ISL_19895004|2025-05-14       ------------------------------------------------------------ 501
hCoV-19/France/OCC-RELAB-HCL725000064101/2025|EPI_ISL_19895005|2025-05-14       ------------------------------------------------------------ 501
hCoV-19/France/NAQ-RELAB-HCL725000062601/2025|EPI_ISL_19895009|2025-05-16       ------------------------------------------------------------ 501
hCoV-19/France/ARA-RELAB-HCL725000062401/2025|EPI_ISL_19895017|2025-05-16       ------------------------------------------------------------ 501
hCoV-19/France/ARA-RELAB-HCL725000062101/2025|EPI_ISL_19895018|2025-05-13       ------------------------------------------------------------ 501
hCoV-19/France/ARA-HCL125019625101/2025|EPI_ISL_19895034|2025-05-21             ------------------------------------------------------------ 501
hCoV-19/France/ARA-HCL125019754801/2025|EPI_ISL_19895035|2025-05-22             ------------------------------------------------------------ 501
hCoV-19/France/ARA-RELAB-HCL725000064601/2025|EPI_ISL_19895041|2025-05-15       ------------------------------------------------------------ 501
hCoV-19/France/ARA-RELAB-HCL725000066001/2025|EPI_ISL_19895046|2025-05-13       ------------------------------------------------------------ 501
hCoV-19/France/OCC-RELAB-HCL725000068701/2025|EPI_ISL_19905466|2025-05-21       ------------------------------------------------------------ 501
hCoV-19/France/OCC-RELAB-HCL725000069001/2025|EPI_ISL_19905468|2025-05-21       ------------------------------------------------------------ 501
hCoV-19/France/ARA-HCL025089279801/2025|EPI_ISL_19905469|2025-06-02             ------------------------------------------------------------ 501
hCoV-19/France/PAC-RELAB-HCL725000069901/2025|EPI_ISL_19908847|2025-05-26       ------------------------------------------------------------ 501
hCoV-19/France/PAC-RELAB-HCL725000069501/2025|EPI_ISL_19908849|2025-05-30       ------------------------------------------------------------ 501
hCoV-19/France/ARA-RELAB-HCL725000071701/2025|EPI_ISL_19908858|2025-05-22       ------------------------------------------------------------ 501
hCoV-19/France/ARA-HCL025093352901/2025|EPI_ISL_19908867|2025-06-10             ------------------------------------------------------------ 501
hCoV-19/France/ARA-RELAB-HCL725000065801/2025|EPI_ISL_19895045|2025-05-16       ------------------------------------------------------------ 501
hCoV-19/Denmark/DCGC-692234/2025|EPI_ISL_19879694|2025-05-05                    ------------------------------------------------------------ 501
hCoV-19/Finland/THL-02388/2025|EPI_ISL_19875931|2025-04-16                      ------------------------------------------------------------ 501
hCoV-19/Spain/PV-HUD-98024244/2025|EPI_ISL_19907759|2025-06-09                  ------------------------------------------------------------ 501
hCoV-19/Netherlands/GE-RIVM-145386/2025|EPI_ISL_19873764|2025-05-01             ------------------------------------------------------------ 501
hCoV-19/Netherlands/ZH-RIVM-145413/2025|EPI_ISL_19891397|2025-04-30             ------------------------------------------------------------ 501
hCoV-19/Netherlands/NH-RIVM-145458/2025|EPI_ISL_19905062|2025-05-25             ------------------------------------------------------------ 501
hCoV-19/Netherlands/NH-RIVM-145463/2025|EPI_ISL_19905063|2025-05-20             ------------------------------------------------------------ 501
hCoV-19/Netherlands/UT-RIVM-145436/2025|EPI_ISL_19905072|2025-05-15             ------------------------------------------------------------ 501
hCoV-19/Netherlands/UT-RIVM-145470/2025|EPI_ISL_19905077|2025-06-02             ------------------------------------------------------------ 501
hCoV-19/Netherlands/ZH-RIVM-145478/2025|EPI_ISL_19905094|2025-05-30             ------------------------------------------------------------ 501
hCoV-19/Netherlands/ZH-RIVM-145480/2025|EPI_ISL_19905096|2025-05-31             ------------------------------------------------------------ 501
hCoV-19/Netherlands/UT-RIVM-145481/2025|EPI_ISL_19905078|2025-05-31             ------------------------------------------------------------ 501
hCoV-19/Scotland/CLIMB-CM7YRW9H/2025|EPI_ISL_19881711|2025-05-06                ------------------------------------------------------------ 501
hCoV-19/France/IDF-RELAB-IPP04872/2025|EPI_ISL_19911293|2025-05-22              ------------------------------------------------------------ 501
```

```
hCoV-19/Canary_Islands/CN-HUC-925112913/2025|EPI_ISL_19900668|2025-04-22       ------------------------------------------------------------ 501
hCoV-19/Scotland/CLIMB-CM7YJ5BB/2025|EPI_ISL_19906458|2025-05-27               ------------------------------------------------------------ 501
hCoV-19/Scotland/CLIMB-CM7Y8PHH/2025|EPI_ISL_19897755|2025-05-14               ------------------------------------------------------------ 501
hCoV-19/Spain/AN-PMC-42706/2025|EPI_ISL_19888325|2025-05-25                    ------------------------------------------------------------ 501
hCoV-19/Scotland/CLIMB-CM7YRIA1/2025|EPI_ISL_19869961|2025-04-20               ------------------------------------------------------------ 501
hCoV-19/Scotland/CLIMB-CM7Y8UWT/2025|EPI_ISL_19869966|2025-04-21               ------------------------------------------------------------ 501
hCoV-19/England/CLIMB-CM7YMFHB/2025|EPI_ISL_19877436|2025-05-10                ------------------------------------------------------------ 501
hCoV-19/Denmark/DCGC-692213/2025|EPI_ISL_19879673|2025-04-28                   ------------------------------------------------------------ 501
hCoV-19/Wales/CLIMB-CM7YRAXQ/2025|EPI_ISL_19883075|2025-05-14                  ------------------------------------------------------------ 501
hCoV-19/Spain/AN-PMC-42696/2025|EPI_ISL_19888316|2025-05-14                    ------------------------------------------------------------ 501
hCoV-19/Spain/AN-PMC-42719/2025|EPI_ISL_19888338|2025-05-15                    ------------------------------------------------------------ 501
hCoV-19/England/CLIMB-CM7YE7HB/2025|EPI_ISL_19892115|2025-05-19                ------------------------------------------------------------ 501
hCoV-19/France/GES-RELAB-IPP04599/2025|EPI_ISL_19893547|2025-05-05             ------------------------------------------------------------ 501
hCoV-19/Scotland/CLIMB-CM7YEPXZ/2025|EPI_ISL_19896028|2025-05-25               ------------------------------------------------------------ 501
hCoV-19/Scotland/CLIMB-CM7YF4QT/2025|EPI_ISL_19896032|2025-05-27               ------------------------------------------------------------ 501
hCoV-19/Scotland/CLIMB-CM7YKDZ6/2025|EPI_ISL_19896033|2025-05-27               ------------------------------------------------------------ 501
hCoV-19/Scotland/CLIMB-CM7YG8H4/2025|EPI_ISL_19897748|2025-05-02               ------------------------------------------------------------ 501
hCoV-19/Wales/CLIMB-CM7YJXNZ/2025|EPI_ISL_19900036|2025-05-26                  ------------------------------------------------------------ 501
hCoV-19/Germany/NW-RKI-I-1148178/2025|EPI_ISL_19902055|2025-05-21              ------------------------------------------------------------ 501
hCoV-19/Germany/NI-RKI-I-1148183/2025|EPI_ISL_19902060|2025-05-18              ------------------------------------------------------------ 501
hCoV-19/Germany/NW-RKI-I-1148191/2025|EPI_ISL_19902068|2025-05-19              ------------------------------------------------------------ 501
hCoV-19/Spain/MD-HGUGM-66414184/2025|EPI_ISL_19906298|2025-05-25               ------------------------------------------------------------ 501
hCoV-19/Spain/MD-HGUGM-66400167/2025|EPI_ISL_19906320|2025-05-22               ------------------------------------------------------------ 501
hCoV-19/France/IDF-RELAB-IPP04646/2025|EPI_ISL_19907194|2025-05-16             ------------------------------------------------------------ 501
hCoV-19/England/CLIMB-CM7YKIUQ/2025|EPI_ISL_19908573|2025-06-07                ------------------------------------------------------------ 501
hCoV-19/France/GES-RELAB-IPP04823/2025|EPI_ISL_19911257|2025-05-26             ------------------------------------------------------------ 501
hCoV-19/France/IDF-RELAB-IPP04863/2025|EPI_ISL_19911265|2025-05-24             ------------------------------------------------------------ 501
hCoV-19/Denmark/DCGC-692245/2025|EPI_ISL_19879704|2025-04-28                   ------------------------------------------------------------ 501


hCoV-19/Ireland/D-SVUH-FBA98650-31/2025|EPI_ISL_19866548|2025-05-04            GCCATAGGTACGGCGCCGATCTAAAGTCATTTGACTTAGGCGACGAGCTTGGCACTGATC 264
hCoV-19/Spain/PV-HUB-5628663/2025|EPI_ISL_19908839|2025-05-16                  GCCATAGGTACGGCGCCGATCTAAAGTCATTTGACTTAGGCGAC---------------- 501
hCoV-19/Spain/PV-HUB-41587498/2025|EPI_ISL_19908930|2025-05-20                 GCCATAGGTACGGCGCCGATCTAAAGTCATTTGACTTAGGCGACGAGCT----------- 501
hCoV-19/Spain/PV-HUB-56291851/2025|EPI_ISL_19908970|2025-05-31                 GCCATAGGTACGGCGCCGATCTAAAGTCATTTGACTTAGGCGACGAGC------------ 501
hCoV-19/Northern_Ireland/CLIMB-CM7YR8I6/2025|EPI_ISL_19877334|2025-04-23       ------------------------------------------------------------ 501
hCoV-19/Germany/NW-RKI-I-1148136/2025|EPI_ISL_19871950|2025-04-23              ------------------------------------------------------------ 501
hCoV-19/Germany/NW-RKI-I-1148141/2025|EPI_ISL_19871955|2025-04-23              ------------------------------------------------------------ 501
hCoV-19/Germany/BW-RKI-I-1148164/2025|EPI_ISL_19884987|2025-04-17              ------------------------------------------------------------ 501
hCoV-19/Scotland/CLIMB-CM7YFGW7/2025|EPI_ISL_19897691|2025-05-24               ------------------------------------------------------------ 501
hCoV-19/Ireland/G-LUH-N3473/2025|EPI_ISL_19893181|2025-05-14                   ------------------------------------------------------------ 501
hCoV-19/Spain/AN-PMC-42728/2025|EPI_ISL_19888347|2025-05-25                    ------------------------------------------------------------ 501
hCoV-19/Sweden/000200866492N2/2025|EPI_ISL_19870050|2025-04-22                 ------------------------------------------------------------ 501
hCoV-19/Sweden/000200867159N2/2025|EPI_ISL_19870052|2025-04-23                 ------------------------------------------------------------ 501
hCoV-19/Ireland/G-LUH-N3445/2025|EPI_ISL_19893154|2025-05-07                   ------------------------------------------------------------ 501
hCoV-19/Ireland/G-LUH-N3443/2025|EPI_ISL_19893152|2025-05-07                   ------------------------------------------------------------ 501
hCoV-19/Scotland/CLIMB-CM7YF9PJ/2025|EPI_ISL_19895983|2025-05-19               ------------------------------------------------------------ 501
hCoV-19/Scotland/CLIMB-CM7YKMK8/2025|EPI_ISL_19869976|2025-04-25               ------------------------------------------------------------ 501
hCoV-19/Ireland/G-LUH-N3444/2025|EPI_ISL_19893153|2025-05-07                   ------------------------------------------------------------ 501
hCoV-19/Switzerland/VD-CHUV-8604552682/2025|EPI_ISL_19906527|2025-05-19        ------------------------------------------------------------ 501
hCoV-19/Scotland/CLIMB-CM7YR3YO/2025|EPI_ISL_19896015|2025-05-24               ------------------------------------------------------------ 501
hCoV-19/Sweden/O-18_SE100_25CS500180/2025|EPI_ISL_19866288|2025-04-15          ------------------------------------------------------------ 501
hCoV-19/Ireland/D-CS250088/2025|EPI_ISL_19877457|2025-04-16                    ------------------------------------------------------------ 501
hCoV-19/Ireland/G-LUH-N3442/2025|EPI_ISL_19893151|2025-05-07                   ------------------------------------------------------------ 501
```

hCoV-19/Scotland/CLIMB-CM7YMZ5M/2025|EPI_ISL_19906439|2025-05-22 -- 501
hCoV-19/Scotland/CLIMB-CM7YR3ZG/2025|EPI_ISL_19897722|2025-05-13 -- 501
hCoV-19/Scotland/CLIMB-CM7YEYGT/2025|EPI_ISL_19897741|2025-04-28 -- 501
hCoV-19/Scotland/CLIMB-CM7YGAJ3/2025|EPI_ISL_19895963|2025-05-13 -- 501
hCoV-19/Scotland/CLIMB-CM7YJ1ZO/2025|EPI_ISL_19897757|2025-05-14 -- 501
hCoV-19/Scotland/CLIMB-CM7Y8C4Y/2025|EPI_ISL_19906404|2025-05-15 -- 501
hCoV-19/Northern_Ireland/CLIMB-CM7YGGYM/2025|EPI_ISL_19897641|2025-05-23 -- 501
hCoV-19/Scotland/CLIMB-CM7YFW7M/2025|EPI_ISL_19881718|2025-05-10 -- 501
hCoV-19/Scotland/CLIMB-CM7YEMXP/2025|EPI_ISL_19888387|2025-05-10 -- 501
hCoV-19/Spain/CT-HUVH-E593511/2025|EPI_ISL_19881731|2025-05-06 -- 501
hCoV-19/Spain/CT-HUJT-E325362/2025|EPI_ISL_19889935|2025-05-20 -- 501
hCoV-19/Spain/PV-HUD-84743204/2025|EPI_ISL_19907746|2025-06-06 -- 501
hCoV-19/Ireland/D-SVUH-FBB21492-54/2025|EPI_ISL_19881758|2025-05-11 -- 501
hCoV-19/Spain/CT-HUVH-E596725/2025|EPI_ISL_19881729|2025-05-10 -- 501
hCoV-19/Spain/CT-HUVH-E593882/2025|EPI_ISL_19881732|2025-05-06 -- 501
hCoV-19/Finland/THL-02385/2025|EPI_ISL_19875928|2025-04-16 -- 501
hCoV-19/Ireland/D-BH-02351167/2025|EPI_ISL_19911337|2025-05-28 -- 501
hCoV-19/Ireland/D-NVRL-XIRL00001782/2025|EPI_ISL_19896104|2025-05-14 -- 501
hCoV-19/Ireland/TA-NVRL-GIRL00039444/2025|EPI_ISL_19896118|2025-05-16 -- 501
hCoV-19/Spain/GA-CHUAC-13223/2025|EPI_ISL_19894946|2025-05-31 -- 501
hCoV-19/Spain/GA-CHUAC-13215/2025|EPI_ISL_19894939|2025-05-29 -- 501
hCoV-19/Finland/THL-02398/2025|EPI_ISL_19875939|2025-05-04 -- 501
hCoV-19/Finland/THL-02400/2025|EPI_ISL_19875941|2025-05-03 -- 501
hCoV-19/Ireland/LK-UHL-702929/2025|EPI_ISL_19877895|2025-04-16 -- 501
hCoV-19/Ireland/LK-UHL-703788/2025|EPI_ISL_19877905|2025-05-03 -- 501
hCoV-19/Ireland/LK-UHL-703851/2025|EPI_ISL_19877907|2025-05-04 -- 501
hCoV-19/Ireland/LK-UHL-703889/2025|EPI_ISL_19877908|2025-05-05 -- 501
hCoV-19/Ireland/D-SVUH-FBB21492-49/2025|EPI_ISL_19881756|2025-05-14 -- 501
hCoV-19/Ireland/D-SVUH-FBB21492-59/2025|EPI_ISL_19881763|2025-05-07 -- 501
hCoV-19/Spain/CT-HUGTiP-G3R212/2025|EPI_ISL_19883055|2025-05-20 -- 501
hCoV-19/Ireland/LK-UHL-704398/2025|EPI_ISL_19888621|2025-05-16 -- 501
hCoV-19/Ireland/G-GUH-N3466/2025|EPI_ISL_19893175|2025-05-03 -- 501
hCoV-19/Belgium/Sciensano-LS-S5276/2025|EPI_ISL_19893834|2025-04-22 -- 501
hCoV-19/Italy/VEN-ULSS8-0019244_VI/2025|EPI_ISL_19899362|2025-05-26 -- 501
hCoV-19/Spain/CT-HUVH-E618109/2025|EPI_ISL_19907773|2025-06-10 -- 501
hCoV-19/Ireland/D-BH-03819149/2025|EPI_ISL_19911343|2025-05-29 -- 501
hCoV-19/Ireland/D-BH-03993161/2025|EPI_ISL_19911355|2025-06-10 -- 501
hCoV-19/Spain/CT-HUVH-E587040/2025|EPI_ISL_19871881|2025-04-26 -- 501
hCoV-19/Finland/THL-02392/2025|EPI_ISL_19875934|2025-05-02 -- 501
hCoV-19/Spain/GA-CHUAC-13181/2025|EPI_ISL_19890617|2025-05-20 -- 501
hCoV-19/Spain/CT-HUVH-E614843/2025|EPI_ISL_19907763|2025-06-05 -- 501
hCoV-19/Italy/UMB-LAOP-172-4-02/2025|EPI_ISL_19899474|2025-05-08 -- 501
hCoV-19/Spain/NC-CHN-01008932/2025|EPI_ISL_19900595|2025-04-04 -- 501
hCoV-19/Northern_Ireland/CLIMB-CM7YGDRO/2025|EPI_ISL_19877331|2025-04-14 -- 501
hCoV-19/Scotland/CLIMB-CM7YEE96/2025|EPI_ISL_19897754|2025-05-13 -- 501
hCoV-19/France/ARA-HCL125018289001/2025|EPI_ISL_19880005|2025-05-12 -- 501
hCoV-19/France/ARA-RELAB-HCL725000057501/2025|EPI_ISL_19880014|2025-04-28 -- 501
hCoV-19/France/ARA-RELAB-HCL725000059501/2025|EPI_ISL_19885160|2025-04-25 -- 501
hCoV-19/France/NAQ-RELAB-HCL725000061001/2025|EPI_ISL_19885161|2025-05-10 -- 501
hCoV-19/France/NAQ-RELAB-HCL725000060901/2025|EPI_ISL_19885180|2025-05-09 -- 501
hCoV-19/France/ARA-HCL025075546601/2025|EPI_ISL_19885182|2025-05-09 -- 501
hCoV-19/France/ARA-HCL125018461901/2025|EPI_ISL_19885204|2025-05-14 -- 501
hCoV-19/France/ARA-HCL125018655901/2025|EPI_ISL_19885206|2025-05-14 -- 501

```
hCoV-19/France/ARA-HCL125019155501/2025|EPI_ISL_19885209|2025-05-18          ------------------------------------------------------------ 501
hCoV-19/France/ARA-HCL125019240901/2025|EPI_ISL_19885210|2025-05-19          ------------------------------------------------------------ 501
hCoV-19/France/ARA-HCL125019249201/2025|EPI_ISL_19885211|2025-05-19          ------------------------------------------------------------ 501
hCoV-19/France/ARA-HCL125019399701/2025|EPI_ISL_19885212|2025-05-20          ------------------------------------------------------------ 501
hCoV-19/France/ARA-HCL125019408901/2025|EPI_ISL_19885213|2025-05-20          ------------------------------------------------------------ 501
hCoV-19/France/OCC-RELAB-HCL725000064001/2025|EPI_ISL_19895004|2025-05-14    ------------------------------------------------------------ 501
hCoV-19/France/OCC-RELAB-HCL725000064101/2025|EPI_ISL_19895005|2025-05-14    ------------------------------------------------------------ 501
hCoV-19/France/NAQ-RELAB-HCL725000062601/2025|EPI_ISL_19895009|2025-05-16    ------------------------------------------------------------ 501
hCoV-19/France/ARA-RELAB-HCL725000062401/2025|EPI_ISL_19895017|2025-05-16    ------------------------------------------------------------ 501
hCoV-19/France/ARA-RELAB-HCL725000062101/2025|EPI_ISL_19895018|2025-05-13    ------------------------------------------------------------ 501
hCoV-19/France/ARA-HCL125019625101/2025|EPI_ISL_19895034|2025-05-21          ------------------------------------------------------------ 501
hCoV-19/France/ARA-HCL125019754801/2025|EPI_ISL_19895035|2025-05-22          ------------------------------------------------------------ 501
hCoV-19/France/ARA-RELAB-HCL725000064601/2025|EPI_ISL_19895041|2025-05-15    ------------------------------------------------------------ 501
hCoV-19/France/ARA-RELAB-HCL725000066001/2025|EPI_ISL_19895046|2025-05-13    ------------------------------------------------------------ 501
hCoV-19/France/OCC-RELAB-HCL725000068701/2025|EPI_ISL_19905466|2025-05-21    ------------------------------------------------------------ 501
hCoV-19/France/OCC-RELAB-HCL725000069001/2025|EPI_ISL_19905468|2025-05-21    ------------------------------------------------------------ 501
hCoV-19/France/ARA-HCL025089279801/2025|EPI_ISL_19905469|2025-06-02          ------------------------------------------------------------ 501
hCoV-19/France/PAC-RELAB-HCL725000069901/2025|EPI_ISL_19908847|2025-05-26    ------------------------------------------------------------ 501
hCoV-19/France/PAC-RELAB-HCL725000069501/2025|EPI_ISL_19908849|2025-05-30    ------------------------------------------------------------ 501
hCoV-19/France/ARA-RELAB-HCL725000071701/2025|EPI_ISL_19908858|2025-05-22    ------------------------------------------------------------ 501
hCoV-19/France/ARA-HCL025093352901/2025|EPI_ISL_19908867|2025-06-10          ------------------------------------------------------------ 501
hCoV-19/France/ARA-RELAB-HCL725000065801/2025|EPI_ISL_19895045|2025-05-16    ------------------------------------------------------------ 501
hCoV-19/Denmark/DCGC-692234/2025|EPI_ISL_19879694|2025-05-05                 ------------------------------------------------------------ 501
hCoV-19/Finland/THL-02388/2025|EPI_ISL_19875931|2025-04-16                   ------------------------------------------------------------ 501
hCoV-19/Spain/PV-HUD-98024244/2025|EPI_ISL_19907759|2025-06-09               ------------------------------------------------------------ 501
hCoV-19/Netherlands/GE-RIVM-145386/2025|EPI_ISL_19873764|2025-05-01          ------------------------------------------------------------ 501
hCoV-19/Netherlands/ZH-RIVM-145413/2025|EPI_ISL_19891397|2025-04-30          ------------------------------------------------------------ 501
hCoV-19/Netherlands/NH-RIVM-145458/2025|EPI_ISL_19905062|2025-05-25          ------------------------------------------------------------ 501
hCoV-19/Netherlands/NH-RIVM-145463/2025|EPI_ISL_19905063|2025-05-20          ------------------------------------------------------------ 501
hCoV-19/Netherlands/UT-RIVM-145436/2025|EPI_ISL_19905072|2025-05-15          ------------------------------------------------------------ 501
hCoV-19/Netherlands/UT-RIVM-145470/2025|EPI_ISL_19905077|2025-06-02          ------------------------------------------------------------ 501
hCoV-19/Netherlands/ZH-RIVM-145478/2025|EPI_ISL_19905094|2025-05-30          ------------------------------------------------------------ 501
hCoV-19/Netherlands/ZH-RIVM-145480/2025|EPI_ISL_19905096|2025-05-31          ------------------------------------------------------------ 501
hCoV-19/Netherlands/UT-RIVM-145481/2025|EPI_ISL_19905078|2025-05-31          ------------------------------------------------------------ 501
hCoV-19/Scotland/CLIMB-CM7YRW9H/2025|EPI_ISL_19881711|2025-05-06             ------------------------------------------------------------ 501
hCoV-19/France/IDF-RELAB-IPP04872/2025|EPI_ISL_19911293|2025-05-22           ------------------------------------------------------------ 501
hCoV-19/Canary_Islands/CN-HUC-925112913/2025|EPI_ISL_19900668|2025-04-22     ------------------------------------------------------------ 501
hCoV-19/Scotland/CLIMB-CM7YJ5BB/2025|EPI_ISL_19906458|2025-05-27             ------------------------------------------------------------ 501
hCoV-19/Scotland/CLIMB-CM7Y8PHH/2025|EPI_ISL_19897755|2025-05-14             ------------------------------------------------------------ 501
hCoV-19/Spain/AN-PMC-42706/2025|EPI_ISL_19888325|2025-05-25                  ------------------------------------------------------------ 501
hCoV-19/Scotland/CLIMB-CM7YRIA1/2025|EPI_ISL_19869961|2025-04-20             ------------------------------------------------------------ 501
hCoV-19/Scotland/CLIMB-CM7Y8UWT/2025|EPI_ISL_19869966|2025-04-21             ------------------------------------------------------------ 501
hCoV-19/England/CLIMB-CM7YMFHB/2025|EPI_ISL_19877436|2025-05-10              ------------------------------------------------------------ 501
hCoV-19/Denmark/DCGC-692213/2025|EPI_ISL_19879673|2025-04-28                 ------------------------------------------------------------ 501
hCoV-19/Wales/CLIMB-CM7YRAXQ/2025|EPI_ISL_19883075|2025-05-14                ------------------------------------------------------------ 501
hCoV-19/Spain/AN-PMC-42696/2025|EPI_ISL_19888316|2025-05-14                  ------------------------------------------------------------ 501
hCoV-19/Spain/AN-PMC-42719/2025|EPI_ISL_19888338|2025-05-15                  ------------------------------------------------------------ 501
hCoV-19/England/CLIMB-CM7YE7HB/2025|EPI_ISL_19892115|2025-05-19              ------------------------------------------------------------ 501
hCoV-19/France/GES-RELAB-IPP04599/2025|EPI_ISL_19893547|2025-05-05           ------------------------------------------------------------ 501
hCoV-19/Scotland/CLIMB-CM7YEPXZ/2025|EPI_ISL_19896028|2025-05-25             ------------------------------------------------------------ 501
hCoV-19/Scotland/CLIMB-CM7YF4QT/2025|EPI_ISL_19896032|2025-05-27             ------------------------------------------------------------ 501
hCoV-19/Scotland/CLIMB-CM7YKDZ6/2025|EPI_ISL_19896033|2025-05-27             ------------------------------------------------------------ 501
hCoV-19/Scotland/CLIMB-CM7YG8H4/2025|EPI_ISL_19897748|2025-05-02             ------------------------------------------------------------ 501
```

```
hCoV-19/Wales/CLIMB-CM7YJXNZ/2025|EPI_ISL_19900036|2025-05-26              ------------------------------------------------------------ 501
hCoV-19/Germany/NW-RKI-I-1148178/2025|EPI_ISL_19902055|2025-05-21          ------------------------------------------------------------ 501
hCoV-19/Germany/NI-RKI-I-1148183/2025|EPI_ISL_19902060|2025-05-18          ------------------------------------------------------------ 501
hCoV-19/Germany/NW-RKI-I-1148191/2025|EPI_ISL_19902068|2025-05-19          ------------------------------------------------------------ 501
hCoV-19/Spain/MD-HGUGM-66414184/2025|EPI_ISL_19906298|2025-05-25           ------------------------------------------------------------ 501
hCoV-19/Spain/MD-HGUGM-66400167/2025|EPI_ISL_19906320|2025-05-22           ------------------------------------------------------------ 501
hCoV-19/France/IDF-RELAB-IPP04646/2025|EPI_ISL_19907194|2025-05-16         ------------------------------------------------------------ 501
hCoV-19/England/CLIMB-CM7YKIUQ/2025|EPI_ISL_19908573|2025-06-07            ------------------------------------------------------------ 501
hCoV-19/France/GES-RELAB-IPP04823/2025|EPI_ISL_19911257|2025-05-26         ------------------------------------------------------------ 501
hCoV-19/France/IDF-RELAB-IPP04863/2025|EPI_ISL_19911265|2025-05-24         ------------------------------------------------------------ 501
hCoV-19/Denmark/DCGC-692245/2025|EPI_ISL_19879704|2025-04-28               ------------------------------------------------------------ 501

hCoV-19/Ireland/D-SVUH-FBA98650-31/2025|EPI_ISL_19866548|2025-05-04        CTTATGAAGATTTTCAAGAAAACTGGAACACTAAACATAGCAGTGGTGTTACCCGTGAAC 324
hCoV-19/Spain/PV-HUB-5628663/2025|EPI_ISL_19908839|2025-05-16              ------------------------------------------------------------ 501
hCoV-19/Spain/PV-HUB-41587498/2025|EPI_ISL_19908930|2025-05-20             ------------------------------------------------------------ 501
hCoV-19/Spain/PV-HUB-56291851/2025|EPI_ISL_19908970|2025-05-31             ------------------------------------------------------------ 501
hCoV-19/Northern_Ireland/CLIMB-CM7YR8I6/2025|EPI_ISL_19877334|2025-04-23   ------------------------------------------------------------ 501
hCoV-19/Germany/NW-RKI-I-1148136/2025|EPI_ISL_19871950|2025-04-23          ------------------------------------------------------------ 501
hCoV-19/Germany/NW-RKI-I-1148141/2025|EPI_ISL_19871955|2025-04-23          ------------------------------------------------------------ 501
hCoV-19/Germany/BW-RKI-I-1148164/2025|EPI_ISL_19884987|2025-04-17          ------------------------------------------------------------ 501
hCoV-19/Scotland/CLIMB-CM7YFGW7/2025|EPI_ISL_19897691|2025-05-24           ------------------------------------------------------------ 501
hCoV-19/Ireland/G-LUH-N3473/2025|EPI_ISL_19893181|2025-05-14               ------------------------------------------------------------ 501
hCoV-19/Spain/AN-PMC-42728/2025|EPI_ISL_19888347|2025-05-25                ------------------------------------------------------------ 501
hCoV-19/Sweden/000200866492N2/2025|EPI_ISL_19870050|2025-04-22             ------------------------------------------------------------ 501
hCoV-19/Sweden/000200867159N2/2025|EPI_ISL_19870052|2025-04-23             ------------------------------------------------------------ 501
hCoV-19/Ireland/G-LUH-N3445/2025|EPI_ISL_19893154|2025-05-07               ------------------------------------------------------------ 501
hCoV-19/Ireland/G-LUH-N3443/2025|EPI_ISL_19893152|2025-05-07               ------------------------------------------------------------ 501
hCoV-19/Scotland/CLIMB-CM7YF9PJ/2025|EPI_ISL_19895983|2025-05-19           ------------------------------------------------------------ 501
hCoV-19/Scotland/CLIMB-CM7YKMK8/2025|EPI_ISL_19869976|2025-04-25           ------------------------------------------------------------ 501
hCoV-19/Ireland/G-LUH-N3444/2025|EPI_ISL_19893153|2025-05-07               ------------------------------------------------------------ 501
hCoV-19/Switzerland/VD-CHUV-8604552682/2025|EPI_ISL_19906527|2025-05-19    ------------------------------------------------------------ 501
hCoV-19/Scotland/CLIMB-CM7YR3YO/2025|EPI_ISL_19896015|2025-05-24           ------------------------------------------------------------ 501
hCoV-19/Sweden/O-18_SE100_25CS500180/2025|EPI_ISL_19866288|2025-04-15      ------------------------------------------------------------ 501
hCoV-19/Ireland/D-CS250088/2025|EPI_ISL_19877457|2025-04-16                ------------------------------------------------------------ 501
hCoV-19/Ireland/G-LUH-N3442/2025|EPI_ISL_19893151|2025-05-07               ------------------------------------------------------------ 501
hCoV-19/Scotland/CLIMB-CM7YMZ5M/2025|EPI_ISL_19906439|2025-05-22           ------------------------------------------------------------ 501
hCoV-19/Scotland/CLIMB-CM7YR3ZG/2025|EPI_ISL_19897722|2025-05-13           ------------------------------------------------------------ 501
hCoV-19/Scotland/CLIMB-CM7YEYGT/2025|EPI_ISL_19897741|2025-04-28           ------------------------------------------------------------ 501
hCoV-19/Scotland/CLIMB-CM7YGAJ3/2025|EPI_ISL_19895963|2025-05-13           ------------------------------------------------------------ 501
hCoV-19/Scotland/CLIMB-CM7YJ1ZO/2025|EPI_ISL_19897757|2025-05-14           ------------------------------------------------------------ 501
hCoV-19/Scotland/CLIMB-CM7Y8C4Y/2025|EPI_ISL_19906404|2025-05-15           ------------------------------------------------------------ 501
hCoV-19/Northern_Ireland/CLIMB-CM7YGGYM/2025|EPI_ISL_19897641|2025-05-23   ------------------------------------------------------------ 501
hCoV-19/Scotland/CLIMB-CM7YFW7M/2025|EPI_ISL_19881718|2025-05-10           ------------------------------------------------------------ 501
hCoV-19/Scotland/CLIMB-CM7YEMXP/2025|EPI_ISL_19888387|2025-05-10           ------------------------------------------------------------ 501
hCoV-19/Spain/CT-HUVH-E593511/2025|EPI_ISL_19881731|2025-05-06             ------------------------------------------------------------ 501
hCoV-19/Spain/CT-HUJT-E325362/2025|EPI_ISL_19889935|2025-05-20             ------------------------------------------------------------ 501
hCoV-19/Spain/PV-HUD-84743204/2025|EPI_ISL_19907746|2025-06-06             ------------------------------------------------------------ 501
hCoV-19/Ireland/D-SVUH-FBB21492-54/2025|EPI_ISL_19881758|2025-05-11        ------------------------------------------------------------ 501
hCoV-19/Spain/CT-HUVH-E596725/2025|EPI_ISL_19881729|2025-05-10             ------------------------------------------------------------ 501
hCoV-19/Spain/CT-HUVH-E593882/2025|EPI_ISL_19881732|2025-05-06             ------------------------------------------------------------ 501
hCoV-19/Finland/THL-02385/2025|EPI_ISL_19875928|2025-04-16                 ------------------------------------------------------------ 501
hCoV-19/Ireland/D-BH-02351167/2025|EPI_ISL_19911337|2025-05-28             ------------------------------------------------------------ 501
```

```
hCoV-19/Ireland/D-NVRL-XIRL00001782/2025|EPI_ISL_19896104|2025-05-14          ------------------------------------------------------------ 501
hCoV-19/Ireland/TA-NVRL-GIRL00039444/2025|EPI_ISL_19896118|2025-05-16         ------------------------------------------------------------ 501
hCoV-19/Spain/GA-CHUAC-13223/2025|EPI_ISL_19894946|2025-05-31                 ------------------------------------------------------------ 501
hCoV-19/Spain/GA-CHUAC-13215/2025|EPI_ISL_19894939|2025-05-29                 ------------------------------------------------------------ 501
hCoV-19/Finland/THL-02398/2025|EPI_ISL_19875939|2025-05-04                    ------------------------------------------------------------ 501
hCoV-19/Finland/THL-02400/2025|EPI_ISL_19875941|2025-05-03                    ------------------------------------------------------------ 501
hCoV-19/Ireland/LK-UHL-702929/2025|EPI_ISL_19877895|2025-04-16                ------------------------------------------------------------ 501
hCoV-19/Ireland/LK-UHL-703788/2025|EPI_ISL_19877905|2025-05-03                ------------------------------------------------------------ 501
hCoV-19/Ireland/LK-UHL-703851/2025|EPI_ISL_19877907|2025-05-04                ------------------------------------------------------------ 501
hCoV-19/Ireland/LK-UHL-703889/2025|EPI_ISL_19877908|2025-05-05                ------------------------------------------------------------ 501
hCoV-19/Ireland/D-SVUH-FBB21492-49/2025|EPI_ISL_19881756|2025-05-14           ------------------------------------------------------------ 501
hCoV-19/Ireland/D-SVUH-FBB21492-59/2025|EPI_ISL_19881763|2025-05-07           ------------------------------------------------------------ 501
hCoV-19/Spain/CT-HUGTiP-G3R212/2025|EPI_ISL_19883055|2025-05-20               ------------------------------------------------------------ 501
hCoV-19/Ireland/LK-UHL-704398/2025|EPI_ISL_19888621|2025-05-16                ------------------------------------------------------------ 501
hCoV-19/Ireland/G-GUH-N3466/2025|EPI_ISL_19893175|2025-05-03                  ------------------------------------------------------------ 501
hCoV-19/Belgium/Sciensano-LS-S5276/2025|EPI_ISL_19893834|2025-04-22           ------------------------------------------------------------ 501
hCoV-19/Italy/VEN-ULSS8-0019244_VI/2025|EPI_ISL_19899362|2025-05-26           ------------------------------------------------------------ 501
hCoV-19/Spain/CT-HUVH-E618109/2025|EPI_ISL_19907773|2025-06-10                ------------------------------------------------------------ 501
hCoV-19/Ireland/D-BH-03819149/2025|EPI_ISL_19911343|2025-05-29                ------------------------------------------------------------ 501
hCoV-19/Ireland/D-BH-03993161/2025|EPI_ISL_19911355|2025-06-10                ------------------------------------------------------------ 501
hCoV-19/Spain/CT-HUVH-E587040/2025|EPI_ISL_19871881|2025-04-26                ------------------------------------------------------------ 501
hCoV-19/Finland/THL-02392/2025|EPI_ISL_19875934|2025-05-02                    ------------------------------------------------------------ 501
hCoV-19/Spain/GA-CHUAC-13181/2025|EPI_ISL_19890617|2025-05-20                 ------------------------------------------------------------ 501
hCoV-19/Spain/CT-HUVH-E614843/2025|EPI_ISL_19907763|2025-06-05                ------------------------------------------------------------ 501
hCoV-19/Italy/UMB-LAOP-172-4-02/2025|EPI_ISL_19899474|2025-05-08              ------------------------------------------------------------ 501
hCoV-19/Spain/NC-CHN-01008932/2025|EPI_ISL_19900595|2025-04-04                ------------------------------------------------------------ 501
hCoV-19/Northern_Ireland/CLIMB-CM7YGDRO/2025|EPI_ISL_19877331|2025-04-14      ------------------------------------------------------------ 501
hCoV-19/Scotland/CLIMB-CM7YEE96/2025|EPI_ISL_19897754|2025-05-13              ------------------------------------------------------------ 501
hCoV-19/France/ARA-HCL125018289001/2025|EPI_ISL_19880005|2025-05-12           ------------------------------------------------------------ 501
hCoV-19/France/ARA-RELAB-HCL725000057501/2025|EPI_ISL_19880014|2025-04-28     ------------------------------------------------------------ 501
hCoV-19/France/ARA-RELAB-HCL725000059501/2025|EPI_ISL_19885160|2025-04-25     ------------------------------------------------------------ 501
hCoV-19/France/NAQ-RELAB-HCL725000061001/2025|EPI_ISL_19885161|2025-05-10     ------------------------------------------------------------ 501
hCoV-19/France/NAQ-RELAB-HCL725000060901/2025|EPI_ISL_19885180|2025-05-09     ------------------------------------------------------------ 501
hCoV-19/France/ARA-HCL025075546601/2025|EPI_ISL_19885182|2025-05-09           ------------------------------------------------------------ 501
hCoV-19/France/ARA-HCL125018461901/2025|EPI_ISL_19885204|2025-05-14           ------------------------------------------------------------ 501
hCoV-19/France/ARA-HCL125018655901/2025|EPI_ISL_19885206|2025-05-14           ------------------------------------------------------------ 501
hCoV-19/France/ARA-HCL125019155501/2025|EPI_ISL_19885209|2025-05-18           ------------------------------------------------------------ 501
hCoV-19/France/ARA-HCL125019240901/2025|EPI_ISL_19885210|2025-05-19           ------------------------------------------------------------ 501
hCoV-19/France/ARA-HCL125019249201/2025|EPI_ISL_19885211|2025-05-19           ------------------------------------------------------------ 501
hCoV-19/France/ARA-HCL125019399701/2025|EPI_ISL_19885212|2025-05-20           ------------------------------------------------------------ 501
hCoV-19/France/ARA-HCL125019408901/2025|EPI_ISL_19885213|2025-05-20           ------------------------------------------------------------ 501
hCoV-19/France/OCC-RELAB-HCL725000064001/2025|EPI_ISL_19895004|2025-05-14     ------------------------------------------------------------ 501
hCoV-19/France/OCC-RELAB-HCL725000064101/2025|EPI_ISL_19895005|2025-05-14     ------------------------------------------------------------ 501
hCoV-19/France/NAQ-RELAB-HCL725000062601/2025|EPI_ISL_19895009|2025-05-16     ------------------------------------------------------------ 501
hCoV-19/France/ARA-RELAB-HCL725000062401/2025|EPI_ISL_19895017|2025-05-16     ------------------------------------------------------------ 501
hCoV-19/France/ARA-RELAB-HCL725000062101/2025|EPI_ISL_19895018|2025-05-13     ------------------------------------------------------------ 501
hCoV-19/France/ARA-HCL125019625101/2025|EPI_ISL_19895034|2025-05-21           ------------------------------------------------------------ 501
hCoV-19/France/ARA-HCL125019754801/2025|EPI_ISL_19895035|2025-05-22           ------------------------------------------------------------ 501
hCoV-19/France/ARA-RELAB-HCL725000064601/2025|EPI_ISL_19895041|2025-05-15     ------------------------------------------------------------ 501
hCoV-19/France/ARA-RELAB-HCL725000066001/2025|EPI_ISL_19895046|2025-05-13     ------------------------------------------------------------ 501
hCoV-19/France/OCC-RELAB-HCL725000068701/2025|EPI_ISL_19905466|2025-05-21     ------------------------------------------------------------ 501
hCoV-19/France/OCC-RELAB-HCL725000069001/2025|EPI_ISL_19905468|2025-05-21     ------------------------------------------------------------ 501
hCoV-19/France/ARA-HCL025089279801/2025|EPI_ISL_19905469|2025-06-02           ------------------------------------------------------------ 501
```

```
hCoV-19/France/PAC-RELAB-HCL725000069901/2025|EPI_ISL_19908847|2025-05-26      ------------------------------------------------------------ 501
hCoV-19/France/PAC-RELAB-HCL725000069501/2025|EPI_ISL_19908849|2025-05-30      ------------------------------------------------------------ 501
hCoV-19/France/ARA-RELAB-HCL725000071701/2025|EPI_ISL_19908858|2025-05-22      ------------------------------------------------------------ 501
hCoV-19/France/ARA-HCL025093352901/2025|EPI_ISL_19908867|2025-06-10            ------------------------------------------------------------ 501
hCoV-19/France/ARA-RELAB-HCL725000065801/2025|EPI_ISL_19895045|2025-05-16      ------------------------------------------------------------ 501
hCoV-19/Denmark/DCGC-692234/2025|EPI_ISL_19879694|2025-05-05                   ------------------------------------------------------------ 501
hCoV-19/Finland/THL-02388/2025|EPI_ISL_19875931|2025-04-16                     ------------------------------------------------------------ 501
hCoV-19/Spain/PV-HUD-98024244/2025|EPI_ISL_19907759|2025-06-09                 ------------------------------------------------------------ 501
hCoV-19/Netherlands/GE-RIVM-145386/2025|EPI_ISL_19873764|2025-05-01            ------------------------------------------------------------ 501
hCoV-19/Netherlands/ZH-RIVM-145413/2025|EPI_ISL_19891397|2025-04-30            ------------------------------------------------------------ 501
hCoV-19/Netherlands/NH-RIVM-145458/2025|EPI_ISL_19905062|2025-05-25            ------------------------------------------------------------ 501
hCoV-19/Netherlands/NH-RIVM-145463/2025|EPI_ISL_19905063|2025-05-20            ------------------------------------------------------------ 501
hCoV-19/Netherlands/UT-RIVM-145436/2025|EPI_ISL_19905072|2025-05-15            ------------------------------------------------------------ 501
hCoV-19/Netherlands/UT-RIVM-145470/2025|EPI_ISL_19905077|2025-06-02            ------------------------------------------------------------ 501
hCoV-19/Netherlands/ZH-RIVM-145478/2025|EPI_ISL_19905094|2025-05-30            ------------------------------------------------------------ 501
hCoV-19/Netherlands/ZH-RIVM-145480/2025|EPI_ISL_19905096|2025-05-31            ------------------------------------------------------------ 501
hCoV-19/Netherlands/UT-RIVM-145481/2025|EPI_ISL_19905078|2025-05-31            ------------------------------------------------------------ 501
hCoV-19/Scotland/CLIMB-CM7YRW9H/2025|EPI_ISL_19881711|2025-05-06               ------------------------------------------------------------ 501
hCoV-19/France/IDF-RELAB-IPP04872/2025|EPI_ISL_19911293|2025-05-22             ------------------------------------------------------------ 501
hCoV-19/Canary_Islands/CN-HUC-925112913/2025|EPI_ISL_19900668|2025-04-22       ------------------------------------------------------------ 501
hCoV-19/Scotland/CLIMB-CM7YJ5BB/2025|EPI_ISL_19906458|2025-05-27               ------------------------------------------------------------ 501
hCoV-19/Scotland/CLIMB-CM7Y8PHH/2025|EPI_ISL_19897755|2025-05-14               ------------------------------------------------------------ 501
hCoV-19/Spain/AN-PMC-42706/2025|EPI_ISL_19888325|2025-05-25                    ------------------------------------------------------------ 501
hCoV-19/Scotland/CLIMB-CM7YRIA1/2025|EPI_ISL_19869961|2025-04-20               ------------------------------------------------------------ 501
hCoV-19/Scotland/CLIMB-CM7Y8UWT/2025|EPI_ISL_19869966|2025-04-21               ------------------------------------------------------------ 501
hCoV-19/England/CLIMB-CM7YMFHB/2025|EPI_ISL_19877436|2025-05-10                ------------------------------------------------------------ 501
hCoV-19/Denmark/DCGC-692213/2025|EPI_ISL_19879673|2025-04-28                   ------------------------------------------------------------ 501
hCoV-19/Wales/CLIMB-CM7YRAXQ/2025|EPI_ISL_19883075|2025-05-14                  ------------------------------------------------------------ 501
hCoV-19/Spain/AN-PMC-42696/2025|EPI_ISL_19888316|2025-05-14                    ------------------------------------------------------------ 501
hCoV-19/Spain/AN-PMC-42719/2025|EPI_ISL_19888338|2025-05-15                    ------------------------------------------------------------ 501
hCoV-19/England/CLIMB-CM7YE7HB/2025|EPI_ISL_19892115|2025-05-19                ------------------------------------------------------------ 501
hCoV-19/France/GES-RELAB-IPP04599/2025|EPI_ISL_19893547|2025-05-05             ------------------------------------------------------------ 501
hCoV-19/Scotland/CLIMB-CM7YEPXZ/2025|EPI_ISL_19896028|2025-05-25               ------------------------------------------------------------ 501
hCoV-19/Scotland/CLIMB-CM7YF4QT/2025|EPI_ISL_19896032|2025-05-27               ------------------------------------------------------------ 501
hCoV-19/Scotland/CLIMB-CM7YKDZ6/2025|EPI_ISL_19896033|2025-05-27               ------------------------------------------------------------ 501
hCoV-19/Scotland/CLIMB-CM7YG8H4/2025|EPI_ISL_19897748|2025-05-02               ------------------------------------------------------------ 501
hCoV-19/Wales/CLIMB-CM7YJXNZ/2025|EPI_ISL_19900036|2025-05-26                  ------------------------------------------------------------ 501
hCoV-19/Germany/NW-RKI-I-1148178/2025|EPI_ISL_19902055|2025-05-21              ------------------------------------------------------------ 501
hCoV-19/Germany/NI-RKI-I-1148183/2025|EPI_ISL_19902060|2025-05-18              ------------------------------------------------------------ 501
hCoV-19/Germany/NW-RKI-I-1148191/2025|EPI_ISL_19902068|2025-05-19              ------------------------------------------------------------ 501
hCoV-19/Spain/MD-HGUGM-66414184/2025|EPI_ISL_19906298|2025-05-25               ------------------------------------------------------------ 501
hCoV-19/Spain/MD-HGUGM-66400167/2025|EPI_ISL_19906320|2025-05-22               ------------------------------------------------------------ 501
hCoV-19/France/IDF-RELAB-IPP04646/2025|EPI_ISL_19907194|2025-05-16             ------------------------------------------------------------ 501
hCoV-19/England/CLIMB-CM7YKIUQ/2025|EPI_ISL_19908573|2025-06-07                ------------------------------------------------------------ 501
hCoV-19/France/GES-RELAB-IPP04823/2025|EPI_ISL_19911257|2025-05-26             ------------------------------------------------------------ 501
hCoV-19/France/IDF-RELAB-IPP04863/2025|EPI_ISL_19911265|2025-05-24             ------------------------------------------------------------ 501
hCoV-19/Denmark/DCGC-692245/2025|EPI_ISL_19879704|2025-04-28                   ------------------------------------------------------------ 501

hCoV-19/Ireland/D-SVUH-FBA98650-31/2025|EPI_ISL_19866548|2025-05-04            TCATGCGTGAGCTTAACGGAGGGGCATACACTCGCTATGTTGATAACAACTTCTGTGGCC 384
hCoV-19/Spain/PV-HUB-5628663/2025|EPI_ISL_19908839|2025-05-16                  ------------------------------------------------------------ 501
hCoV-19/Spain/PV-HUB-41587498/2025|EPI_ISL_19908930|2025-05-20                 ------------------------------------------------------------ 501
hCoV-19/Spain/PV-HUB-56291851/2025|EPI_ISL_19908970|2025-05-31                 ------------------------------------------------------------ 501
```

```
hCoV-19/Northern_Ireland/CLIMB-CM7YR8I6/2025|EPI_ISL_19877334|2025-04-23       ------------------------------------------------------------ 501
hCoV-19/Germany/NW-RKI-I-1148136/2025|EPI_ISL_19871950|2025-04-23              ------------------------------------------------------------ 501
hCoV-19/Germany/NW-RKI-I-1148141/2025|EPI_ISL_19871955|2025-04-23              ------------------------------------------------------------ 501
hCoV-19/Germany/BW-RKI-I-1148164/2025|EPI_ISL_19884987|2025-04-17              ------------------------------------------------------------ 501
hCoV-19/Scotland/CLIMB-CM7YFGW7/2025|EPI_ISL_19897691|2025-05-24               ------------------------------------------------------------ 501
hCoV-19/Ireland/G-LUH-N3473/2025|EPI_ISL_19893181|2025-05-14                   ------------------------------------------------------------ 501
hCoV-19/Spain/AN-PMC-42728/2025|EPI_ISL_19888347|2025-05-25                    ------------------------------------------------------------ 501
hCoV-19/Sweden/000200866492N2/2025|EPI_ISL_19870050|2025-04-22                 ------------------------------------------------------------ 501
hCoV-19/Sweden/000200867159N2/2025|EPI_ISL_19870052|2025-04-23                 ------------------------------------------------------------ 501
hCoV-19/Ireland/G-LUH-N3445/2025|EPI_ISL_19893154|2025-05-07                   ------------------------------------------------------------ 501
hCoV-19/Ireland/G-LUH-N3443/2025|EPI_ISL_19893152|2025-05-07                   ------------------------------------------------------------ 501
hCoV-19/Scotland/CLIMB-CM7YF9PJ/2025|EPI_ISL_19895983|2025-05-19               ------------------------------------------------------------ 501
hCoV-19/Scotland/CLIMB-CM7YKMK8/2025|EPI_ISL_19869976|2025-04-25               ------------------------------------------------------------ 501
hCoV-19/Ireland/G-LUH-N3444/2025|EPI_ISL_19893153|2025-05-07                   ------------------------------------------------------------ 501
hCoV-19/Switzerland/VD-CHUV-8604552682/2025|EPI_ISL_19906527|2025-05-19        ------------------------------------------------------------ 501
hCoV-19/Scotland/CLIMB-CM7YR3YO/2025|EPI_ISL_19896015|2025-05-24               ------------------------------------------------------------ 501
hCoV-19/Sweden/O-18_SE100_25CS500180/2025|EPI_ISL_19866288|2025-04-15          ------------------------------------------------------------ 501
hCoV-19/Ireland/D-CS250088/2025|EPI_ISL_19877457|2025-04-16                    ------------------------------------------------------------ 501
hCoV-19/Ireland/G-LUH-N3442/2025|EPI_ISL_19893151|2025-05-07                   ------------------------------------------------------------ 501
hCoV-19/Scotland/CLIMB-CM7YMZ5M/2025|EPI_ISL_19906439|2025-05-22               ------------------------------------------------------------ 501
hCoV-19/Scotland/CLIMB-CM7YR3ZG/2025|EPI_ISL_19897722|2025-05-13               ------------------------------------------------------------ 501
hCoV-19/Scotland/CLIMB-CM7YEYGT/2025|EPI_ISL_19897741|2025-04-28               ------------------------------------------------------------ 501
hCoV-19/Scotland/CLIMB-CM7YGAJ3/2025|EPI_ISL_19895963|2025-05-13               ------------------------------------------------------------ 501
hCoV-19/Scotland/CLIMB-CM7YJ1ZO/2025|EPI_ISL_19897757|2025-05-14               ------------------------------------------------------------ 501
hCoV-19/Scotland/CLIMB-CM7Y8C4Y/2025|EPI_ISL_19906404|2025-05-15               ------------------------------------------------------------ 501
hCoV-19/Northern_Ireland/CLIMB-CM7YGGYM/2025|EPI_ISL_19897641|2025-05-23       ------------------------------------------------------------ 501
hCoV-19/Scotland/CLIMB-CM7YFW7M/2025|EPI_ISL_19881718|2025-05-10               ------------------------------------------------------------ 501
hCoV-19/Scotland/CLIMB-CM7YEMXP/2025|EPI_ISL_19888387|2025-05-10               ------------------------------------------------------------ 501
hCoV-19/Spain/CT-HUVH-E593511/2025|EPI_ISL_19881731|2025-05-06                 ------------------------------------------------------------ 501
hCoV-19/Spain/CT-HUJT-E325362/2025|EPI_ISL_19889935|2025-05-20                 ------------------------------------------------------------ 501
hCoV-19/Spain/PV-HUD-84743204/2025|EPI_ISL_19907746|2025-06-06                 ------------------------------------------------------------ 501
hCoV-19/Ireland/D-SVUH-FBB21492-54/2025|EPI_ISL_19881758|2025-05-11            ------------------------------------------------------------ 501
hCoV-19/Spain/CT-HUVH-E596725/2025|EPI_ISL_19881729|2025-05-10                 ------------------------------------------------------------ 501
hCoV-19/Spain/CT-HUVH-E593882/2025|EPI_ISL_19881732|2025-05-06                 ------------------------------------------------------------ 501
hCoV-19/Finland/THL-02385/2025|EPI_ISL_19875928|2025-04-16                     ------------------------------------------------------------ 501
hCoV-19/Ireland/D-BH-02351167/2025|EPI_ISL_19911337|2025-05-28                 ------------------------------------------------------------ 501
hCoV-19/Ireland/D-NVRL-XIRL00001782/2025|EPI_ISL_19896104|2025-05-14           ------------------------------------------------------------ 501
hCoV-19/Ireland/TA-NVRL-GIRL00039444/2025|EPI_ISL_19896118|2025-05-16          ------------------------------------------------------------ 501
hCoV-19/Spain/GA-CHUAC-13223/2025|EPI_ISL_19894946|2025-05-31                  ------------------------------------------------------------ 501
hCoV-19/Spain/GA-CHUAC-13215/2025|EPI_ISL_19894939|2025-05-29                  ------------------------------------------------------------ 501
hCoV-19/Finland/THL-02398/2025|EPI_ISL_19875939|2025-05-04                     ------------------------------------------------------------ 501
hCoV-19/Finland/THL-02400/2025|EPI_ISL_19875941|2025-05-03                     ------------------------------------------------------------ 501
hCoV-19/Ireland/LK-UHL-702929/2025|EPI_ISL_19877895|2025-04-16                 ------------------------------------------------------------ 501
hCoV-19/Ireland/LK-UHL-703788/2025|EPI_ISL_19877905|2025-05-03                 ------------------------------------------------------------ 501
hCoV-19/Ireland/LK-UHL-703851/2025|EPI_ISL_19877907|2025-05-04                 ------------------------------------------------------------ 501
hCoV-19/Ireland/LK-UHL-703889/2025|EPI_ISL_19877908|2025-05-05                 ------------------------------------------------------------ 501
hCoV-19/Ireland/D-SVUH-FBB21492-49/2025|EPI_ISL_19881756|2025-05-14            ------------------------------------------------------------ 501
hCoV-19/Ireland/D-SVUH-FBB21492-59/2025|EPI_ISL_19881763|2025-05-07            ------------------------------------------------------------ 501
hCoV-19/Spain/CT-HUGTiP-G3R212/2025|EPI_ISL_19883055|2025-05-20                ------------------------------------------------------------ 501
hCoV-19/Ireland/LK-UHL-704398/2025|EPI_ISL_19888621|2025-05-16                 ------------------------------------------------------------ 501
hCoV-19/Ireland/G-GUH-N3466/2025|EPI_ISL_19893175|2025-05-03                   ------------------------------------------------------------ 501
hCoV-19/Belgium/Sciensano-LS-S5276/2025|EPI_ISL_19893834|2025-04-22            ------------------------------------------------------------ 501
hCoV-19/Italy/VEN-ULSS8-0019244_VI/2025|EPI_ISL_19899362|2025-05-26            ------------------------------------------------------------ 501
```

```
hCoV-19/Spain/CT-HUVH-E618109/2025|EPI_ISL_19907773|2025-06-10                  ------------------------------------------------------------ 501
hCoV-19/Ireland/D-BH-03819149/2025|EPI_ISL_19911343|2025-05-29                  ------------------------------------------------------------ 501
hCoV-19/Ireland/D-BH-03993161/2025|EPI_ISL_19911355|2025-06-10                  ------------------------------------------------------------ 501
hCoV-19/Spain/CT-HUVH-E587040/2025|EPI_ISL_19871881|2025-04-26                  ------------------------------------------------------------ 501
hCoV-19/Finland/THL-02392/2025|EPI_ISL_19875934|2025-05-02                      ------------------------------------------------------------ 501
hCoV-19/Spain/GA-CHUAC-13181/2025|EPI_ISL_19890617|2025-05-20                   ------------------------------------------------------------ 501
hCoV-19/Spain/CT-HUVH-E614843/2025|EPI_ISL_19907763|2025-06-05                  ------------------------------------------------------------ 501
hCoV-19/Italy/UMB-LAOP-172-4-02/2025|EPI_ISL_19899474|2025-05-08                ------------------------------------------------------------ 501
hCoV-19/Spain/NC-CHN-01008932/2025|EPI_ISL_19900595|2025-04-04                  ------------------------------------------------------------ 501
hCoV-19/Northern_Ireland/CLIMB-CM7YGDRO/2025|EPI_ISL_19877331|2025-04-14        ------------------------------------------------------------ 501
hCoV-19/Scotland/CLIMB-CM7YEE96/2025|EPI_ISL_19897754|2025-05-13                ------------------------------------------------------------ 501
hCoV-19/France/ARA-HCL125018289001/2025|EPI_ISL_19880005|2025-05-12             ------------------------------------------------------------ 501
hCoV-19/France/ARA-RELAB-HCL725000057501/2025|EPI_ISL_19880014|2025-04-28       ------------------------------------------------------------ 501
hCoV-19/France/ARA-RELAB-HCL725000059501/2025|EPI_ISL_19885160|2025-04-25       ------------------------------------------------------------ 501
hCoV-19/France/NAQ-RELAB-HCL725000061001/2025|EPI_ISL_19885161|2025-05-10       ------------------------------------------------------------ 501
hCoV-19/France/NAQ-RELAB-HCL725000060901/2025|EPI_ISL_19885180|2025-05-09       ------------------------------------------------------------ 501
hCoV-19/France/ARA-HCL025075546601/2025|EPI_ISL_19885182|2025-05-09             ------------------------------------------------------------ 501
hCoV-19/France/ARA-HCL125018461901/2025|EPI_ISL_19885204|2025-05-14             ------------------------------------------------------------ 501
hCoV-19/France/ARA-HCL125018655901/2025|EPI_ISL_19885206|2025-05-14             ------------------------------------------------------------ 501
hCoV-19/France/ARA-HCL125019155501/2025|EPI_ISL_19885209|2025-05-18             ------------------------------------------------------------ 501
hCoV-19/France/ARA-HCL125019240901/2025|EPI_ISL_19885210|2025-05-19             ------------------------------------------------------------ 501
hCoV-19/France/ARA-HCL125019249201/2025|EPI_ISL_19885211|2025-05-19             ------------------------------------------------------------ 501
hCoV-19/France/ARA-HCL125019399701/2025|EPI_ISL_19885212|2025-05-20             ------------------------------------------------------------ 501
hCoV-19/France/ARA-HCL125019408901/2025|EPI_ISL_19885213|2025-05-20             ------------------------------------------------------------ 501
hCoV-19/France/OCC-RELAB-HCL725000064001/2025|EPI_ISL_19895004|2025-05-14       ------------------------------------------------------------ 501
hCoV-19/France/OCC-RELAB-HCL725000064101/2025|EPI_ISL_19895005|2025-05-14       ------------------------------------------------------------ 501
hCoV-19/France/NAQ-RELAB-HCL725000062601/2025|EPI_ISL_19895009|2025-05-16       ------------------------------------------------------------ 501
hCoV-19/France/ARA-RELAB-HCL725000062401/2025|EPI_ISL_19895017|2025-05-16       ------------------------------------------------------------ 501
hCoV-19/France/ARA-RELAB-HCL725000062101/2025|EPI_ISL_19895018|2025-05-13       ------------------------------------------------------------ 501
hCoV-19/France/ARA-HCL125019625101/2025|EPI_ISL_19895034|2025-05-21             ------------------------------------------------------------ 501
hCoV-19/France/ARA-HCL125019754801/2025|EPI_ISL_19895035|2025-05-22             ------------------------------------------------------------ 501
hCoV-19/France/ARA-RELAB-HCL725000064601/2025|EPI_ISL_19895041|2025-05-15       ------------------------------------------------------------ 501
hCoV-19/France/ARA-RELAB-HCL725000066001/2025|EPI_ISL_19895046|2025-05-13       ------------------------------------------------------------ 501
hCoV-19/France/OCC-RELAB-HCL725000068701/2025|EPI_ISL_19905466|2025-05-21       ------------------------------------------------------------ 501
hCoV-19/France/OCC-RELAB-HCL725000069001/2025|EPI_ISL_19905468|2025-05-21       ------------------------------------------------------------ 501
hCoV-19/France/ARA-HCL025089279801/2025|EPI_ISL_19905469|2025-06-02             ------------------------------------------------------------ 501
hCoV-19/France/PAC-RELAB-HCL725000069901/2025|EPI_ISL_19908847|2025-05-26       ------------------------------------------------------------ 501
hCoV-19/France/PAC-RELAB-HCL725000069501/2025|EPI_ISL_19908849|2025-05-30       ------------------------------------------------------------ 501
hCoV-19/France/ARA-RELAB-HCL725000071701/2025|EPI_ISL_19908858|2025-05-22       ------------------------------------------------------------ 501
hCoV-19/France/ARA-HCL025093352901/2025|EPI_ISL_19908867|2025-06-10             ------------------------------------------------------------ 501
hCoV-19/France/ARA-RELAB-HCL725000065801/2025|EPI_ISL_19895045|2025-05-16       ------------------------------------------------------------ 501
hCoV-19/Denmark/DCGC-692234/2025|EPI_ISL_19879694|2025-05-05                    ------------------------------------------------------------ 501
hCoV-19/Finland/THL-02388/2025|EPI_ISL_19875931|2025-04-16                      ------------------------------------------------------------ 501
hCoV-19/Spain/PV-HUD-98024244/2025|EPI_ISL_19907759|2025-06-09                  ------------------------------------------------------------ 501
hCoV-19/Netherlands/GE-RIVM-145386/2025|EPI_ISL_19873764|2025-05-01             ------------------------------------------------------------ 501
hCoV-19/Netherlands/ZH-RIVM-145413/2025|EPI_ISL_19891397|2025-04-30             ------------------------------------------------------------ 501
hCoV-19/Netherlands/NH-RIVM-145458/2025|EPI_ISL_19905062|2025-05-25             ------------------------------------------------------------ 501
hCoV-19/Netherlands/NH-RIVM-145463/2025|EPI_ISL_19905063|2025-05-20             ------------------------------------------------------------ 501
hCoV-19/Netherlands/UT-RIVM-145436/2025|EPI_ISL_19905072|2025-05-15             ------------------------------------------------------------ 501
hCoV-19/Netherlands/UT-RIVM-145470/2025|EPI_ISL_19905077|2025-06-02             ------------------------------------------------------------ 501
hCoV-19/Netherlands/ZH-RIVM-145478/2025|EPI_ISL_19905094|2025-05-30             ------------------------------------------------------------ 501
hCoV-19/Netherlands/ZH-RIVM-145480/2025|EPI_ISL_19905096|2025-05-31             ------------------------------------------------------------ 501
hCoV-19/Netherlands/UT-RIVM-145481/2025|EPI_ISL_19905078|2025-05-31             ------------------------------------------------------------ 501
```

```
hCoV-19/Scotland/CLIMB-CM7YRW9H/2025|EPI_ISL_19881711|2025-05-06          ------------------------------------------------------------ 501
hCoV-19/France/IDF-RELAB-IPP04872/2025|EPI_ISL_19911293|2025-05-22        ------------------------------------------------------------ 501
hCoV-19/Canary_Islands/CN-HUC-925112913/2025|EPI_ISL_19900668|2025-04-22  ------------------------------------------------------------ 501
hCoV-19/Scotland/CLIMB-CM7YJ5BB/2025|EPI_ISL_19906458|2025-05-27          ------------------------------------------------------------ 501
hCoV-19/Scotland/CLIMB-CM7Y8PHH/2025|EPI_ISL_19897755|2025-05-14          ------------------------------------------------------------ 501
hCoV-19/Spain/AN-PMC-42706/2025|EPI_ISL_19888325|2025-05-25               ------------------------------------------------------------ 501
hCoV-19/Scotland/CLIMB-CM7YRIA1/2025|EPI_ISL_19869961|2025-04-20          ------------------------------------------------------------ 501
hCoV-19/Scotland/CLIMB-CM7Y8UWT/2025|EPI_ISL_19869966|2025-04-21          ------------------------------------------------------------ 501
hCoV-19/England/CLIMB-CM7YMFHB/2025|EPI_ISL_19877436|2025-05-10           ------------------------------------------------------------ 501
hCoV-19/Denmark/DCGC-692213/2025|EPI_ISL_19879673|2025-04-28              ------------------------------------------------------------ 501
hCoV-19/Wales/CLIMB-CM7YRAXQ/2025|EPI_ISL_19883075|2025-05-14             ------------------------------------------------------------ 501
hCoV-19/Spain/AN-PMC-42696/2025|EPI_ISL_19888316|2025-05-14               ------------------------------------------------------------ 501
hCoV-19/Spain/AN-PMC-42719/2025|EPI_ISL_19888338|2025-05-15               ------------------------------------------------------------ 501
hCoV-19/England/CLIMB-CM7YE7HB/2025|EPI_ISL_19892115|2025-05-19           ------------------------------------------------------------ 501
hCoV-19/France/GES-RELAB-IPP04599/2025|EPI_ISL_19893547|2025-05-05        ------------------------------------------------------------ 501
hCoV-19/Scotland/CLIMB-CM7YEPXZ/2025|EPI_ISL_19896028|2025-05-25          ------------------------------------------------------------ 501
hCoV-19/Scotland/CLIMB-CM7YF4QT/2025|EPI_ISL_19896032|2025-05-27          ------------------------------------------------------------ 501
hCoV-19/Scotland/CLIMB-CM7YKDZ6/2025|EPI_ISL_19896033|2025-05-27          ------------------------------------------------------------ 501
hCoV-19/Scotland/CLIMB-CM7YG8H4/2025|EPI_ISL_19897748|2025-05-02          ------------------------------------------------------------ 501
hCoV-19/Wales/CLIMB-CM7YJXNZ/2025|EPI_ISL_19900036|2025-05-26             ------------------------------------------------------------ 501
hCoV-19/Germany/NW-RKI-I-1148178/2025|EPI_ISL_19902055|2025-05-21         ------------------------------------------------------------ 501
hCoV-19/Germany/NI-RKI-I-1148183/2025|EPI_ISL_19902060|2025-05-18         ------------------------------------------------------------ 501
hCoV-19/Germany/NW-RKI-I-1148191/2025|EPI_ISL_19902068|2025-05-19         ------------------------------------------------------------ 501
hCoV-19/Spain/MD-HGUGM-66414184/2025|EPI_ISL_19906298|2025-05-25          ------------------------------------------------------------ 501
hCoV-19/Spain/MD-HGUGM-66400167/2025|EPI_ISL_19906320|2025-05-22          ------------------------------------------------------------ 501
hCoV-19/France/IDF-RELAB-IPP04646/2025|EPI_ISL_19907194|2025-05-16        ------------------------------------------------------------ 501
hCoV-19/England/CLIMB-CM7YKIUQ/2025|EPI_ISL_19908573|2025-06-07           ------------------------------------------------------------ 501
hCoV-19/France/GES-RELAB-IPP04823/2025|EPI_ISL_19911257|2025-05-26        ------------------------------------------------------------ 501
hCoV-19/France/IDF-RELAB-IPP04863/2025|EPI_ISL_19911265|2025-05-24        ------------------------------------------------------------ 501
hCoV-19/Denmark/DCGC-692245/2025|EPI_ISL_19879704|2025-04-28              ------------------------------------------------------------ 501

hCoV-19/Ireland/D-SVUH-FBA98650-31/2025|EPI_ISL_19866548|2025-05-04       CTGATGGCTACCCTCTTGAGTGCATTAAAGACCTTCTAGCACGTGCTGGTAAAGCTTCAT 444
hCoV-19/Spain/PV-HUB-5628663/2025|EPI_ISL_19908839|2025-05-16             ------------------------------------------------------------ 501
hCoV-19/Spain/PV-HUB-41587498/2025|EPI_ISL_19908930|2025-05-20            ------------------------------------------------------------ 501
hCoV-19/Spain/PV-HUB-56291851/2025|EPI_ISL_19908970|2025-05-31            ------------------------------------------------------------ 501
hCoV-19/Northern_Ireland/CLIMB-CM7YR8I6/2025|EPI_ISL_19877334|2025-04-23  ------------------------------------------------------------ 501
hCoV-19/Germany/NW-RKI-I-1148136/2025|EPI_ISL_19871950|2025-04-23         ------------------------------------------------------------ 501
hCoV-19/Germany/NW-RKI-I-1148141/2025|EPI_ISL_19871955|2025-04-23         ------------------------------------------------------------ 501
hCoV-19/Germany/BW-RKI-I-1148164/2025|EPI_ISL_19884987|2025-04-17         ------------------------------------------------------------ 501
hCoV-19/Scotland/CLIMB-CM7YFGW7/2025|EPI_ISL_19897691|2025-05-24          ------------------------------------------------------------ 501
hCoV-19/Ireland/G-LUH-N3473/2025|EPI_ISL_19893181|2025-05-14              ------------------------------------------------------------ 501
hCoV-19/Spain/AN-PMC-42728/2025|EPI_ISL_19888347|2025-05-25               ------------------------------------------------------------ 501
hCoV-19/Sweden/000200866492N2/2025|EPI_ISL_19870050|2025-04-22            ------------------------------------------------------------ 501
hCoV-19/Sweden/000200867159N2/2025|EPI_ISL_19870052|2025-04-23            ------------------------------------------------------------ 501
hCoV-19/Ireland/G-LUH-N3445/2025|EPI_ISL_19893154|2025-05-07              ------------------------------------------------------------ 501
hCoV-19/Ireland/G-LUH-N3443/2025|EPI_ISL_19893152|2025-05-07              ------------------------------------------------------------ 501
hCoV-19/Scotland/CLIMB-CM7YF9PJ/2025|EPI_ISL_19895983|2025-05-19          ------------------------------------------------------------ 501
hCoV-19/Scotland/CLIMB-CM7YKMK8/2025|EPI_ISL_19869976|2025-04-25          ------------------------------------------------------------ 501
hCoV-19/Ireland/G-LUH-N3444/2025|EPI_ISL_19893153|2025-05-07              ------------------------------------------------------------ 501
hCoV-19/Switzerland/VD-CHUV-8604552682/2025|EPI_ISL_19906527|2025-05-19   ------------------------------------------------------------ 501
hCoV-19/Scotland/CLIMB-CM7YR3YO/2025|EPI_ISL_19896015|2025-05-24          ------------------------------------------------------------ 501
hCoV-19/Sweden/O-18_SE100_25CS500180/2025|EPI_ISL_19866288|2025-04-15     ------------------------------------------------------------ 501
```

hCoV-19/Ireland/D-CS250088/2025|EPI_ISL_19877457|2025-04-16 -- 501
hCoV-19/Ireland/G-LUH-N3442/2025|EPI_ISL_19893151|2025-05-07 -- 501
hCoV-19/Scotland/CLIMB-CM7YMZ5M/2025|EPI_ISL_19906439|2025-05-22 -- 501
hCoV-19/Scotland/CLIMB-CM7YR3ZG/2025|EPI_ISL_19897722|2025-05-13 -- 501
hCoV-19/Scotland/CLIMB-CM7YEYGT/2025|EPI_ISL_19897741|2025-04-28 -- 501
hCoV-19/Scotland/CLIMB-CM7YGAJ3/2025|EPI_ISL_19895963|2025-05-13 -- 501
hCoV-19/Scotland/CLIMB-CM7YJ1ZO/2025|EPI_ISL_19897757|2025-05-14 -- 501
hCoV-19/Scotland/CLIMB-CM7Y8C4Y/2025|EPI_ISL_19906404|2025-05-15 -- 501
hCoV-19/Northern_Ireland/CLIMB-CM7YGGYM/2025|EPI_ISL_19897641|2025-05-23 -- 501
hCoV-19/Scotland/CLIMB-CM7YFW7M/2025|EPI_ISL_19881718|2025-05-10 -- 501
hCoV-19/Scotland/CLIMB-CM7YEMXP/2025|EPI_ISL_19888387|2025-05-10 -- 501
hCoV-19/Spain/CT-HUVH-E593511/2025|EPI_ISL_19881731|2025-05-06 -- 501
hCoV-19/Spain/CT-HUJT-E325362/2025|EPI_ISL_19889935|2025-05-20 -- 501
hCoV-19/Spain/PV-HUD-84743204/2025|EPI_ISL_19907746|2025-06-06 -- 501
hCoV-19/Ireland/D-SVUH-FBB21492-54/2025|EPI_ISL_19881758|2025-05-11 -- 501
hCoV-19/Spain/CT-HUVH-E596725/2025|EPI_ISL_19881729|2025-05-10 -- 501
hCoV-19/Spain/CT-HUVH-E593882/2025|EPI_ISL_19881732|2025-05-06 -- 501
hCoV-19/Finland/THL-02385/2025|EPI_ISL_19875928|2025-04-16 -- 501
hCoV-19/Ireland/D-BH-02351167/2025|EPI_ISL_19911337|2025-05-28 -- 501
hCoV-19/Ireland/D-NVRL-XIRL00001782/2025|EPI_ISL_19896104|2025-05-14 -- 501
hCoV-19/Ireland/TA-NVRL-GIRL00039444/2025|EPI_ISL_19896118|2025-05-16 -- 501
hCoV-19/Spain/GA-CHUAC-13223/2025|EPI_ISL_19894946|2025-05-31 -- 501
hCoV-19/Spain/GA-CHUAC-13215/2025|EPI_ISL_19894939|2025-05-29 -- 501
hCoV-19/Finland/THL-02398/2025|EPI_ISL_19875939|2025-05-04 -- 501
hCoV-19/Finland/THL-02400/2025|EPI_ISL_19875941|2025-05-03 -- 501
hCoV-19/Ireland/LK-UHL-702929/2025|EPI_ISL_19877895|2025-04-16 -- 501
hCoV-19/Ireland/LK-UHL-703788/2025|EPI_ISL_19877905|2025-05-03 -- 501
hCoV-19/Ireland/LK-UHL-703851/2025|EPI_ISL_19877907|2025-05-04 -- 501
hCoV-19/Ireland/LK-UHL-703889/2025|EPI_ISL_19877908|2025-05-05 -- 501
hCoV-19/Ireland/D-SVUH-FBB21492-49/2025|EPI_ISL_19881756|2025-05-14 -- 501
hCoV-19/Ireland/D-SVUH-FBB21492-59/2025|EPI_ISL_19881763|2025-05-07 -- 501
hCoV-19/Spain/CT-HUGTiP-G3R212/2025|EPI_ISL_19883055|2025-05-20 -- 501
hCoV-19/Ireland/LK-UHL-704398/2025|EPI_ISL_19888621|2025-05-16 -- 501
hCoV-19/Ireland/G-GUH-N3466/2025|EPI_ISL_19893175|2025-05-03 -- 501
hCoV-19/Belgium/Sciensano-LS-S5276/2025|EPI_ISL_19893834|2025-04-22 -- 501
hCoV-19/Italy/VEN-ULSS8-0019244_VI/2025|EPI_ISL_19899362|2025-05-26 -- 501
hCoV-19/Spain/CT-HUVH-E618109/2025|EPI_ISL_19907773|2025-06-10 -- 501
hCoV-19/Ireland/D-BH-03819149/2025|EPI_ISL_19911343|2025-05-29 -- 501
hCoV-19/Ireland/D-BH-03993161/2025|EPI_ISL_19911355|2025-06-10 -- 501
hCoV-19/Spain/CT-HUVH-E587040/2025|EPI_ISL_19871881|2025-04-26 -- 501
hCoV-19/Finland/THL-02392/2025|EPI_ISL_19875934|2025-05-02 -- 501
hCoV-19/Spain/GA-CHUAC-13181/2025|EPI_ISL_19890617|2025-05-20 -- 501
hCoV-19/Spain/CT-HUVH-E614843/2025|EPI_ISL_19907763|2025-06-05 -- 501
hCoV-19/Italy/UMB-LAOP-172-4-02/2025|EPI_ISL_19899474|2025-05-08 -- 501
hCoV-19/Spain/NC-CHN-01008932/2025|EPI_ISL_19900595|2025-04-04 -- 501
hCoV-19/Northern_Ireland/CLIMB-CM7YGDRO/2025|EPI_ISL_19877331|2025-04-14 -- 501
hCoV-19/Scotland/CLIMB-CM7YEE96/2025|EPI_ISL_19897754|2025-05-13 -- 501
hCoV-19/France/ARA-HCL125018289001/2025|EPI_ISL_19880005|2025-05-12 -- 501
hCoV-19/France/ARA-RELAB-HCL725000057501/2025|EPI_ISL_19880014|2025-04-28 -- 501
hCoV-19/France/ARA-RELAB-HCL725000059501/2025|EPI_ISL_19885160|2025-04-25 -- 501
hCoV-19/France/NAQ-RELAB-HCL725000061001/2025|EPI_ISL_19885161|2025-05-10 -- 501
hCoV-19/France/NAQ-RELAB-HCL725000060901/2025|EPI_ISL_19885180|2025-05-09 -- 501
hCoV-19/France/ARA-HCL025075546601/2025|EPI_ISL_19885182|2025-05-09 -- 501

```
hCoV-19/France/ARA-HCL125018461901/2025|EPI_ISL_19885204|2025-05-14            ------------------------------------------------------------ 501
hCoV-19/France/ARA-HCL125018655901/2025|EPI_ISL_19885206|2025-05-14            ------------------------------------------------------------ 501
hCoV-19/France/ARA-HCL125019155501/2025|EPI_ISL_19885209|2025-05-18            ------------------------------------------------------------ 501
hCoV-19/France/ARA-HCL125019240901/2025|EPI_ISL_19885210|2025-05-19            ------------------------------------------------------------ 501
hCoV-19/France/ARA-HCL125019249201/2025|EPI_ISL_19885211|2025-05-19            ------------------------------------------------------------ 501
hCoV-19/France/ARA-HCL125019399701/2025|EPI_ISL_19885212|2025-05-20            ------------------------------------------------------------ 501
hCoV-19/France/ARA-HCL125019408901/2025|EPI_ISL_19885213|2025-05-20            ------------------------------------------------------------ 501
hCoV-19/France/OCC-RELAB-HCL725000064001/2025|EPI_ISL_19895004|2025-05-14      ------------------------------------------------------------ 501
hCoV-19/France/OCC-RELAB-HCL725000064101/2025|EPI_ISL_19895005|2025-05-14      ------------------------------------------------------------ 501
hCoV-19/France/NAQ-RELAB-HCL725000062601/2025|EPI_ISL_19895009|2025-05-16      ------------------------------------------------------------ 501
hCoV-19/France/ARA-RELAB-HCL725000062401/2025|EPI_ISL_19895017|2025-05-16      ------------------------------------------------------------ 501
hCoV-19/France/ARA-RELAB-HCL725000062101/2025|EPI_ISL_19895018|2025-05-13      ------------------------------------------------------------ 501
hCoV-19/France/ARA-HCL125019625101/2025|EPI_ISL_19895034|2025-05-21            ------------------------------------------------------------ 501
hCoV-19/France/ARA-HCL125019754801/2025|EPI_ISL_19895035|2025-05-22            ------------------------------------------------------------ 501
hCoV-19/France/ARA-RELAB-HCL725000064601/2025|EPI_ISL_19895041|2025-05-15      ------------------------------------------------------------ 501
hCoV-19/France/ARA-RELAB-HCL725000066001/2025|EPI_ISL_19895046|2025-05-13      ------------------------------------------------------------ 501
hCoV-19/France/OCC-RELAB-HCL725000068701/2025|EPI_ISL_19905466|2025-05-21      ------------------------------------------------------------ 501
hCoV-19/France/OCC-RELAB-HCL725000069001/2025|EPI_ISL_19905468|2025-05-21      ------------------------------------------------------------ 501
hCoV-19/France/ARA-HCL025089279801/2025|EPI_ISL_19905469|2025-06-02            ------------------------------------------------------------ 501
hCoV-19/France/PAC-RELAB-HCL725000069901/2025|EPI_ISL_19908847|2025-05-26      ------------------------------------------------------------ 501
hCoV-19/France/PAC-RELAB-HCL725000069501/2025|EPI_ISL_19908849|2025-05-30      ------------------------------------------------------------ 501
hCoV-19/France/ARA-RELAB-HCL725000071701/2025|EPI_ISL_19908858|2025-05-22      ------------------------------------------------------------ 501
hCoV-19/France/ARA-HCL025093352901/2025|EPI_ISL_19908867|2025-06-10            ------------------------------------------------------------ 501
hCoV-19/France/ARA-RELAB-HCL725000065801/2025|EPI_ISL_19895045|2025-05-16      ------------------------------------------------------------ 501
hCoV-19/Denmark/DCGC-692234/2025|EPI_ISL_19879694|2025-05-05                   ------------------------------------------------------------ 501
hCoV-19/Finland/THL-02388/2025|EPI_ISL_19875931|2025-04-16                     ------------------------------------------------------------ 501
hCoV-19/Spain/PV-HUD-98024244/2025|EPI_ISL_19907759|2025-06-09                 ------------------------------------------------------------ 501
hCoV-19/Netherlands/GE-RIVM-145386/2025|EPI_ISL_19873764|2025-05-01            ------------------------------------------------------------ 501
hCoV-19/Netherlands/ZH-RIVM-145413/2025|EPI_ISL_19891397|2025-04-30            ------------------------------------------------------------ 501
hCoV-19/Netherlands/NH-RIVM-145458/2025|EPI_ISL_19905062|2025-05-25            ------------------------------------------------------------ 501
hCoV-19/Netherlands/NH-RIVM-145463/2025|EPI_ISL_19905063|2025-05-20            ------------------------------------------------------------ 501
hCoV-19/Netherlands/UT-RIVM-145436/2025|EPI_ISL_19905072|2025-05-15            ------------------------------------------------------------ 501
hCoV-19/Netherlands/UT-RIVM-145470/2025|EPI_ISL_19905077|2025-06-02            ------------------------------------------------------------ 501
hCoV-19/Netherlands/ZH-RIVM-145478/2025|EPI_ISL_19905094|2025-05-30            ------------------------------------------------------------ 501
hCoV-19/Netherlands/ZH-RIVM-145480/2025|EPI_ISL_19905096|2025-05-31            ------------------------------------------------------------ 501
hCoV-19/Netherlands/UT-RIVM-145481/2025|EPI_ISL_19905078|2025-05-31            ------------------------------------------------------------ 501
hCoV-19/Scotland/CLIMB-CM7YRW9H/2025|EPI_ISL_19881711|2025-05-06               ------------------------------------------------------------ 501
hCoV-19/France/IDF-RELAB-IPP04872/2025|EPI_ISL_19911293|2025-05-22             ------------------------------------------------------------ 501
hCoV-19/Canary_Islands/CN-HUC-925112913/2025|EPI_ISL_19900668|2025-04-22       ------------------------------------------------------------ 501
hCoV-19/Scotland/CLIMB-CM7YJ5BB/2025|EPI_ISL_19906458|2025-05-27               ------------------------------------------------------------ 501
hCoV-19/Scotland/CLIMB-CM7Y8PHH/2025|EPI_ISL_19897755|2025-05-14               ------------------------------------------------------------ 501
hCoV-19/Spain/AN-PMC-42706/2025|EPI_ISL_19888325|2025-05-25                    ------------------------------------------------------------ 501
hCoV-19/Scotland/CLIMB-CM7YRIA1/2025|EPI_ISL_19869961|2025-04-20               ------------------------------------------------------------ 501
hCoV-19/Scotland/CLIMB-CM7Y8UWT/2025|EPI_ISL_19869966|2025-04-21               ------------------------------------------------------------ 501
hCoV-19/England/CLIMB-CM7YMFHB/2025|EPI_ISL_19877436|2025-05-10                ------------------------------------------------------------ 501
hCoV-19/Denmark/DCGC-692213/2025|EPI_ISL_19879673|2025-04-28                   ------------------------------------------------------------ 501
hCoV-19/Wales/CLIMB-CM7YRAXQ/2025|EPI_ISL_19883075|2025-05-14                  ------------------------------------------------------------ 501
hCoV-19/Spain/AN-PMC-42696/2025|EPI_ISL_19888316|2025-05-14                    ------------------------------------------------------------ 501
hCoV-19/Spain/AN-PMC-42719/2025|EPI_ISL_19888338|2025-05-15                    ------------------------------------------------------------ 501
hCoV-19/England/CLIMB-CM7YE7HB/2025|EPI_ISL_19892115|2025-05-19                ------------------------------------------------------------ 501
hCoV-19/France/GES-RELAB-IPP04599/2025|EPI_ISL_19893547|2025-05-05             ------------------------------------------------------------ 501
hCoV-19/Scotland/CLIMB-CM7YEPXZ/2025|EPI_ISL_19896028|2025-05-25               ------------------------------------------------------------ 501
hCoV-19/Scotland/CLIMB-CM7YF4QT/2025|EPI_ISL_19896032|2025-05-27               ------------------------------------------------------------ 501
```

```
hCoV-19/Scotland/CLIMB-CM7YKDZ6/2025|EPI_ISL_19896033|2025-05-27          ------------------------------------------------------------ 501
hCoV-19/Scotland/CLIMB-CM7YG8H4/2025|EPI_ISL_19897748|2025-05-02          ------------------------------------------------------------ 501
hCoV-19/Wales/CLIMB-CM7YJXNZ/2025|EPI_ISL_19900036|2025-05-26             ------------------------------------------------------------ 501
hCoV-19/Germany/NW-RKI-I-1148178/2025|EPI_ISL_19902055|2025-05-21         ------------------------------------------------------------ 501
hCoV-19/Germany/NI-RKI-I-1148183/2025|EPI_ISL_19902060|2025-05-18         ------------------------------------------------------------ 501
hCoV-19/Germany/NW-RKI-I-1148191/2025|EPI_ISL_19902068|2025-05-19         ------------------------------------------------------------ 501
hCoV-19/Spain/MD-HGUGM-66414184/2025|EPI_ISL_19906298|2025-05-25          ------------------------------------------------------------ 501
hCoV-19/Spain/MD-HGUGM-66400167/2025|EPI_ISL_19906320|2025-05-22          ------------------------------------------------------------ 501
hCoV-19/France/IDF-RELAB-IPP04646/2025|EPI_ISL_19907194|2025-05-16        ------------------------------------------------------------ 501
hCoV-19/England/CLIMB-CM7YKIUQ/2025|EPI_ISL_19908573|2025-06-07           ------------------------------------------------------------ 501
hCoV-19/France/GES-RELAB-IPP04823/2025|EPI_ISL_19911257|2025-05-26        ------------------------------------------------------------ 501
hCoV-19/France/IDF-RELAB-IPP04863/2025|EPI_ISL_19911265|2025-05-24        ------------------------------------------------------------ 501
hCoV-19/Denmark/DCGC-692245/2025|EPI_ISL_19879704|2025-04-28              ------------------------------------------------------------ 501

hCoV-19/Ireland/D-SVUH-FBA98650-31/2025|EPI_ISL_19866548|2025-05-04       GCACTTTGTCCGAACAACTGGACTTTATTGACACTAAGAGGGGTGTATNCTGCTGCC    501
hCoV-19/Spain/PV-HUB-5628663/2025|EPI_ISL_19908839|2025-05-16             ---------------------------------------------------------    501
hCoV-19/Spain/PV-HUB-41587498/2025|EPI_ISL_19908930|2025-05-20            ---------------------------------------------------------    501
hCoV-19/Spain/PV-HUB-56291851/2025|EPI_ISL_19908970|2025-05-31            ---------------------------------------------------------    501
hCoV-19/Northern_Ireland/CLIMB-CM7YR8I6/2025|EPI_ISL_19877334|2025-04-23  ---------------------------------------------------------    501
hCoV-19/Germany/NW-RKI-I-1148136/2025|EPI_ISL_19871950|2025-04-23         ---------------------------------------------------------    501
hCoV-19/Germany/NW-RKI-I-1148141/2025|EPI_ISL_19871955|2025-04-23         ---------------------------------------------------------    501
hCoV-19/Germany/BW-RKI-I-1148164/2025|EPI_ISL_19884987|2025-04-17         ---------------------------------------------------------    501
hCoV-19/Scotland/CLIMB-CM7YFGW7/2025|EPI_ISL_19897691|2025-05-24          ---------------------------------------------------------    501
hCoV-19/Ireland/G-LUH-N3473/2025|EPI_ISL_19893181|2025-05-14              ---------------------------------------------------------    501
hCoV-19/Spain/AN-PMC-42728/2025|EPI_ISL_19888347|2025-05-25               ---------------------------------------------------------    501
hCoV-19/Sweden/000200866492N2/2025|EPI_ISL_19870050|2025-04-22            ---------------------------------------------------------    501
hCoV-19/Sweden/000200867159N2/2025|EPI_ISL_19870052|2025-04-23            ---------------------------------------------------------    501
hCoV-19/Ireland/G-LUH-N3445/2025|EPI_ISL_19893154|2025-05-07              ---------------------------------------------------------    501
hCoV-19/Ireland/G-LUH-N3443/2025|EPI_ISL_19893152|2025-05-07              ---------------------------------------------------------    501
hCoV-19/Scotland/CLIMB-CM7YF9PJ/2025|EPI_ISL_19895983|2025-05-19          ---------------------------------------------------------    501
hCoV-19/Scotland/CLIMB-CM7YKMK8/2025|EPI_ISL_19869976|2025-04-25          ---------------------------------------------------------    501
hCoV-19/Ireland/G-LUH-N3444/2025|EPI_ISL_19893153|2025-05-07              ---------------------------------------------------------    501
hCoV-19/Switzerland/VD-CHUV-8604552682/2025|EPI_ISL_19906527|2025-05-19   ---------------------------------------------------------    501
hCoV-19/Scotland/CLIMB-CM7YR3YO/2025|EPI_ISL_19896015|2025-05-24          ---------------------------------------------------------    501
hCoV-19/Sweden/O-18_SE100_25CS500180/2025|EPI_ISL_19866288|2025-04-15     ---------------------------------------------------------    501
hCoV-19/Ireland/D-CS250088/2025|EPI_ISL_19877457|2025-04-16               ---------------------------------------------------------    501
hCoV-19/Ireland/G-LUH-N3442/2025|EPI_ISL_19893151|2025-05-07              ---------------------------------------------------------    501
hCoV-19/Scotland/CLIMB-CM7YMZ5M/2025|EPI_ISL_19906439|2025-05-22          ---------------------------------------------------------    501
hCoV-19/Scotland/CLIMB-CM7YR3ZG/2025|EPI_ISL_19897722|2025-05-13          ---------------------------------------------------------    501
hCoV-19/Scotland/CLIMB-CM7YEYGT/2025|EPI_ISL_19897741|2025-04-28          ---------------------------------------------------------    501
hCoV-19/Scotland/CLIMB-CM7YGAJ3/2025|EPI_ISL_19895963|2025-05-13          ---------------------------------------------------------    501
hCoV-19/Scotland/CLIMB-CM7YJ1ZO/2025|EPI_ISL_19897757|2025-05-14          ---------------------------------------------------------    501
hCoV-19/Scotland/CLIMB-CM7Y8C4Y/2025|EPI_ISL_19906404|2025-05-15          ---------------------------------------------------------    501
hCoV-19/Northern_Ireland/CLIMB-CM7YGGYM/2025|EPI_ISL_19897641|2025-05-23  ---------------------------------------------------------    501
hCoV-19/Scotland/CLIMB-CM7YFW7M/2025|EPI_ISL_19881718|2025-05-10          ---------------------------------------------------------    501
hCoV-19/Scotland/CLIMB-CM7YEMXP/2025|EPI_ISL_19888387|2025-05-10          ---------------------------------------------------------    501
hCoV-19/Spain/CT-HUVH-E593511/2025|EPI_ISL_19881731|2025-05-06            ---------------------------------------------------------    501
hCoV-19/Spain/CT-HUJT-E325362/2025|EPI_ISL_19889935|2025-05-20            ---------------------------------------------------------    501
hCoV-19/Spain/PV-HUD-84743204/2025|EPI_ISL_19907746|2025-06-06            ---------------------------------------------------------    501
hCoV-19/Ireland/D-SVUH-FBB21492-54/2025|EPI_ISL_19881758|2025-05-11       ---------------------------------------------------------    501
hCoV-19/Spain/CT-HUVH-E596725/2025|EPI_ISL_19881729|2025-05-10            ---------------------------------------------------------    501
hCoV-19/Spain/CT-HUVH-E593882/2025|EPI_ISL_19881732|2025-05-06            ---------------------------------------------------------    501
```

hCoV-19/Finland/THL-02385/2025|EPI_ISL_19875928|2025-04-16 --- 501
hCoV-19/Ireland/D-BH-02351167/2025|EPI_ISL_19911337|2025-05-28 --- 501
hCoV-19/Ireland/D-NVRL-XIRL00001782/2025|EPI_ISL_19896104|2025-05-14 --- 501
hCoV-19/Ireland/TA-NVRL-GIRL00039444/2025|EPI_ISL_19896118|2025-05-16 --- 501
hCoV-19/Spain/GA-CHUAC-13223/2025|EPI_ISL_19894946|2025-05-31 --- 501
hCoV-19/Spain/GA-CHUAC-13215/2025|EPI_ISL_19894939|2025-05-29 --- 501
hCoV-19/Finland/THL-02398/2025|EPI_ISL_19875939|2025-05-04 --- 501
hCoV-19/Finland/THL-02400/2025|EPI_ISL_19875941|2025-05-03 --- 501
hCoV-19/Ireland/LK-UHL-702929/2025|EPI_ISL_19877895|2025-04-16 --- 501
hCoV-19/Ireland/LK-UHL-703788/2025|EPI_ISL_19877905|2025-05-03 --- 501
hCoV-19/Ireland/LK-UHL-703851/2025|EPI_ISL_19877907|2025-05-04 --- 501
hCoV-19/Ireland/LK-UHL-703889/2025|EPI_ISL_19877908|2025-05-05 --- 501
hCoV-19/Ireland/D-SVUH-FBB21492-49/2025|EPI_ISL_19881756|2025-05-14 --- 501
hCoV-19/Ireland/D-SVUH-FBB21492-59/2025|EPI_ISL_19881763|2025-05-07 --- 501
hCoV-19/Spain/CT-HUGTiP-G3R212/2025|EPI_ISL_19883055|2025-05-20 --- 501
hCoV-19/Ireland/LK-UHL-704398/2025|EPI_ISL_19888621|2025-05-16 --- 501
hCoV-19/Ireland/G-GUH-N3466/2025|EPI_ISL_19893175|2025-05-03 --- 501
hCoV-19/Belgium/Sciensano-LS-S5276/2025|EPI_ISL_19893834|2025-04-22 --- 501
hCoV-19/Italy/VEN-ULSS8-0019244_VI/2025|EPI_ISL_19899362|2025-05-26 --- 501
hCoV-19/Spain/CT-HUVH-E618109/2025|EPI_ISL_19907773|2025-06-10 --- 501
hCoV-19/Ireland/D-BH-03819149/2025|EPI_ISL_19911343|2025-05-29 --- 501
hCoV-19/Ireland/D-BH-03993161/2025|EPI_ISL_19911355|2025-06-10 --- 501
hCoV-19/Spain/CT-HUVH-E587040/2025|EPI_ISL_19871881|2025-04-26 --- 501
hCoV-19/Finland/THL-02392/2025|EPI_ISL_19875934|2025-05-02 --- 501
hCoV-19/Spain/GA-CHUAC-13181/2025|EPI_ISL_19890617|2025-05-20 --- 501
hCoV-19/Spain/CT-HUVH-E614843/2025|EPI_ISL_19907763|2025-06-05 --- 501
hCoV-19/Italy/UMB-LAOP-172-4-02/2025|EPI_ISL_19899474|2025-05-08 --- 501
hCoV-19/Spain/NC-CHN-01008932/2025|EPI_ISL_19900595|2025-04-04 --- 501
hCoV-19/Northern_Ireland/CLIMB-CM7YGDRO/2025|EPI_ISL_19877331|2025-04-14 --- 501
hCoV-19/Scotland/CLIMB-CM7YEE96/2025|EPI_ISL_19897754|2025-05-13 --- 501
hCoV-19/France/ARA-HCL125018289001/2025|EPI_ISL_19880005|2025-05-12 --- 501
hCoV-19/France/ARA-RELAB-HCL725000057501/2025|EPI_ISL_19880014|2025-04-28 --- 501
hCoV-19/France/ARA-RELAB-HCL725000059501/2025|EPI_ISL_19885160|2025-04-25 --- 501
hCoV-19/France/NAQ-RELAB-HCL725000061001/2025|EPI_ISL_19885161|2025-05-10 --- 501
hCoV-19/France/NAQ-RELAB-HCL725000060901/2025|EPI_ISL_19885180|2025-05-09 --- 501
hCoV-19/France/ARA-HCL025075546601/2025|EPI_ISL_19885182|2025-05-09 --- 501
hCoV-19/France/ARA-HCL125018461901/2025|EPI_ISL_19885204|2025-05-14 --- 501
hCoV-19/France/ARA-HCL125018655901/2025|EPI_ISL_19885206|2025-05-14 --- 501
hCoV-19/France/ARA-HCL125019155501/2025|EPI_ISL_19885209|2025-05-18 --- 501
hCoV-19/France/ARA-HCL125019240901/2025|EPI_ISL_19885210|2025-05-19 --- 501
hCoV-19/France/ARA-HCL125019249201/2025|EPI_ISL_19885211|2025-05-19 --- 501
hCoV-19/France/ARA-HCL125019399701/2025|EPI_ISL_19885212|2025-05-20 --- 501
hCoV-19/France/ARA-HCL125019408901/2025|EPI_ISL_19885213|2025-05-20 --- 501
hCoV-19/France/OCC-RELAB-HCL725000064001/2025|EPI_ISL_19895004|2025-05-14 --- 501
hCoV-19/France/OCC-RELAB-HCL725000064101/2025|EPI_ISL_19895005|2025-05-14 --- 501
hCoV-19/France/NAQ-RELAB-HCL725000062601/2025|EPI_ISL_19895009|2025-05-16 --- 501
hCoV-19/France/ARA-RELAB-HCL725000062401/2025|EPI_ISL_19895017|2025-05-16 --- 501
hCoV-19/France/ARA-RELAB-HCL725000062101/2025|EPI_ISL_19895018|2025-05-13 --- 501
hCoV-19/France/ARA-HCL125019625101/2025|EPI_ISL_19895034|2025-05-21 --- 501
hCoV-19/France/ARA-HCL125019754801/2025|EPI_ISL_19895035|2025-05-22 --- 501
hCoV-19/France/ARA-RELAB-HCL725000064601/2025|EPI_ISL_19895041|2025-05-15 --- 501
hCoV-19/France/ARA-RELAB-HCL725000066001/2025|EPI_ISL_19895046|2025-05-13 --- 501
hCoV-19/France/OCC-RELAB-HCL725000068701/2025|EPI_ISL_19905466|2025-05-21 --- 501

```
hCoV-19/France/OCC-RELAB-HCL725000069001/2025|EPI_ISL_19905468|2025-05-21      ----------------------------------------------------------    501
hCoV-19/France/ARA-HCL025089279801/2025|EPI_ISL_19905469|2025-06-02            ----------------------------------------------------------    501
hCoV-19/France/PAC-RELAB-HCL725000069901/2025|EPI_ISL_19908847|2025-05-26      ----------------------------------------------------------    501
hCoV-19/France/PAC-RELAB-HCL725000069501/2025|EPI_ISL_19908849|2025-05-30      ----------------------------------------------------------    501
hCoV-19/France/ARA-RELAB-HCL725000071701/2025|EPI_ISL_19908858|2025-05-22      ----------------------------------------------------------    501
hCoV-19/France/ARA-HCL025093352901/2025|EPI_ISL_19908867|2025-06-10            ----------------------------------------------------------    501
hCoV-19/France/ARA-RELAB-HCL725000065801/2025|EPI_ISL_19895045|2025-05-16      ----------------------------------------------------------    501
hCoV-19/Denmark/DCGC-692234/2025|EPI_ISL_19879694|2025-05-05                   ----------------------------------------------------------    501
hCoV-19/Finland/THL-02388/2025|EPI_ISL_19875931|2025-04-16                     ----------------------------------------------------------    501
hCoV-19/Spain/PV-HUD-98024244/2025|EPI_ISL_19907759|2025-06-09                 ----------------------------------------------------------    501
hCoV-19/Netherlands/GE-RIVM-145386/2025|EPI_ISL_19873764|2025-05-01            ----------------------------------------------------------    501
hCoV-19/Netherlands/ZH-RIVM-145413/2025|EPI_ISL_19891397|2025-04-30            ----------------------------------------------------------    501
hCoV-19/Netherlands/NH-RIVM-145458/2025|EPI_ISL_19905062|2025-05-25            ----------------------------------------------------------    501
hCoV-19/Netherlands/NH-RIVM-145463/2025|EPI_ISL_19905063|2025-05-20            ----------------------------------------------------------    501
hCoV-19/Netherlands/UT-RIVM-145436/2025|EPI_ISL_19905072|2025-05-15            ----------------------------------------------------------    501
hCoV-19/Netherlands/UT-RIVM-145470/2025|EPI_ISL_19905077|2025-06-02            ----------------------------------------------------------    501
hCoV-19/Netherlands/ZH-RIVM-145478/2025|EPI_ISL_19905094|2025-05-30            ----------------------------------------------------------    501
hCoV-19/Netherlands/ZH-RIVM-145480/2025|EPI_ISL_19905096|2025-05-31            ----------------------------------------------------------    501
hCoV-19/Netherlands/UT-RIVM-145481/2025|EPI_ISL_19905078|2025-05-31            ----------------------------------------------------------    501
hCoV-19/Scotland/CLIMB-CM7YRW9H/2025|EPI_ISL_19881711|2025-05-06               ----------------------------------------------------------    501
hCoV-19/France/IDF-RELAB-IPP04872/2025|EPI_ISL_19911293|2025-05-22             ----------------------------------------------------------    501
hCoV-19/Canary_Islands/CN-HUC-925112913/2025|EPI_ISL_19900668|2025-04-22       ----------------------------------------------------------    501
hCoV-19/Scotland/CLIMB-CM7YJ5BB/2025|EPI_ISL_19906458|2025-05-27               ----------------------------------------------------------    501
hCoV-19/Scotland/CLIMB-CM7Y8PHH/2025|EPI_ISL_19897755|2025-05-14               ----------------------------------------------------------    501
hCoV-19/Spain/AN-PMC-42706/2025|EPI_ISL_19888325|2025-05-25                    ----------------------------------------------------------    501
hCoV-19/Scotland/CLIMB-CM7YRIA1/2025|EPI_ISL_19869961|2025-04-20               ----------------------------------------------------------    501
hCoV-19/Scotland/CLIMB-CM7Y8UWT/2025|EPI_ISL_19869966|2025-04-21               ----------------------------------------------------------    501
hCoV-19/England/CLIMB-CM7YMFHB/2025|EPI_ISL_19877436|2025-05-10                ----------------------------------------------------------    501
hCoV-19/Denmark/DCGC-692213/2025|EPI_ISL_19879673|2025-04-28                   ----------------------------------------------------------    501
hCoV-19/Wales/CLIMB-CM7YRAXQ/2025|EPI_ISL_19883075|2025-05-14                  ----------------------------------------------------------    501
hCoV-19/Spain/AN-PMC-42696/2025|EPI_ISL_19888316|2025-05-14                    ----------------------------------------------------------    501
hCoV-19/Spain/AN-PMC-42719/2025|EPI_ISL_19888338|2025-05-15                    ----------------------------------------------------------    501
hCoV-19/England/CLIMB-CM7YE7HB/2025|EPI_ISL_19892115|2025-05-19                ----------------------------------------------------------    501
hCoV-19/France/GES-RELAB-IPP04599/2025|EPI_ISL_19893547|2025-05-05             ----------------------------------------------------------    501
hCoV-19/Scotland/CLIMB-CM7YEPXZ/2025|EPI_ISL_19896028|2025-05-25               ----------------------------------------------------------    501
hCoV-19/Scotland/CLIMB-CM7YF4QT/2025|EPI_ISL_19896032|2025-05-27               ----------------------------------------------------------    501
hCoV-19/Scotland/CLIMB-CM7YKDZ6/2025|EPI_ISL_19896033|2025-05-27               ----------------------------------------------------------    501
hCoV-19/Scotland/CLIMB-CM7YG8H4/2025|EPI_ISL_19897748|2025-05-02               ----------------------------------------------------------    501
hCoV-19/Wales/CLIMB-CM7YJXNZ/2025|EPI_ISL_19900036|2025-05-26                  ----------------------------------------------------------    501
hCoV-19/Germany/NW-RKI-I-1148178/2025|EPI_ISL_19902055|2025-05-21              ----------------------------------------------------------    501
hCoV-19/Germany/NI-RKI-I-1148183/2025|EPI_ISL_19902060|2025-05-18              ----------------------------------------------------------    501
hCoV-19/Germany/NW-RKI-I-1148191/2025|EPI_ISL_19902068|2025-05-19              ----------------------------------------------------------    501
hCoV-19/Spain/MD-HGUGM-66414184/2025|EPI_ISL_19906298|2025-05-25               ----------------------------------------------------------    501
hCoV-19/Spain/MD-HGUGM-66400167/2025|EPI_ISL_19906320|2025-05-22               ----------------------------------------------------------    501
hCoV-19/France/IDF-RELAB-IPP04646/2025|EPI_ISL_19907194|2025-05-16             ----------------------------------------------------------    501
hCoV-19/England/CLIMB-CM7YKIUQ/2025|EPI_ISL_19908573|2025-06-07                ----------------------------------------------------------    501
hCoV-19/France/GES-RELAB-IPP04823/2025|EPI_ISL_19911257|2025-05-26             ----------------------------------------------------------    501
hCoV-19/France/IDF-RELAB-IPP04863/2025|EPI_ISL_19911265|2025-05-24             ----------------------------------------------------------    501
hCoV-19/Denmark/DCGC-692245/2025|EPI_ISL_19879704|2025-04-28                   ----------------------------------------------------------    501
```

Supplementary Figure S2. All of the SARS-CoV-2 genomes considered in this paper. The conserved region is outlined in bold (blue and red), where the leader sequence is colored in red.

```
                                                                                    30                  50                  70                  90                  100
                                                                                    |         |         |         |         |         |         |         |         |
hCoV-19/Ireland/D-SVUH-FBA98650-31/2025|EPI_ISL_19866548|2025-05-04               ---------------------------------------------------------------------------------------------
hCoV-19/Spain/PV-HUB-5628663/2025|EPI_ISL_19908839|2025-05-16                     ---------------------------------------------------------------------------------------------
hCoV-19/Spain/PV-HUB-41587498/2025|EPI_ISL_19908930|2025-05-20                    ---------------------------------------------------------------------------------------------
hCoV-19/Spain/PV-HUB-56291851/2025|EPI_ISL_19908970|2025-05-31                    ---------------------------------------------------------------------------------------------
hCoV-19/Northern_Ireland/CLIMB-CM7YR8I6/2025|EPI_ISL_19877334|2025-04-23          ------------------------------------------------------------------------GCTGCATGCTTAGTGCACTCA
hCoV-19/Germany/NW-RKI-I-1148136/2025|EPI_ISL_19871950|2025-04-23                 -------------------------------------------------AAAATCTGTGTGGCTGTCACTCGGCTGCATGCTTAGTGCACTCA
hCoV-19/Germany/NW-RKI-I-1148141/2025|EPI_ISL_19871955|2025-04-23                 -------------------------------------------------AAAATCTGTGTGGCTGTCACTCGGCTGCATGCTTAGTGCACTCA
hCoV-19/Germany/BW-RKI-I-1148164/2025|EPI_ISL_19884987|2025-04-17                 -------------------------------------------------AAAATCTGTGTGGCTGTCACTCGGCTGCATGCTTAGTGCACTCA
hCoV-19/Scotland/CLIMB-CM7YFGW7/2025|EPI_ISL_19897691|2025-05-24                  -------------------------------------------------AAAATCTGTGTGGCTGTCACTCGGCTGCATGCTTAGTGCACTCA
hCoV-19/Ireland/G-LUH-N3473/2025|EPI_ISL_19893181|2025-05-14                      AACCAACCAACTTTTGATCTCTTGTAGATCTGTTCTCTAAACGAACTTTAAAATCTGTGTGGCTGTCACTCGGCTGCATGCTTAGTGCACTCA
hCoV-19/Spain/AN-PMC-42728/2025|EPI_ISL_19888347|2025-05-25                       AACCAACTAACTTTTGATCTCTTGTAGATCTGTTCTCTAAACGAACTTNAAAATCTGTGTGGCTGTCACTCGGCTGCATGCTTAGTGCACTCA
hCoV-19/Sweden/000200866492N2/2025|EPI_ISL_19870050|2025-04-22                    AACCAACCAACTTTCGATCTCTTGTAGATCTGTTCTCTAAACGAACTTTAAAATCTGTGTGGCTGTCACTCGGCTGCATGCTTAGTGCACTCA
hCoV-19/Sweden/000200867159N2/2025|EPI_ISL_19870052|2025-04-23                    AACCAACCAACTTTCGATCTCTTGTAGATCTGTTCTCTAAACGAACTTTAAAATCTGTGTGGCTGTCACTCGGCTGCATGCTTAGTGCACTCA
hCoV-19/Ireland/G-LUH-N3445/2025|EPI_ISL_19893154|2025-05-07                      AACCAACCAACTTTTGATCTCTTGTAGATCTGTTCTCTAAACGAACTTTAAAATCTGTGTGGCTGTCACTCGGCTGCATGCTTAGTGCACTCA
hCoV-19/Ireland/G-LUH-N3443/2025|EPI_ISL_19893152|2025-05-07                      AACCAACCAACTTTTGATCTCTTGTAGATCTGTTCTCTAAACGAACTTTAAAATCTGTGTGGCTGTCACTCGGCTGCATGCTTAGTGCACTCA
hCoV-19/Scotland/CLIMB-CM7YF9PJ/2025|EPI_ISL_19895983|2025-05-19                  AACCAACCAACTTTTGATCTCTTGTAGATCTGTTCTCTAAACGAACTTNAAAATCTGTGTGGCTGTCACTCGGCTGCATGCTTAGTGCACTCA
hCoV-19/Scotland/CLIMB-CM7YKMK8/2025|EPI_ISL_19869976|2025-04-25                  AACCAACCAACTTTTGATCTCTTGTAGATCTGTTCTCTAAACGAACTTNAAAATCTGTGTGGCTGTCACTCGGCTGCATGCTTAGTGCACTCA
hCoV-19/Ireland/G-LUH-N3444/2025|EPI_ISL_19893153|2025-05-07                      AACCAACCAACTTTTGATCTCTTGTAGATCTGTTCTCTAAACGAACTTTAAAATCTGTGTGGCTGTCACTCGGCTGCATGCTTAGTGCACTCA
hCoV-19/Switzerland/VD-CHUV-8604552682/2025|EPI_ISL_19906527|2025-05-19           AACCAACCAACTTTTGATCTCTTGTAGATCTGTTCTCTAAACGAACTTTAAAATCTGTGTGGCTGTCACTCGGCTGCATGCTTAGTGCACTCA
hCoV-19/Scotland/CLIMB-CM7YR3YO/2025|EPI_ISL_19896015|2025-05-24                  AACCAACCAACTTTTGATCTCTTGTAGATCTGTTCTCTAAACGAACTTNAAAATCTGTGTGGCTGTCACTCGGCTGCATGCTTAGTGCACTCA
hCoV-19/Sweden/O-18_SE100_25CS500180/2025|EPI_ISL_19866288|2025-04-15            AACCAACCAACTTTYGATCTCTTGTAGATCTGTTCTCTAAACGAACTTTAAAATCTGTGTGGCTGTCACTCGGCTGCATGCTTAGTGCACTCA
hCoV-19/Ireland/D-CS250088/2025|EPI_ISL_19877457|2025-04-16                       AACCAACCAACTTTCGATCTCTTGTAGATCTGTTCTCTAAACGAACTTTAAAATCTGTGTGGCTGTCACTCGGCTGCATGCTTAGTGCACTCA
hCoV-19/Ireland/G-LUH-N3442/2025|EPI_ISL_19893151|2025-05-07                      AACCAACCAACTTTTGATCTCTTGTAGATCTGTTCTCTAAACGAACTTTAAAATCTGTGTGGCTGTCACTCGGCTGCATGCTTAGTGCACTCA
hCoV-19/Scotland/CLIMB-CM7YMZ5M/2025|EPI_ISL_19906439|2025-05-22                  AACCAACCAACTTTTGATCTCTTGTAGATCTGTTCTCTAAACGAACNNNAAAATCTGTGTGGCTGTCACTCGGCTGCATGCTTAGTGCACTCA
hCoV-19/Scotland/CLIMB-CM7YR3ZG/2025|EPI_ISL_19897722|2025-05-13                  AACCAACTAACTTTTGATCTCTTGTAGATCTGTTCTCTAAACGAACNNNAAAATCTGTGTGGCTGTCACTCGGCTGCATGCTTAGTGCACTCA
hCoV-19/Scotland/CLIMB-CM7YEYGT/2025|EPI_ISL_19897741|2025-04-28                  AACCAACCAACTTTTGATCTCTTGTAGATCTGTTCTCTAAACGAACNNNAAAATCTGTGTGGCTGTCACTCGGCTGCATGCTTAGTGCACTCA
hCoV-19/Scotland/CLIMB-CM7YGAJ3/2025|EPI_ISL_19895963|2025-05-13                  AACCAACCAACTTTTGATCTCTTGTAGATCTGTTCTCTAAACGAACNNNAAAATCTGTGTGGCTGTCACTCGGCTGCATGCTTAGTGCACTCA
hCoV-19/Scotland/CLIMB-CM7YJ1ZO/2025|EPI_ISL_19897757|2025-05-14                  AACCAACCAACTTTTGATCTCTTGTAGATCTGTTCTCTAAACGAACNNNAAAATCTGTGTGGCTGTCACTCGGCTGCATGCTTAGTGCACTCA
hCoV-19/Scotland/CLIMB-CM7Y8C4Y/2025|EPI_ISL_19906404|2025-05-15                  AACCAACCAACTTTTGATCTCTTGTAGATCTGTTCTCTAAACGAACNNNAAAATCTGTGTGGCTGTCACTCGGCTGCATGCTTAGTGCACTCA
hCoV-19/Northern_Ireland/CLIMB-CM7YGGYM/2025|EPI_ISL_19897641|2025-05-23          AACCAACCAACTTTTGATCTCTTGTAGATCTGTTCTCTAAACGAACNNNAAAATCTGTGTGGCTGTCACTCGGCTGCATGCTTAGTGCACTCA
hCoV-19/Scotland/CLIMB-CM7YFW7M/2025|EPI_ISL_19881718|2025-05-10                  AACCAACCAACTTTTGATCTCTTGTAGATCTGTTCTCTAAACGAACNNNAAAATCTGTGTGGCTGTCACTCGGCTGCATGCTTAGTGCACTCA
hCoV-19/Scotland/CLIMB-CM7YEMXP/2025|EPI_ISL_19888387|2025-05-10                  AACCAACCAACTTTTGATCTCTTGTAGATCTGTTCTCTAAACGAACNNNAAAATCTGTGTGGCTGTCACTCGGCTGCATGCTTAGTGCACTCA
hCoV-19/Spain/CT-HUVH-E593511/2025|EPI_ISL_19881731|2025-05-06                    ----------------------------TCTGTTCTCTAAACGAACTTTAAAATCTGTGTGGCTGTCACTCGGCTGCATGCTTAGTGCACTCA
hCoV-19/Spain/CT-HUJT-E325362/2025|EPI_ISL_19889935|2025-05-20                    ---------------------------ATCTGTTCTCTAAACGAACTTTAAAATCTGTGTGGCTGTCACTCGGCTGCATGCTTAGTGCACTCA
hCoV-19/Spain/PV-HUD-84743204/2025|EPI_ISL_19907746|2025-06-06                    --------------------------GATCTGTTCTCTAAACGAACTTTAAAATCTGTGTGGCTGTCACTCGGCTGCATGCTTAGTGCACTCA
hCoV-19/Ireland/D-SVUH-FBB21492-54/2025|EPI_ISL_19881758|2025-05-11               -------------------------AGATCTGTTCTCTAAACGAACTTTAAAATCTGTGTGGCTGTCACTCGGCTGCATGCTTAGTGCACTCA
hCoV-19/Spain/CT-HUVH-E596725/2025|EPI_ISL_19881729|2025-05-10                    -------------------------------GTTCTCTAAACGAACTTTAAAATCTGTGTGGCTGTCACTCGGCTGCATGCTTAGTGCACTCA
hCoV-19/Spain/CT-HUVH-E593882/2025|EPI_ISL_19881732|2025-05-06                    -------------------------------GTTCTCTAAACGAACTTTAAAATCTGTGTGGCTGTCACTCGGCTGCATGCTTAGTGCACTCA
hCoV-19/Finland/THL-02385/2025|EPI_ISL_19875928|2025-04-16                        ------------------------TAGATCTGTTCTCTAAACGAACTTTAAAATCTGTGTGGCTGTCACTCGGCTGCATGCTTAGTGCACTCA
hCoV-19/Ireland/D-BH-02351167/2025|EPI_ISL_19911337|2025-05-28                    -------------------------AGATCTGTTCTCTAAACGAACTTTAAAATCTGTGTGGCTGTCACTTGGCTGCATGCTTAGTGCACTCA
hCoV-19/Ireland/D-NVRL-XIRL00001782/2025|EPI_ISL_19896104|2025-05-14              -------------------------AGATCTGTTCTTTAAACGAACTTTAAAATCTGTGTGGCTGTCACTCGGCTGCATGCTTAGTGCACTCA
hCoV-19/Ireland/TA-NVRL-GIRL00039444/2025|EPI_ISL_19896118|2025-05-16             -------------------------AGATCTGTTCTTTAAACGAACTTTAAAATCTGTGTGGCTGTCACTCGGCTGCATGCTTAGTGCACTCA
hCoV-19/Spain/GA-CHUAC-13223/2025|EPI_ISL_19894946|2025-05-31                     --------------------------GATCTGTTCTCTAAACGAACTTTAAAATCTGTGTGGCTGTCACTCGGCTGCATGCTTAGTGCACTCA
hCoV-19/Spain/GA-CHUAC-13215/2025|EPI_ISL_19894939|2025-05-29                     --------------------------GATCTGTTCTCTAAACGAACTTTAAAATCTGTGTGGCTGTCACTCGGCTGTATGCTTAGTGCACTCA
hCoV-19/Finland/THL-02398/2025|EPI_ISL_19875939|2025-05-04                        -------------------------AGATCTGTTCTCTAAACGAACTTTAAAATCTGTGTGGCTGTCACTCGGCTGCATGCTTAGTGCACTCA
hCoV-19/Finland/THL-02400/2025|EPI_ISL_19875941|2025-05-03                        -------------------------AGATCTGTTCTCTAAACGAACTTTAAAATCTGTGTGGCTGTCACTCGGCTGCATGCTTAGTGCACTCA
hCoV-19/Ireland/LK-UHL-702929/2025|EPI_ISL_19877895|2025-04-16                    -------------------------AGATCTGTTCTCTAAACGAACTTTAAAATCTGTGTGGCTGTCACTCGGCTGCATGCTTAGTGCACTCA
hCoV-19/Ireland/LK-UHL-703788/2025|EPI_ISL_19877905|2025-05-03                    -------------------------AGATCTGTTCTCTAAACGAACTTTAAAATCTGTGTGGCTGTCACTCGGCTGCATGCTTAGTGCACTCA
hCoV-19/Ireland/LK-UHL-703851/2025|EPI_ISL_19877907|2025-05-04                    -------------------------AGATCTGTTCTCTAAACGAACTTTAAAATCTGTGTGGCTGTCACTCGGCTGCATGCTTAGTGCACTCA
hCoV-19/Ireland/LK-UHL-703889/2025|EPI_ISL_19877908|2025-05-05                    -------------------------AGATCTGTTCTCTAAACGAACTTTAAAATCTGTGTGGCTGTCACTCGGCTGCATGCTTAGTGCACTCA
hCoV-19/Ireland/D-SVUH-FBB21492-49/2025|EPI_ISL_19881756|2025-05-14               -------------------------AGATCTGTTCTCTAAACGAACTTTAAAATCTGTGTGGCTGTCACTCGGCTGCATGCTTAGTGCACTCA
hCoV-19/Ireland/D-SVUH-FBB21492-59/2025|EPI_ISL_19881763|2025-05-07               -------------------------AGATCTGTTCTCTAAACGAACTTTAAAATCTGTGTGGCTGTCACTCGGCTGCATGCTTAGTGCACTCA
hCoV-19/Spain/CT-HUGTiP-G3R212/2025|EPI_ISL_19883055|2025-05-20                   -------------------------AGATCTGTTCTCTAAACGAACTTTAAAATCTGTGTGGCTGTCACTCGGCTGCATGCTTAGTGCACTCA
hCoV-19/Ireland/LK-UHL-704398/2025|EPI_ISL_19888621|2025-05-16                    -------------------------AGATCTGTTCTCTAAACGAACTTTAAAATCTGTGTGGCTGTCACTCGGCTGCATGCTTAGTGCACTCA
hCoV-19/Ireland/G-GUH-N3466/2025|EPI_ISL_19893175|2025-05-03                      -------------------------AGATCTGTTCTCTAAACGAACTTTAAAATCTGTGTGGCTGTCACTCGGCTGCATGCTTAGTGCACTCA
hCoV-19/Belgium/Sciensano-LS-S5276/2025|EPI_ISL_19893834|2025-04-22               -------------------------AGATCTGTTCTCTAAACGAACTTTAAAATCTGTGTGGCTGTCACTCGGCTGCATGCTTAGTGCACTCA
hCoV-19/Italy/VEN-ULSS8-0019244_VI/2025|EPI_ISL_19899362|2025-05-26               -------------------------AGATCTGTTCTCTAAACGAACTTTAAAATCTGTGTGGCTGTCACTCGGCTGCATGCTTAGTGCACTCA
hCoV-19/Spain/CT-HUVH-E618109/2025|EPI_ISL_19907773|2025-06-10                    -------------------------AGATCTGTTCTCTAAACGAACTTTAAAATCTGTGTGGCTGTCACTCGGCTGCATGCTTAGTGCACTCA
hCoV-19/Ireland/D-BH-03819149/2025|EPI_ISL_19911343|2025-05-29                    -------------------------AGATCTGTTCTCTAAACGAACTTTAAAATCTGTGTGGCTGTCACTCGGCTGCATGCTTAGTGCACTCA
hCoV-19/Ireland/D-BH-03993161/2025|EPI_ISL_19911355|2025-06-10                    -------------------------AGATCTGTTCTCTAAACGAACTTTAAAATCTGTGTGGCTGTCACTCGGCTGCATGCTTAGTGCACTCA
hCoV-19/Spain/CT-HUVH-E587040/2025|EPI_ISL_19871881|2025-04-26                    --------------------------GATCTGTTCTCTAAACGAACTTTAAAATCTGTGTGGCTGTCACTCGGCTGCATGCTTAGTGCACTCA
hCoV-19/Finland/THL-02392/2025|EPI_ISL_19875934|2025-05-02                        --------------------------GATCTGTTCTCTAAACGAACTTTAAAATCTGTGTGGCTGTCACTCGGCTGCATGCTTAGTGCACTCA
hCoV-19/Spain/GA-CHUAC-13181/2025|EPI_ISL_19890617|2025-05-20                     --------------------------GATCTGTTCTCTAAACGAACTTTAAAATCTGTGTGGCTGTCACTCGGCTGCATGCTTAGTGCACTCA
```

```
hCoV-19/Spain/CT-HUVH-E614843/2025|EPI_ISL_19907763|2025-06-05    --------------------------GATCTGTTCTCTAAACGAACTTTAAAATCTGTGTGGCTGTCACTCGGCTGCATGCTTAGTGCACTCA
hCoV-19/Italy/UMB-LAOP-172-4-02/2025|EPI_ISL_19899474|2025-05-08    ----AACCAACTTTCGATCTCTTGTAGATCTGTTCTCTAAACGAACTTTAAAATCTGTGTGGCTGTCACTCGGCTGCATGCTTAGTGCACTCA
hCoV-19/Spain/NC-CHN-01008932/2025|EPI_ISL_19900595|2025-04-04    --CCAACCAACTTTCGATCTCTTGTAGATCTGTTCTCTAAACGAACTTTAAAATCTGTGTGGCTGTCACTCGGCTGCATGCTTAGTGCACTCA
hCoV-19/Northern_Ireland/CLIMB-CM7YGDRO/2025|EPI_ISL_19877331|2025-04-14    --------------YGATCTCTTGTAGATCTGTTCTCTAAACGAACTNNAAAATCTGTGTGGCTGTCACTCGGCTGCATGCTTAGTGCACTCA
hCoV-19/Scotland/CLIMB-CM7YEE96/2025|EPI_ISL_19897754|2025-05-13    ---------------GATCTCTTGTAGATCTGTTCTCTAAACGAACTTTAAAATCTGTGTGGCTGTCACTCGGCTGCATGCTTAGTGCACTCA
hCoV-19/France/ARA-HCL125018289001/2025|EPI_ISL_19880005|2025-05-12    ---------------------TTGTAGATCTGTTCTCTAAACGAACNTNAAAATCTGTGTGGCTGTCACTCGGCTGCATGCTTAGTGCACTCA
hCoV-19/France/ARA-RELAB-HCL725000057501/2025|EPI_ISL_19880014|2025-04-28    ---------------------TTGTAGATCTGTTCTCTAAACGAACNTNAAAATCTGTGTGGCTGTCACTCGGCTGCATGCTTAGTGCACTCA
hCoV-19/France/ARA-RELAB-HCL725000059501/2025|EPI_ISL_19885160|2025-04-25    ---------------------TTGTAGATCTGTTCTCTAAACGAACNTNAAAATCTGTGTGGCTGTCACTCGGCTGCATGCTTAGTGCACTCA
hCoV-19/France/NAQ-RELAB-HCL725000061001/2025|EPI_ISL_19885161|2025-05-10    ---------------------TTGTAGATCTGTTCTCTAAACGAACNTNAAAATCTGTGTGGCTGTCACTCGGCTGCATGCTTAGTGCACTCA
hCoV-19/France/NAQ-RELAB-HCL725000060901/2025|EPI_ISL_19885180|2025-05-09    ---------------------TTGTAGATCTGTTCTCTAAACGAACNTNAAAATCTGTGTGGCTGTCACTCGGCTGCATGCTTAGTGCACTCA
hCoV-19/France/ARA-HCL025075546601/2025|EPI_ISL_19885182|2025-05-09    ---------------------TTGTAGATCTGTTCTCTAAACGAACNTNAAAATCTGTGTGGCTGTCACTCGGCTGCATGCTTAGTGCACTCA
hCoV-19/France/ARA-HCL125018461901/2025|EPI_ISL_19885204|2025-05-14    ---------------------TTGTAGATCTGTTCTCTAAACGAACNTNAAAATCTGTGTGGCTGTCACTCGGCTGCATGCTTAGTGCACTCA
hCoV-19/France/ARA-HCL125018655901/2025|EPI_ISL_19885206|2025-05-14    ---------------------TTGTAGATCTGTTCTCTAAACGAACNTNAAAATCTGTGTGGCTGTCACTCGGCTGCATGCTTAGTGCACTCA
hCoV-19/France/ARA-HCL125019155501/2025|EPI_ISL_19885209|2025-05-18    ---------------------TTGTAGATCTGTTCTCTAAACGAACNTNAAAATCTGTGTGGCTGTCACTCGGCTGCATGCTTAGTGCACTCA
hCoV-19/France/ARA-HCL125019240901/2025|EPI_ISL_19885210|2025-05-19    ---------------------TTGTAGATCTGTTCTCTAAACGAACNTNAAAATCTGTGTGGCTGTCACTCGGCTGCATGCTTAGTGCACTCA
hCoV-19/France/ARA-HCL125019249201/2025|EPI_ISL_19885211|2025-05-19    ---------------------TTGTAGATCTGTTCTCTAAACGAACNTNAAAATCTGTGTGGCTGTCACTCGGCTGCATGCTTAGTGCACTCA
hCoV-19/France/ARA-HCL125019399701/2025|EPI_ISL_19885212|2025-05-20    ---------------------TTGTAGATCTGTTCTCTAAACGAACNTNAAAATCTGTGTGGCTGTCACTCGGCTGCATGCTTAGTGCACTCA
hCoV-19/France/ARA-HCL125019408901/2025|EPI_ISL_19885213|2025-05-20    ---------------------TTGTAGATCTGTTCTCTAAACGAACNTNAAAATCTGTGTGGCTGTCACTCGGCTGCATGCTTAGTGCACTCA
hCoV-19/France/OCC-RELAB-HCL725000064001/2025|EPI_ISL_19895004|2025-05-14    ---------------------TTGTAGATCTGTTCTCTAAACGAACNTNAAAATCTGTGTGGCTGTCACTCGGCTGCATGCTTAGTGCACTCA
hCoV-19/France/OCC-RELAB-HCL725000064101/2025|EPI_ISL_19895005|2025-05-14    ---------------------TTGTAGATCTGTTCTCTAAACGAACNTNAAAATCTGTGTGGCTGTCACTCGGCTGCATGCTTAGTGCACTCA
hCoV-19/France/NAQ-RELAB-HCL725000062601/2025|EPI_ISL_19895009|2025-05-16    ---------------------TTGTAGATCTGTTCTCTAAACGAACNTNAAAATCTGTGTGGCTGTCACTCGGCTGCATGCTTAGTGCACTCA
hCoV-19/France/ARA-RELAB-HCL725000062401/2025|EPI_ISL_19895017|2025-05-16    ---------------------TTGTAGATCTGTTCTCTAAACGAACNTNAAAATCTGTGTGGCTGTCACTCGGCTGCATGCTTAGTGCACTCA
hCoV-19/France/ARA-RELAB-HCL725000062101/2025|EPI_ISL_19895018|2025-05-13    ---------------------TTGTAGATCTGTTCTCTAAACGAACNTNAAAATCTGTGTGGCTGTCACTCGGCTGCATGCTTAGTGCACTCA
hCoV-19/France/ARA-HCL125019625101/2025|EPI_ISL_19895034|2025-05-21    ---------------------TTGTAGATCTGTTCTCTAAACGAACNTNAAAATCTGTGTGGCTGTCACTCGGCTGCATGCTTAGTGCACTCA
hCoV-19/France/ARA-HCL125019754801/2025|EPI_ISL_19895035|2025-05-22    ---------------------TTGTAGATCTGTTCTCTAAACGAACNTNAAAATCTGTGTGGCTGTCACTCGGCTGCATGCTTAGTGCACTCA
hCoV-19/France/ARA-RELAB-HCL725000064601/2025|EPI_ISL_19895041|2025-05-15    ---------------------TTGTAGATCTGTTCTCTAAACGAACNTNAAAATCTGTGTGGCTGTCACTCGGCTGCATGCTTAGTGCACTCA
hCoV-19/France/ARA-RELAB-HCL725000066001/2025|EPI_ISL_19895046|2025-05-13    ---------------------TTGTAGATCTGTTCTCTAAACGAACNTNAAAATCTGTGTGGCTGTCACTCGGCTGCATGCTTAGTGCACTCA
hCoV-19/France/OCC-RELAB-HCL725000068701/2025|EPI_ISL_19905466|2025-05-21    ---------------------TTGTAGATCTGTTCTCTAAACGAACNTNAAAATCTGTGTGGCTGTCACTCGGCTGCATGCTTAGTGCACTCA
hCoV-19/France/OCC-RELAB-HCL725000069001/2025|EPI_ISL_19905468|2025-05-21    ---------------------TTGTAGATCTGTTCTCTAAACGAACNTNAAAATCTGTGTGGCTGTCACTCGGCTGCATGCTTAGTGCACTCA
hCoV-19/France/ARA-HCL025089279801/2025|EPI_ISL_19905469|2025-06-02    ---------------------TTGTAGATCTGTTCTCTAAACGAACNTNAAAATCTGTGTGGCTGTCACTCGGCTGCATGCTTAGTGCACTCA
hCoV-19/France/PAC-RELAB-HCL725000069901/2025|EPI_ISL_19908847|2025-05-26    ---------------------TTGTAGATCTGTTCTCTAAACGAACNTNAAAATCTGTGTGGCTGTCACTCGGCTGCATGCTTAGTGCACTCA
hCoV-19/France/PAC-RELAB-HCL725000069501/2025|EPI_ISL_19908849|2025-05-30    ---------------------TTGTAGATCTGTTCTCTAAACGAACNTNAAAATCTGTGTGGCTGTCACTCGGCTGCATGCTTAGTGCACTCA
hCoV-19/France/ARA-RELAB-HCL725000071701/2025|EPI_ISL_19908858|2025-05-22    ---------------------TTGTAGATCTGTTCTCTAAACGAACNTNAAAATCTGTGTGGCTGTCACTCGGCTGCATGCTTAGTGCACTCA
hCoV-19/France/ARA-HCL025093352901/2025|EPI_ISL_19908867|2025-06-10    ---------------------TTGTAGATCTGTTCTCTAAACGAACNTNAAAATCTGTGTGGCTGTCACTCGGCTGCATGCTTAGTGCACTCA
hCoV-19/France/ARA-RELAB-HCL725000065801/2025|EPI_ISL_19895045|2025-05-16    ---------------------TTGTAGATCTGTTCTCTAAACGAACNTNAAAATCTGTGTGGCTGTCACTCGGCTGCATGCTTAGTGCACTCA
hCoV-19/Denmark/DCGC-692234/2025|EPI_ISL_19879694|2025-05-05    -------------------TCTTGTAGATCTGTTCTCTAAACGAACTTTAAAATCTGTGTGGCTGTCACTCGGCTGCATGCTTAGTGCACTCA
hCoV-19/Finland/THL-02388/2025|EPI_ISL_19875931|2025-04-16    ------------------CTCTTGTAGATCTGTTCTCTAAACGAACTTTAAAATCTGTGTGGCTGTCACTCGGCTGCATGCTTAGTGCACTCA
hCoV-19/Spain/PV-HUD-98024244/2025|EPI_ISL_19907759|2025-06-09    ------------------CTCTTGTAGATCTGTTCTCTAAACGAACTTTAAAATCTGTGTGGCTGTCACTCGGCTGCATGCTTAGTGCACTCA
hCoV-19/Netherlands/GE-RIVM-145386/2025|EPI_ISL_19873764|2025-05-01    ---------------------TTGTAGATCTGTTCTCTAAACGAACTTTAAAATCTGTGTGGCTGTCACTCGGCTGCATGCTTAGTGCACTCA
hCoV-19/Netherlands/ZH-RIVM-145413/2025|EPI_ISL_19891397|2025-04-30    ---------------------TTGTAGATCTGTTCTCTAAACGAACTTTAAAATCTGTGTGGCTGTCACTCGGCTGCATGCTTAGTGCACTCA
hCoV-19/Netherlands/NH-RIVM-145458/2025|EPI_ISL_19905062|2025-05-25    ---------------------TTGTAGATCTGTTCTCTAAACGAACTTTAAAATCTGTGTGGCTGTCACTCGGCTGCATGCTTAGTGCACTCA
hCoV-19/Netherlands/NH-RIVM-145463/2025|EPI_ISL_19905063|2025-05-20    ---------------------TTGTAGATCTGTTCTCTAAACGAACTTTAAAATCTGTGTGGCTGTCACTCGGCTGCATGCTTAGTGCACTCA
hCoV-19/Netherlands/UT-RIVM-145436/2025|EPI_ISL_19905072|2025-05-15    ---------------------TTGTAGATCTGTTCTCTAAACGAACTTTAAAATCTGTGTGGCTGTCACTCGGCTGCATGCTTAGTGCACTCA
hCoV-19/Netherlands/UT-RIVM-145470/2025|EPI_ISL_19905077|2025-06-02    ---------------------TTGTAGATCTGTTCTCTAAACGAACTTTAAAATCTGTGTGGCTGTCACTCGGCTGCATGCTTAGTGCACTCA
hCoV-19/Netherlands/ZH-RIVM-145478/2025|EPI_ISL_19905094|2025-05-30    ---------------------TTGTAGATCTGTTCTCTAAACGAACTTTAAAATCTGTGTGGCTGTCACTCGGCTGCATGCTTAGTGCACTCA
hCoV-19/Netherlands/ZH-RIVM-145480/2025|EPI_ISL_19905096|2025-05-31    ---------------------TTGTAGATCTGTTCTCTAAACGAACTTTAAAATCTGTGTGGCTGTCACTCGGCTGCATGCTTAGTGCACTCA
hCoV-19/Netherlands/UT-RIVM-145481/2025|EPI_ISL_19905078|2025-05-31    ---------------------TTGTAGATCTGTTCTCTAAACGAACTTTAAAATCTGTGTGGCTGTCACTTGGCTGCATGCTTAGTGCACTCA
hCoV-19/Scotland/CLIMB-CM7YRW9H/2025|EPI_ISL_19881711|2025-05-06    -------------------------------------------------AAAATCTGTGTGGCTGTCACTCGGCTGCATGCTTAGTGCACTCA
hCoV-19/France/IDF-RELAB-IPP04872/2025|EPI_ISL_19911293|2025-05-22    -----------------------------------------------------TCTGTGTGGCTGTCACTCGGCTGCATGCTTAGTGCACTCA
hCoV-19/Canary_Islands/CN-HUC-925112913/2025|EPI_ISL_19900668|2025-04-22    ---------------------------------------------CTTTAAAATCTGTGTGGCTGTCACTCGGCTGCATGCTTAGTGCACTCA
hCoV-19/Scotland/CLIMB-CM7YJ5BB/2025|EPI_ISL_19906458|2025-05-27    -------------------------------------------------AAAATCTGTGTGACTGTCACTCGGCTGCATGCTTAGTGCACTCA
hCoV-19/Scotland/CLIMB-CM7Y8PHH/2025|EPI_ISL_19897755|2025-05-14    -------------------------------------------------AAAATCTGTGTGGCTGTCACTCGGCTGCATGCTTAGTGCACTCA
hCoV-19/Spain/AN-PMC-42706/2025|EPI_ISL_19888325|2025-05-25    -------------------------------------------------AAAATCTGTGTGGCTGTCACTCGGCTGCATGCTTAGTGCACTCA
hCoV-19/Scotland/CLIMB-CM7YRIA1/2025|EPI_ISL_19869961|2025-04-20    -------------------------------------------------AAAATCTGTGTGGCTGTCACTCGGCTGCATGCTTAGTGCACTCA
hCoV-19/Scotland/CLIMB-CM7Y8UWT/2025|EPI_ISL_19869966|2025-04-21    -------------------------------------------------AAAATCTGTGTGGCTGTCACTCGGCTGCATGCTTAGTGCACTCA
hCoV-19/England/CLIMB-CM7YMFHB/2025|EPI_ISL_19877436|2025-05-10    -------------------------------------------------AAAATCTGTGTGGCTGTCACTCGGCTGCATGCTTAGTGCACTCA
hCoV-19/Denmark/DCGC-692213/2025|EPI_ISL_19879673|2025-04-28    -------------------------------------------------AAAATCTGTGTGGCTGTCACTCGGCTGCATGCTTAGTGCACTCA
hCoV-19/Wales/CLIMB-CM7YRAXQ/2025|EPI_ISL_19883075|2025-05-14    -------------------------------------------------AAAATCTGTGTGGCTGTCACTCGGCTGCATGCTTAGTGCACTCA
hCoV-19/Spain/AN-PMC-42696/2025|EPI_ISL_19888316|2025-05-14    -------------------------------------------------AAAATCTGTGTGGCTGTCACTCGGCTGCATGCTTAGTGCACTCA
hCoV-19/Spain/AN-PMC-42719/2025|EPI_ISL_19888338|2025-05-15    -------------------------------------------------AAAATCTGTGTGGCTGTCACTCGGCTGCATGCTTAGTGCACTCA
hCoV-19/England/CLIMB-CM7YE7HB/2025|EPI_ISL_19892115|2025-05-19    -------------------------------------------------AAAATCTGTGTGGCTGTCACTCGGCTGCATGCTTAGTGCACTCA
hCoV-19/France/GES-RELAB-IPP04599/2025|EPI_ISL_19893547|2025-05-05    -------------------------------------------------AAAATCTGTGTGGCTGTCACTCGGCTGCATGCTTAGTGCACTCA
hCoV-19/Scotland/CLIMB-CM7YEPXZ/2025|EPI_ISL_19896028|2025-05-25    -------------------------------------------------AAAATCTGTGTGGCTGTCACTCGGCTGCATGCTTAGTGCACTCA
hCoV-19/Scotland/CLIMB-CM7YF4QT/2025|EPI_ISL_19896032|2025-05-27    -------------------------------------------------AAAATCTGTGTGGCTGTCACTCGGCTGCATGCTTAGTGCACTCA
hCoV-19/Scotland/CLIMB-CM7YKDZ6/2025|EPI_ISL_19896033|2025-05-27    -------------------------------------------------AAAATCTGTGTGGCTGTCACTCGGCTGCATGCTTAGTGCACTCA
hCoV-19/Scotland/CLIMB-CM7YG8H4/2025|EPI_ISL_19897748|2025-05-02    -------------------------------------------------AAAATCTGTGTGGCTGTCACTCGGCTGCATGCTTAGTGCACTCA
hCoV-19/Wales/CLIMB-CM7YJXNZ/2025|EPI_ISL_19900036|2025-05-26    -------------------------------------------------AAAATCTGTGTGGCTGTCACTCGGCTGCATGCTTAGTGCACTCA
hCoV-19/Germany/NW-RKI-I-1148178/2025|EPI_ISL_19902055|2025-05-21    -------------------------------------------------AAAATCTGTGTGGCTGTCACTCGGCTGCATGCTTAGTGCACTCA
hCoV-19/Germany/NI-RKI-I-1148183/2025|EPI_ISL_19902060|2025-05-18    -------------------------------------------------AAAATCTGTGTGGCTGTCACTCGGCTGCATGCTTAGTGCACTCA
hCoV-19/Germany/NW-RKI-I-1148191/2025|EPI_ISL_19902068|2025-05-19    -------------------------------------------------AAAATCTGTGTGGCTGTCACTCGGCTGCATGCTTAGTGCACTCA
```

```
hCoV-19/Spain/MD-HGUGM-66414184/2025|EPI_ISL_19906298|2025-05-25          -------------------------------------------------AAAATCTGTGTGGCTGTCACTCGGCTGCATGCTTAGTGCACTCA
hCoV-19/Spain/MD-HGUGM-66400167/2025|EPI_ISL_19906320|2025-05-22          -------------------------------------------------AAAATCTGTGTGGCTGTCACTCGGCTGCATGCTTAGTGCACTCA
hCoV-19/France/IDF-RELAB-IPP04646/2025|EPI_ISL_19907194|2025-05-16        -------------------------------------------------AAAATCTGTGTGGCTGTCACTCGGCTGCATGCTTAGTGCACTCA
hCoV-19/England/CLIMB-CM7YKIUQ/2025|EPI_ISL_19908573|2025-06-07           -------------------------------------------------AAAATCTGTGTGGCTGTCACTCGGCTGCATGCTTAGTGCACTCA
hCoV-19/France/GES-RELAB-IPP04823/2025|EPI_ISL_19911257|2025-05-26        -------------------------------------------------AAAATCTGTGTGGCTGTCACTCGGCTGCATGCTTAGTGCACTCA
hCoV-19/France/IDF-RELAB-IPP04863/2025|EPI_ISL_19911265|2025-05-24        -------------------------------------------------AAAATCTGTGTGGCTGTCACTCGGCTGCATGCTTAGTGCACTCA
hCoV-19/Denmark/DCGC-692245/2025|EPI_ISL_19879704|2025-04-28              -------------------------------------------------AAAATCTGTGTGGCTGTCACTCGGCTGCATGCTTAGTGCACTCA
```

Supplementary Figure S3. The representation of the 5′UTR region of SARS-CoV-2 genome (from 1 to 265 bases) considered in this paper.

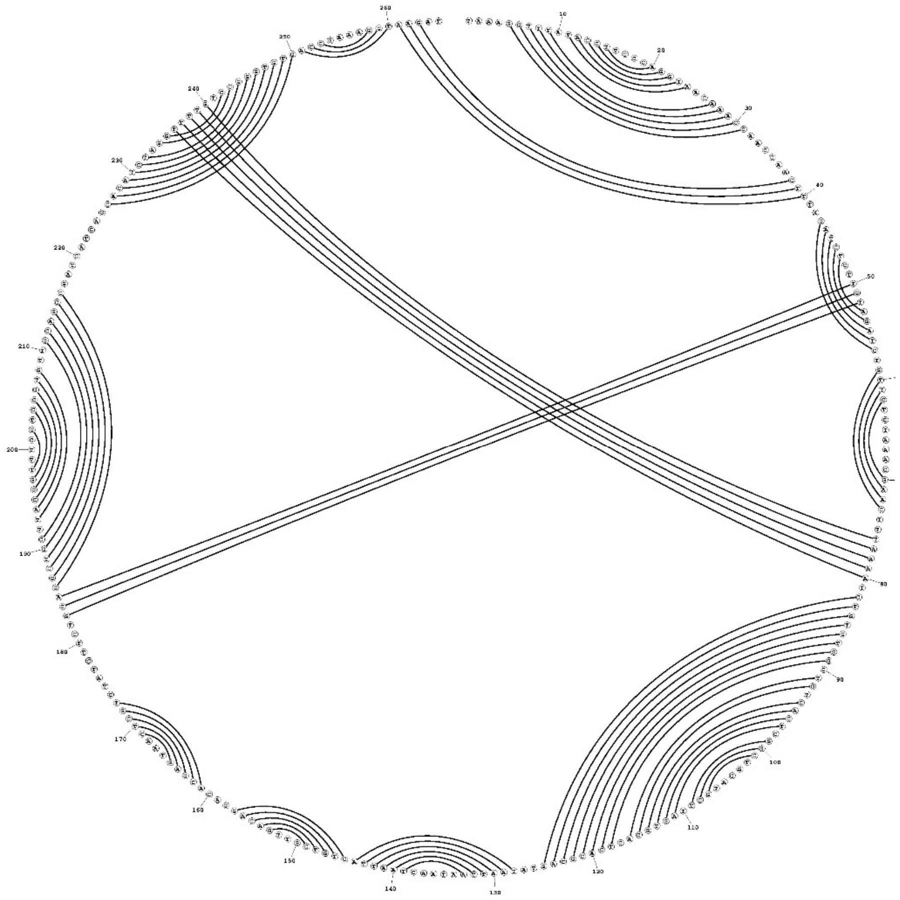

Supplementary Table S1:

hCoV-19/Ireland/D-SVUH-FBA98650-31/2025|EPI_ISL_19866548|2025-05-04	100.00	100.00	100.00	100.00	55.56	0.00	0.00	0.00	95.04	100.00	100.00	100.00	[illegible]
hCoV-19/Spain/PV-HUB-5628663/2025|EPI_ISL_19908839|2025-05-16	100.00	100.00	100.00	100.00	80.60	57.22	57.22	57.22	87.97	100.00	100.00	100.00	[illegible]
hCoV-19/Spain/PV-HUB-41587498/2025|EPI_ISL_19908930|2025-05-20	100.00	100.00	100.00	100.00	80.36	56.64	56.64	56.64	87.80	100.00	100.00	100.00	[illegible]
hCoV-19/Spain/PV-HUB-56291851/2025|EPI_ISL_19908970|2025-05-31	100.00	100.00	100.00	100.00	80.41	56.76	56.76	56.76	87.84	100.00	100.00	100.00	[illegible]
hCoV-19/Northern_Ireland/CLIMB-CM7YR8I6/2025|EPI_ISL_19877334|2025-04-23	55.56	80.60	80.36	80.41	100.00	82.22	82.22	82.22	84.73	86.13	86.34	87.62	[illegible]
hCoV-19/Germany/NW-RKI-I-1148136/2025|EPI_ISL_19871950|2025-04-23	0.00	57.22	56.64	56.76	82.22	100.00	100.00	100.00	80.54	79.49	79.67	80.81	[illegible]
hCoV-19/Germany/NW-RKI-I-1148141/2025|EPI_ISL_19871955|2025-04-23	0.00	57.22	56.64	56.76	82.22	100.00	100.00	100.00	80.54	79.49	79.67	80.81	[illegible]
hCoV-19/Germany/BW-RKI-I-1148164/2025|EPI_ISL_19884987|2025-04-17	0.00	57.22	56.64	56.76	82.22	100.00	100.00	100.00	80.54	79.49	79.67	80.81	[illegible]
hCoV-19/Scotland/CLIMB-CM7YFGW7/2025|EPI_ISL_19897691|2025-05-24	95.04	87.97	87.80	87.84	84.73	80.54	80.54	80.54	100.00	89.86	89.84	89.70	[illegible]
hCoV-19/Ireland/G-LUH-N3473/2025|EPI_ISL_19893181|2025-05-14	100.00	100.00	100.00	100.00	86.13	79.49	79.49	79.49	89.86	100.00	99.60	99.80	[illegible]
hCoV-19/Spain/AN-PMC-42728/2025|EPI_ISL_19888347|2025-05-25	100.00	100.00	100.00	100.00	86.34	79.67	79.67	79.67	89.84	99.60	100.00	99.39	[illegible]
hCoV-19/Sweden/000200866492N2/2025|EPI_ISL_19870050|2025-04-22	100.00	100.00	100.00	100.00	87.62	80.81	80.81	80.81	89.70	99.80	99.39	100.00	[illegible]
hCoV-19/Sweden/000200867159N2/2025|EPI_ISL_19870052|2025-04-23	100.00	100.00	100.00	100.00	87.62	80.81	80.81	80.81	89.70	99.80	99.39	100.00	[illegible]
hCoV-19/Ireland/G-LUH-N3445/2025|EPI_ISL_19893154|2025-05-07	100.00	100.00	100.00	100.00	87.62	80.81	80.81	80.81	89.70	100.00	99.60	99.80	[illegible]
hCoV-19/Ireland/G-LUH-N3443/2025|EPI_ISL_19893152|2025-05-07	100.00	100.00	100.00	100.00	87.19	80.42	80.42	80.42	89.74	100.00	99.60	99.80	[illegible]
hCoV-19/Scotland/CLIMB-CM7YF9PJ/2025|EPI_ISL_19895983|2025-05-19	100.00	100.00	100.00	100.00	86.98	80.24	80.24	80.24	89.77	99.80	99.80	99.60	[illegible]
hCoV-19/Scotland/CLIMB-CM7YKMK8/2025|EPI_ISL_19869976|2025-04-25	100.00	100.00	100.00	100.00	86.76	80.05	80.05	80.05	89.79	99.80	99.80	99.60	[illegible]
hCoV-19/Ireland/G-LUH-N3444/2025|EPI_ISL_19893153|2025-05-07	100.00	100.00	100.00	100.00	86.76	80.05	80.05	80.05	89.79	100.00	99.60	99.80	[illegible]
hCoV-19/Switzerland/VD-CHUV-8604552682/2025|EPI_ISL_19906527|2025-05-19	100.00	100.00	100.00	100.00	87.84	81.00	81.00	81.00	89.67	99.80	99.39	99.40	[illegible]
hCoV-19/Scotland/CLIMB-CM7YR3YO/2025|EPI_ISL_19896015|2025-05-24	100.00	100.00	[illegible]	[illegible]	[illegible]	[illegible]	[illegible]	[illegible]	[illegible]	[illegible]	[illegible]	[illegible]	[illegible]
hCoV-19/Sweden/O-18_SE100_25CS500180/2025|EPI_ISL_19866288|2025-04-15	100.00	100.00	[illegible]	[illegible]	[illegible]	[illegible]	[illegible]	[illegible]	[illegible]	[illegible]	[illegible]	[illegible]	[illegible]
hCoV-19/Ireland/D-CS250088/2025|EPI_ISL_19877457|2025-04-16	100.00	100.00	[illegible]	[illegible]	[illegible]	[illegible]	[illegible]	[illegible]	[illegible]	[illegible]	[illegible]	[illegible]	[illegible]
hCoV-19/Ireland/G-LUH-N3442/2025|EPI_ISL_19893151|2025-05-07	100.00	100.00	[illegible]	[illegible]	[illegible]	[illegible]	[illegible]	[illegible]	[illegible]	[illegible]	[illegible]	[illegible]	[illegible]
hCoV-19/Scotland/CLIMB-CM7YMZ5M/2025|EPI_ISL_19906439|2025-05-22	100.00	100.00	[illegible]	[illegible]	[illegible]	[illegible]	[illegible]	[illegible]	[illegible]	[illegible]	[illegible]	[illegible]	[illegible]
hCoV-19/Scotland/CLIMB-CM7YR3ZG/2025|EPI_ISL_19897722|2025-05-13	100.00	100.00	[illegible]	[illegible]	[illegible]	[illegible]	[illegible]	[illegible]	[illegible]	[illegible]	[illegible]	[illegible]	[illegible]
hCoV-19/Scotland/CLIMB-CM7YEYGT/2025|EPI_ISL_19897741|2025-04-28	100.00	100.00	[illegible]	[illegible]	[illegible]	[illegible]	[illegible]	[illegible]	[illegible]	[illegible]	[illegible]	[illegible]	[illegible]
hCoV-19/Scotland/CLIMB-CM7YGAJ3/2025|EPI_ISL_19895963|2025-05-13	100.00	100.00	[illegible]	[illegible]	[illegible]	[illegible]	[illegible]	[illegible]	[illegible]	[illegible]	[illegible]	[illegible]	[illegible]
hCoV-19/Scotland/CLIMB-CM7YJ1ZO/2025|EPI_ISL_19897757|2025-05-14	100.00	100.00	[illegible]	[illegible]	[illegible]	[illegible]	[illegible]	[illegible]	[illegible]	[illegible]	[illegible]	[illegible]	[illegible]
hCoV-19/Scotland/CLIMB-CM7Y8C4Y/2025|EPI_ISL_19906404|2025-05-15	100.00	100.00	[illegible]	[illegible]	[illegible]	[illegible]	[illegible]	[illegible]	[illegible]	[illegible]	[illegible]	[illegible]	[illegible]
hCoV-19/Northern_Ireland/CLIMB-CM7YGGYM/2025|EPI_ISL_19897641|2025-05-23	100.00	100.00	[illegible]	[illegible]	[illegible]	[illegible]	[illegible]	[illegible]	[illegible]	[illegible]	[illegible]	[illegible]	[illegible]
hCoV-19/Scotland/CLIMB-CM7YFW7M/2025|EPI_ISL_19881718|2025-05-10	100.00	100.00	[illegible]	[illegible]	[illegible]	[illegible]	[illegible]	[illegible]	[illegible]	[illegible]	[illegible]	[illegible]	[illegible]
hCoV-19/Scotland/CLIMB-CM7YEMXP/2025|EPI_ISL_19888387|2025-05-10	100.00	100.00	[illegible]	[illegible]	[illegible]	[illegible]	[illegible]	[illegible]	[illegible]	[illegible]	[illegible]	[illegible]	[illegible]
hCoV-19/Spain/CT-HUVH-E593511/2025|EPI_ISL_19881731|2025-05-06	65.00	90.08	[illegible]	[illegible]	[illegible]	[illegible]	[illegible]	[illegible]	[illegible]	[illegible]	[illegible]	[illegible]	[illegible]
hCoV-19/Spain/CT-HUJT-E325362/2025|EPI_ISL_19889935|2025-05-20	21.21	76.70	[illegible]	[illegible]	[illegible]	[illegible]	[illegible]	[illegible]	[illegible]	[illegible]	[illegible]	[illegible]	[illegible]
hCoV-19/Spain/PV-HUD-84743204/2025|EPI_ISL_19907746|2025-06-06	100.00	100.00	[illegible]	[illegible]	[illegible]	[illegible]	[illegible]	[illegible]	[illegible]	[illegible]	[illegible]	[illegible]	[illegible]
hCoV-19/Ireland/D-SVUH-FBB21492-54/2025|EPI_ISL_19881758|2025-05-11	100.00	100.00	[illegible]	[illegible]	[illegible]	[illegible]	[illegible]	[illegible]	[illegible]	[illegible]	[illegible]	[illegible]	[illegible]
hCoV-19/Spain/CT-HUVH-E596725/2025|EPI_ISL_19881729|2025-05-10	100.00	100.00	[illegible]	[illegible]	[illegible]	[illegible]	[illegible]	[illegible]	[illegible]	[illegible]	[illegible]	[illegible]	[illegible]
hCoV-19/Spain/CT-HUVH-E593882/2025|EPI_ISL_19881732|2025-05-06	100.00	100.00	[illegible]	[illegible]	[illegible]	[illegible]	[illegible]	[illegible]	[illegible]	[illegible]	[illegible]	[illegible]	[illegible]
hCoV-19/Finland/THL-02385/2025|EPI_ISL_19875928|2025-04-16	100.00	99.43	[illegible]	[illegible]	[illegible]	[illegible]	[illegible]	[illegible]	[illegible]	[illegible]	[illegible]	[illegible]	[illegible]
hCoV-19/Ireland/D-BH-02351167/2025|EPI_ISL_19911337|2025-05-28	100.00	100.00	[illegible]	[illegible]	[illegible]	[illegible]	[illegible]	[illegible]	[illegible]	[illegible]	[illegible]	[illegible]	[illegible]
hCoV-19/Ireland/D-NVRL-XIRL00001782/2025|EPI_ISL_19896104|2025-05-14	100.00	100.00	[illegible]	[illegible]	[illegible]	[illegible]	[illegible]	[illegible]	[illegible]	[illegible]	[illegible]	[illegible]	[illegible]
hCoV-19/Ireland/TA-NVRL-GIRL00039444/2025|EPI_ISL_19896118|2025-05-16	100.00	100.00	[illegible]	[illegible]	[illegible]	[illegible]	[illegible]	[illegible]	[illegible]	[illegible]	[illegible]	[illegible]	[illegible]
hCoV-19/Spain/GA-CHUAC-13223/2025|EPI_ISL_19894946|2025-05-31	100.00	99.72	[illegible]	[illegible]	[illegible]	[illegible]	[illegible]	[illegible]	[illegible]	[illegible]	[illegible]	[illegible]	[illegible]
hCoV-19/Spain/GA-CHUAC-13215/2025|EPI_ISL_19894939|2025-05-29	100.00	100.00	[illegible]	[illegible]	[illegible]	[illegible]	[illegible]	[illegible]	[illegible]	[illegible]	[illegible]	[illegible]	[illegible]
hCoV-19/Finland/THL-02398/2025|EPI_ISL_19875939|2025-05-04	100.00	100.00	[illegible]	[illegible]	[illegible]	[illegible]	[illegible]	[illegible]	[illegible]	[illegible]	[illegible]	[illegible]	[illegible]
hCoV-19/Finland/THL-02400/2025|EPI_ISL_19875941|2025-05-03	100.00	100.00	[illegible]	[illegible]	[illegible]	[illegible]	[illegible]	[illegible]	[illegible]	[illegible]	[illegible]	[illegible]	[illegible]
hCoV-19/Ireland/LK-UHL-702929/2025|EPI_ISL_19877895|2025-04-16	100.00	100.00	[illegible]	[illegible]	[illegible]	[illegible]	[illegible]	[illegible]	[illegible]	[illegible]	[illegible]	[illegible]	[illegible]
hCoV-19/Ireland/LK-UHL-703788/2025|EPI_ISL_19877905|2025-05-03	100.00	100.00	[illegible]	[illegible]	[illegible]	[illegible]	[illegible]	[illegible]	[illegible]	[illegible]	[illegible]	[illegible]	[illegible]
hCoV-19/Ireland/LK-UHL-703851/2025|EPI_ISL_19877907|2025-05-04	100.00	100.00	[illegible]	[illegible]	[illegible]	[illegible]	[illegible]	[illegible]	[illegible]	[illegible]	[illegible]	[illegible]	[illegible]
hCoV-19/Ireland/LK-UHL-703889/2025|EPI_ISL_19877908|2025-05-05	100.00	100.00	[illegible]	[illegible]	[illegible]	[illegible]	[illegible]	[illegible]	[illegible]	[illegible]	[illegible]	[illegible]	[illegible]
hCoV-19/Ireland/D-SVUH-FBB21492-49/2025|EPI_ISL_19881756|2025-05-14	100.00	100.00	[illegible]	[illegible]	[illegible]	[illegible]	[illegible]	[illegible]	[illegible]	[illegible]	[illegible]	[illegible]	[illegible]
hCoV-19/Ireland/D-SVUH-FBB21492-59/2025|EPI_ISL_19881763|2025-05-07	100.00	100.00	[illegible]	[illegible]	[illegible]	[illegible]	[illegible]	[illegible]	[illegible]	[illegible]	[illegible]	[illegible]	[illegible]
hCoV-19/Spain/CT-HUGTiP-G3R212/2025|EPI_ISL_19883055|2025-05-20	100.00	100.00	[illegible]	[illegible]	[illegible]	[illegible]	[illegible]	[illegible]	[illegible]	[illegible]	[illegible]	[illegible]	[illegible]
hCoV-19/Ireland/LK-UHL-704398/2025|EPI_ISL_19888621|2025-05-16	100.00	100.00	[illegible]	[illegible]	[illegible]	[illegible]	[illegible]	[illegible]	[illegible]	[illegible]	[illegible]	[illegible]	[illegible]
hCoV-19/Ireland/G-GUH-N3466/2025|EPI_ISL_19893175|2025-05-03	100.00	100.00	[illegible]	[illegible]	[illegible]	[illegible]	[illegible]	[illegible]	[illegible]	[illegible]	[illegible]	[illegible]	[illegible]
hCoV-19/Belgium/Sciensano-LS-S5276/2025|EPI_ISL_19893834|2025-04-22	100.00	100.00	[illegible]	[illegible]	[illegible]	[illegible]	[illegible]	[illegible]	[illegible]	[illegible]	[illegible]	[illegible]	[illegible]
hCoV-19/Italy/VEN-ULSS8-0019244_VI/2025|EPI_ISL_19899362|2025-05-26	100.00	100.00	[illegible]	[illegible]	[illegible]	[illegible]	[illegible]	[illegible]	[illegible]	[illegible]	[illegible]	[illegible]	[illegible]
hCoV-19/Spain/CT-HUVH-E618109/2025|EPI_ISL_19907773|2025-06-10	100.00	100.00	[illegible]	[illegible]	[illegible]	[illegible]	[illegible]	[illegible]	[illegible]	[illegible]	[illegible]	[illegible]	[illegible]
hCoV-19/Ireland/D-BH-03819149/2025|EPI_ISL_19911343|2025-05-29	100.00	100.00	[illegible]	[illegible]	[illegible]	[illegible]	[illegible]	[illegible]	[illegible]	[illegible]	[illegible]	[illegible]	[illegible]
hCoV-19/Ireland/D-BH-03993161/2025|EPI_ISL_19911355|2025-06-10	100.00	100.00	[illegible]	[illegible]	[illegible]	[illegible]	[illegible]	[illegible]	[illegible]	[illegible]	[illegible]	[illegible]	[illegible]
hCoV-19/Spain/CT-HUVH-E587040/2025|EPI_ISL_19871881|2025-04-26	100.00	100.00	[illegible]	[illegible]	[illegible]	[illegible]	[illegible]	[illegible]	[illegible]	[illegible]	[illegible]	[illegible]	[illegible]
hCoV-19/Finland/THL-02392/2025|EPI_ISL_19875934|2025-05-02	100.00	100.00	[illegible]	[illegible]	[illegible]	[illegible]	[illegible]	[illegible]	[illegible]	[illegible]	[illegible]	[illegible]	[illegible]
hCoV-19/Spain/GA-CHUAC-13181/2025|EPI_ISL_19890617|2025-05-20	100.00	100.00	[illegible]	[illegible]	[illegible]	[illegible]	[illegible]	[illegible]	[illegible]	[illegible]	[illegible]	[illegible]	[illegible]
hCoV-19/Spain/CT-HUVH-E614843/2025|EPI_ISL_19907763|2025-06-05	100.00	100.00	[illegible]	[illegible]	[illegible]	[illegible]	[illegible]	[illegible]	[illegible]	[illegible]	[illegible]	[illegible]	[illegible]
hCoV-19/Italy/UMB-LAOP-172-4-02/2025|EPI_ISL_19899474|2025-05-08	100.00	100.00	[illegible]	[illegible]	[illegible]	[illegible]	[illegible]	[illegible]	[illegible]	[illegible]	[illegible]	[illegible]	[illegible]
hCoV-19/Spain/NC-CHN-01008932/2025|EPI_ISL_19900595|2025-04-04	100.00	100.00	[illegible]	[illegible]	[illegible]	[illegible]	[illegible]	[illegible]	[illegible]	[illegible]	[illegible]	[illegible]	[illegible]
hCoV-19/Northern_Ireland/CLIMB-CM7YGDRO/2025|EPI_ISL_19877331|2025-04-14	100.00	100.00	[illegible]	[illegible]	[illegible]	[illegible]	[illegible]	[illegible]	[illegible]	[illegible]	[illegible]	[illegible]	[illegible]
hCoV-19/Scotland/CLIMB-CM7YEE96/2025|EPI_ISL_19897754|2025-05-13	100.00	100.00	[illegible]	[illegible]	[illegible]	[illegible]	[illegible]	[illegible]	[illegible]	[illegible]	[illegible]	[illegible]	[illegible]
hCoV-19/France/ARA-HCL125018289001/2025|EPI_ISL_19880005|2025-05-12	100.00	100.00	[illegible]	[illegible]	[illegible]	[illegible]	[illegible]	[illegible]	[illegible]	[illegible]	[illegible]	[illegible]	[illegible]
hCoV-19/France/ARA-RELAB-HCL725000057501/2025|EPI_ISL_19880014|2025-04-28	100.00	100.00	[illegible]	[illegible]	[illegible]	[illegible]	[illegible]	[illegible]	[illegible]	[illegible]	[illegible]	[illegible]	[illegible]
hCoV-19/France/ARA-RELAB-HCL725000059501/2025|EPI_ISL_19885160|2025-04-25	100.00	100.00	[illegible]	[illegible]	[illegible]	[illegible]	[illegible]	[illegible]	[illegible]	[illegible]	[illegible]	[illegible]	[illegible]
hCoV-19/France/NAQ-RELAB-HCL725000061001/2025|EPI_ISL_19885161|2025-05-10	100.00	100.00	[illegible]	[illegible]	[illegible]	[illegible]	[illegible]	[illegible]	[illegible]	[illegible]	[illegible]	[illegible]	[illegible]
hCoV-19/France/NAQ-RELAB-HCL725000060901/2025|EPI_ISL_19885180|2025-05-09	100.00	100.00	[illegible]	[illegible]	[illegible]	[illegible]	[illegible]	[illegible]	[illegible]	[illegible]	[illegible]	[illegible]	[illegible]
hCoV-19/France/ARA-HCL025075546601/2025|EPI_ISL_19885182|2025-05-09	100.00	100.00	[illegible]	[illegible]	[illegible]	[illegible]	[illegible]	[illegible]	[illegible]	[illegible]	[illegible]	[illegible]	[illegible]
hCoV-19/France/ARA-HCL125018461901/2025|EPI_ISL_19885204|2025-05-14	100.00	100.00	[illegible]	[illegible]	[illegible]	[illegible]	[illegible]	[illegible]	[illegible]	[illegible]	[illegible]	[illegible]	[illegible]
hCoV-19/France/ARA-HCL125018655901/2025|EPI_ISL_19885206|2025-05-14	100.00	100.00	[illegible]	[illegible]	[illegible]	[illegible]	[illegible]	[illegible]	[illegible]	[illegible]	[illegible]	[illegible]	[illegible]
hCoV-19/France/ARA-HCL125019155501/2025|EPI_ISL_19885209|2025-05-18	100.00	100.00	[illegible]	[illegible]	[illegible]	[illegible]	[illegible]	[illegible]	[illegible]	[illegible]	[illegible]	[illegible]	[illegible]
hCoV-19/France/ARA-HCL125019240901/2025|EPI_ISL_19885210|2025-05-19	100.00	100.00	[illegible]	[illegible]	[illegible]	[illegible]	[illegible]	[illegible]	[illegible]	[illegible]	[illegible]	[illegible]	[illegible]
hCoV-19/France/ARA-HCL125019249201/2025|EPI_ISL_19885211|2025-05-19	100.00	100.00	[illegible]	[illegible]	[illegible]	[illegible]	[illegible]	[illegible]	[illegible]	[illegible]	[illegible]	[illegible]	[illegible]
hCoV-19/France/ARA-HCL125019399701/2025|EPI_ISL_19885212|2025-05-20	100.00	100.00	[illegible]	[illegible]	[illegible]	[illegible]	[illegible]	[illegible]	[illegible]	[illegible]	[illegible]	[illegible]	[illegible]
hCoV-19/France/ARA-HCL125019408901/2025|EPI_ISL_19885213|2025-05-20	100.00	100.00	[illegible]	[illegible]	[illegible]	[illegible]	[illegible]	[illegible]	[illegible]	[illegible]	[illegible]	[illegible]	[illegible]
hCoV-19/France/OCC-RELAB-HCL725000064001/2025|EPI_ISL_19895004|2025-05-14	100.00	100.00	[illegible]	[illegible]	[illegible]	[illegible]	[illegible]	[illegible]	[illegible]	[illegible]	[illegible]	[illegible]	[illegible]
hCoV-19/France/OCC-RELAB-HCL725000064101/2025|EPI_ISL_19895005|2025-05-14	100.00	100.00	[illegible]	[illegible]	[illegible]	[illegible]	[illegible]	[illegible]	[illegible]	[illegible]	[illegible]	[illegible]	[illegible]
hCoV-19/France/NAQ-RELAB-HCL725000062601/2025|EPI_ISL_19895009|2025-05-16	100.00	100.00	[illegible]	[illegible]	[illegible]	[illegible]	[illegible]	[illegible]	[illegible]	[illegible]	[illegible]	[illegible]	[illegible]
hCoV-19/France/ARA-RELAB-HCL725000062401/2025|EPI_ISL_19895017|2025-05-16	100.00	100.00	[illegible]	[illegible]	[illegible]	[illegible]	[illegible]	[illegible]	[illegible]	[illegible]	[illegible]	[illegible]	[illegible]
hCoV-19/France/ARA-RELAB-HCL725000062101/2025|EPI_ISL_19895018|2025-05-13	100.00	100.00	[illegible]	[illegible]	[illegible]	[illegible]	[illegible]	[illegible]	[illegible]	[illegible]	[illegible]	[illegible]	[illegible]
hCoV-19/France/ARA-HCL125019625101/2025|EPI_ISL_19895034|2025-05-21	100.00	100.00	[illegible]	[illegible]	[illegible]	[illegible]	[illegible]	[illegible]	[illegible]	[illegible]	[illegible]	[illegible]	[illegible]
hCoV-19/France/ARA-HCL125019754801/2025|EPI_ISL_19895035|2025-05-22	100.00	100.00	[illegible]	[illegible]	[illegible]	[illegible]	[illegible]	[illegible]	[illegible]	[illegible]	[illegible]	[illegible]	[illegible]
hCoV-19/France/ARA-RELAB-HCL725000064601/2025|EPI_ISL_19895041|2025-05-15	100.00	100.00	[illegible]	[illegible]	[illegible]	[illegible]	[illegible]	[illegible]	[illegible]	[illegible]	[illegible]	[illegible]	[illegible]
hCoV-19/France/ARA-RELAB-HCL725000066001/2025|EPI_ISL_19895046|2025-05-13	100.00	100.00	[illegible]	[illegible]	[illegible]	[illegible]	[illegible]	[illegible]	[illegible]	[illegible]	[illegible]	[illegible]	[illegible]
hCoV-19/France/OCC-RELAB-HCL725000068701/2025|EPI_ISL_19905466|2025-05-21	100.00	100.00	[illegible]	[illegible]	[illegible]	[illegible]	[illegible]	[illegible]	[illegible]	[illegible]	[illegible]	[illegible]	[illegible]
hCoV-19/France/OCC-RELAB-HCL725000069001/2025|EPI_ISL_19905468|2025-05-21	100.00	100.00	[illegible]	[illegible]	[illegible]	[illegible]	[illegible]	[illegible]	[illegible]	[illegible]	[illegible]	[illegible]	[illegible]
hCoV-19/France/ARA-HCL025089279801/2025|EPI_ISL_19905469|2025-06-02	100.00	100.00	[illegible]	[illegible]	[illegible]	[illegible]	[illegible]	[illegible]	[illegible]	[illegible]	[illegible]	[illegible]	[illegible]
hCoV-19/France/PAC-RELAB-HCL725000069901/2025|EPI_ISL_19908847|2025-05-26	100.00	100.00	[illegible]	[illegible]	[illegible]	[illegible]	[illegible]	[illegible]	[illegible]	[illegible]	[illegible]	[illegible]	[illegible]
hCoV-19/France/PAC-RELAB-HCL725000069501/2025|EPI_ISL_19908849|2025-05-30	100.00	100.00	[illegible]	[illegible]	[illegible]	[illegible]	[illegible]	[illegible]	[illegible]	[illegible]	[illegible]	[illegible]	[illegible]
hCoV-19/France/ARA-RELAB-HCL725000071701/2025|EPI_ISL_19908858|2025-05-22	100.00	100.00	[illegible]	[illegible]	[illegible]	[illegible]	[illegible]	[illegible]	[illegible]	[illegible]	[illegible]	[illegible]	[illegible]
hCoV-19/France/ARA-HCL025093352901/2025|EPI_ISL_19908867|2025-06-10	100.00	100.00	[illegible]	[illegible]	[illegible]	[illegible]	[illegible]	[illegible]	[illegible]	[illegible]	[illegible]	[illegible]	[illegible]
hCoV-19/France/ARA-RELAB-HCL725000065801/2025|EPI_ISL_19895045|2025-05-16	100.00	99.71	[illegible]	[illegible]	[illegible]	[illegible]	[illegible]	[illegible]	[illegible]	[illegible]	[illegible]	[illegible]	[illegible]
hCoV-19/Denmark/DCGC-692234/2025|EPI_ISL_19879694|2025-05-05	97.80	99.42	[illegible]	[illegible]	[illegible]	[illegible]	[illegible]	[illegible]	[illegible]	[illegible]	[illegible]	[illegible]	[illegible]
hCoV-19/Finland/THL-02388/2025|EPI_ISL_19875931|2025-04-16	100.00	100.00	[illegible]	[illegible]	[illegible]	[illegible]	[illegible]	[illegible]	[illegible]	[illegible]	[illegible]	[illegible]	[illegible]
hCoV-19/Spain/PV-HUD-98024244/2025|EPI_ISL_19907759|2025-06-09	100.00	100.00	[illegible]	[illegible]	[illegible]	[illegible]	[illegible]	[illegible]	[illegible]	[illegible]	[illegible]	[illegible]	[illegible]
hCoV-19/Netherlands/GE-RIVM-145386/2025|EPI_ISL_19873764|2025-05-01	100.00	100.00	[illegible]	[illegible]	[illegible]	[illegible]	[illegible]	[illegible]	[illegible]	[illegible]	[illegible]	[illegible]	[illegible]
hCoV-19/Netherlands/ZH-RIVM-145413/2025|EPI_ISL_19891397|2025-04-30	100.00	100.00	[illegible]	[illegible]	[illegible]	[illegible]	[illegible]	[illegible]	[illegible]	[illegible]	[illegible]	[illegible]	[illegible]
hCoV-19/Netherlands/NH-RIVM-145458/2025|EPI_ISL_19905062|2025-05-25	100.00	100.00	[illegible]	[illegible]	[illegible]	[illegible]	[illegible]	[illegible]	[illegible]	[illegible]	[illegible]	[illegible]	[illegible]
hCoV-19/Netherlands/NH-RIVM-145463/2025|EPI_ISL_19905063|2025-05-20	100.00	100.00	[illegible]	[illegible]	[illegible]	[illegible]	[illegible]	[illegible]	[illegible]	[illegible]	[illegible]	[illegible]	[illegible]
hCoV-19/Netherlands/UT-RIVM-145436/2025|EPI_ISL_19905072|2025-05-15	100.00	100.00	[illegible]	[illegible]	[illegible]	[illegible]	[illegible]	[illegible]	[illegible]	[illegible]	[illegible]	[illegible]	[illegible]
hCoV-19/Netherlands/UT-RIVM-145470/2025|EPI_ISL_19905077|2025-06-02	100.00	100.00	[illegible]	[illegible]	[illegible]	[illegible]	[illegible]	[illegible]	[illegible]	[illegible]	[illegible]	[illegible]	[illegible]
hCoV-19/Netherlands/ZH-RIVM-145478/2025|EPI_ISL_19905094|2025-05-30	100.00	100.00	[illegible]	[illegible]	[illegible]	[illegible]	[illegible]	[illegible]	[illegible]	[illegible]	[illegible]	[illegible]	[illegible]
hCoV-19/Netherlands/ZH-RIVM-145480/2025|EPI_ISL_19905096|2025-05-31	100.00	100.00	[illegible]	[illegible]	[illegible]	[illegible]	[illegible]	[illegible]	[illegible]	[illegible]	[illegible]	[illegible]	[illegible]
hCoV-19/Netherlands/UT-RIVM-145481/2025|EPI_ISL_19905078|2025-05-31	100.00	100.00	[illegible]	[illegible]	[illegible]	[illegible]	[illegible]	[illegible]	[illegible]	[illegible]	[illegible]	[illegible]	[illegible]
hCoV-19/Scotland/CLIMB-CM7YRW9H/2025|EPI_ISL_19881711|2025-05-06	100.00	100.00	[illegible]	[illegible]	[illegible]	[illegible]	[illegible]	[illegible]	[illegible]	[illegible]	[illegible]	[illegible]	[illegible]
hCoV-19/France/IDF-RELAB-IPP04872/2025|EPI_ISL_19911293|2025-05-22	100.00	100.00	[illegible]	[illegible]	[illegible]	[illegible]	[illegible]	[illegible]	[illegible]	[illegible]	[illegible]	[illegible]	[illegible]
hCoV-19/Canary_Islands/CN-HUC-925112913/2025|EPI_ISL_19900668|2025-04-22	100.00	100.00	[illegible]	[illegible]	[illegible]	[illegible]	[illegible]	[illegible]	[illegible]	[illegible]	[illegible]	[illegible]	[illegible]
hCoV-19/Scotland/CLIMB-CM7YJ5BB/2025|EPI_ISL_19906458|2025-05-27	100.00	100.00	[illegible]	[illegible]	[illegible]	[illegible]	[illegible]	[illegible]	[illegible]	[illegible]	[illegible]	[illegible]	[illegible]
hCoV-19/Scotland/CLIMB-CM7Y8PHH/2025|EPI_ISL_19897755|2025-05-14	100.00	100.00	[illegible]	[illegible]	[illegible]	[illegible]	[illegible]	[illegible]	[illegible]	[illegible]	[illegible]	[illegible]	[illegible]
hCoV-19/Spain/AN-PMC-42706/2025|EPI_ISL_19888325|2025-05-25	100.00	99.73	[illegible]	[illegible]	[illegible]	[illegible]	[illegible]	[illegible]	[illegible]	[illegible]	[illegible]	[illegible]	[illegible]
hCoV-19/Scotland/CLIMB-CM7YRIA1/2025|EPI_ISL_19869961|2025-04-20	100.00	100.00	[illegible]	[illegible]	[illegible]	[illegible]	[illegible]	[illegible]	[illegible]	[illegible]	[illegible]	[illegible]	[illegible]
hCoV-19/Scotland/CLIMB-CM7Y8UWT/2025|EPI_ISL_19869966|2025-04-21	100.00	100.00	[illegible]	[illegible]	[illegible]	[illegible]	[illegible]	[illegible]	[illegible]	[illegible]	[illegible]	[illegible]	[illegible]
hCoV-19/England/CLIMB-CM7YMFHB/2025|EPI_ISL_19877436|2025-05-10	100.00	100.00	[illegible]	[illegible]	[illegible]	[illegible]	[illegible]	[illegible]	[illegible]	[illegible]	[illegible]	[illegible]	[illegible]
hCoV-19/Denmark/DCGC-692213/2025|EPI_ISL_19879673|2025-04-28	100.00	100.00	[illegible]	[illegible]	[illegible]	[illegible]	[illegible]	[illegible]	[illegible]	[illegible]	[illegible]	[illegible]	[illegible]
hCoV-19/Wales/CLIMB-CM7YRAXQ/2025|EPI_ISL_19883075|2025-05-14	100.00	100.00	[illegible]	[illegible]	[illegible]	[illegible]	[illegible]	[illegible]	[illegible]	[illegible]	[illegible]	[illegible]	[illegible]
hCoV-19/Spain/AN-PMC-42696/2025|EPI_ISL_19888316|2025-05-14	100.00	100.00	[illegible]	[illegible]	[illegible]	[illegible]	[illegible]	[illegible]	[illegible]	[illegible]	[illegible]	[illegible]	[illegible]
hCoV-19/Spain/AN-PMC-42719/2025|EPI_ISL_19888338|2025-05-15	100.00	100.00	[illegible]	[illegible]	[illegible]	[illegible]	[illegible]	[illegible]	[illegible]	[illegible]	[illegible]	[illegible]	[illegible]
hCoV-19/England/CLIMB-CM7YE7HB/2025|EPI_ISL_19892115|2025-05-19	100.00	100.00	[illegible]	[illegible]	[illegible]	[illegible]	[illegible]	[illegible]	[illegible]	[illegible]	[illegible]	[illegible]	[illegible]
hCoV-19/France/GES-RELAB-IPP04599/2025|EPI_ISL_19893547|2025-05-05	100.00	100.00	[illegible]	[illegible]	[illegible]	[illegible]	[illegible]	[illegible]	[illegible]	[illegible]	[illegible]	[illegible]	[illegible]
hCoV-19/Scotland/CLIMB-CM7YEPXZ/2025|EPI_ISL_19896028|2025-05-25	100.00	100.00	[illegible]	[illegible]	[illegible]	[illegible]	[illegible]	[illegible]	[illegible]	[illegible]	[illegible]	[illegible]	[illegible]
hCoV-19/Scotland/CLIMB-CM7YF4QT/2025|EPI_ISL_19896032|2025-05-27	100.00	100.00	[illegible]	[illegible]	[illegible]	[illegible]	[illegible]	[illegible]	[illegible]	[illegible]	[illegible]	[illegible]	[illegible]
hCoV-19/Scotland/CLIMB-CM7YKDZ6/2025|EPI_ISL_19896033|2025-05-27	100.00	100.00	[illegible]	[illegible]	[illegible]	[illegible]	[illegible]	[illegible]	[illegible]	[illegible]	[illegible]	[illegible]	[illegible]
hCoV-19/Scotland/CLIMB-CM7YG8H4/2025|EPI_ISL_19897748|2025-05-02	100.00	100.00	[illegible]	[illegible]	[illegible]	[illegible]	[illegible]	[illegible]	[illegible]	[illegible]	[illegible]	[illegible]	[illegible]
hCoV-19/Wales/CLIMB-CM7YJXNZ/2025|EPI_ISL_19900036|2025-05-26	100.00	100.00	[illegible]	[illegible]	[illegible]	[illegible]	[illegible]	[illegible]	[illegible]	[illegible]	[illegible]	[illegible]	[illegible]
hCoV-19/Germany/NW-RKI-I-1148178/2025|EPI_ISL_19902055|2025-05-21	100.00	100.00	[illegible]	[illegible]	[illegible]	[illegible]	[illegible]	[illegible]	[illegible]	[illegible]	[illegible]	[illegible]	[illegible]
hCoV-19/Germany/NI-RKI-I-1148183/2025|EPI_ISL_19902060|2025-05-18	100.00	100.00	[illegible]	[illegible]	[illegible]	[illegible]	[illegible]	[illegible]	[illegible]	[illegible]	[illegible]	[illegible]	[illegible]
hCoV-19/Germany/NW-RKI-I-1148191/2025|EPI_ISL_19902068|2025-05-19	100.00	100.00	[illegible]	[illegible]	[illegible]	[illegible]	[illegible]	[illegible]	[illegible]	[illegible]	[illegible]	[illegible]	[illegible]
hCoV-19/Spain/MD-HGUGM-66414184/2025|EPI_ISL_19906298|2025-05-25	100.00	100.00	[illegible]	[illegible]	[illegible]	[illegible]	[illegible]	[illegible]	[illegible]	[illegible]	[illegible]	[illegible]	[illegible]
hCoV-19/Spain/MD-HGUGM-66400167/2025|EPI_ISL_19906320|2025-05-22	100.00	100.00	[illegible]	[illegible]	[illegible]	[illegible]	[illegible]	[illegible]	[illegible]	[illegible]	[illegible]	[illegible]	[illegible]
hCoV-19/France/IDF-RELAB-IPP04646/2025|EPI_ISL_19907194|2025-05-16	100.00	100.00	[illegible]	[illegible]	[illegible]	[illegible]	[illegible]	[illegible]	[illegible]	[illegible]	[illegible]	[illegible]	[illegible]
hCoV-19/England/CLIMB-CM7YKIUQ/2025|EPI_ISL_19908573|2025-06-07	100.00	100.00	[illegible]	[illegible]	[illegible]	[illegible]	[illegible]	[illegible]	[illegible]	[illegible]	[illegible]	[illegible]	[illegible]
hCoV-19/France/GES-RELAB-IPP04823/2025|EPI_ISL_19911257|2025-05-26	100.00	100.00	[illegible]	[illegible]	[illegible]	[illegible]	[illegible]	[illegible]	[illegible]	[illegible]	[illegible]	[illegible]	[illegible]
hCoV-19/France/IDF-RELAB-IPP04863/2025|EPI_ISL_19911265|2025-05-24	100.00	100.00	[illegible]	[illegible]	[illegible]	[illegible]	[illegible]	[illegible]	[illegible]	[illegible]	[illegible]	[illegible]	[illegible]
hCoV-19/Denmark/DCGC-692245/2025|EPI_ISL_19879704|2025-04-28	99.17	99.73	[illegible]	[illegible]	[illegible]	[illegible]	[illegible]	[illegible]	[illegible]	[illegible]	[illegible]	[illegible]	[illegible]

0.00	100.00	100.00	100.00	100.00	100.00	100.00	100.00	100.00	100.00	100.00	100.00	100.00	100.00	100.00	100.00	100.00	100.00	100.00	100.00
0.00	100.00	100.00	100.00	100.00	100.00	100.00	100.00	100.00	100.00	100.00	100.00	100.00	100.00	100.00	100.00	100.00	100.00	100.00	100.00
0.00	100.00	100.00	100.00	100.00	100.00	100.00	100.00	100.00	100.00	100.00	100.00	100.00	100.00	100.00	100.00	100.00	100.00	100.00	100.00
0.00	100.00	100.00	100.00	100.00	100.00	100.00	100.00	100.00	100.00	100.00	100.00	100.00	100.00	100.00	100.00	100.00	100.00	100.00	100.00
7.03	87.25	87.25	87.25	86.61	86.54	86.91	86.94	87.11	87.11	87.11	87.11	87.11	87.11	87.11	87.11	87.11	87.11	87.11	87.11
3.91	74.13	74.13	74.13	76.46	76.80	75.44	75.28	74.62	74.62	74.62	74.62	74.62	74.62	74.62	74.62	74.62	74.62	74.62	74.62
3.91	74.13	74.13	74.13	76.46	76.80	75.44	75.28	74.62	74.62	74.62	74.62	74.62	74.62	74.62	74.62	74.62	74.62	74.62	74.62
3.91	74.13	74.13	74.13	76.46	76.80	75.44	75.28	74.62	74.62	74.62	74.62	74.62	74.62	74.62	74.62	74.62	74.62	74.62	74.62
0.38	90.59	90.59	90.59	90.13	90.09	90.34	90.36	90.49	90.49	90.49	90.49	90.49	90.49	90.49	90.49	90.49	90.49	90.49	90.49
9.78	100.00	100.00	100.00	99.79	99.79	99.36	100.00	99.57	99.57	99.57	99.57	99.57	99.57	99.57	99.57	99.57	99.57	99.57	99.57
9.56	99.78	99.78	99.78	99.37	99.38	99.57	99.79	99.78	99.78	99.78	99.78	99.78	99.78	99.78	99.78	99.78	99.78	99.78	99.78
9.78	100.00	100.00	100.00	100.00	100.00	99.35	100.00	99.56	99.56	99.56	99.56	99.56	99.56	99.56	99.56	99.56	99.56	99.56	99.56
9.78	100.00	100.00	100.00	100.00	100.00	99.35	100.00	99.56	99.56	99.56	99.56	99.56	99.56	99.56	99.56	99.56	99.56	99.56	99.56
9.78	100.00	100.00	100.00	99.79	99.79	99.35	100.00	99.56	99.56	99.56	99.56	99.56	99.56	99.56	99.56	99.56	99.56	99.56	99.56
9.78	100.00	100.00	100.00	99.79	99.79	99.35	100.00	99.56	99.56	99.56	99.56	99.56	99.56	99.56	99.56	99.56	99.56	99.56	99.56
9.56	99.78	99.78	99.78	99.58	99.58	99.57	99.78	99.78	99.78	99.78	99.78	99.78	99.78	99.78	99.78	99.78	99.78	99.78	99.78
9.56	99.78	99.78	99.78	99.58	99.58	99.57	99.78	99.78	99.78	99.78	99.78	99.78	99.78	99.78	99.78	99.78	99.78	99.78	99.78
9.78	100.00	100.00	100.00	99.79	99.79	99.36	100.00	99.56	99.56	99.56	99.56	99.56	99.56	99.56	99.56	99.56	99.56	99.56	99.56
9.78	100.00	100.00	100.00	99.79	99.79	99.35	100.00	99.56	99.56	99.56	99.56	99.56	99.56	99.56	99.56	99.56	99.56	99.56	99.56
